# Widespread shifts in the coastal biota of northern California during the 2014–2016 marine heatwaves

**DOI:** 10.1038/s41598-019-40784-3

**Published:** 2019-03-12

**Authors:** Eric Sanford, Jacqueline L. Sones, Marisol García-Reyes, Jeffrey H. R. Goddard, John L. Largier

**Affiliations:** 1 Bodega Marine Laboratory, University of California, Davis, Bodega Bay, California 94923 USA; 2 Department of Evolution and Ecology, University of California, Davis, California 95616 USA; 3 Bodega Marine Reserve, University of California, Davis, Bodega Bay, California 94923 USA; 4 Farallon Institute, 101 H Street, Suite Q, Petaluma, California 94952 USA; 5 Marine Science Institute, University of California, Santa Barbara, California 93106 USA; 6 Department of Environmental Science and Policy, University of California, Davis, California 95616 USA

## Abstract

During 2014–2016, severe marine heatwaves in the northeast Pacific triggered well-documented disturbances including mass mortalities, harmful algal blooms, and declines in subtidal kelp beds. However, less attention has been directed towards understanding how changes in sea surface temperature (SST) and alongshore currents during this period influenced the geographic distribution of coastal taxa. Here, we examine these effects in northern California, USA, with a focus on the region between Point Reyes and Point Arena. This region represents an important biogeographic transition zone that lies <150 km north of Monterey Bay, California, where numerous southern species have historically reached their northern (poleward) range limits. We report substantial changes in geographic distributions and/or abundances across a diverse suite of 67 southern species, including an unprecedented number of poleward range extensions (37) and striking increases in the recruitment of owl limpets (*Lottia gigantea*) and volcano barnacles (*Tetraclita rubescens*). These ecological responses likely arose through the combined effects of extreme SST, periods of anomalous poleward flow, and the unusually long duration of heatwave events. Prolonged marine heatwaves and enhanced poleward dispersal may play an important role in longer-term shifts in the composition of coastal communities in northern California and other biogeographic transition zones.

## Introduction

Marine heatwaves are defined as periods of extreme sea surface temperature (SST) persisting for days to months^1,2^. Although the ecological consequences of gradual, long-term increases in mean SST have generally received greater attention^3–5^, a series of recent marine heatwaves has highlighted the importance of extreme events in coastal ecosystems^2,6^. For example, extreme SST events in the Mediterranean Sea and Australia have been associated with extensive disturbances in subtidal communities including mass mortalities, coral bleaching, loss of habitat-forming kelps, and cascading ecological effects^7–10^. Marine heatwaves have increased in frequency and duration over the past century^11^, prompting calls for increased study of their ecological effects^1,2,11^.

During 2014–2016, the northeast Pacific experienced an unprecedented period of anomalously warm water, which has been characterized as the largest marine heatwave on record^12^. Oceanographic changes began during the winter of 2013–2014 in the Gulf of Alaska with the formation of a large warm-water anomaly, also referred to as the “warm-water Blob”^13^. By Fall 2014, this warm water had spread south and influenced much of the California Current Large Marine Ecosystem (CCLME). Subsequently, a strong El Niño event developed in the equatorial Pacific in 2015, with warm water spreading poleward into the northeast Pacific^12,14^. The combination of these events in the CCLME during 2014–2016 led to persistent sea surface temperature (SST) anomalies that were often 2–4 °C above mean climatological values^15^.

A broad range of ecological disturbances was observed in the CCLME during 2014–2016 including disruption of pelagic food webs and mortality of seabirds and marine mammals^12,16,17^. There were also striking declines in the abundance of subtidal kelp in northern California with cascading negative impacts on red abalone populations, which prompted closure of the recreational abalone fishery in 2018^18^. A toxic algal bloom of unprecedented extent and severity also occurred throughout much of the CCLME, leading to the closure of an important commercial crab fishery^19^. Although it has been suggested that this marine heatwave might have been the most ecologically significant in recorded history^12,17^, documentation of these ecological effects has been incomplete. In particular, there has been relatively little consideration of numerous shifts in the geographic distribution and abundance of coastal species in the CCLME during 2014–2016^19,20^.

During past strong El Niño events, marine species have often been documented poleward of their typical geographic ranges in the CCLME^21–23^. However, prior studies of these ecological effects in the CCLME have been limited with regards to habitat, taxonomic scope, and geographic region. Most studies to date have examined responses in pelagic ecosystems and have focused primarily on fish and other vertebrates^19,23–26^, with some consideration of shifts in the composition of zooplankton communities^27,28^. The few studies that have considered the influence of El Niño events on benthic intertidal communities in the CCLME have suggested that effects on these coastal ecosystems may be negligible^29,30^. Geographically, there has been a strong focus on effects of warm-water events in the Southern California Bight^30–32^, with some studies in Oregon and Washington state^26,28^, but few studies in northern California.

The paucity of studies of marine heatwaves in northern California is a significant gap as many coastal species with southern biogeographic affinities reach their northern (poleward) range limits in the CCLME at Monterey Bay, California^33–35^. Thus, northern California is an important transition zone between the mild-temperate taxa of the Montereyan biogeographic province (ranging from Point Conception in southern California to Monterey Bay) and the cool-temperate taxa of the Mendocinian province (ranging from Monterey Bay north to Cape Flattery, Washington^33,36^). Such transition zones are considered ideal barometers for documenting geographic range expansions and shifts in community composition as species from lower latitudes disperse poleward in response to warmer water^5,37,38^. Indeed, recent decades have seen a global trend towards tropicalization, as warm-adapted species disperse further poleward and increase in abundance in well-studied transition zones such as those found along the coasts of Europe and Australia^8,39,40^. It is increasingly clear that such shifts in community composition often involve not only increased SST, but also changes in the oceanographic currents that transport larvae and other reproductive propagules poleward^8,41–44^. Documenting the effects of marine heatwaves on the distribution of taxa in biogeographic transition zones thus provides a lens through which to advance our understanding of alongshore transport, dispersal, geographic range expansions, and the influence of climate change on coastal ecosystems.

**Figure 1:**
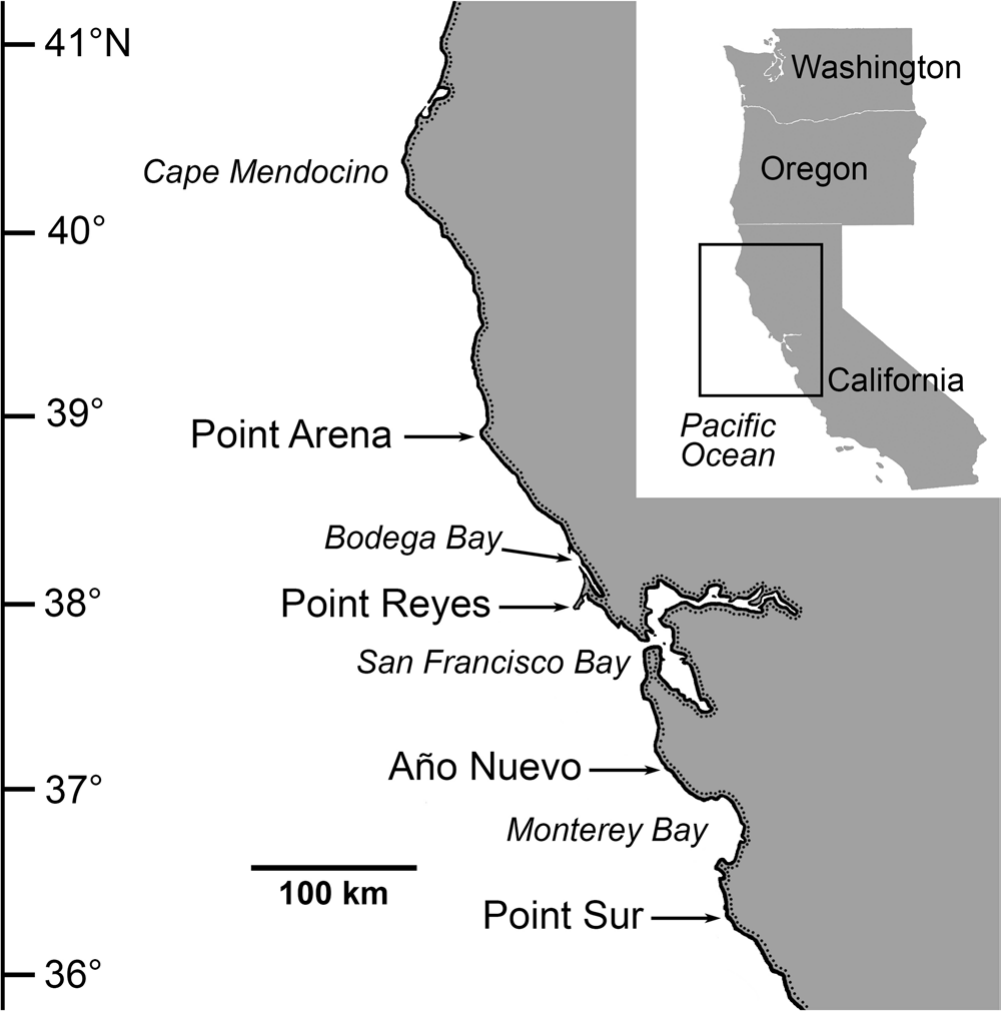
Primary study region in northern California, USA. Inset map (upper right) shows location of study region along the Pacific coast of the United States.

Here, we document oceanographic changes and associated shifts in the distribution of coastal biota in northern California, USA, during the marine heatwaves of 2014–2016. Our primary study region extended from Point Reyes to Point Arena (Fig. 1), although we include some records of southern taxa transported farther north, to regions ranging from northern California to British Columbia, Canada. We analyze SST and current anomalies in northern California, but also consider oceanographic conditions in central California (e.g., at Point Sur and Año Nuevo, Fig. 1) to explore potential mechanisms of poleward transport from Monterey Bay and lower latitudes.

## Results

### Oceanographic conditions

**Figure 2:**
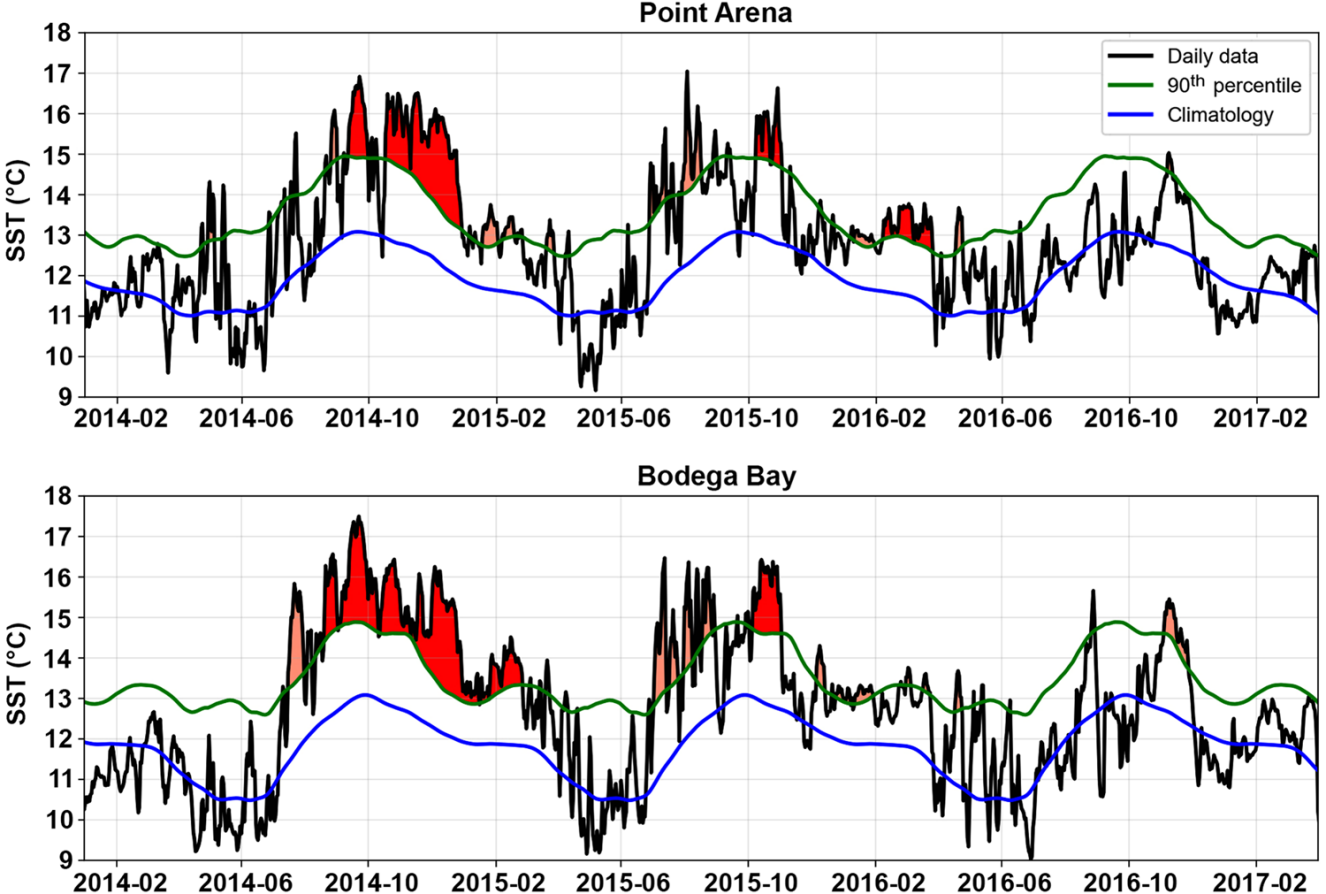
Sea surface temperature (SST) for January 2014 to March 2017 from NOAA/NDBC buoys at Point Arena (upper panel), and Bodega Bay (lower panel). Black line indicates daily SST, blue line indicates the climatology based on the 1981–2011 period, green line indicates the 90^th^ percentile threshold that defines marine heatwaves, and shaded areas indicate events identified as heatwaves^1^. Red shading indicates events that rank among the 10 most intense marine heatwaves based on cumulative intensity of events during 1981–2017.

During 2014–2016, SST in our study region were frequently 2–4 °C above the climatological mean for 1981–2011 (Fig. 2). Using previously defined metrics^1^, our analyses of SST during this period identified 14 heatwaves (see Methods) of varying duration (range = 5–199 days; Fig. 2, Tables S2 and S3) at both Bodega Bay and Point Arena. Based on cumulative intensity, two heatwaves at Bodega Bay ranked among the 10 most intense events since 1981. The most intense lasted 199 days (August 2014 to late February 2015), had a cumulative intensity of 518.3 °C days, and a maximum intensity of 4.4 °C above the climatological mean. The duration and intensity of this heatwave surpassed those that occurred at this location even during strong El Niño events in the past (e.g., 1997–1998, Table S2). At Point Arena, four shorter heatwaves during 2014–2016 ranked among the 10 most intense events since 1981 (Table S3). The most intense occurred during October–December 2014 (74 days), and had a cumulative intensity of 231.8 °C days, and a maximum intensity of 3.9 °C.

**Figure 3:**
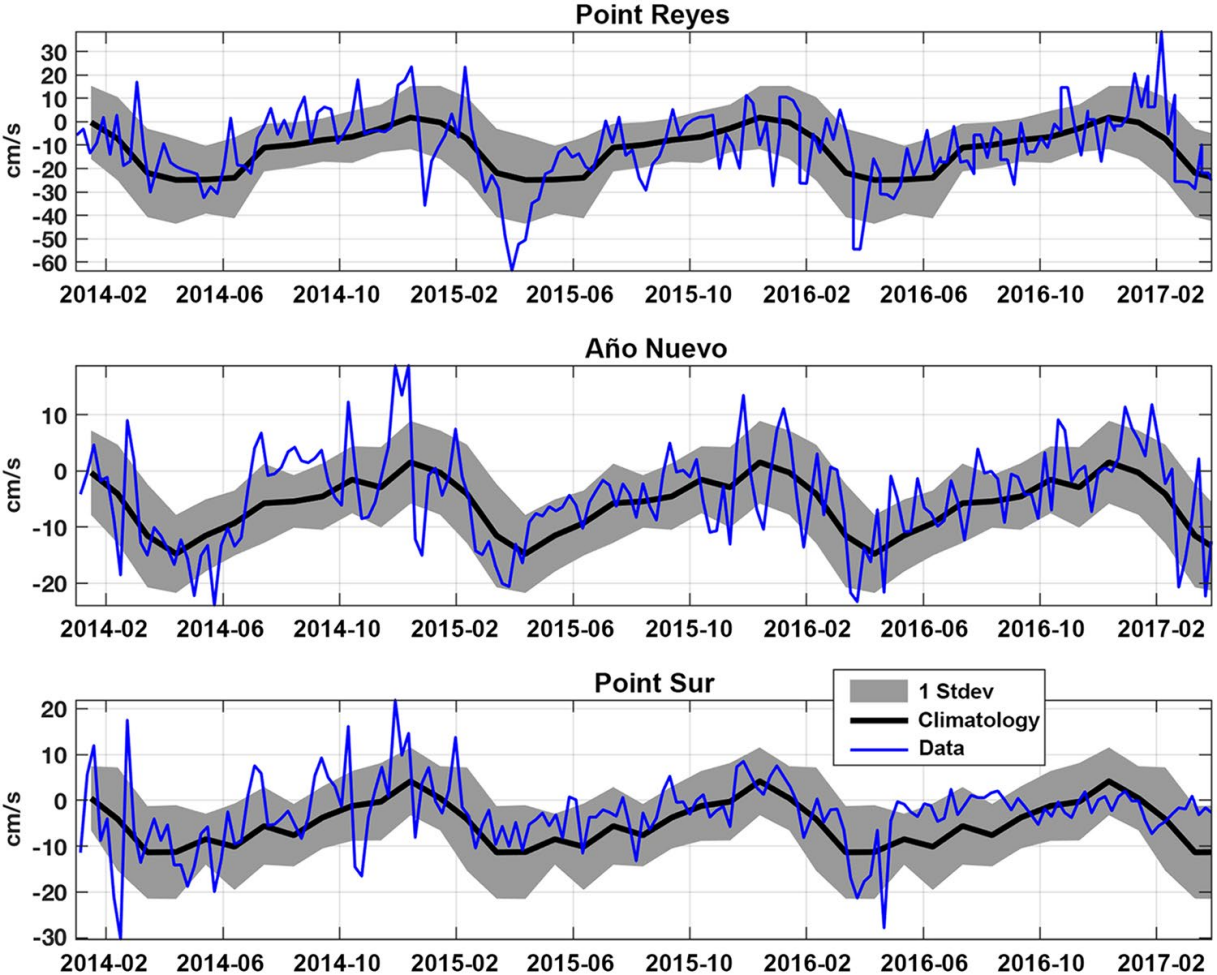
Temporal variation in alongshore flow velocity (North-South) at three locations: northwest of Point Reyes (upper panel), Año Nuevo (middle panel), Point Sur (lower panel). Values are weekly-averaged velocities (cm/s) derived from high-frequency radar. Positive values indicate northward flow, and negative values indicate southward flow. Data compare 2014–2017 (blue line) to mean climatology (2002–2011) and standard deviation (black line and gray shading, respectively).

Anomalous poleward surface currents were common along the coast from Point Sur to Point Reyes during July–November 2014 (Fig. 3). Several shorter periods of anomalous northward flow were also recorded at Año Nuevo and Point Reyes in late 2015/early 2016, during the El Niño event. Although warming dissipated by mid-2016, several short periods of anomalous poleward currents were observed in late 2016 and early 2017.

During 1991–2017, oceanographic conditions in northern California varied substantially among years, as reflected by the Multivariate Ocean Climate Indicator (MOCI; Fig. S68). The period of 2014–2016 was characterized by many (non-continuous) seasons with warm conditions, comparable only to El Niño periods in magnitude (e.g., the 1982–1983 and 1997–1998 events), although more persistent than during prior El Niño events. Warm conditions were broken by quasi-normal upwelling season conditions in northern California during 2014–2016.

### New records of northern geographic range boundaries

During 2014–2017, we recorded poleward range extensions into northern California and higher latitudes for 37 species with primarily southern distributions (Table 1; see also Supplementary Information). These species spanned a diversity of taxa including molluscs (n = 18), crustaceans (6), cnidarians (4), echinoderms (3), ctenophores (1), tunicates (1), fish (1), seabirds (1), marine mammals (1), and red algae (1). In contrast, during this period we observed an equatorward range expansion into our study region of only one northern species (a poorly studied sea cucumber, *Pentamera pediparva*). Of these 37 species, nine belonged to the pelagic community (including gastropods, siphonophores, a jellyfish, a ctenophore, a salp, and pelagic red crabs). Twenty-one were benthic animals with planktonic, feeding larvae. An additional five benthic species had life histories associated with limited dispersal including direct developers, crawling larvae (i.e., the nudibranch *Phidiana hiltoni*^45^), or macroalgal spores. The distance of the range extensions varied significantly among these three groups of taxa (Table 1; ANOVA, F_2,32_ = 17.28, p < 0.0001). Range extensions for pelagic species (mean distance ± SE = 690.0 ± 154.3 km) were 2.6x greater than those of benthic species with planktonic larvae (265.7 ± 33.3 km; Tukey HSD, p = 0.044), and were 9.6x greater than those of benthic species with limited dispersal potential (72.2 ± 48.1 km; Tukey HSD, p < 0.0001). Range extensions for benthic species with planktonic larvae were 3.7x greater than those of benthic species with limited dispersal (Tukey HSD, p = 0.0002).

**Table 1:** Geographic range extensions (n = 37).

Common name	Scientific name	New northern record	Former range limit	Habitat/Life history	Range extension
Red Alga (A)	*Dasya binghamiae*	Bodega Harbor, CA	Estero de San Antonio, CA	Benthic/LD	7
Sunburst Sea Anemone (CN)	*Anthopleura sola*	Bruhel Point, CA	Bodega Marine Reserve, CA	Benthic/PL	165
Siphonophore (CN)	*Hippopodius hippopus*	Salmon Creek Beach, CA	Point Conception, CA	Pelagic	490
Hula Skirt Siphonophore (CN)	*Physophora hydrostatica*	Mad River Beach, CA	Monterey Bay, CA	Pelagic	530
Purple-striped Jellyfish (CN)	*Chrysaora colorata*	Arcadia Beach, OR	Bodega Bay, CA	Pelagic	875
Venus’ Girdle Ctenophore (CT)	*Cestum veneris*	Rainy Bay, BC	Monterey Bay, CA	Pelagic	1400
Angular Unicorn Snail (M)	*Acanthinucella spirata*	Cape Mendocino, CA	Bodega Harbor, CA	Benthic/LD	260
Appleseed Erato Snail (M)	*Hespererato vitellina*	Cape Mendocino, CA	Bodega Marine Reserve, CA	Benthic/PL	255
Purple Sea Snail (M)	*Janthina umbilicata*	Leadbetter Point, WA	Neskowin, OR	Pelagic	140
Scaled Tube Snail (M)	*Thylacodes squamigerus*	Salt Point State Park, CA	Bodega Marine Reserve, CA	Benthic/PL	36
Curved Needle Pteropod (M)	*Creseis virgula*	Bodega Line Station 4	off Central California	Pelagic	150
Striated Sea Butterfly (M)	*Hyalocylis striata*	Bodega Line Station 4	Baja California, Mexico	Pelagic	1400
Strong’s Sidegill (M)	*Berthella strongi*	MacKerricher State Park, CA	Moss Beach, CA	Benthic/PL	255
Black-tipped Spiny Dorid (M)	*Acanthodoris rhodoceras*	Chup Point, BC	Cape Arago, OR	Benthic/PL	620
Olive’s Nudibranch (M)	*Anteaeolidiella oliviae*	Fort Bragg, CA	Duxbury Reef, CA	Benthic/PL	210
Rabbit Dorid Nudibranch (M)	*Crimora coneja*	Boiler Bay, OR	Cape Arago, OR	Benthic/PL	170
Colorful Dirona Nudibranch (M)	*Dirona picta*	Bamfield, BC	Cape Meares, OR	Benthic/PL	385
White-spotted Sea Goddess (M)	*Doriopsilla albopunctata*	Whiskey Creek, OR	Mendocino, CA	Benthic/PL	335
White-spotted Dorid (M)	*Doriopsilla fulva*	Netarts Bay, OR	Abalone Beach, CA	Benthic/PL	485
Janolus Nudibanch (M)	*Janolus barbarensis*	Bodega Harbor, CA	San Francisco Bay, CA	Benthic/PL	87
Los Angeles Okenia (M)	*Okenia angelensis*	Miwok Beach, CA	San Francisco Bay, CA	Benthic/PL	94
Hilton’s Nudibranch (M)	*Phidiana hiltoni*	Pinnacle Gulch, CA	Duxbury Reef, CA	Benthic/LD	63
Orange-spike Polycera (M)	*Polycera atra*	Monas Island, BC	Westport, WA	Benthic/PL	290
Spotted Triopha Nudibranch (M)	*Triopha maculata*	Port Hardy, BC	Bamfield, BC	Benthic/PL	425
Spiny Lobster (CR)	*Panulirus interruptus*	Horseshoe Cove (BMR), CA	San Francisco Bay, CA	Benthic/PL	87
Chocolate Porcelain Crab (CR)	*Petrolisthes manimaculis*	Point St. George, CA	Trinidad, CA	Benthic/PL	80
Xantus’ Swimming Crab (CR)	*Portunus xantusii*	Tomales Bay, CA	Morro Bay, CA	Benthic/PL	390
Pelagic Red Crab (CR)	*Pleuroncodes planipes*	Agate Beach, OR	Fort Bragg, CA	Pelagic	595
Pink-striped Barnacle (CR)	*Megabalanus californicus*	Humbug State Park, OR	Humboldt Bay, CA	Benthic/PL	215
Red-striped Barnacle (CR)	*Paraconcavus pacificus*	MacKerricher State Park, CA	San Francisco, CA	Benthic/PL	240
Glass-spined Brittle Star (E)	*Ophiothrix spiculata*	Patrick’s Point State Park, CA	Cordell Bank, CA	Benthic/PL	360
Scarlet Sea Cucumber (E)	*Lissothuria nutriens*	Fort Ross Reef, CA	Bodega Marine Reserve, CA	Benthic/LD	25
Red Sea Cucumber (E)	*Pachythyone rubra*	Bodega Marine Reserve, CA	Pinnacle Gulch, CA	Benthic/LD	6
Salp (T)	*Thetys vagina*	Calvert Island, BC	Grays Canyon, WA	Pelagic	630
Pacific Snake Eel (F)	*Ophichthus triserialis*	Lincoln City, OR	Klamath River, CA	Benthic/PL	395
Wedge-rumped Storm-Petrel (B)	*Oceanodroma tethys*	Humboldt Bay, CA	Monterey Bay, CA	N/A	500
Common Bottlenose Dolphin (MM)	*Tursiops truncatus*	Little River, CA	Doran Beach, CA	N/A	130

New records of southern-ranging species observed north of Point Reyes, California, during 2014–2017. Taxonomic group indicated in parentheses: A = alga, B = bird, CN = cnidarian, CR = crustacean, CT = ctenophore, E = echinoderm, F = fish, M = mollusc, MM = marine mammal, T = tunicate. Columns 3–4 show the new northern range limit and the former range limit, respectively (see Supplementary Information). Column 5 classifies species based on whether adults are pelagic vs. benthic, and whether benthic species have planktonic, feeding larvae (PL), or limited dispersal (LD) associated with direct development, for example. N/A = not applicable. The final column shows the distance of the range extension (km). Coordinates for all geographic locations are listed in Table S1.

### Unusual records of occurrence

In addition to records of new northern geographic range limits, we observed a large influx of 21 primarily southern species that are rare in northern California (Table 2). Historical records and museum collections suggest that many of these species had been observed in northern California only in association with past El Niño events (Supplementary Information). For example, we are aware of only three prior records of the pelagic snail *Janthina janthina* occurring north of Point Conception, California, and all are associated with El Niño events (Supplementary Information). During 2014–2016, some of these species were recorded at very low densities (e.g., one or a few individuals at a particular site, such as the nudibranch *Hancockia californica*). Others were locally abundant and were observed at many sites throughout northern California (e.g., the nudibranch *Okenia rosacea*^45^).

**Table 2:** Rare occurrences (n = 21).

Common name	Scientific name	Local site	Known range limit
Siphonophore (CN)	*Diphyes dispar*	Salmon Creek Beach, CA	off Brookings, OR
White Flatworm (PL)	*Pseudoceros luteus*	Bodega Harbor, CA	Bodega Harbor, CA
Violet Sea Snail (M)	*Janthina janthina*	Salmon Creek Beach, CA	Neskowin, OR
Spanish Shawl Nudibranch (M)	*Flabellinopsis iodinea*	Pinnacle Gulch, CA	Vancouver Island, BC
Hancock’s Nudibranch (M)	*Hancockia californica*	Pinnacle Gulch, CA	Trinidad, CA
Hopkins’ Rose Nudibranch (M)	*Okenia rosacea*	Bodega Head, CA	Gregory Point, OR
Hedgpeth’s Nudibranch (M)	*Polycera hedgpethi*	Bodega Harbor, CA	Bodega Harbor, CA
California Sea Hare (M)	*Aplysia californica*	Miwok Beach, CA	Yaquina Bay, OR
California Aglaja (M)	*Navanax inermis*	Bodega Harbor, CA	Bodega Harbor, CA
Blue Buoy Barnacle (CR)	*Dosima fascicularis*	Salmon Creek Beach, CA	Salisbury Sound, AK
Salp (T)	*Thalia democratica*	Salmon Creek Beach, CA	Newport, OR
Ocean Whitefish (F)	*Caulolatilus princeps*	Farallon Islands, CA	Vancouver Island, BC
Black Storm-Petrel (B)	*Oceanodroma melania*	Cordell Bank, CA	Seaside, OR
Black-vented Shearwater (B)	*Puffinus opisthomelas*	off Bodega Marine Reserve, CA	Vancouver Island, BC
Guadalupe Murrelet (B)	*Synthliboramphus hypoleucus*	Cordell Bank, CA	Washington
Brown Booby (B)	*Sula leucogaster*	Tomales Bay, CA	Alaska
Green Sea Turtle (R)	*Chelonia mydas*	Golden Gate, CA	Alaska
Olive Ridley Sea Turtle (R)	*Lepidochelys olivacea*	Salmon Creek Beach, CA	Alaska
Guadalupe Fur Seal (MM)	*Arctocephalus townsendi*	Doran Beach, CA	Alaska
Long-beaked Common Dolphin (MM)	*Delphinus capensis*	Cordell Bank, CA	British Columbia
Short-beaked Common Dolphin (MM)	*Delphinus delphis*	Cordell Bank, CA	British Columbia

Southern-ranging species not typically found in northern California, but with rare historical records in this and other northern regions (often in association with El Niño events, see Supplementary Information). Taxonomic group indicated in parentheses: B = bird, CN = cnidarian, CR = crustacean, F = fish, M = mollusc, MM = marine mammal, PL = platyhelminth, R = reptile, T = tunicate. Columns 3 and 4 show where the species was observed locally during the study, and the known northern geographic range limit, respectively. Coordinates for all geographic locations are listed in Table S1.

### Increases in the abundance and larval recruitment of southern species

An additional set of primarily southern species also responded strongly during the marine heatwaves (Table 3). These species had been present routinely at our field sites in very low abundance during 2004–2013 (see Supplementary Information), but each experienced a pronounced increase in local abundance during 2014–2017. For two of these species with planktonic larvae (the owl limpet *Lottia gigantea* and volcano barnacle *Tetraclita rubsecens*), we conducted targeted field surveys to quantify changes in recruitment and abundance through time.

**Table 3:** Southern-ranging species that experienced a pronounced increase in local abundance during 2014–2016 (n = 14).

Common name	Scientific name	Local site(s)	Known range limit
By-the-wind Sailor (CN)	*Velella velella*	Salmon Creek Beach, CA	Alaska
Sunburst Sea Anemone (CN)	*Anthopleura sola*	Bodega Marine Reserve, CA	Bruhel Point, CA*
Owl Limpet (M)	*Lottia gigantea*	Bodega Marine Reserve, CA	Crescent City, CA
Monterey Tube Snail (M)	*Petaloconchus montereyensis*	Bodega Marine Reserve, CA	Van Damme State Park, CA**
White-spotted Dorid (M)	*Doriopsilla fulva*	Salt Point State Park, CA	Netarts Bay, OR*
Bryozoan (BR)	*Jellyella tuberculata*	Bodega Marine Reserve, CA	Bodega Marine Reserve, CA
Pink-striped Barnacle (CR)	*Megabalanus californicus*	Bodega Marine Reserve, CA	Humbug State Park, OR*
Pink Volcano Barnacle (CR)	*Tetraclita rubescens*	Salt Point & Van Damme, CA	Burnt Hill, OR
Spiny Mole Crab (CR)	*Blepharipoda occidentalis*	Salmon Creek Beach, CA	Salmon Creek Beach, CA**
Mole Crab (CR)	*Emerita analoga*	Salmon Creek Beach, CA	Alaska
Chocolate Porcelain Crab (CR)	*Petrolisthes manimaculis*	Pinnacle Gulch, CA	Point St. George, CA*
Scarlet Sea Cucumber (E)	*Lissothuria nutriens*	Bodega Marine Reserve, CA	Fort Ross Reef, CA*
Pyrosome (T)	*Pyrosoma atlanticum*	Salmon Creek Beach, CA	Sitka, AK
Ocean Sunfish (F)	*Mola mola*	Bodega Bay & Cordell Bank, CA	Alaska

Taxonomic group indicated in parentheses: BR = bryozoan, CN = cnidarian, CR = crustacean, E = echinoderm, F = fish, M = mollusc, T = tunicate. Column 3 shows the local site where the increase was observed. Column 4 shows the known northern geographic range limit. One asterisk (*) indicates a new northern range limit recorded during this study (Table 1), whereas two asterisks (**) indicates an unpublished northern range record that we observed prior to 2014 (see Supplementary Information). Coordinates for all geographic locations are listed in Table S1.

#### Owl limpets

**Figure 4:**
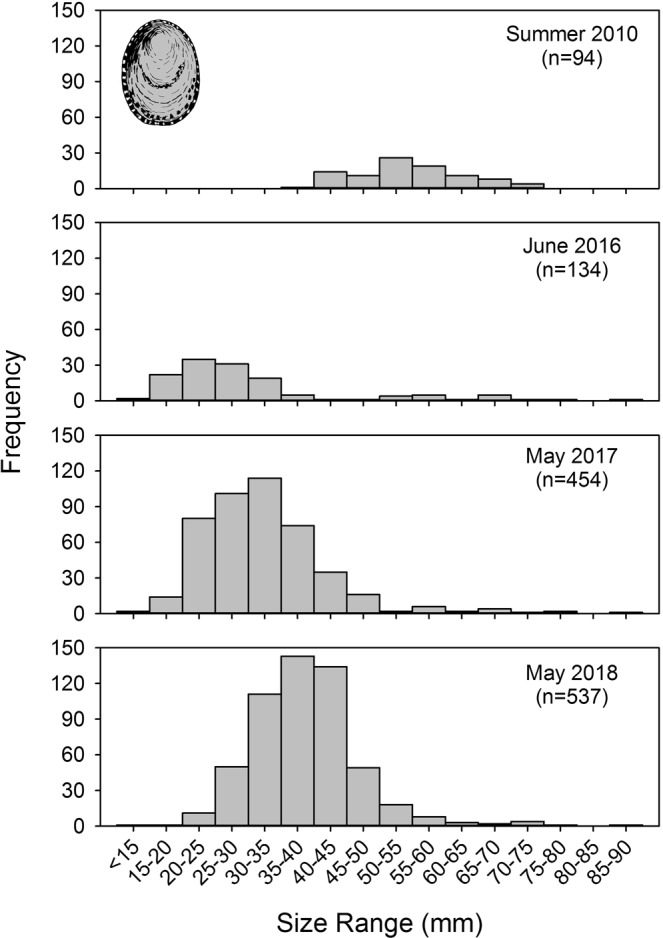
Owl limpet (*Lottia gigantea*) size frequency distributions at Bodega Marine Reserve, California. During 2006–2013, the population was characterized by little to no recruitment and almost all individuals were in larger size classes. Two cohorts of juvenile owl limpets (15–40 mm long) recruited during late 2014/early 2015 and late 2015/early 2016 in association with heatwave events, with subsequent growth into larger size classes in 2017 and 2018. n = sample size.

Only larger size classes of *Lottia gigantea* were present at Bodega Marine Reserve in 2010 with no evidence of recent recruitment of smaller individuals (Fig. 4). During 2011–2014, we noted the gradual loss of these larger owl limpets, but never recorded the presence of small owl limpets (J.L.S. and E.S., *personal observations*). In June 2016, we recorded a striking increase in recruitment of owl limpets (Fig. 4). The majority of these individuals were 15–35 mm long, and based on published age-size relationships for this species^46^, likely settled during late 2014 in association with anomalously warm conditions and poleward currents. By May 2017, many of these owl limpets had grown into the larger size classes (30–50 mm) expected for ~2.5-year old owl limpets^46^. Also in May 2017, a second large cohort of small (15–35 mm) owl limpets was apparent, and these likely represented ~1.5-year old limpets that settled in late 2015 in association with the El Niño event. By May 2018, limpets from the two years of successful recruitment (late 2014, late 2015) continued to grow into larger size classes (30–55 mm). Small numbers of new recruits (15–35 mm) were also recorded, suggesting some recruitment in late 2016 after warm-water conditions had dissipated (Fig. 4). Survival of *L*. *gigantea* recruits was high; 90.6% of juvenile limpets survived between the 2016 and 2017 annual surveys, and 82.4% of juvenile limpets survived between the 2017 and 2018 annual surveys.

#### Volcano barnacles

**Figure 5:**
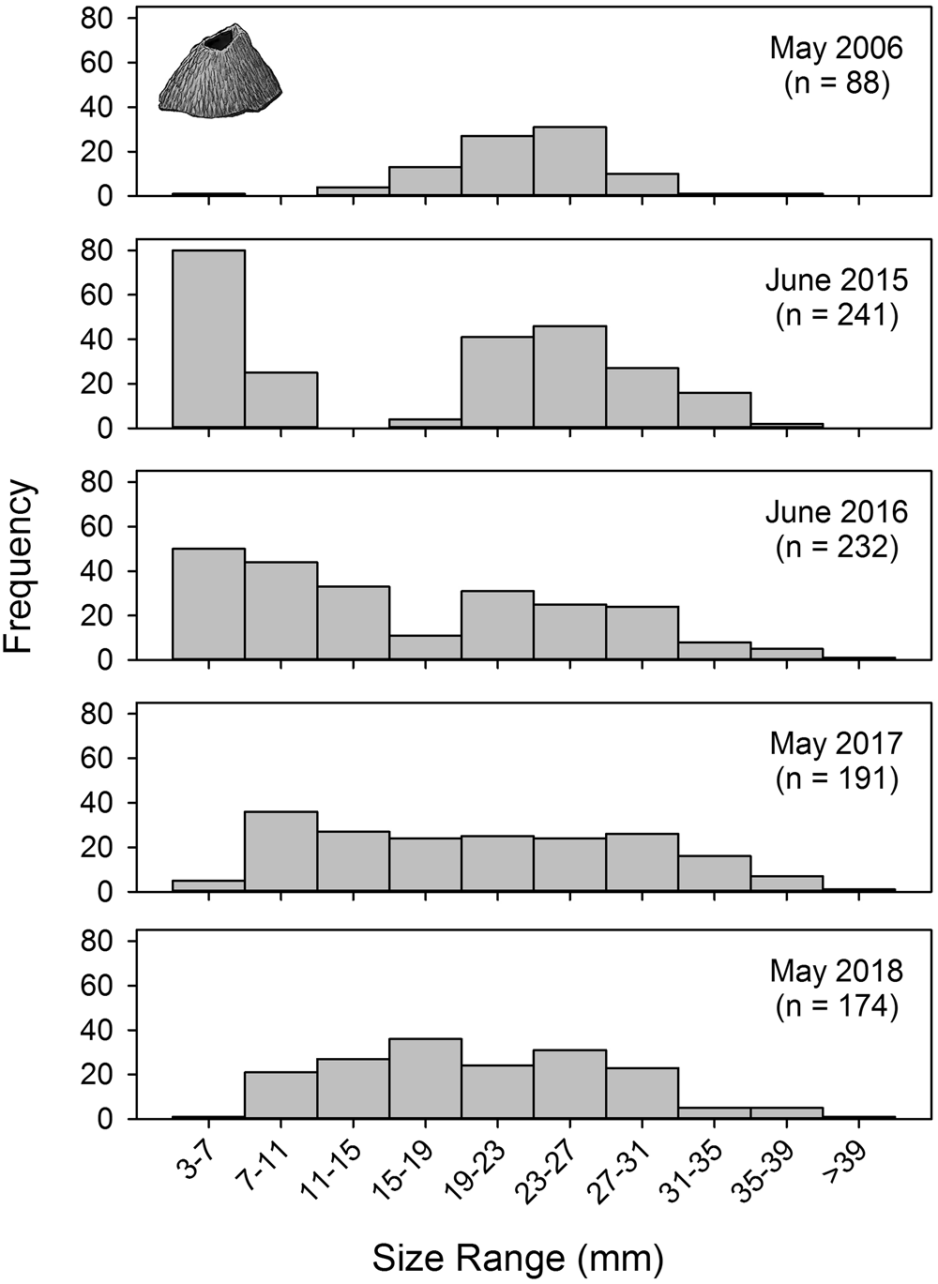
Volcano barnacle (*Tetraclita rubescens*) size frequency distributions at Van Damme State Park, California. During 2006–2013, the population was characterized by infrequent recruitment and almost all individuals were in larger size classes. Discrete recruitment events (barnacles < 11 mm diameter) occurred during late Summer/Fall 2014 and late Summer/Fall 2015, with minimal recruitment during late Summer/Fall or 2016 and 2017. n = sample size.

In May 2006, the population of *Tetraclita rubescens* at Van Damme State Park was composed almost entirely of larger barnacles (Fig. 5). Observations at this site during 2006–2014 (at least one visit per year) indicated a similar distribution of sizes, with very low recruitment of *Tetraclita* during this period (E.S. and J.L.S., *personal observations*). In June 2015, we recorded a large recruitment pulse of small *Tetraclita* (basal diameter ≤11 mm). Based on published age-size relationships for this species^47^, these individuals recruited during Fall 2014 in association with the anomalously warm conditions and poleward currents. By June 2016, many of these barnacles had grown into larger size classes (11–20 mm), and there was evidence of a second, smaller recruitment event from Fall 2015 associated with the El Niño event. In May 2017 and May 2018, a continued shift of recruits from the warm-water events into larger size classes was apparent, but these surveys suggested minimal recruitment of new individuals (basal diameter <11 mm) during Fall 2016 and Fall 2017, respectively.

## Discussion

### Oceanographic changes and the distribution and abundance of southern taxa

Although shifts in geographic distribution have frequently been associated with strong El Niño events^21,22,26,48^ and other marine heatwaves^49^, the number of poleward range extensions observed during this study appears unprecedented. Conditions in northern California during 2014–2016 were characterized by SST that were often 2–4 °C above the climatological mean, and included a series of more than a dozen heatwaves (i.e., events lasting ≥5 days with SSTs warmer than the 90^th^ percentile based on the 30-year climatology^1^). The most intense heatwave at Bodega Bay lasted 199 days (August 2014 to late February 2015), with a cumulative intensity (518.3 °C days) that exceeded that of other extreme heatwaves recorded in Western Australia (2011), the NW Atlantic (2012), the Mediterranean Sea (2014), and the Tasman Sea (2015–2016)^1,50^. Given that cooler SST can limit the development and successful recruitment of larval stages of biota from lower latitudes^43,51^, the unusually long duration of anomalously warm temperatures in northern California during 2014–2016 was likely a primary driver of poleward range shifts observed in our study.

In addition, anomalous poleward transport might have contributed to shifts in the distribution of southern biota during 2014–2016, a hypothesis proposed to explain geographic range expansions during some previous El Niño events^21,23,52,53^. Larval dispersal is influenced by a combination of advective and diffusive processes^54^, both of which may be important in explaining poleward transport in our study. Alongshore currents during 2014–2016 exhibited anomalous poleward flows, especially during the second half of 2014. Although such anomalies were infrequent overall, the persistent 15 cm/s poleward flow past Point Año Nuevo during November 2014 could have advected plankton over distances of ~500 km in one month. Moreover, it is possible that some plankton were transported north through a sequence of poleward flows rather than a single advection event. During late 2014, alongshore flow often did not revert to equatorward flow between poleward flow anomalies. This pulsed advection, such as the stop-start poleward flow past Point Año Nuevo during July–August 2014, can account for ~300 km displacement. Lastly, poleward transport might arise via a diffusion mechanism in which some of the plankton transported north during a brief event are not returned south as flows reverse, but are retained and transported farther north during the next brief poleward event. This process might ultimately result in a portion of the larval pool moving considerable distances north through a series of poleward flow events, such as flows past Point Reyes during July–October 2014 (potential displacement of ~400 km). This last diffusive transport scenario can be enhanced by retention features like bays (e.g., Monterey Bay, or the Gulf of Farallones) or offshore mesoscale eddies and requires that some flows be directed poleward, especially from March to August when mean flow off central and northern California is typically equatorward. Thus, even marginally anomalous flows, as observed during 2014–2016, might result in a marked increase in the probability of poleward transport of propagules.

There are other possible explanations for anomalous transport of warm-water biota into our region during 2014–2016. In particular, enhanced onshore transport might have delivered organisms to coastal waters that are typically found in warmer, offshore waters, including many of the pelagic species reported in this study. Although there was no evidence of anomalous downwelling along the California coast during 2014–2016^15^, previous studies suggest additional mechanisms of onshore transport during El Niño years^55^.

It is likely that both temperature and transport played a role in the poleward shift of biota, and that the combination of these effects would be more effective than either one alone^41,42,56^. For example, warmer temperatures shorten planktonic larval durations^57^ and thus may increase the chances that southern species will be transported poleward, complete development, and settle in benthic habitats within the timespan of temporary current reversals^51,58^. Indeed, the combined influence of changes in temperature and currents has facilitated range expansions in other geographic regions^8,9,41–43,56,59^.

While we recorded range extensions and/or increased recruitment of many southern species, others species with northern range boundaries at Monterey Bay did not undergo range expansions into our study region. Individualistic responses of this kind have been observed in other studies of geographic range shifts and likely depend in part on the specific life histories, dispersal potentials, and habitat requirements of a given species^39,42,60,61^. For example, many of the nudibranch species in our study reproduce over a large proportion of the year, including production of larvae during fall and winter (Table S4). Similarly, the larvae of owl limpets and volcano barnacles both occur in the plankton primarily during the second half of the year (September–January, and July–November, respectively^62,63^). The timing of larval dispersal in these species thus overlapped with periods of strong surface poleward flow and warm SST during 2014–2016 (Figs 2 and 3). In contrast, planktonic larvae of species that reproduce during the spring likely encountered primarily equatorward flow and more typical, cooler SST during 2014–2016.

The magnitude of geographic range extensions was greatest for taxa that are members of the pelagic community as adults (e.g., jellyfish, ctenophores, pteropods, and pelagic red crabs). The prolonged occurrence of these species in the pelagic environment presumably increases the chances that some individuals will be transported poleward by advective and diffusive processes. For benthic taxa that experienced range extensions, species with planktonic, feeding larvae exhibited greater range extensions than those species with direct development and limited dispersal potential, such as brooding sea cucumbers. Although some meta-analyses have found faster range extensions in pelagic organisms and highly mobile fish than in benthic species^64^, others have failed to find a significant effect of reproductive mode and dispersal potential on the rate of range shifts^65,66^. When evaluated over the same timescale as anomalous conditions during 2014–2016, dispersal potential likely had a direct influence on the colonization of poleward regions. In contrast, when range shifts are assessed over decadal timescales^65,66^, initial patterns of dispersal and colonization during heatwaves may sometimes be reshaped by additional abiotic and biotic factors.

### Persistence of ecological changes

Ecological changes associated with marine heatwaves can lead to population changes that range from short-lived to persistent^9,67^. Poleward appearances of southern biota beyond their typical range boundaries are often ephemeral, with species disappearing from the region when the oceanographic event ends, due to unsuitable conditions in the northern habitat and/or the short lifespan of some species^53^. This was the case, for example, for many of the primarily southern nudibranchs that occurred only ephemerally in our study region during the marine heatwaves (e.g., *Flabellinopsis iodinea*, *Anteaeolidiella oliviae*, *Janolus barbarensis*, and others^20^). In other cases, larval recruitment may establish “relict populations” that decline in abundance more slowly before eventually disappearing^67^. This may be the case for the Hopkins’ Rose Nudibranch (*Okenia rosacea*), which was still present in northern California during summer 2018, over two years after the El Niño ended, but at much lower densities than occurred in the region during 2015–2016 (see Supplementary Information). A final possible outcome of marine heatwaves is that warmer water and increased larval transport from lower latitudes may establish or sustain “marginal” or sink populations that can persist indefinitely^67^. Such sink populations are maintained primarily by episodic larval recruitment from southern source populations^68^, as appears to be the case for owl limpets and volcano barnacles in our study region^63,69^.

Whether or not northern populations established during marine heatwaves persist will likely be influenced by several other factors beyond future larval recruitment and connectivity with southern source populations. First, the physiological tolerances of a species will determine if new recruits can persist in poleward habitats when more typical temperatures return^42,51,56,59^. Second, the lifespan of a species will also influence the persistence of marginal populations. For example, volcano barnacles and owl limpets are relatively long-lived species, and individuals can live 10–15 years^62^ or >20 years^69^, respectively. Moreover, our results suggest that survival of juvenile owl limpets in northern California is relatively high. Thus, even infrequent recruitment associated with El Niño events may be sufficient to maintain marginal populations of some southern species^68,69^. Finally, mode of reproduction and habitat are likely important in determining whether marginal populations will contribute to future recruitment in the northern region. For example, for broadcast spawners, low population density may reduce fertilization success via Allee effects^39^. Open coast populations that produce planktonic larvae near the northern edge of a geographic range might contribute minimally to future recruitment in this region if prevailing coastal currents carry most larvae equatorward^58^. In contrast, species with direct development (e.g., brooders) might be self-sustaining once a sufficiently dense population is established in a northern location. During our study, two species with direct development (*Lissothuria nutriens*, *Petaloconchus montereyensis*) underwent striking increases in local abundance in northern populations (see Supplementary Information). The capacity for self-recruitment may allow southern species with direct development to respond rapidly to favorable, warm-water conditions.

### Range expansions in a temperate transition zone

**Figure 6:**
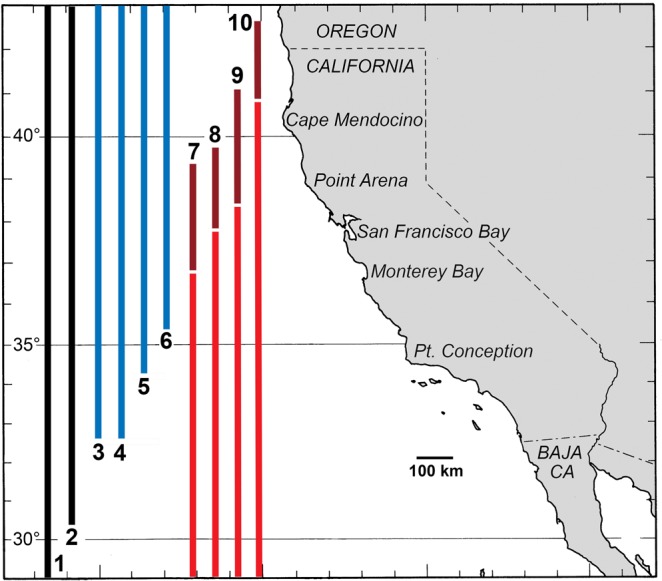
Geographic ranges of common intertidal/shallow subtidal barnacles of California (after Newman^34^). This assemblage includes species that are cosmopolitan (black bars), primarily northern (blue bars), and primarily southern (red bars). The highest species richness of intertidal barnacles occurs in central/north central California between Point Conception and Point Arena. For southern species, red bars indicate northern range limits during the 1970s^34^, and dark red bars indicate geographic range expansions to current poleward boundaries (see Supplementary Information). Species are coded as follows: (1) *Pollicipes polymerus*, (2) *Balanus glandula*, (3) *Chthamalus dalli*, (4) *Balanus nubilus*, (5) *Balanus crenatus*, (6) *Semibalanus cariosus*, (7) *Paraconcavus pacificus*, (8) *Chthamalus fissus*, (9) *Tetraclita rubescens*, (10) *Megabalanus californicus*. Note that southern range limits are those published by Newman^34^, as we are unaware of data that address whether these equatorward boundaries have retracted.

Temperate transition zones are often hotspots of diversity because these regions contain a mix of both warm-adapted and cool-adapted species^37,39^. The coast of California from Monterey Bay to Point Arena represents such a transition zone with high species richness that arises in part from benthic communities that include a mix of species with differing biogeographic affinities^5^. For example, of the 10 common intertidal barnacles in California, two are cosmopolitan species found coastwide, four are primarily northern, and four are primarily southern^34^. All 10 species occur in the region from Monterey Bay to Point Arena, and the boundary of this transition zone has shifted poleward since the 1970s (Fig. 6). Although El Niño events are typically viewed as ecological disturbances, not all associated effects on marine ecosystems are negative^70^. Indeed, episodic periods of warmer water and enhanced northward transport during warm-water events may be critical to the recruitment and population persistence of some primarily southern species in northern California. This may be particularly true for relatively long-lived species where northern populations can persist through extended periods of low recruitment. These recruitment dynamics may play an underappreciated role in maintaining the high species richness of transition zones. For example, during 2014–2016, all four southern barnacles in our study region increased in abundance and/or experienced geographic range extensions.

Episodic recruitment of southern species during El Niño events may also play a key role in facilitating geographic range expansions against a backdrop of ongoing climate change. In recent decades, the species composition of intertidal communities in the Bodega Bay region has shifted to include more southern fauna of the Montereyan biogeographic province, including the sea anemone *Anthopleura sola*, the barnacles *Tetraclita rubescens* and *Megabalanus californicus*, the porcelain crab *Petrolisthes manimaculis*, the owl limpet *Lottia gigantea*, and the vermetid tube snail *Thylacodes squamigerus*. These six species were historically rare or absent in the region during the 1970s (see Supplementary Information), but all have become more common in recent decades, including marked increases in abundance during 2014–2016. In addition, the geographic ranges of four of the six species expanded farther north during 2014–2016. Our results are thus consistent with a gradual shift in the poleward boundary of the Montereyan biogeographic province^5^ analogous to trends towards tropicalization seen in other regions of the world^8,37,39^.

Growing evidence suggests that gradual, long-term shifts in geographic distributions can be punctuated by rapid expansions where marginal populations are established during extreme events^6^. Meta-analyses suggest that marine range extensions have occurred at a mean rate (±SE) of 72.0 km (±13.5) per decade^64^. In contrast, the mean (±SE) range extension recorded in our study was 345.4 km (±53.9 ) occurring within a period of a few years or less. Thus, marine heatwaves provide a mechanism for the rapid poleward expansion of some species into new regions. While many of these northern populations might not persist, the establishment of such marginal populations may be essential if poleward range expansions proceed in a stepping-stone fashion^71^. Marginal populations established during extreme events might be more likely to persist if background levels of ocean warming make northern habitats more favorable for southern species during intervening periods between these events^59^. In addition, more frequent and longer heatwaves might also increase the persistence of marginal populations and accelerate shifts in species ranges^37^. For example, the prolonged duration of warm-water conditions and two years of successful larval recruitment might have allowed populations of volcano barnacles, owl limpets, and other southern species to reach a threshold density for effective reproduction (e.g., a minimum density required for fertilization success in broadcast spawning limpets or copulating barnacles). Our surveys of owl limpets in May 2018 indicate low levels of larval recruitment occurred in northern California in late 2016 after warm-water conditions had dissipated. This suggests the possibility that owl limpet populations in our study region have become sufficiently dense that they are starting to contribute larvae that may help sustain northern populations and perhaps facilitate a poleward shift in geographic distribution.

Whereas relatively short, high-intensity marine heatwaves can be sufficient to trigger mass mortality events^1,7^, we suggest that greatly prolonged heatwaves, like those that occurred along the California coast during 2014–2016, are especially likely to facilitate poleward dispersal and range extensions. Understanding the oceanographic, ecological, and evolutionary processes that mediate the success of such range expansions will be critical to predicting future shifts in the community composition of biogeographic transition zones in an era of accelerating climatic change.

## Methods

### Oceanographic Conditions

We considered two oceanographic parameters that were likely to influence poleward transport of southern fauna: sea surface temperatures (SST) and surface currents. We also reviewed wind data, which did not exhibit marked anomalies, seldom exceeding one standard deviation owing to high variability in winds.

Daily SST data for the period were obtained from NOAA/NDBC Buoys 46013 (off Bodega Bay) and 46014 (off Point Arena) located over the continental shelf (Fig. 1). Gaps in these data were filled with linear regressions with data from the Optimal Interpolation SST database V2^72^. Climatological means and variances (SD) were calculated for the period 1981–2011, and daily anomalies as well as 90^th^ percentiles were calculated from the climatological mean. Marine heatwaves during January 2014 to March 2017 were identified using previously published metrics^1^, and were defined as any event that lasted five days or longer with SST warmer than the 90^th^ percentile based on the 30-year climatology; short periods of one or two days below the 90^th^ percentile do not end a marine heatwave event. We used the Python scripts provided by E. C. J. Oliver (https://github.com/ecjoliver/marineHeatWaves) to identify these events (start and end dates) and to calculate the duration and intensity (maximum, mean, and cumulative) for each event.

Daily North-South surface flow was calculated from High Frequency (HF) Radar at three California coastal areas, where values were averaged within prescribed geographic units defined as: Bodega Bay, 38.0–38.2°N, 123.33–123.00°W; Año Nuevo, 36.9–37.4°N, 122.9–122.4°W; and Point Sur, 36.1–36.6°N, 122.4–121.9°W. Anomalies were calculated based on the climatological means and variances (SD) for the period 2002–2011. Detailed methods for the processing of HF Radar are provided elsewhere^73^.

We used the Multivariate Ocean Climate Indicator (MOCI)^74^ to compare oceanographic conditions during 2014–2016 to past patterns of interannual variability in the northern California region. MOCI has seasonal resolution and synthesizes local and regional data and indices (including the upwelling index and data on sea surface and air temperature, winds, sea level, atmospheric pressure at sea level), with climate indices including El Niño Southern Oscillation (ENSO), Pacific Decadal Oscillation, and the North Pacific Gyre Oscillation. Data included in MOCI and the methodology of calculation are described elsewhere^74^.

### Field Surveys and Biological Observations

Our biological surveys were conducted primarily in the region between Point Reyes and Point Arena (Fig. 1). We include some survey data and records of geographic range extensions from regions farther north, ranging from northern California to British Columbia, Canada. Unusual biological records from south of Point Reyes (e.g., tropical and subtropical animals recorded for the first time in Baja California, southern California, or central California^19,20^) are beyond the scope of this study.

#### Sandy beach surveys

During this study period, E.S. and J.L.S. routinely searched for animals washed ashore along the southern end of Salmon Creek Beach and at Horseshoe Cove on the Bodega Marine Reserve. Surveys consisted of inspecting the beach wrack zone along 1.0 km at Salmon Creek Beach, and/or 0.25 km at Horseshoe Cove Beach, on approximately 229 dates between June 2014 and March 2017. Animals that were uncommon or unfamiliar to us (based on our 12 years of experience at these locations) were recorded and collected for further identification in the laboratory.

#### Rocky intertidal surveys

Between June 2014 and March 2017, E.S. and J.L.S. inspected the intertidal zones of rocky shores in the study region on approximately 118 dates. These visits were associated with monitoring efforts, other research projects, and field courses. Field sites included Bodega Marine Reserve, Van Damme State Park, and other rocky intertidal locations in northern California (Supplementary Information). During these visits, we recorded the presence of any uncommon or unfamiliar marine invertebrates (based on our 12 years of experience working at these locations), and collected specimens for further identification in the laboratory. When possible, voucher specimens of invertebrates were deposited in the collections at the California Academy of Sciences in San Francisco, California (Supplementary Information). For two southern species (owl limpets and volcano barnacles), we conducted field surveys to quantify changes in recruitment and abundance through time, as described in the following two sections.

#### Owl limpet surveys

In spring/summer of 2010, 2016, 2017, and 2018, we conducted targeted searches for owl limpets (*Lottia gigantea*) along 1.0 km of rocky intertidal shoreline within the Bodega Marine Reserve. *Lottia gigantea* are large grazing gastropods that maintain permanent territories on vertical and sloping surfaces in the mid and high intertidal zones^69^. The territories of owl limpets are easily recognized during visual surveys because these patches of rock remain free of sessile animals and support the growth of conspicuous diatom films. We measured the shell length of each owl limpet that was discovered and mapped and recorded its location within the same intertidal areas during each survey.

#### Volcano barnacle surveys

We quantified the size frequency distributions of volcano barnacles (*Tetraclita rubescens*) in the mid intertidal zone at Van Damme State Park in May/June 2006, 2015, 2016, 2017, and 2018. We placed five mid intertidal transects (~10 m long) parallel to the shoreline and randomly sampled 0.25 m^2^ quadrats placed along each transect (n = 30 quadrats total). Within each quadrat, we counted the number of live *Tetraclita* present and measured the basal diameter of each individual. In May 2006, we did not quantify density but instead measured the basal diameters of the first 88 individuals encountered.

#### Other records of unusual occurrences

In addition to our own field surveys, we collected records from several other sources. These included specimens that we identified in plankton tows that were conducted by colleagues within Bodega Marine Reserve and over Bodega Canyon (approximately 45 km offshore), primarily during Fall 2014. We verified and compiled additional observations made by other researchers at Bodega Marine Laboratory and observations reported via other resources^20^, including records from the online database iNaturalist (http://www.inaturalist.org/).

#### Analyses of ecological responses to warm-water events

We classified these observations into three categories of ecological responses^19,67^:

1. *Geographic range extension*: a new record of a species occurring beyond its previously recorded northern range limit.
2. *Unusual northern occurrence*: an observation of a southern species not normally found in our study region, but with some prior records (often in association with past El Niño events).
3. *Increase in abundance*: a primarily southern species that had been observed in the region during most years, but that underwent a large increase in abundance.

For each species in this study, we determined its previously documented northern range limit using a variety of resources, including published journal articles, reference guides, and taxonomic keys^62,75^. Published range records are often incomplete, so we also searched online museum databases and visited the California Academy of Sciences to search for uncatalogued northern records of these species. Although historical data are invaluable for the study of range shifts, we acknowledge that some species in our study that lacked prior records from poleward regions might in fact represent cases of non-detection arising from insufficient or unequal sampling^76^. Using the best estimates of prior range boundaries (Supplementary Information), we then calculated the distance along the coastline for each new geographic range extension (km) using Google Earth. These species were separated into three categories based on habitat and life history: (a) members of the pelagic community, (b) benthic species with planktonic, feeding larvae, or (c) benthic species with limited dispersal potential (i.e., direct development, lecithotrophic/non-feeding larvae, or algal spores). Two vertebrates (*Oceanodroma tethys*, *Tursiops truncatus*; Table 1) did not fit into these life history categories and were excluded from this analysis. We used an Analysis of Variance (ANOVA) to test whether the distance of range extensions varied among these three groups of taxa. Distances were log-transformed prior to analysis to meet model assumptions regarding homogeneity of variances. Analyses were conducted in JMP Pro v.12.0.1.

## Supplementary information

Supplementary Information

## Data Availability

Sea surface temperature (SST) data analyzed during this study are available from the U.S. National Data Buoy Center repository [http://www.ndbc.noaa.gov], and the Physical Oceanography Distributed Active Archive Center [https://podaac.jpl.nasa.gov/dataset/AVHRR_OI-NCEI-L4-GLOB-v2.0]. Surface flow data analyzed during this study are available from the Bodega Ocean Observing Node repository [http://boon.ucdavis.edu/flow_pointreyes.html] and [http://boon.ucdavis.edu/data/flow/boxes/]. Multivariate Ocean Climate Indicator (MOCI) data analyzed in this study are available from the Farallon Institute repository [http://www.faralloninstitute.org/moci]. All data regarding species’ distributions and occurrences (Tables 1–3) generated and analyzed during this study are included in this article and its Supplementary Information file. All remaining datasets generated and/or analyzed during the current study are available from the corresponding author on request.
