# Supplementary material for: Widespread shifts in the coastal biota of northern California during the 2014–2016 marine heatwaves
Source: Sci Rep. 2019 Mar 12;9:4216. doi: 10.1038/s41598-019-40784-3 (PMC6414504; doi:10.1038/s41598-019-40784-3)
Supplement: Supplementary file 1 — Supplementary Information [file 41598_2019_40784_MOESM1_ESM.pdf]

SUPPLEMENTARY INFORMATION

Widespread shifts in the coastal biota of northern California during the 2014–2016 marine heatwaves

Eric Sanford[1,2*], Jacqueline L. Sones[3], Marisol García-Reyes[4], Jeffrey H. R. Goddard[5], and John L. Largier[1,6]

[1]Bodega Marine Laboratory, University of California, Davis, Bodega Bay, California 94923 USA
[2]Department of Evolution and Ecology, University of California, Davis, California 95616 USA
[3]Bodega Marine Reserve, University of California, Davis, Bodega Bay, California 94923 USA
[4]Farallon Institute, 101 H Street, Suite Q, Petaluma, California 94952 USA
[5]Marine Science Institute, University of California, Santa Barbara, California 93106 USA
[6]Department of Environmental Science and Policy, University of California, Davis, California 95616 USA

[*]Correspondence and requests for materials should be addressed to E.S.
Email: edsanford@ucdavis.edu

SUPPLEMENTARY INFORMATION: TABLE OF CONTENTS

A. SPECIES ACCOUNTS: Summaries of changes in geographic distribution and/or abundance during 2014–2017, listed alphabetically by species name:

1. *Acanthinucella spirata* (Angular Unicorn Snail) .... p. 4
2. *Acanthodoris rhodoceras* (Black-tipped Spiny Dorid Nudibranch) .... p. 5
3. *Anteaeolidiella oliviae* (Olive's Nudibranch) .... p. 5
4. *Anthopleura sola* (Sunburst Sea Anemone) .... p. 5
5. *Aplysia californica* (California Sea Hare) .... p. 6
6. *Arctocephalus townsendi* (Guadalupe Fur Seal) .... p. 6
7. *Berthella strongi* (Strong's Sidegill) .... p. 7
8. *Blepharipoda occidentalis* (Spiny Mole Crab) .... p. 7
9. *Caulolatilus princeps* (Ocean Whitefish) .... p. 7
10. *Cestum veneris* (Venus' Girdle Ctenophore) .... p. 7
11. *Chelonia mydas* (Green Sea Turtle) .... p. 8
12. *Chrysaora colorata* (Purple-striped Jellyfish) .... p. 8
13. *Creseis virgula* (Curved Needle Pteropod) .... p. 9
14. *Crimora coneja* (Rabbit Dorid Nudibranch) .... p. 9
15. *Dasya binghamiae* (Red Alga) .... p. 9
16. *Delphinus capensis* (Long-beaked Common Dolphin) .... p. 9
17. *Delphinus delphis* (Short-beaked Common Dolphin) .... p. 10
18. *Diphyes dispar* (Siphonophore) .... p. 10
19. *Dirona picta* (Colorful Dirona Nudibranch) .... p. 10
20. *Doriopsilla albopunctata* (White-spotted Sea Goddess Nudibranch) .... p. 10
21. *Doriopsilla fulva* (White-spotted Dorid Nudibranch) .... p. 11
22. *Dosima fascicularis* (Blue Buoy Barnacle) .... p. 11
23. *Emerita analoga* (Mole Crab) .... p. 12
24. *Flabellinopsis iodinea* (Spanish Shawl Nudibranch) .... p. 12
25. *Hancockia californica* (Hancock's Nudibranch) .... p. 13
26. *Hespererato vitellina* (Appleseed Erato Snail) .... p. 13
27. *Hippopodius hippopus* (Siphonophore) .... p. 13
28. *Hyalocylis striata* (Striated Sea Butterfly) .... p. 13
29. *Janolus barbarensis* (Janolus Nudibranch) .... p. 14
30. *Janthina janthina* (Violet Sea Snail) .... p. 14
31. *Janthina umbilicata* (Purple Sea Snail) .... p. 14
32. *Jellyella tuberculata* (Bryozoan) .... p. 15

33. *Lepidochelys olivacea* (Olive Ridley Sea Turtle) .... p. 16
34. *Lissothuria nutriens* (Scarlet Sea Cucumber) .... p. 16
35. *Lottia gigantea* (Owl Limpet) .... p. 16
36. *Megabalanus californicus* (Pink-striped Barnacle) .... p. 17
37. *Mola mola* (Ocean Sunfish) .... p. 18
38. *Navanax inermis* (California Aglaja) .... p. 18
39. *Oceanodroma melania* (Black Storm-Petrel) .... p. 19
40. *Oceanodroma tethys* (Wedge-rumped Storm-Petrel) .... p. 19
41. *Okenia angelensis* (Los Angeles Okenia Nudibranch) .... p. 20
42. *Okenia rosacea* (Hopkins' Rose Nudibranch) .... p. 20
43. *Ophichthus triserialis* (Pacific Snake Eel) .... p. 20
44. *Ophiothrix spiculata* (Glass-spined Brittle Star) .... p. 21
45. *Pachythyone rubra* (Red Sea Cucumber) .... p. 21
46. *Panulirus interruptus* (Spiny Lobster) .... p. 22
47. *Paraconcavus pacificus* (Red-striped Barnacle) .... p. 23
48. *Petaloconchus montereyensis* (Monterey Tube Snail) .... p. 23
49. *Petrolisthes manimaculis* (Chocolate Porcelain Crab) .... p. 24
50. *Phidiana hiltoni* (Hilton's Nudibranch) .... p. 24
51. *Physophora hydrostatica* (Hula Skirt Siphonophore) .... p. 25
52. *Pleuroncodes planipes* (Pelagic Red Crab) .... p. 25
53. *Polycera atra* (Orange-spike Polycera Nudibranch) .... p. 26
54. *Polycera hedgpethi* (Hedgpeth's Nudibranch) .... p. 26
55. *Portunus xantusii* (Xantus' Swimming Crab) .... p. 26
56. *Pseudoceros luteus* (White Flatworm) .... p. 26
57. *Puffinus opisthomelas* (Black-vented Shearwater) .... p. 27
58. *Pyrosoma atlanticum* (Pyrosome) .... p. 27
59. *Sula leucogaster* (Brown Booby) .... p. 28
60. *Synthliboramphus hypoleucus* (Guadalupe Murrelet) .... p. 29
61. *Tetraclita rubescens* (Pink Volcano Barnacle) .... p. 29
62. *Thalia democratica* (Salp) .... p. 29
63. *Thetys vagina* (Salp) .... p. 30
64. *Thylacodes squamigerus* (Scaled Tube Snail) .... p. 30
65. *Triopha maculata* (Spotted Triopha Nudibranch) .... p. 31
66. *Tursiops truncatus* (Common Bottlenose Dolphin) .... p. 31
67. *Velella velella* (By-the-wind Sailor) .... p. 31

B. ACKNOWLEDGEMENTS – List of people, museums, and organizations contributing observations, specimens, and data — pp. 32–33

C. REFERENCES CITED – pp. 34–43

D. SUPPLEMENTARY TABLES

TABLE S1 – Coordinates (latitude, longitude) for all site locations referred to in the species accounts (pp. 44–47)

TABLE S2 – Summary statistics for all marine heatwave events identified from analysis of 2014–2016 sea surface temperature data at the Bodega Bay buoy (p. 48)

TABLE S3 – Summary statistics for all marine heatwave events identified from analysis of 2014–2016 sea surface temperature data at the Point Arena buoy (p. 49)

TABLE S4 – Recorded egg-laying seasons in California and southern Oregon of heterobranch sea slugs found north of their usual ranges during 2014–2016 (p. 50)

E. SUPPLEMENTARY FIGURES (S1–S68) – pp. 51–118

SUPPLEMENTARY INFORMATION:

A. SPECIES ACCOUNTS: Summaries of changes in geographic distribution and abundance during 2014–2017, listed alphabetically by species name:

Notes:

• Museum collections are referenced using the following abbreviations:

ANSP = Academy of Natural Sciences, Drexel University, Philadelphia, PA
CAS-ICH = California Academy of Sciences (Ichthyology), San Francisco, CA
CASIZ = California Academy of Sciences (Invertebrate Zoology), San Francisco, CA
LACM = Natural History Museum of Los Angeles County, Los Angeles, CA
MCZ:IZ = Harvard Museum of Comparative Zoology (Invertebrate Zoology), Cambridge, MA
OS = Oregon State Ichthyology Collection, Oregon State University, Corvallis, OR
RBCM = Royal British Columbia Museum, Victoria, British Columbia, Canada
UAM = University of Alaska Museum of the North, Fairbanks, AK
UC = University Herbarium, University of California, Berkeley, CA
USNM = United States National Museum, Smithsonian Museum of Natural History, Washington, D.C.
YPM IZ = Yale Peabody Museum (Invertebrate Zoology), New Haven, CT

• iNaturalist is an online database where the public and scientists can report species observations (https://www.inaturalist.org/). iNaturalist records are listed with the unique observation number.

---

1. *Acanthinucella spirata* (Angular Unicorn Snail). The northern range limit for this carnivorous snail has often been published as Tomales Bay (Morris et al. 1980, Hellberg et al. 2001). However, populations of *A. spirata* have been recorded in Bodega Harbor since at least the 1940s (Colonel Lee O. Miles, unpublished field notes, California Academy of Sciences). Specimens of *A. spirata* were collected in Bodega Harbor by Col. Miles in 1948–1949 (CASIZ 219753; LACM 160770). *A. spirata* was also recorded in Bodega Harbor during the early 1970s (Standing et al. 1975), the mid-1990s (J.T. Carlton, personal communication to ES and JLS), and was still present at this location in 2017 (JLS, personal observation). In 1946, *A. spirata* was also reported by E.P. Chace at Haven's Neck in Mendocino County, CA (Chace 1948). During June 2017, an intertidal population of *A. spirata* was discovered by Torre Flagor and Paul Bourdeau, just south of Devil's Gate at Cape Mendocino, CA, representing the new northern range record for this species (Fig. S1; Flagor and Bourdeau 2018). This represents a range extension of 260 km for *A. spirata*, which is surprising given that this gastropod has benthic egg capsules with crawl-away, juvenile snails. In the absence of planktonic dispersal, it is possible that egg capsules were carried poleward by rafting (Flagor and Bourdeau 2018), or that this population

was present before 2014, but not previously detected in this remote area of northern California.

2. *Acanthodoris rhodoceras* (Black-tipped Spiny Dorid Nudibranch). This nudibranch formerly had a published range limit of Dillon Beach, CA (Morris et al. 1980), but was observed as far north as Oregon during the 1987–1988 and 1991–1992 El Niño events (Goddard et al. 2016). We observed an increase in the abundance of this species in the Bodega Bay region during 2015 (JLS and ES, personal observations). *Acanthodoris rhodoceras* (eight individuals) was observed in southern Oregon in August 2015 (Goddard et al. 2016), and a single individual was observed in Coos Bay, Oregon, during July 2016 (C.D. Trowbridge, iNaturalist 3775748). A single *A. rhodoceras* was photographed on the south side of the Netarts Jetty in Netarts Bay, Oregon, in June 2015 by Tara Maginnis (University of Portland). Finally, on 21 May 2018, Kathy Johnson observed *A. rhodoceras* at Chup Point in Barkley Sound, British Columbia, a new northern range limit for this species (Fig. S2; Goddard et al. 2018).

3. *Anteaeolidiella oliviae* (Olive's Nudibranch). The published northern range limit of this nudibranch was previously Duxbury Reef, CA (Gosliner and Williams 1970). Single individuals of *A. oliviae* were observed at Coleman Beach on two dates in January 2015, by K. Ueda (iNaturalist 1183371) and Goddard et al. (2016). On 25 November 2015, we also documented one individual at Pinnacle Gulch (ES and JLS, personal observations; Figure S3A). Other *A. oliviae* were observed by Alison Young at Shell Beach during May 2015 (iNaturalist 1527637), and again in February–April 2016 (iNaturalist 2652429, 2880118, 2880217, 2919739). On 16 February 2015, D. Mason photographed one *A. oliviae* in Fort Bragg, CA, which represents the new northern range limit for this species (Fig. S3B; Goddard et al. 2016).

4. *Anthopleura sola* (Sunburst Sea Anemone). Prior to the 1970s, anemones now recognized as *A. sola* (Pearse and Francis 2000) were considered to be a solitary form of *A. elegantissima*. *Anthopleura sola* is a primarily southern species that had a northern range limit of Monterey Bay in the late 1970s (Francis 1979). This species was apparently rare at Hopkins Marine Station in Monterey Bay during the 1930s, but it had increased in abundance at this site by the 1990s (Sagarin et al. 1999). *Anthopleura sola* was absent from a survey conducted at Bodega Marine Reserve in June 1978 (Francis 1979; and L. Francis, personal communication to ES), but was documented in Bodega Bay in the 1990s at Pinnacle Gulch (McFadden et al. 1997). Since 2009, there has been a striking increase in the abundance of *A. sola* in the Bodega Bay region. In November 2009, an informal intertidal survey along the inner 60m of the south side of Horseshoe Cove (Bodega Marine Reserve) documented ~25 *A. sola* (JLS, unpublished data). In July 2013, this area was resurveyed and 338 *A. sola* were recorded along the same 60m of shoreline (Matt Cline, student report, Cadet Hand Library, Bodega Marine Laboratory, University of California Davis). The area was resurveyed again in July 2017, and 521 *A. sola* were recorded along the same 60m of shoreline (Maxine Pontius and Seth Strumwasser, student report, Cadet Hand Library, Bodega Marine Laboratory). In the 2017 survey, 47% of the *A. sola* were smaller individuals (< 6.0 cm basal diameter), suggesting enhanced recruitment of

this species during 2014–2016. Observations from 2016–2018 also suggest that the distribution of *A. sola* has now expanded northward beyond Bodega Bay. In November 2017, we recorded 22 likely *A. sola* along a 135m section of mid intertidal shore at Fort Ross State Park (ES and JLS, unpublished data; Fig. S4A). In May 2018, Maxine Pontius photographed several *A. sola* at Sea Ranch, and S. Strumwasser photographed one large individual at Bruhel Point (Fig. S4B). Smaller *A. sola* can be difficult to distinguish from *A. elegantissima*, so molecular methods are useful to confirm the identification of this species (B.H. Cornwell, unpublished data). The current northern range limit for *A. sola* may be as far north as Trinidad (iNaturalist 10495418), but further surveys and sequencing data are needed to confirm the current poleward limit of this species.

5. *Aplysia californica* (California Sea Hare). This low intertidal to subtidal sea hare occurs on both wave-protected shores and in shallow estuaries and harbors (Morris et al. 1980). *Aplysia californica* is a primarily southern species that is rare north of Monterey Bay, but it has been observed occasionally in San Francisco Bay, Tomales Bay, Bodega Bay, and Humboldt Bay, especially in association with El Niño events (Goddard et al. 2016). In October 1983, following the 1982–1983 El Niño event, *A. californica* was observed as far north as Yaquina Bay, Oregon (Pearcy and Schoener 1987), its current northern range limit. During 2015–2016, there were numerous reports of *A. californica* north of San Francisco. On 23 May 2015, we observed >50 individuals in the low intertidal zone at Miwok Beach (Fig. S5; JLS and ES, personal observations). *Aplysia californica* was reported from Tomales Bay and Coleman Beach on at least seven dates between January 2015 and April 2016 (iNaturalist 1183373, 1410997, 1482509, 1628521, 1633733, 2120968, 2920403). Farther north, *A. californica* was reported from the Trinidad area on two occasions, in November 2016 and February 2017 (iNaturalist 5126092, 5126006). In August 2015, *A. californica* was observed at Gregory Point, Oregon (Goddard et al. 2016). In August 2016, one specimen was found in a water storage reservoir at the Oregon Coast Aquarium, presumably drawn into the reservoir through the seawater intake from Yaquina Bay as a planktonic larva (Goddard et al. 2018).

6. *Arctocephalus townsendi* (Guadalupe Fur Seal). Guadalupe Fur Seals breed primarily at Guadalupe Island, Mexico (Allen et al. 2011). An additional small breeding colony located in the San Benito Islands, Baja California, has expanded since its discovery in 1997 (Aurioles-Gamboa 2015, Carretta et al. 2017). A single pup was born on San Miguel Island in the Channel Islands of southern California in 1997, and 1–3 pups per year have been observed at this breeding site since 2008 (Carretta et al. 2017). Outside the breeding season, *A. townsendi* has been thought traditionally to have a geographic distribution that ranges primarily from Guadalupe Island to the Channel Islands, with occasional vagrants sighted in central and northern California, Oregon, Washington, and Alaska (Allen et al. 2011, Lowe and Sternfeld 2007). Young *A. townsendi* occasionally strand on northern beaches, particularly during El Niño events (Aurioles-Gamboa 2015). In 2015–2016, there was a striking increase in strandings of Guadalupe Fur Seals on California beaches, with approximately 175 strandings compared to typical observations of approximately 10 per year (Carretta et al. 2017). During 2015–2017, small numbers of *A. townsendi* were observed stranded on beaches in Sonoma County, CA (2015 = 3,

2016 = 1, and 2017 = 4 individuals; The Marine Mammal Center, unpublished data; Fig. S6). On 6 September 2015, we also observed an *A. townsendi* near Cordell Bank (JLS, personal observations). While it is clear that there was an increase in strandings and nearshore occurrences of *A. townsendi* during the marine heatwaves, recent studies suggest that, even in ENSO-neutral years, some Guadalupe Fur Seals may range farther north than has been appreciated. In particular, preliminary results of satellite tracking suggest that some Guadalupe Fur Seals may frequent offshore waters along the coast of northern California (Tenaya Norris, unpublished data; personal communication to ES).

7. *Berthella strongi* (Strong's Sidegill). This pleurobranch gastropod formerly had a northern range boundary of Moss Beach, CA (Bertsch et al. 1972). On 4 July 2016, C. Pirrone observed *Berthella strongi* at MacKerricher State Park (iNaturalist 3634325), a new northern record for this species (Fig. S7; Goddard et al. 2018).

8. *Blepharipoda occidentalis* (Spiny Mole Crab). *Blepharipoda occidentalis* is a low intertidal to subtidal crab found on sandy beaches. Its known northern range limit was Drakes Beach in Point Reyes National Seashore (Ricketts and Calvin 1968), until JLS photographed one live *B. occidentalis* at Doran Beach, CA, on 21 June 2005. JLS and ES also found one molt of *B. occidentalis* on Salmon Creek Beach later in 2005. Molts of *B. occidentalis* were not observed at Salmon Creek Beach in 2006–2012, but JLS and ES observed single molts at this site on three dates in November 2013, August 2014, and June 2015 (Fig. S8). In 2017, there was a large increase in the number of *B. occidentalis* molts at Salmon Creek Beach, suggesting an increase in the abundance of this local population. JLS and ES found nine molts in June 2017, 47 molts in August 2017, and six molts in September 2017.

9. *Caulolatilus princeps* (Ocean Whitefish). *Caulolatilus princeps* is a subtropical fish that commonly ranges from the Galapagos Islands to Monterey Bay (Pattie and Baker 1969). Although *C. princeps* is uncommon north of central California, this species has been caught farther north in association with strong El Niño events in the past. In August and September 1957, two *C. princeps* were caught off the Farallon Islands, CA (CAS-ICH 26306; Radovich 1961). In September 1962, *C. princeps* was caught off Redding Rock, CA (CAS-ICH 27009). Several *C. princeps* were caught off the coast of Oregon and Washington during warm-water events in 1951, 1963, and 1965 (Robinson 1965). Finally, *C. princeps* was observed as far north as Vancouver Island, British Columbia, Canada, in association with strong El Niño events during January 1965 (Pattie and Baker 1969) and May 1982 (Canadian Museum of Nature, CMNFI-1982-0418.7). On 10 July 2015, a *C. princeps* was caught by a fisherman off the Farallon Islands (Fig. S9).

10. *Cestum veneris* (Venus' Girdle Ctenophore). *Cestum veneris* is a ctenophore found worldwide in tropical and sub-tropical waters (Harbison et al. 1978, Wrobel and Mills 1998). In the northeast Pacific, *C. veneris* is known from Baja California, Mexico, and southern and central California (Wrobel and Mills 1998), with numerous occurrences recorded by dives of remotely operated underwater vehicles (ROV) in Monterey Bay, at a median depth of ~80m (S.

Haddock/ Monterey Bay Aquarium Research Institute, Video Annotation and Reference System database). Based on this database, the previous northernmost occurrence of *C. veneris* was recorded in Monterey Bay at 36.796°N, -121.844°W. On 1 April 2014, Peter Mieras filmed *C. veneris* at a depth of 12m in Rainy Bay, Barkley Sound, British Columbia, Canada (Fig. S10), a new northern range record for this species in the northeast Pacific. During summer 2016, a second individual was observed by Kathy Johnson near Pill Point, Barkley Sound (P. Mieras, personal communication to ES).

11. *Chelonia mydas* (Green Sea Turtle). *Chelonia mydas* is found worldwide, primarily in tropical and subtropical waters with temperatures >20°C (NMFS and USFWS, 1998a). In the northeastern Pacific, *C. mydas* occurs most commonly from San Diego southward, but this species has been observed as far north as British Columbia, Canada, and Alaska (Hodge and Wing 2000, McAlpine et al. 2004). Such northern observations are often (but not always) associated with El Niño events, including prior records from British Columbia during 1958 (Radovich 1961) and 1998 (McAlpine et al. 2004). During 2014–2016, sightings of *C. mydas* were reported from locations in northern California, including outside the Golden Gate (6 September 2014; Fig. S11; Jones 2014); at Marshall's Beach in San Francisco on 12 September 2015 (iNaturalist 1949313); and in the San Joaquin River (San Francisco Bay-Delta system) on 13 November 2015 (iNaturalist 2464700, Preovolos 2015). *Chelonia mydas* was observed as far north as Wickaninnish Beach, Pacific Rim National Park Reserve, Vancouver Island, British Columbia, Canada, in January 2016 (Johnson 2016).

12. *Chrysaora colorata* (Purple-striped Jellyfish). The distribution of *C. colorata* is restricted to warmer coastal waters of the northeast Pacific where it is commonly found from Baja California, Mexico, to Monterey Bay (Morandini and Marques 2010). Its previous northern range limit was Bodega Bay (Wrobel and Mills 1998, Mills and Larson 2007), based on two specimens recorded by C.E. Mills in Summers 1972 and 1973, in association with the strong 1972–1973 El Niño event. The first specimen was collected in Summer 1972 from Bodega Harbor, and the second specimen was observed on 20 July 1973 off Salmon Creek Beach (C.E. Mills, personal communication to JLS and ES). In October 2006, Mike Kelly photographed *C. colorata* washed ashore on Mad River Beach in Humboldt County, CA (personal communication to ES). During 2014–2017, *C. colorata* was recorded at northern sites on several occasions. On 27 July 2014, JLS observed a single *C. colorata* washed ashore at Horseshoe Cove (Bodega Marine Reserve; Fig. S12A). On 29 August 2014, Mike Kelly photographed a live *C. colorata* in Humboldt Bay (personal communication to ES). On 6 August 2017, S. Miller also photographed a single individual in Bodega Bay at Dillon Beach (personal communication to JLS). On 10 September 2017, Mike Kelly photographed a single individual washed ashore on Mad River Beach (personal communication to ES). On 20 February 2017, Steve Morey recorded two *C. colorata* washed ashore at Arcadia Beach, Oregon (Fig. S12B; iNaturalist 5284085), a new northern range record for this species.

13. *Creseis virgula* (Curved Needle Pteropod). The pteropod *Creseis virgula* is typically a tropical and subtropical species, occurring in warm oceans throughout the world (Bé and Gilmer 1977). In the eastern Pacific, *C. virgula* ranges from Peru to Southern California (Bé and Gilmer 1977), and can sometimes be abundant in plankton tows south of Point Conception (McGowan 1967). To our knowledge, there is only one record of *C. virgula* from north of Point Conception, when this species was found at a single offshore station (~37°N, 130°W) during a California Cooperative Oceanic Fisheries Investigations (CalCOFI) cruise in April 1950 (McGowan 1967). On 4 November 2014, several *C. virgula* (including both adults and veliger larvae) were collected by C.V. Davis and E.B. Rivest in a plankton tow at Bodega Line Station 4 (located ~32 km offshore from Bodega Marine Laboratory), a northern range record for this species in the eastern Pacific (Fig. S13).

14. *Crimora coneja* (Rabbit Dorid Nudibranch). This dorid nudibranch previously had a northern range limit of Cape Arago, Oregon (Goddard 1984). On 29 April 2017, P. Nosler photographed two *C. coneja* in the low intertidal zone at Boiler Bay, Oregon (Fig. S14; Goddard et al. 2018).

15. *Dasya binghamiae* (Red Alga). This mid intertidal to subtidal red alga has a published geographic range of Punta Abreojos, Baja California, Mexico, to Bodega Bay in Marin County, CA (Abbott and Hollenberg 1976). The previous northern range record for *D. binghamiae* was a specimen collected in July 1912 from 0.20 km north of the Estero de San Antonio in Bodega Bay (UC276143). On 18 June 2015, Peter G. Connors collected intertidal *D. binghamiae* from the South Jetty at the Bodega Harbor entrance (UC2056114), the first record from Sonoma County, CA, and the new northern range limit for this species (Fig. S15).

16. *Delphinus capensis* (Long-beaked Common Dolphin). *Delphinus capensis* is found in warm-temperate and tropical coastal waters along many of the world's continents (Allen et al. 2011). In the northeast Pacific, the distribution of *D. capensis* is concentrated along the coast of Mexico, including the Gulf of California, and north to central California (Allen et al. 2011). *D. capensis* is generally associated with warmer water than its congener, the Short-beaked Common Dolphin (*D. delphis*). In 1993 and 2002–2003, several live *D. capensis* were observed for the first time in British Columbia, Canada (Ford 2005). Other than these isolated northern sightings, this species had not been recorded previously north of Monterey Bay (Ford 2005). For example, in surveys conducted during 1996–2014, *D. capensis* was not recorded in northern California, Oregon, or Washington (Barlow 2016). During 2014–2016, numerous *D. capensis* were sighted in northern California in association with warm water conditions. On 26 September 2014, a large pod of *D. capensis* was sighted by Daniel George northeast of Cordell Bank (iNaturalist 904101). On 22 August 2015, Steve Howell also recorded 50–100 *D. capensis* offshore at Cordell Bank (personal communication to JLS). On 18 September 2015, we observed approximately 30 *D. capensis* offshore at Cordell Bank (Fig. S16; JLS, personal observations).

17. *Delphinus delphis* (Short-beaked Common Dolphin). *Delphinus delphis* is found worldwide, primarily in warm-temperate and subtropical waters. In the eastern Pacific, its core geographic distribution extends from Central America northward to Point Conception (Allen et al. 2011). Historically, *D. delphis* was sighted north of Monterey Bay only occasionally (Allen et al. 2011). During 2014–2016, we observed approximately 25–30 *D. delphis* offshore at Cordell Bank on three occasions (7 September 2014, 6 September 2015, 18 September 2015; JLS, personal observations; Fig. S17). During October 2015, record numbers of common dolphins were also observed near the Farallon Islands (Point Blue Conservation Science, unpublished data). On 6 January 2017, a dead *D. delphis* was reported washed ashore at Salmon Creek Beach (iNaturalist 4905297). These observations from northern California were also coincident with reports of an increased abundance of *D. delphis* at more northerly sites. In particular, the estimated abundance of *D. delphis* along the coasts of Oregon and Washington during 2014 was twenty times greater than the mean estimate of abundance during 1996–2008 (Barlow 2016). Although a dead *D. delphis* had been recorded at least once in British Columbia, Canada (Ford 2005), small numbers of live *D. delphis* were recorded for the first time in British Columbia in late September 2015 (off the southwestern coast of Vancouver Island; Pynn 2015).

18. *Diphyes dispar* (Siphonophore). *Diphyes dispar* is typically restricted to warmer regions of the Pacific Ocean (Alvariño 1971). Along the Pacific coast of North America, there are numerous coastal records of this species from Baja California, Mexico, and southern California (Alvariño 1971), including near the Channel Islands (e.g., USNM 28508). *Diphyes dispar* was also collected from Monterey Bay in 1911 (USNM 42119). We are aware of only one prior record of this species from north of Monterey Bay in the northeast Pacific; *D. dispar* was collected at below 1000m from 42°N, off the coast of Brookings, Oregon (Alvariño 1971). This appears to be the northern range record for this species, although this sampling station was >380 km offshore, and thus was not a coastal occurrence. On 17 September 2014, *D. dispar* was collected ~100 km offshore from Point Sur, CA (MCZ:IZ:69767). During 2014–2016, JLS and ES recorded *D. dispar* washed ashore at Salmon Creek Beach on the following 11 dates: 29 August 2014, 14 September 2014 (Fig. S18), 16 September 2014, 27 October 2014, 30 October 2014, 9 February 2015, 17 October 2015, 18 February 2016, 27 February 2016, 6 March 2016, 22 March 2016. On some of these occasions, the distinctive nectophores of this species were common (e.g., >50 nectophores/day encountered).

19. *Dirona picta* (Colorful Dirona Nudibranch). This nudibranch formerly had a northern range limit of Cape Meares, Oregon (Goddard 1997). On 6 July 2016, C. Tamis recorded *D. picta* near Bamfield, British Columbia, Canada (Fig. S19; Merlo et al. 2018), a new northern range record for this species (Goddard et al. 2018).

20. *Doriopsilla albopunctata* (White-spotted Sea Goddess Nudibranch). *Doriopsilla albopunctata* is a southern species that is rarely recorded north of San Francisco, and is less common than its congener *D. fulva* in intertidal habitats north of Point Conception (Goddard et al. 2016). We recorded single specimens of *D. albopunctata* at Bodega Marine Reserve on 15 July 2014 and 16 February 2015 (JLS and ES, personal observations). We recorded a single *D.*

albopunctata at Pinnacle Gulch on 8 July 2016, and three individuals at this site on 13 July 2017. We also recorded three *D. albopunctata* at Salt Point on 14 July 2017. *Doriopsilla albopunctata* was previously known to occur as far north as Mendocino County, CA, including Van Damme State Park (Beeman and Williams 1980) and Mendocino, CA (Hoover et al. 2015). In May 2017, N. Treneman recorded a single specimen of *D. albopunctata* at Whiskey Creek, Oregon, representing a new northern range record (Fig. S20; Goddard et al. 2018).

21. *Doriopsilla fulva* (White-spotted Dorid Nudibranch). *Doriopsilla fulva* is typically uncommon north of San Francisco and previously had a northern range limit of Abalone Beach in Humboldt County, CA (Jaeckle 1984). Beginning in February 2015, we observed an increase in abundance of *D. fulva* in the Bodega Bay region relative to 2004–2014 (JLS and ES, personal observations). From 2015–2017, this species was recorded at numerous intertidal sites in central and northern California (iNaturalist records), often in record abundance (Goddard et al. 2016). This nudibranch continued to be common in northern California during 2017, and we recorded 12 individuals at Salt Point on 14 July 2017, and 37 individuals at Salt Point on 13 July 2018 (ES and JLS, personal observations). *Doriopsilla fulva* was recorded in Oregon for the first time in June 2015, at Whiskey Creek (Goddard et al. 2016). During April–August 2016 and June 2017, Todd Cliff recorded *D. fulva* farther north, in Netarts Bay, Oregon, its new northern range limit (Fig. S21; Goddard et al. 2018).

22. *Dosima fascicularis* (Blue Buoy Barnacle). Although this pelagic barnacle has a cosmopolitan distribution (Weisbord 1979), in the north Pacific it is most commonly associated with warmer coastal waters and offshore waters. *Dosima fascicularis* is common in the diet of offshore Shooty Shearwaters (*Puffinus griseus*) in the central north Pacific (Shiomi and Ogi 1992, Gould et al. 2000), especially at lower latitudes (35–40°N, Shiomi and Ogi 1992). In coastal waters of the northeast Pacific, *D. fascicularis* is found in the greatest abundance in the warmer waters of southern California (Knudsen 1962). For example, >1,900 *D. fascicularis* were collected when a 1,000 m stretch of beach in La Jolla, CA, was surveyed on five successive dates (Cheng and Lewin 1976). *Dosima fascicularis* has occasionally washed ashore in northern California in small numbers, including specimens from Dillon Beach in June 1948 (CASIZ 87489) and Spring 1956 (CASIZ 56379, 87485, 87490), and near Humboldt Bay in June 1936 (CASIZ 21990). Many (but not all) of the coastal records of *D. fascicularis* from north of California are associated with El Niño events. For example, *D. fascicularis* was collected from Coos Bay, Oregon, in May 1942, during the strong 1941–1942 El Niño event (CASIZ 21989). Specimens were also collected ~250 km offshore from Tillamook Bay, Oregon (CASIZ 115147), by David L. Stein in July 1973 during the strong 1972–1973 El Niño. During 1926, *D. fascicularis* was collected at Copalis Beach, Washington (CASIZ 15487, 15501, 29157), from north of Vancouver Island, British Columbia, Canada (CASIZ 15473), and at Biorka Island in Southeast Alaska (USNM 78367). These occurrences were associated with the strong 1925–1926 El Niño event. However, there are also a handful of records of *D. fascicularis* from northern sites that have occurred during ENSO-neutral years. These include collections, for example, from British Columbia (USNM 78368, 78369) and Southeast Alaska (UAM 10760, 10761) in 1934 and 1994,

respectively. The northern record for *D. fascicularis* appears to be specimens collected in April 1940 from Salisbury Sound, Alaska (UAM 12262). During 2014–2017, we found *D. fascicularis* washed ashore at Salmon Creek Beach on ten dates: 30 August 2014, 6 September 2014, 21 September 2014, 28 May 2015, 14 June 2015, 5 August 2015, 23 January 2016, 6 March 2016, 20 April 2016, and 27 April 2017 (Fig. S22A; JLS and ES, personal observations). The numbers of individuals found were small (generally 2–6 individuals/date, but occasionally as many as 25). On 26 March 2016, S. Morey also found two specimens washed ashore at Arcadia Beach, Oregon (Fig. S22B; personal communication to ES). During 2014, *D. fascicularis* was also recorded at several sites in British Columbia. On 11 July 2014, Jackie Hildering, Robin Abernethy, and Jared Towers recorded *D. fascicularis* about 65 km offshore from the southern end of Moresby Island, British Columbia (Fig. S22C; J. Hildering, personal communication to ES). On 5 August 2014, J. Hildering found *D. fascicularis* washed ashore on the southwest shore of Princess Royal Island, British Columbia (J. Hildering, personal communication to ES).

23. *Emerita analoga* (Mole Crab). This suspension-feeding crab is found on intertidal sandy beaches from Baja California, Mexico, to Kodiak Island, Alaska (Jensen 2014). However, *E. analoga* is most abundant in the southern part of its geographic range between Ensenada, Baja California, Mexico, and central California (Ricketts and Calvin 1968). During previous El Niño events, larvae were sometimes transported northward in abundance leading to high densities of juvenile *E. analoga* on beaches in Oregon, Washington, and British Columbia, Canada (Ricketts and Calvin 1968, Sorte et al. 2001, Wonham and Hart 2018). The first record of *E. analoga* in Washington State was coincident with the strong 1941–1942 El Niño event (Banner and McKernan 1943). Similarly, *E. analoga* was first recorded in British Columbia in June 1959, following the strong 1957–1958 El Niño event (Radovich 1961). During 2016–2017, *E. analoga* was reported on beaches in Washington (iNaturalist 6014202, 7297400) and British Columbia (iNaturalist 4972976). Wonham and Hart (2018) documented *E. analoga* (including gravid females) as far north as the west coast of Vancouver Island, British Columbia. Relative to 2004–2013, we observed a massive increase in the density of juvenile *E. analoga* at Salmon Creek Beach, Pinnacle Gulch, and other local beaches in the Bodega Bay region during 2014–2017 (ES and JLS, personal observations; Fig. S23).

24. *Flabellinopsis iodinea* (Spanish Shawl Nudibranch). Although the northern range limit for this nudibanch is Vancouver Island, British Columbia, Canada (Bernard 1970), this is a southern species that is rarely found north of Monterey Bay (Goddard et al. 2016). We recorded a total of three *F. iodinea* at Pinnacle Gulch (18 and 19 May 2015; Fig. S24), and Coleman Beach (23 May 2015) (ES and JLS, personal observations). *Flabellinopsis iodinea* was reported from Trinidad on eight dates between April 2015 and July 2016 (e.g., iNaturalist 3751571, 1387158), and 23 individuals were observed at this location on 4 July 2015 (Goddard et al. 2016). *Flabellinopsis iodinea* was observed as far north as Cape Flattery, Washington, during August 2015 (Goddard et al. 2018).

25. *Hancockia californica* (Hancock's Nudibranch). *Hancockia californica* is typically not found north of Dillon Beach, CA, with only a single previous record from Trinidad, CA, reported by Jaeckle (1984). On 23 May 2015, we found one individual at Coleman Beach (Fig. S25A; JLS and ES, personal observations) and on 7 June 2015, A. Young found one individual at Shell Beach (iNaturalist 1604528). During August 2015, at least three *H. californica* were recorded at the previously documented northern range limit in Trinidad (Fig. S25B; Goddard et al. 2016; and iNaturalist 1833410, 1908069, 1908082).

26. *Hespererato vitellina* (Appleseed Erato Snail). *Hespererato vitellina* is a low intertidal and subtidal snail, with a published northern range limit of Bodega Bay, CA (Morris et al. 1980). *Hespererato vitellina* was observed at Shell Beach, CA, on 6 February 2016 and 6 April 2016 by Alison Young (iNaturalist 2652426, 2880086), and a second *H. vitellina* shell was photographed at Fort Ross State Park, CA, on 9 June 2016 by A. Young (iNaturalist 3423549). JLS and ES found a single empty *H. vitellina* shell at Cape Mendocino, CA, on 25 May 2017 (Fig. S26; CASIZ 224108), a new northern range record for this species.

27. *Hippopodius hippopus* (Siphonophore). The siphonophore *Hippopodius hippopus* is most commonly found in warmer, tropical waters (Mapstone 2009). It is easily recognized by its distinctive horseshoe-shaped nectophores (Kirkpatrick and Pugh 1984). The northernmost record for *H. hippopus* in the northeast Pacific was formerly 38°N (Alvariño 1971). However, this record was from far offshore (> 800 km). Coastal records of this species in the northeast Pacific appear to be restricted to locations south of Point Conception (Alvariño 1971). In March 1993, *H. hippopus* was collected 200 km offshore of Point Conception (Haddock and Case 1999), and this species has been recorded at several locations near the coast of Baja California, Mexico (Alvariño 1971). *Hippopodius hippopus* is not known from collections in Monterey Bay (P. Pugh, personal communication to JLS). On six dates during Spring 2015 and Spring 2016 (3 March 2015, 29 March 2015, 10 April 2015, 4 June 2015, 22 March 2016, 16 April 2016), JLS and ES recorded one to three nectophores of *H. hippopus* washed ashore at Salmon Creek Beach, the new northern range record for this species in the northeast Pacific (Fig. S27).

28. *Hyalocylis striata* (Striated Sea Butterfly). The pteropod *Hyalocylis striata* is typically a tropical species, occurring in both the tropical and subtropical Pacific and Atlantic Oceans (Tesch 1946, Tesch 1948, McGowan 1967, Bé and Gilmer 1977). California Cooperative Oceanic Fisheries Investigations (CalCOFI) plankton tows conducted along the west coast of North America in November 1949 recorded *H. striata* at only a single station, off the southern coast of Baja California, Mexico (~24.2°N; McGowan 1967). *Hyalocylis striata* has occasionally been recorded in more temperate regions of the central Pacific, e.g., ~2,000 km west of the California coast (35°30'N, 145°00'W; Tesch 1948). We identified three specimens collected along the California coast in late 2014, which to our knowledge are the first records of this pteropod from California. The first specimen (shell length ~4.8 mm) was collected on 24 September 2014 by C.V. Davis, E.B. Rivest, J. Jahncke, and Point Blue Conservation Science in a vertical plankton tow (400m depth to surface) at Bodega Canyon (Fig. S28A; CASIZ 202355). The second specimen

(Fig. S28B; CASIZ 202356) was a juvenile *H. striata* (shell length ~0.9 mm) collected on 21 October 2014 by C.A. Vines, D. Dann, and M.G. Susner in a vertical tow (15m depth to surface) at the Bodega Marine Laboratory oceanographic buoy (located 1.2 km offshore from the laboratory). This specimen represents the northern geographic record for this species. Finally, another juvenile *H. striata* (shell length ~0.33 mm) was collected by C.V. Davis and E.B. Rivest on 4 November 2014 in a plankton tow at Bodega Line Station 4 (located ~32 km offshore from Bodega Marine Laboratory).

29. *Janolus barbarensis* (Janolus Nudibanch). This nudibranch is rare north of Morro Bay, CA, (Goddard et al. 2016), but has been reported previously in San Francisco Bay (Jaeckle 1983). *Janolus barbarensis* was observed in San Francisco Bay in July–August 2015 (Goddard et al. 2016). It was subsequently recorded in Bodega Harbor on 11 September 2015 by S. Brumbaugh and C.K. Kwan, and on 12 September 2015 by ES and JLS (Fig. S29). These observations in Bodega Harbor represent a new northern range record for *J. barbarensis*.

30. *Janthina janthina* (Violet Sea Snail). *Janthina janthina* is a pelagic snail that occurs worldwide in tropical and temperate waters (Beu 2017). In the northeast Pacific, *J. janthina* is considered "extremely rare" in California (Berry 1958), and is generally only found washed ashore south of Point Conception (Burch 1945). Along the west coast of North America, we are aware of only three prior records of *J. janthina* north of Point Conception. In October 1960, V.L Yadon collected a single specimen in Pacific Grove (CASIZ 135161). In 1935, June Tapscott collected a single specimen on Salmon Creek Beach (CASIZ 135160). Finally, Florian Von Eschen collected a single specimen of *J. janthina* (USNM 365148) in Neskowin, Oregon, the northern range record for this species (Fig. S30A). Although no collection date is available, this latter specimen was deposited in the USNM in July 1926 and thus may have been associated with the strong 1925–1926 El Niño event. During spring 2016, JLS and ES observed four specimens of *J. janthina* washed ashore. Specifically, single specimens were collected on 12 March 2016 and 22 April 2016 on Salmon Creek Beach (Fig. S30B). An additional two specimens were collected on 23 April 2016 in Horseshoe Cove (Bodega Marine Reserve; CASIZ 224106).

31. *Janthina umbilicata* (Purple Sea Snail). *Janthina umbilicata* is a pelagic snail that is cosmopolitan in warm seas (Beu 2017). *J. umbilicata* is considered the most common and abundant janthinid species in offshore waters along the coasts of Baja California, Mexico, and California (Savilov 1969). However, it is rarely observed on beaches north of Monterey Bay (Burch 1945). We are aware of five records of *J. umbilicata* from the Bodega Bay region prior to 2015. In March 1938, a large number of *J. umbilicata* washed ashore in Bodega Bay (Burch 1945), including 30 specimens collected on Salmon Creek Beach by June Tapscott (CASIZ 135155). From Horseshoe Cove (Bodega Marine Reserve), Paul Bettendorf collected one specimen on 13 April 1978 (Bodega Marine Lab synoptic collection), D.A. Fishlyn collected one specimen in 1979 (J.T. Carlton, personal communication to ES and JLS), and JLS collected two specimens on 18 June 2005. The Bodega Marine Reserve formerly belonged to a ranch owner, Rose Gaffney, who also collected nine *J. umbilicata* at this site sometime prior to 1962 (Bodega Marine Laboratory synoptic collection). During 2016, an unprecedented number of *J. umbilicata* washed ashore in the Bodega Bay region. During January–May 2016, JLS and ES

encountered *J. umbilicata* on 22 dates and collected >3,000 individuals from Horseshoe Cove, and especially Salmon Creek Beach. This included at least five separate mass stranding events where from 100 to >400 individuals were found washed ashore on a given day (e.g., 22 April 2016, CASIZ 224107).

The prior northern record of *J. umbilicata* is two specimens collected by Florian Von Eschen in Neskowin, Oregon (USNM 365147). Although no collection date is available, the specimens were deposited in the USNM in July 1926 and thus may have been associated with the strong 1925–1926 El Niño event. A specimen of *J. umbilicata* (ANSP 320948) collected prior to 1891 is associated with the locality of Icy Cape, Alaska, in the Arctic Pacific. However, several lines of evidence suggest that this specimen is likely mislabeled, and we regard Neskowin, Oregon, as the prior northern range record for this species. There were numerous observations of *J. umbilicata* at northern sites during 2015–2016. On 8–9 April 2015, >60 individuals were collected by Mike Kelly on Mad River Beach in Arcata, CA (personal communication to ES). Also in April 2015, Charlie Plybon found ~12 *J. umbilicata* just north of Lost Creek State Park on the central Oregon coast (personal communication to ES). On 14 March 2016, Diane and Dave Bilderback found *J. umbilicata* washed ashore in Bandon, Oregon (personal communication to ES). In March 2016, Jan Hodder observed hundreds of *J. umbilicata* washed ashore (personal communication to ES) on Lighthouse Beach in Coos County, Oregon (CASIZ 2272800). On 27 March 2016, June Jacobson found >100 individuals washed ashore just south of the mouth of the Nestucca River, Oregon (personal communication to JLS). On 21–22 November 2015, Russ Lewis found five large specimens of *J. umbilicata* washed ashore at Leadbetter Point on the Long Beach Peninsula, Washington, representing a new northern range record for this species (Fig. S31A; CASIZ 227279).

Some populations of *J. umbilicata* appear to have persisted in northern regions beyond the 2014–2016 marine heatwaves, and perhaps were associated with minor warm temperature anomalies in early 2017 (Wells et al. 2017). JLS and ES observed very small numbers (1–6 individuals) of *J. umbilicata* washed ashore at Salmon Creek Beach on a handful of dates during early 2017 and early 2018 (on 24 January 2017, 13 March 2017, 25 April 2017, 25 January 2018, 16 March 2018; Fig. S31B). Somewhat larger numbers (~50) of *J. umbilicata* were also observed at more northern sites during winter 2018, including near Seal Rock, Oregon (14 January 2018; C. Plybon, personal communication to ES), and at Leadbetter Point, Washington (6 February 2018; R. Lewis, personal communication to ES).

32. *Jellyella tuberculata* (Bryozoan). *Jellyella tuberculata* (formerly *Membranipora tuberculata*) is known “from warm temperate and tropical seas worldwide” (Tilbrook et al. 2001). In the eastern Pacific, it is distributed from Peru to northern California (Soule et al. 2005). Although it has been suggested that its occurrence in central/northern California is linked to El Niño events (Soule et al. 2007), we have found *J. tuberculata* to be reliably present in the low intertidal zone at Pinnacle Gulch and Dillon Beach since our first search for this species in 2011 (D.S. Swezey and ES, personal observations). This bryozoan appears to reach its northern range limit at Bodega Bay, CA (Swezey et al. 2017). In Fall 2016, there was a striking increase in the abundance of *J. tuberculata* at Pinnacle Gulch relative to 2011–2014, with colonies blanketing extensive patches of algae in the low intertidal zone (Fig. S32; ES and JLS,

personal observations). *J. tuberculata* has remained common on algae at this site through our most recent survey in November 2018.

33. *Lepidochelys olivacea* (Olive Ridley Sea Turtle). *Lepidochelys olivacea* is found worldwide, primarily in tropical and subtropical waters (NMFS and USFWS, 1998b). In the eastern Pacific, *L. olivacea* occurs most commonly from Peru to southern California, but this species is observed occasionally stranded on beaches in northern California, Oregon, and Washington, and has been recorded previously as far north as Alaska (Hodge and Wing 2000). On 15 January 2016, we observed a fresh carcass of *L. olivacea* washed ashore at Salmon Creek Beach (Fig. S33; JLS and ES, personal observations). This observation during winter 2015–2016 was coincident with at least nine strandings of *L. olivacea* in Oregon and Washington, an unprecedented number of northern records for a single year (Valdes 2016).

34. *Lissothuria nutriens* (Scarlet Sea Cucumber). The northern range limit of this low intertidal to subtidal sea cucumber has long been recognized as Bodega Head (Wootten 1949), although it has been rare at this site historically (Rutherford 1975). ES and JLS found a single individual on Bodega Head in July 2010, but saw no others during 2004–2014. In contrast, in March 2017, ES counted 76 *L. nutriens* in a 60-minute search of the low intertidal zone in Bodega Marine Reserve. *Lissothuria nutriens* was still abundant at this site in December 2017 when ES counted 98 individuals in a 45-minute search. On 25 June 2017, one of us (J.H.R. Goddard) photographed one *L. nutriens* in the low intertidal zone near Fort Ross Reef, a new northern range record for this sea cucumber (Fig. S34; iNaturalist 6877999).

35. *Lottia gigantea* (Owl Limpet). *Lottia gigantea* is a territorial, mid to high intertidal limpet that ranges from Baja California, Mexico, to Point Cabrillo in northern California (Fenberg and Rivadeneira 2011). *Lottia gigantea* reaches its highest density in southern California and has historically occurred in low abundance north of Monterey Bay (Fenberg and Rivadeneira 2011). In the vicinity of Bodega Bay, there are museum specimens of *L. gigantea* collected from Tomales Point in June 1941 (CASIZ 227845), Duxbury Reef in July 1947 by Colonel Lee O. Miles (CASIZ 227846), Dillon Beach in 1948 (LACM 48-3.16), and Bodega Bay in 1951 (USNM 664156). We are not aware of museum samples from the Bodega Bay region collected after 1951. In 1964, there was a small population of large *L. gigantea* at Duxbury Reef that dwindled to a few remaining individuals by the early to mid-1970s (Armand Kuris, personal communication to ES). Otherwise, *L. gigantea* appears to have been scarce or absent in this region during the 1960s and 1970s. In particular, *L. gigantea* was not observed in the Bodega Marine Reserve (BMR) during the 1970s (Ristau et al. 1978; J.T. Carlton and A. Kuris, personal communication to ES). When we first began working in BMR in 2005, a small population of *L. gigantea* was present (JLS and ES, personal observations). Our 2010 survey at this site documented only larger individuals with no evidence of recent recruitment (Figure 4). During 2011–2014, we noted the loss of some larger *L. gigantea* from the BMR population, but never recorded the presence of small individuals (JLS and ES, unpublished data). This observation is consistent with surveys conducted at other sites in this region during 2004–2006, which also revealed little evidence of

recent recruitment in northern California. In particular, no juveniles (<25 mm length) were found at Dillon Beach, CA, or six sites north of that location (Fenberg and Rivadeneira 2011). As indicated by our survey data (Figure 4), large numbers of *L. gigantea* recruited to BMR during winter 2014–2015 and winter 2015–2016 (Fig. S35). We also documented a high percentage of juvenile limpets in other nearby populations during 2016–2017 (JLS and ES, unpublished data), indicating widespread recruitment of *L. gigantea* in this region in association with warm water conditions during 2014–2016. Historically, the northern range limit for *L. gigantea* was Crescent City, CA, based on museum specimens collected in four different years: 1889, 1935, 1957, and 1963 (Fenberg and Rivadeneira 2011). However, Fenberg and Rivadeneira (2011) did not find *L. gigantea* present at Crescent City during surveys conducted in 2003, 2005, and 2011. Similarly, we were unable to locate any *L. gigantea* at Crescent City during extensive searches in May 2017 (JLS and ES, personal observations). Thus, despite increases in the abundance of *L. gigantea* in northern California during 2014–2017, it appears that the current northern range limit for this species remains in Mendocino County, CA (Fenberg and Rivadeneira 2011; JLS and ES, unpublished data). Note that the northern range limit for *L. gigantea* has been listed as Neah Bay, Washington, in some publications (McLean 1966, Morris et al. 1980). This record stems from observations by Robert Talmadge, who reported finding two *L. gigantea* in Neah Bay, presumably in the late 1950s or early 1960s (R. Talmadge, personal communication to J.T. Carlton). Although there are no known specimens or photographs associated with this rare northern occurrence, R. Talmadge was an accomplished naturalist and malacologist, so the identification and record are likely valid (J.T. Carlton, personal communication to ES).

36. *Megabalanus californicus* (Pink-striped Barnacle). *Megabalanus californicus* is a southern barnacle, usually regarded as “very uncommon north of Monterey” (Newman and Abbott 1980). Its published northern range limit is Humboldt Bay (Zullo 1968), based on specimens collected from buoys in this location in 1939 (CASIZ 24476). *Megabalanus californicus* was reported at Cordell Bank during 1980 and 1981 (Newman and McConnaughey 1987). In 1996, two *M. californicus* shells were observed washed ashore in Horseshoe Cove (Bodega Marine Reserve) by J.T. Carlton. In September 2006, ES and JLS recorded 22 *M. californicus* growing on the Bodega Marine Laboratory oceanographic buoy (deployed ~1.2 km offshore from the marine laboratory), and in April 2009, ES observed *M. californicus* growing on low intertidal mussel shells at Tomales Point. *Megabalanus californicus* shells and plates were occasionally seen washed ashore in Bodega Marine Reserve during 2004–2014 (ES and JLS, personal observations).

During 2015–2017, there was a noticeable increase in the local abundance of *M. californicus* in Bodega Bay. During this period, live *M. californicus* was observed growing on low intertidal mussel shells, kelp holdfasts, and a crab carapace (*Pugettia producta*) in Bodega Marine Reserve, and *M. californicus* shells and plates were routinely found washed ashore (JLS and ES, personal observations). In March 2016, we recorded >500 *M. californicus* growing on the Bodega Marine Laboratory oceanographic buoy (JLS and ES, personal observations). On 11 December 2014, the St. Georges oceanographic buoy (NOAA Buoy 46027, located 14.8 km

northwest of Crescent City, CA) was dislodged from its mooring. This buoy washed ashore three days later in Cape Sebastian, Oregon, where Nancy Treneman photographed at least nine *M. californicus* growing on mussels attached to the buoy. On 5 June 2015, a single *M. californicus* was photographed by N. Treneman in the low rocky intertidal zone at Humbug State Park, Oregon (Fig. S36), representing the current northern range record for this species. In addition, during July–September 2015, several clusters of *M. californicus* were collected by N. Treneman and David Bilderback from bull kelp holdfasts that had washed ashore in Bandon, Oregon, suggesting that this species might have settled north of Humbug State Park (although it is not possible to identify where the bull kelp originated). During El Niño periods, *M. californicus* has been observed on the hulls of ships moored in British Columbia, Canada, including specimens collected in 1925 (CASIZ 094120; and RBCM A-269-00001) and 1958 (RBCM 979-11147-256). However, there is no definitive evidence of this species occurring on benthic substrate in British Columbia (but see Cornwall 1955, Carlton et al. 2011).

37. *Mola mola* (Ocean Sunfish). *Mola mola* is a large, pelagic fish that occurs globally in tropical and temperate waters. In some regions, *M. mola* undergoes seasonal migrations to track warmer water (Sims et al. 2009). In the eastern Pacific, *M. mola* ranges from Chile to the Gulf of Alaska (Love et al. 2005). Unusual past occurrences of *M. mola* in the coastal waters of Washington, British Columbia, Canada, and Alaska have been associated with warm water temperatures during strong El Niño events, including those in 1925–1926, 1982–1983, and 1997–1998 (Hubbs 1948, Pearcy and Schoener 1987, Mecklenburg et al. 2002, Brodeur et al. 2006). During 2014–2015, we noted an increase in abundance of *M. mola* during offshore pelagic trips to Bodega Canyon/Cordell Bank (Fig. S37; JLS, personal observations) relative to prior trips during 2004–2013. During 2014–2015, we also observed an increase in the number of *M. mola* washed ashore on Salmon Creek Beach (JLS and ES, personal observations). Unusual occurrences of *M. mola* were also documented as far north as Alaska in 2014 (Cavole et al. 2016).

38. *Navanax inermis* (California Aglaja). *Navanax inermis* is a southern heterobranch gastropod (Family Aglajidae) that is found on soft and hard substrata, where it preys on other heterobranchs, particularly bubble snails and soft-bodied nudibranchs. *Navanax inermis* is most common in southern California, but has been observed occasionally in Elkhorn Slough (Ricketts and Calvin 1968). *Navanax inermis* was also reported previously from Bolinas Lagoon and Bodega Bay in association with El Niño events (Goddard et al. 2016). In particular, Standing et al. (1975) reported that *N. inermis* was rare to common in Bodega Harbor during a sampling period that included the strong 1972–1973 El Niño event. On 4 June 2015, *N. inermis* was observed by Grace Ha in Bodega Harbor, its northern range limit (JLS and ES, personal observation). *Navanax inermis* was recorded again in Bodega Harbor during June, July, and August 2016 by JLS, M. Whalen, and Grace Ha, respectively (Fig. S38). During 2015–2016, this species was also noted in Drakes Estero (iNaturalist 3871263) and Tomales Bay (iNaturalist 2354769, 2358770, 4594938).

39. ***Oceanodroma melania*** **(Black Storm-Petrel).** *Oceanodroma melania* nests on islands in Baja California, Mexico, and southern California and then disperses northward after the breeding season, primarily to southern and central California (Ainley 2008). Although *O. melania* regularly occurs in Monterey Bay, only small numbers have historically been seen farther north, including Cordell Bank (Howell 2012). However, *O. melania* has become more abundant in offshore waters north of Monterey Bay since 2007 (Ainley 2008, eBird 2017), a trend seen in some other primarily southern seabirds such as *Sula leucogaster* (Davis et al. 2017). The abundance of *O. melania* off the California coast is also considered a strong indicator of warm-water anomalies, with large population increases observed during previous El Niño events (Ainley 1976, Hyrenbach and Veit 2003, Spear and Ainley 2007). Compared to historical records of seabird observations over the past 36 years, there were notable increases in the frequency and abundance of *O. melania* recorded north of Monterey Bay during 2014 and 2015. In particular, *O. melania* was observed during offshore boat trips at Bodega Canyon/Cordell Bank on at least eight dates in 2014 and six dates in 2015 (Fig. S39; eBird 2017). On four of those trips, 50 or more *O. melania* per trip were reported: >50 on 26 August 2014; 315 on 19 September 2014; 50 on 22 August 2015; and 70 on 18 September 2015 (eBird 2017; Shearwater Journeys, unpublished data; JLS, personal observation). During 2014–2015, there were also unusual occurrences of *O. melania* farther north. On 27 September 2015, eight Black Storm-Petrels were observed off Fort Bragg, representing the first record of this species in Mendocino County, CA (Davis et al. 2017). Prior to 2014, *O. melania* had been recorded in Oregon on only three dates. The first record was off Seaside, Oregon, on 8 September 1983, in association with the strong 1982–1983 El Niño event (Oregon Bird Records Committee 2018). During 2014–2015, single *O. melania* were observed off the Oregon coast on three dates [off Newport, Oregon, on 16 August 2014; off Brookings, Oregon, on 16 September 2014 (Waggoner and Merrill 2017); and off Curry County, Oregon, on 1 August 2015 (Irons et al. 2016)].

40. ***Oceanodroma tethys*** **(Wedge-rumped Storm-Petrel).** This seabird breeds on the Galapagos Islands and along the coast of Peru. Densities of *Oceanodroma tethys* are positively correlated with water temperature (Spear and Ainley 2007). This species' geographic distribution at sea extends northward to offshore of the coast of Baja California, Mexico, although it is considered uncommon to rare in this region (Howell 2012). Prior to 2015, there were only eight records of *O. tethys* (all single birds) from the state of California (California Bird Records Committee 2007, Tietz and McCaskie 2018). Five of these observations were recorded south of Point Conception, and the remaining three were recorded in Monterey County, CA, in association with El Niño events (January 1969, October 1977, and October 1983). In 2015–2016, *O. tethys* were observed north of Monterey Bay for the first time. On 19 April 2015 and 20 May 2015, single *O. tethys* were captured at Southeast Farallon Island (Searcy et al. 2018). Another individual was observed at Cordell Bank on 22–23 August 2015 (Searcy et al. 2018; Steve Howell, personal communication to JLS; Fig. S40). On 1 May 2016, D. Kammerichs-Berke recovered a dead *O. tethys* at South Spit, Humboldt Bay, representing a new northern range record for this species (Kammerichs-Berke 2018).

41. *Okenia angelensis* (Los Angeles Okenia Nudibranch). *Okenia angelensis* is a southern species that had been recorded as far north as San Francisco Bay on only a single occasion in September 1964 (Lance 1966). In July 2015, *O. angelensis* was reported from Pillar Point Harbor in Half Moon Bay, CA (iNaturalist 1773046, 1774128; Goddard et al. 2016). On 4 May 2017, Colby Davidson found a single individual at Miwok Beach, CA, a new geographic range record for this species (Fig. S41; H. Bewley and C. Davidson, personal communication to JLS).

42. *Okenia rosacea* (Hopkins' Rose Nudibranch). *Okenia rosacea* is rarely seen north of San Francisco, but was reported as far north as Cape Arago, Oregon, during the strong 1997–1998 El Niño (Goddard et al. 2016; iNaturalist 2982117). We first observed *O. rosacea* (14 individuals) in Bodega Marine Reserve on 3 January 2015, after having observed no individuals north of San Francisco during 2004–2014 (JLS and ES, unpublished data). *Okenia rosacea* was recorded in unprecedented numbers at many sites in northern California and Oregon during 2015–2016 (Goddard et al. 2016; iNaturalist records). In May–July 2015, *O. rosacea* was found at Cape Arago and Gregory Point, Oregon, its current northernmost range record (Goddard et al. 2016). *O. rosacea* persisted in northern California during 2017 and 2018, and ES and JLS observed this species in Bodega Marine Reserve on 12 February 2017 (nine individuals); at Salt Point State Park on 24 February 2017 (Fig. S42; 16 individuals), 14 July 2017 (five individuals), and 16 July 2018 (two individuals); and at Patrick's Point State Park on 31 May 2017 (three individuals). Mike Kelly observed also observed ~10 individuals at Patrick's Point State Park on 31 December 2017 (personal communication to ES), and R. Agarwal reported two individuals at this site on 15 July 2018 (iNaturalist 14447221).

43. *Ophichthus triserialis* (Pacific Snake Eel). *Ophichthus triserialis* is primarily a subtropical eel with a published geographic range from Peru to northern California (Love et al. 2015). It is considered rare north of Baja California, Mexico (Eschmeyer and Herald 1983), and we are aware of relatively few historical records of *O. triserialis* from north of Point Conception. Three specimens were collected in San Francisco Bay in 1931, 1933, and 1948 (CAS-ICH 19770, 11062, 23715, respectively). From north of San Francisco Bay, a specimen was collected from Tomales Bay in 1916 or before (Hubbs 1916). In September 1977, a specimen was caught off Bodega Bay (CAS-ICH 40255). The prior northern record for *O. triserialis* was a 90 cm individual caught offshore of the Klamath River in October 1972 during a strong El Niño event (Quirollo and Dinnel 1975). On 4 May 2014, T. Carter found a dead specimen of *O. triserialis* (89 cm long) washed ashore on Dillon Beach (Fig. 43A; C. Vogler, personal communication to JLS). In early March 2017, one *O. triserialis* was found washed ashore on a beach near Bandon, Oregon (Kelsey Adkisson, Oregon Department of Fish and Wildlife, personal communication to ES). On 21 January 2017, J. Hill found a specimen (101 cm long) in Lincoln City, Oregon (Fig. 43B; Oregon State Ichthyology Collection, OS 20112), representing a new northern range record for this species (Pickens et al. 2018). Lea and Rosenblatt (2000) suggested that larval *Ophichthus* might be transported north from Baja California with anomalous northward currents associated with El Niño events. The occurrence of large *O. triserialis* (89-101 cm length) at northern locations during 2014 and 2017 suggests that adult *Ophichthus* may also migrate northward during warm-water events.

44. *Ophiothrix spiculata* (Glass-spined Brittle Star). This brittle star formerly had a published northern range limit of San Mateo County, CA (Morris et al. 1980). However, subtidal *O. spiculata* have been known from offshore locations in north central California including the Farallon Islands (USNM E 53137) and Cordell Bank (CASIZ 184877), since 1962 and 1979, respectively. Two specimens of *O. spiculata* were collected from San Francisco Bay in November 2001 (CASIZ 168341) and April 2002 (CASIZ 161795). On 8 November 2014, ES and JLS photographed one *O. spiculata* at Pinnacle Gulch. During 2015–2017, ES and JLS subsequently recorded a total of six other individuals in the low intertidal zone at Pinnacle Gulch, Horseshoe Cove (Bodega Marine Reserve), and Salt Point State Park. Others observers reported additional intertidal records of this species during this same period at several other sites in northern California (iNaturalist records). Most notably, single specimens of *O. spiculata* were recorded by M. Agarwal (3 July 2015, iNaturalist 1727604), M. Kelly (4 July 2015; Fig. S44) and A. Young (21 May 2016, iNaturalist 3261010) at Patrick's Point State Park, establishing the northern range record for benthic individuals of this species. Although benthic *O. spiculata* were not reported north of California, larvae were transported farther north during the heatwaves. During December 2014–January 2015, and December 2015, Richard B. Emlet documented five larvae or freshly metamorphosed juveniles of *O. spiculata* in plankton samples collected at approximately 2 m depth from just inside the entrance of Coos Bay, Oregon. These larvae/juveniles were identified via COI or 16S rRNA sequence data (R.B. Emlet, personal communication to ES). Following the end of the marine heatwaves, some *O. spiculata* persisted in the Bodega Bay region. For example, we recorded three individuals in Horseshoe Cove (Bodega Marine Reserve) during July and August 2018 (ES and JLS, personal observations). The possibility that *O. spiculata* has a disjunct distribution with extant populations in British Columbia, Canada, requires further investigation. Lambert and Austin (2007) reported that several juvenile *O. spiculata* were collected from Victoria, British Columbia, in 2001 (Valerie MacDonald, personal communication to ES). These specimens were collected from the subtidal zone, approximately 200 m from the Macaulay Waste Water Treatment Plant outfall (Valerie MacDonald, personal communication to ES). Although we were unable to locate specimens or photographs to confirm this record, the species identification was made by the late Dr. William C. Austin, who was an authority on ophiuroids. Surveys conducted around the outfall since 2001 have not documented *O. spiculata* (Tara MacDonald, personal communication to ES). Three additional brittle star specimens collected from British Columbia and deposited in the Royal British Columbia Museum were identified as *O. spiculata*. However, two of these specimens were misidentified (RBCM 003-009-04; RBCM 014-00415-002), and the third (RBCM 994-00033-21) is too small to be identified definitively (P. Lambert, personal communication to ES).

45. *Pachythyone rubra* (Red Sea Cucumber). This low intertidal to subtidal sea cucumber is usually considered a narrowly distributed endemic species with a published geographic range of Los Angeles to Monterey Bay (Morris et al. 1980, Bergen 1996, Ekert 2007, Lambert 2007). However, records from museum collections suggest a somewhat broader geographic

distribution. A subtidal specimen of *P. rubra* was dredged from Bahia de Ballenas, Baja California, Mexico, in May 1888 (USNM E 2354), and another subtidal specimen was collected just north of Isla Todos Santos, Baja California, Mexico, in April 1998 (Carpizo-Ituarte and Ramos Rivera 2017). At the northern end of its range, a brooding individual was collected from the intertidal zone at Pinnacle Gulch by James Rutherford in July 1976 (Bodega Marine Laboratory synoptic collection). *Pachythyone rubra* was also recorded in intertidal surveys at Año Nuevo Cove during 1971–1973, and at Año Nuevo Cove and Pigeon Point during 1996–1997 (Zabin et al. 2013). On 30 December 2017, ES collected a single *P. rubra* from the low intertidal zone at Bodega Marine Reserve, representing the new northern record for this species (Fig. S45; CASIZ 227281). Species identification was confirmed by ES by examination of skin and tube foot ossicles (Bergen 1996).

46. *Panulirus interruptus* (Spiny Lobster). *Panulirus interruptus* lives in subtidal rocky reef habitats and is rarely found north of Point Conception (Ricketts and Calvin 1968). Researchers with extensive diving experience in Monterey Bay report never having seen *P. interruptus* in that region (Steve I. Lonhart and James M. Watanabe, personal communication to ES). One juvenile *P. interruptus* (carapace length = 44 mm) was collected from Monterey Bay, sometime prior to December 1895 (USNM 19078), based on the USNM catalog number (Martha Nizinski, personal communication to ES). Although the northern range limit for this species is often reported as Monterey Bay (Duffy 1973), an adult *P. interruptus* (carapace length = 120 mm) was collected from San Francisco Bay in March 1940 (Fig. S46A; USNM 104212). More recently, in April 2015, a small juvenile *P. interruptus* was collected from Monterey Bay (CASIZ 220945). In August 2016, a juvenile *P. interruptus* was collected in Oyster Point Marina, San Francisco Bay (CASIZ 219743). Seventeen *P. interruptus* were also reported from Crissy Field in San Francisco between September 2013 and September 2017 based on molts and/or remains of dead lobsters (iNaturalist records). R.F. Johnson collected three spiny lobsters washed ashore at Duxbury Reef in September 2011 (CASIZ 193049) and in September 2013 (CASIZ 193000). An additional 16 *P. interruptus* were reported from Duxbury Reef based on molts/remains of lobsters recovered between September 2013 and September 2017 (iNaturalist records). Farther north, a post-larva of *P. interruptus* was recorded in Bodega Harbor in 2001 (Jensen 2014). Planktonic larvae of *P. interruptus* were collected in plankton tows off the coast of northern California, as far north as the Point Delgada region in both 2005 and 2014 (Sakuma et al. 2016; NOAA data: SWFSC Fisheries Ecology Division Mid Water Trawl Juvenile Rockfish Survey). In May 2015, *P. interruptus* larvae were collected off the coast of Fort Ross State Park (NOAA data: SWFSC Fisheries Ecology Division Mid Water Trawl Juvenile Rockfish Survey). On 19 September 2017, JLS and ES collected a fresh molt of *P. interruptus* washed ashore at Horseshoe Cove (Bodega Marine Reserve; Fig. S46B; CASIZ 224109), which represents a new northern range limit for the benthic lobster phase. Although the carapace was missing, based on the rest of the intact exoskeleton, we estimated the carapace length (CL) of this specimen to be ~55–60 mm. Juvenile *P. interruptus* are estimated to reach a CL of 55–60 mm in nature in

three years (Serfling and Ford 1975), so the size of this lobster suggests that it likely settled in 2014.

47. *Paraconcavus pacificus* (Red-striped Barnacle). *Paraconcavus pacificus* is a low intertidal to subtidal barnacle often found growing on sand dollars (*Dendraster excentricus*). Although the northern range limit has been reported as Monterey Bay (Newman and Abbott 1980), this barnacle has long occurred as far north as Ocean Beach in San Francisco, CA, with multiple museum specimens collected from this location between 1912 and 1994 (e.g., CASIZ 93558, CASIZ 98210, CASIZ 98208; see also Mooi 1997). Merrill and Hobson (1970) suggested a possible early record of *P. pacificus* in Bodega Bay, but the details are unclear. In November 2015, JLS observed a single *P. pacificus* on a sand dollar washed ashore at Abbotts Lagoon, Point Reyes National Seashore. In February 2016, *P. pacificus* was first observed on a sand dollar at Salmon Creek Beach by Alex Spooner. *Paraconcavus pacificus* was subsequently found on sand dollars at Salmon Creek Beach on six dates between March 2016–August 2017 (ES and JLS, personal observations; and personal communication to JLS). During 2016–2017, this barnacle was also observed on four dates at Dillon Beach, CA, by Stephanie Miller (personal communication to JLS), as well as at several beaches in Point Reyes National Seashore. On 14 September 2015, Doug Forsell found *P. pacificus* attached to a sand dollar washed ashore at Manchester Beach (CASIZ 224113). On 12 November 2017, Les Rohssler collected *P. pacificus* attached to two different sand dollars at MacKerricher State Park (CASIZ 224114), representing a new northern range record for this barnacle (Fig. S47). Some of these populations of *P. pacificus* appear to have persisted beyond the marine heatwaves. For example, at least seven sand dollars with *P. pacificus* were found washed ashore at Salmon Creek Beach on several dates in March 2018 (H. Bewley and P. Walrath, personal communication to JLS). In addition, on 1 February 2019, S. Miller found a live *P. pacificus* washed ashore at Dillon Beach on a clam shell.

48. *Petaloconchus montereyensis* (Monterey Tube Snail). The northern range limit of this vermetid gastropod is often published as Monterey Bay (Morris et al. 1980). However, this species was recorded as far north as Bodega Bay over 150 years ago (Stearns 1867, as *Bivonia compacta*). The earliest museum record of *P. montereyensis* from the Bodega Bay region appears to be specimens collected in June 1941 at Tomales Point by Olga Hartman (CASIZ 227847). We have noted *P. montereyensis* shells (presumably from subtidal populations) commonly washed ashore at Horseshoe Cove (Bodega Marine Reserve) since we first began working at this site in 2004 (ES and JLS, personal observations), and field notes suggest that these shells have been common at this location since at least the 1970s (J.T. Carlton, unpublished notes from July 1972). In November 2007, we recorded *P. montereyensis* in the intertidal zone as far north as Van Damme State Park, but we have been unable to locate this species at sites farther north in California or Oregon (ES and JLS, personal observations). A morphologically similar vermetid (*P. compactus*) occurs in the San Juan Islands, Washington, and Vancouver Island, Canada (Kozloff 1987). This species has sometimes been considered synonymous with *P. montereyensis* (Hadfield 1989). It is presently unknown whether there are

in fact two species of *Petaloconchus* along the west coast of North America, or whether these are populations of a single species (*P. compactus*) with a disjunct geographic distribution (Rüdiger Bieler, personal communication to ES). During 2014–2017, *P. montereyensis* underwent a striking increase in abundance in the intertidal zone at Bodega Marine Reserve. During 2004–2014, we could reliably find small clusters of these tube snails in the Bodega Marine Reserve on only two isolated rock walls in the low intertidal zone. In contrast, by winter 2017, this gastropod was widespread and locally abundant in the low intertidal zone across a large area of Bodega Marine Reserve (ES and JLS, personal observations). During this same period, we observed a similarly striking increase in the intertidal abundance of *P. montereyensis* at Salt Point State Park (Fig. S48; ES and JLS, personal observations).

49. *Petrolisthes manimaculis* (Chocolate Porcelain Crab). Prior to 1999, the northern range limit for *P. manimaculis* was recognized as Bodega Bay (Jensen 1995, Morris et al. 1980). *Petrolisthes manimaculis* was not observed in the Bodega Marine Reserve during the 1970s (Ristau et al. 1978). *Petrolisthes manimaculis* was, however, collected from the North Jetty entrance to Bodega Harbor in March 1971 (CASIZ 227848). In 1999, Wasson et al. (2002) documented a new northern range limit for this intertidal crab at Trinidad, CA. *Petrolisthes manimaculis* has been common in the low intertidal zone at Fort Ross State Park since 2006 (Jonathon H. Stillman, personal communication to ES). Starting in 2015, there was a striking increase in the abundance of small *P. manimaculis* under low intertidal cobbles in the Bodega Bay region that has persisted until our most recent survey in November 2018 (ES and JLS, personal observations). On 27 May 2017, ES and JLS collected two *P. manimaculis* under low intertidal rocks at Point St. George, CA (Fig. S49; CASIZ 224112), a new northern range record for this species.

50. *Phidiana hiltoni* (Hilton's Nudibranch). The range expansion of *Phidiana hiltoni* has been well documented. This species had a northern range limit of Monterey Bay prior to 1977, but by 1992 had expanded north to Duxbury Reef, CA (Goddard et al. 2011). Goddard et al. (2011, 2016) hypothesized that Point Reyes might be a significant barrier to further expansion of *P. hiltoni*, given its crawling, lectithotrophic larval form and the extensive sandy beaches of Point Reyes National Seashore. *Phidiana hiltoni* was recorded north of Point Reyes for the first time on 9 April 2014, when Abigail Thompson photographed an individual at Dillon Beach. A. Thompson subsequently photographed this species at Dillon Beach on 14 dates during 2015–2017 (photographs reviewed by JLS and ES). JLS and ES recorded *P. hiltoni* at Dillon Beach on 8 March 2017 (eight individuals) and 15 June 2018 (one individual). On 25 November 2015, ES and JLS recorded seven *P. hiltoni* at Pinnacle Gulch, CA (Fig. S50), a new northern range record for this species. *Phidiana hiltoni* was still present at Pinnacle Gulch on 4 December 2017 and 25–26 November 2018, with five individuals recorded on both of these occasions (ES and JLS, personal observations). Three of the specimens observed in November 2018 were small (7–13 mm length), suggesting continued recruitment of *P. hiltoni* north of Point Reyes and possible persistence of this population.

51. *Physophora hydrostatica* (Hula Skirt Siphonophore). Although this siphonophore has a global distribution (Alvariño 1971), most records of this species in the northeast Pacific occur at lower latitudes. For example, *P. hydrostatica* has been recorded off the coast of Baja California, Mexico (Alvariño 1967), and in the Gulf of California (YPM IZ 035820). The previous northern range limit for this species along the California coast was Monterey Bay, with records from 1968 (CASIZ 95481), 2006 (Gasca et al. 2015), and a few other observations during 1995–2015 (P. Pugh, personal communication to JLS). A single specimen of *P. hydrostatica* was also observed in Monterey Bay in February 2017 (iNaturalist 5117073). On four dates during 2015–2016 (26 April 2015, 4 June 2015, 19 February 2016, and 6 March 2016), as well as 16 March 2018, JLS and ES observed one to four *P. hydrostatica* washed ashore at Salmon Creek Beach. These appear to be the first records of this species north of Monterey Bay. Even when damaged in the surf zone, this species is easily recognized by its distinctive pneumatophore and enlarged, pinkish palpons (Kirkpatrick and Pugh 1984). In December 2017 and March 2018, Mike Kelly photographed single specimens of *P. hydrostatica* washed ashore at Mad River Beach in Humboldt County, CA (personal communication to ES), a new northern range record for this species in the northeast Pacific (Fig. S51). *P. hydrostatica* was also likely observed off the coast of central Oregon in July 2018 (Marco Corrales and Kelly Sutherland, personal communication to ES), suggesting that the geographic distribution of this species may have expanded north beyond California.

52. *Pleuroncodes planipes* (Pelagic Red Crab). This pelagic crab is generally restricted to waters off central and southern Baja California, Mexico (Boyd 1967). *Pleuroncodes planipes* has been observed in California rarely, but large numbers washed ashore in Monterey Bay in association with strong El Niño events in 1859, 1960 (Glynn 1961, Boyd 1967), 1973 (CASIZ 003694), and 1982–1983 (Wells et al. 1990). *Pleuroncodes planipes* was also observed offshore of Monterey Bay during the 1997–98 El Niño (Marinovic et al. 2002). In 1983, *P. planipes* was observed as far north as the Farallon Islands (Ainley 1990). The only known historical records of this species north of San Francisco, CA, are from 1985. In this year, three individuals were collected at Duxbury Reef, CA (CASIZ 173779) and *P. planipes* was also reported as far north as Fort Bragg (Pearcy et al. 1985). *Pleuroncodes planipes* was observed on beaches in Monterey Bay during many months between October 2015 and August 2017, with mass strandings reported on multiple occasions (including October 2015, May 2016, January 2017, and March 2017; Monterey Bay National Marine Sanctuary). J. Iselin reported a mass stranding on the beach at Fort Funston (San Francisco, CA) on 1 November 2016 (iNaturalist 4489090). We recorded fresh remnants of single *P. planipes* at Salmon Creek Beach on 19 October 2015 and 18 February 2016 (ES and JLS, personal observations), and collected 19 live individuals washed ashore at Salmon Creek Beach on 24 January 2017 (CASIZ 224110). Remnants of *P. planipes* were also found at Salmon Creek Beach on 4 January 2017 (ES and JLS, personal observations) and 8 January 2017 (C. Dibble, personal communication to ES). Small numbers (1–2 individuals) of *P. planipes* were subsequently observed at Van Damme State Park on 4 February 2017 (S. Gabara and K.R. Griffith), in Humboldt Bay on 5 February 2017 (B. Elvert), and in Bandon,

Oregon, on 15 February 2017 (D. Bilderback). Finally, on 1 March 2017, D. Fox photographed hundreds of *P. planipes* washed ashore at Agate Beach in Newport, Oregon (Fig. S52), representing the new northern range record for *P. planipes*.

53. *Polycera atra* (Orange-spike Polycera Nudibranch). This southern nudibranch is generally restricted to protected bays and harbors and is typically found only as far north as Point Reyes, CA (Morris et al. 1980). In association with the strong El Niño events of 1982–1983 and 1997–1998, *P. atra* was recorded as far north as Oregon and Washington (Goddard et al. 2016). JLS and ES recorded a high abundance of *P. atra* on the floating docks in Spud Point Marina, Bodega Harbor, during October 2014 and September 2015 (Fig. S53). In May 2016, *P. atra* was recorded in the harbor at Crescent City by Alison Young (iNaturalist 3219430). *Polycera atra* was also found in the Charleston boat basin, Oregon, in June 2015 (Goddard et al. 2016), and during July–August 2015, was found on Vancouver Island, British Columbia, representing a new northern range record (Goddard et al. 2018).

54. *Polycera hedgpethi* (Hedgpeth's Nudibranch). This southern nudibranch was first described from specimens collected in Tomales Bay, CA (Marcus 1964). Although its geographic distribution has been described as ranging from Baja California, Mexico, to Tomales Bay (Beeman and Williams 1980), *P. hedgpethi* was reported as far north as Bodega Harbor during a sampling period that included the strong 1972–1973 El Niño event (Standing et al. 1975). We did not observe *P. hedgpethi* in Bodega Harbor during 2004–2014 (JLS and ES, personal observations). In November 2015, K. Norris and M.A Whalen observed *P. hedgpethi* in Bodega Harbor (personal communication to JLS), and we subsequently photographed several *P. hedgpethi* at this site during November 2015 (Figure S54).

55. *Portunus xantusii* (Xantus' Swimming Crab). This low intertidal to subtidal crab is found on sand flats and in eelgrass beds. Its northern range limit was formerly reported as Santa Barbara, CA (Morris et al. 1980), but in 1999 it was found as far north as Morro Bay, CA, following the 1997–1998 El Niño event (Jensen 2014). *Portunus xantusii* was documented in San Francisco Bay for the first time in 2016, with observations reported from Alameda, San Mateo, and San Francisco in March, June, and December 2016, respectively (iNaturalist 2786987, 3384283, 4875295). On 21 August 2016, *P. xantusii* was first observed in Tomales Bay (Long Cove) by Jason Sadowski and Ted Grosholz (Fig. S55; iNaturalist 3944526; Sadowski et al. 2018). On 22 November 2016, eight *P. xantusii* (CASIZ 224111) were captured in a crab trap by Edward Parsons at Tom's Point, Tomales Bay, the northern range record for this species.

56. *Pseudoceros luteus* (White Flatworm). *Pseudoceros luteus* is a primarily southern flatworm that formerly had a published northern range limit of Monterey Bay (Morris et al. 1980). In January 2007, *P. luteus* was first observed in Bodega Harbor at Spud Point Marina by staff at Bodega Marine Laboratory (personal communication to JLS). In March 2007, this flatworm was common on the floating docks at Spud Point Marina (J.E. Byrnes, personal communication to JLS). During 2007–2014, single individuals of *P. luteus* were occasionally observed at Spud Point Marina (JLS and ES, personal observations). In September 2015, there was a striking increase in

abundance in *P. luteus* in Bodega Harbor (Fig. S56; M.A. Whalen and JLS, personal observations). This flatworm was also reported in San Francisco Bay during August–November 2015 (iNaturalist 1892041, 1956261, 2180423, 2358716), and in Tomales Bay in August 2015 (iNaturalist 1852549) and September 2016 (iNaturalist 4153031). We are aware of only one record of this flatworm north of Bodega Harbor. Lamb and Hanby (2005) reported a single *P. luteus* was collected by Doug Swanston near Tofino, British Columbia, Canada. This specimen was photographed in early May 1997 (B. Hanby, personal communication to ES), and thus its appearance in British Columbia was coincident with the strong 1997–1998 El Niño event. Although Tofino, British Columbia, is the northern range record for *P. luteus*, we are aware of no occurrences of this species in British Columbia since 1997 (D. Swanston, A. Lamb, and B. Hanby, personal communication to ES), nor has it been reported from any locations north of Bodega Harbor.

57. *Puffinus opisthomelas* (Black-vented Shearwater). *Puffinus opisthomelas* is common along the Pacific coast from Baja California, Mexico, to as far north as Point Conception in southern California. In years of warm ocean temperatures, thousands of *P. opisthomelas* disperse farther north to Monterey Bay (Keitt et al. 2000, Howell 2012). In these years, small numbers of individuals have also been observed north of Monterey Bay, on rare occasions reaching as far north as Vancouver Island, British Columbia, Canada (Keitt et al. 2000, Howell 2012). The abundance of *P. opisthomelas* in central California is considered a strong indicator of warm-water anomalies, with large population increases observed during previous El Niño events (Ainley et al. 1995, Hyrenbach and Veit 2003). From September 2014 to November 2016, there were over 75 reports of *P. opisthomelas* from Sonoma County, CA (Fig. S57; eBird 2017). Abundances in Sonoma County were particularly high during October and November 2015, with record counts numbering thousands of individuals. On 12 October 2015, JLS observed thousands of *P. opisthomelas* from the Bodega Marine Reserve. Record numbers of *P. opisthomelas* were also reported off Mendocino, Humboldt, and Del Norte counties, CA – e.g., 10,000 off Fort Bragg on 7 November 2015; 3,500 off Humboldt Bay on 7 October 2015; and 60 off the mouth of Wilson Creek on 7 October 2015 (Davis et al. 2017). Prior to 2015, *P. opisthomelas* was reported from Oregon on only five dates during 1992–2007, and all of these observations were of single birds (Oregon Bird Records Committee 2018). On 8 October 2015 and 25 October 2015, >200 Black-vented Shearwaters were recorded off the coast of Curry County, Oregon (eBird 2017).

58. *Pyrosoma atlanticum* (Pyrosome). This pyrosome (pelagic tunicate) has a global distribution in tropical and temperate oceans (Wrobel and Mills 1998). In the northeast Pacific, *P. atlanticum* was frequently documented in surveys conducted off the coast of southern California during 1951–2002 (Lavaniegos and Ohman 2003). Similarly, surveys conducted off the central California coast during 1983–2002 often recorded *P. atlanticum* in low abundance (Wells et al. 2013). At higher latitudes, *P. atlanticum* has been observed only rarely in surveys conducted >80 km off Newport, Oregon, prior to 2016 (Jennifer Fisher, personal communication to ES). Although the geographic range limit of *P. atlanticum* in the northeastern Pacific has not been defined well, this species was observed previously at least as

far north as Puget Sound, Washington, in 2003 (USNM 14572). We recorded *P. atlanticum* washed ashore at Salmon Creek Beach on at least 14 dates between December 2014 and February 2017 (Fig. S58), but did not record this species during 2004–2013 (JLS and ES, personal observations). *Pyrosoma atlanticum* was abundant on some of these dates (i.e., hundreds of colonies washed ashore on 1 km of Salmon Creek Beach). Similarly, *P. atlanticum* was observed in record abundances off the coast of central California in 2015 (Sakuma et al. 2016), and off the coasts of Oregon and Vancouver Island, British Columbia, Canada, in Summer 2017 (Sutherland et al. 2018, Brodeur et al. 2018). *Pyrosoma atlanticum* was also recorded washed ashore along the Oregon coast in December 2016 (iNaturalist 4862742) and 2017 (e.g., iNaturalist 5227221), and as far north as southeast Alaska during January–April 2017 (e.g., iNaturalist 5007884, 5118649). In general, there does not seem to be an association between periodic increases in the abundance of *P. atlanticum* in southern California and past El Niño events during 1951–2002 (Lavaniegos and Ohman 2003). In addition, there was a relatively large increase in the abundance of *P. atlanticum* in central and southern California in 2012 that was apparently unrelated to a warm-water event (Wells *et al*. 2013). Thus, although it remains unclear whether *P. atlanticum* populations in central and southern California are influenced by El Niño events, the recent appearance of large numbers of *P. atlanticum* at more northerly locations was highly anomalous and coincident with the marine heatwaves (Sutherland et al. 2018).

59. *Sula leucogaster* (Brown Booby). *Sula leucogaster* breeds on tropical and subtropical islands throughout the world, and historically was rarely observed in California (Ainley 1976). However, *S. leucogaster* has been recorded annually in California since 1990, with rare sightings off the coasts of Oregon and Washington (California Bird Records Committee 2007), and a single unconfirmed record from the Bering Sea, Alaska, in July 2000 (Tobish 2016). In California north of San Francisco, there were <10 records of *S. leucogaster* between 1998–2007 (California Bird Records Committee 2007, Tietz and McCaskie 2018), and there were only two records from Sonoma County, CA, both in 2013 (eBird 2017). In association with warm-water conditions during 2014–2015, there was a striking increase in *S. leucogaster* sightings in northern California, with >75 reports north of San Francisco extending to Del Norte County, CA (Rogers et al. 2015, Davis et al. 2016, Pandolfino et al. 2016, Rottenborn et al. 2016, Davis et al. 2017). In October 2015, as many as 30 individuals were observed at the Farallon Islands (Davis et al. 2017). During 2014–2015, there were five records of *S. leucogaster* in Sonoma County (eBird 2017). During 2014–2015, there was also an increased frequency of *S. leucogaster* records off the Pacific Northwest, with up to seven records from Oregon and at least eight records from Washington (Irons et al. 2016, Waggoner and Merrill 2017), and five records from British Columbia, Canada (Charlesworth 2016, Charlesworth 2017). Finally, four *S. leucogaster* were observed in Alaska in 2014 and 2015, with three records from coastal Southeast Alaska, and one individual observed off Amlia Island in the Aleutian Islands in August 2015 (Tobish 2016, Tobish 2017). Northern observations of *S. leucogaster* continued into 2016–2017, including a sighting of *S. leucogaster* in Tomales Bay (Fig. S59; 6 February 2016).

60. ***Synthliboramphus hypoleucus*** **(Guadalupe Murrelet).** *Synthliboramphus hypoleucus* nests on offshore islands along the Pacific coast of Baja California, Mexico. Following the breeding season, very small numbers of *S. hypoleucus* disperse to areas well offshore of central California in most years (Chesser et al. 2012). Records of *S. hypoleucus* north of central California are rare and are often associated with warm-water years (Ainley 1976, Ainley et al. 1995). *S. hypoleucus* appears to seek warmer water during the non-breeding season and will disperse as far north as temperatures allow (Ainley 1976), occasionally reaching waters off the coast of Washington (Chesser et al. 2012). Guadalupe Murrelets were observed at Bodega Canyon/Cordell Bank on four dates in 2014–2015: one on 19 September 2014 (Fig. S60); two on 22 August 2015; three on 16 September 2015; and one on 18 September 2015 (Davis et al. 2017, eBird 2017). Single Guadalupe Murrelets were recorded off the coast of Westport, Washington, on 1 August 2015 and 22 July 2017 (Waggoner and Merrill 2017, Washington Bird Records Committee 2017).

61. ***Tetraclita rubescens*** **(Pink Volcano Barnacle).** Prior to the 1980s, this intertidal barnacle was regarded as rare north of San Francisco, with single specimens collected at Shell Beach, CA, in 1957 and 1970, and two specimens collected at Fort Ross State Park, CA, no later than the early to mid-1970s (Dawson et al. 2010). In 1984, *T. rubescens* was found at Sea Ranch and Saunders Reef, CA, by J.S. Pearse (Dawson et al. 2010), and in 1995–1996 a single individual was recorded at Cape Mendocino, CA (Connolly & Roughgarden 1998). In 2007, the northern range limit of *T. rubescens* was established when a single individual was found by the Coastal Biodiversity Survey (P.T. Raimondi, UC Santa Cruz) at Burnt Hill, Oregon, although the individual was no longer present in a subsequent survey (Dawson et al. 2010). During surveys at Cape Mendocino, we recorded six *T. rubescens* in Spring 2005, three individuals in Spring 2012, and 12 individuals in Spring 2017 (ES and JLS, personal observations). In Spring 2017, JLS and ES also recorded five *T. rubescens* at Patrick's Point State Park, CA (Fig. S61B). Our surveys at Van Damme State Park (Fig. 5, S61A) and observations at other sites (ES and JLS, personal observation) indicate strong recruitment and increased abundance of *T. rubescens* in northern California during 2014–2015.

62. ***Thalia democratica*** **(Salp).** *Thalia democratica* is a salp (pelagic tunicate) found globally in tropical and warm temperate waters (Wrobel and Mills 1998). In the northeast Pacific, Berner (1957) reported that *T. democratica* was most common in waters with a mean temperature of 14–21°C. Similarly, in the Mediterranean, *T. democratica* was associated with water temperatures >15.5°C (Licandro et al. 2006). Warmer temperatures approaching 20°C appear to shorten the generation time of *T. democratica* and can lead to rapid population blooms (Licandro et al. 2006). *Thalia democratica* can be very abundant offshore along the coasts Baja California, Mexico, and southern California (Berner 1957, Blackburn 1979). In association with the 1963–1964 El Niño event, small numbers of *T. democratica* were observed as far north as Newport, Oregon, the northern range limit for this species in the northeast Pacific (Hubbard and Pearcy 1971, Lavaniegos and Ohman 2003). We recorded *T. democratica* washed ashore at Horseshoe Cove on 25 August 2014 and 14 September 2014 (Fig. S62) and on Salmon Creek Beach on 17 October 2015, but we did not observe this species during 2004–2013 (JLS and ES,

personal observations). Our observation from 25 August 2014 was a mass stranding event of thousands of zooids.

63. *Thetys vagina* (Salp). This large salp (pelagic tunicate) is broadly distributed in warmer waters of the Atlantic, Pacific, and Indian Oceans (McAlice 1986, Wrobel and Mills 1998). In California, interannual variation in the abundance of *T. vagina* does not appear to be strongly correlated with El Niños or other warm-water events (Wells et al. 2013, Sakuma et al. 2016). However, records of *T. vagina* at sites north of California are uncommon and appear to be associated with El Niño events. For example, *T. vagina* was observed off the coast of Oregon in association with the El Niño events of 1963–1964 (Hubbard and Pearcy 1971), 1982–1983 (Pearcy et al. 1985), and 1997–1998 (UAM 11898). Prior to 2014, the northern range record for *Thetys* in the northeast Pacific appears to be a specimen collected at Grays Canyon (off the Washington coast) in 1888 (USNM 6703). During 2014–2017, we observed small numbers (up to eight individuals) of *T. vagina* washed ashore at Salmon Creek Beach on eight dates, but we only observed this species a few times during 2004–2013 (JLS and ES, personal observations). *Thetys vagina* was also found on Mad River Beach in northern California during September–November 2017 (Mike Kelly, personal communication to ES). Surveys conducted off the Oregon coast in June 2016 recorded increased abundances of *T. vagina* (McClatchie et al. 2016). During 2015–2017, *T. vagina* was also observed washed ashore on beaches in Oregon (e.g., iNaturalist 8093139), and was recorded as far north as British Columbia, Canada, on multiple occasions. Specifically, Jackie Hildering and Natasha Dickison observed *T. vagina* in the shallow subtidal zone on 15 February 2015 while diving near Port Hardy, British Columbia (Fig. S63A; J. Hildering, personal communication to ES). Additional observations of *Thetys* in the Port Hardy area were recorded in November 2016 and March 2017 (J. Hildering, personal communication to ES). On 13 June 2017, Brian Starzomski found a single specimen of *T. vagina* washed ashore at Calvert Island, British Columbia (Fig. S63B; iNaturalist 7662368; B. Starzomski, personal communication to ES). To our knowledge, this represents the new northern range record for this species.

64. *Thylacodes squamigerus* (Scaled Tube Snail). This vermetid gastropod occurs in the low intertidal zone of rocky shores and has a published northern range limit of Monterey Bay (Morris et al. 1980). *Thylacodes squamigerus* was collected from Pacific Grove as early as 1910 (CASIZ 119499), but was apparently uncommon in the Monterey Bay region until sometime after 1973. *Thylacodes squamigerus* was absent from transect surveys conducted at Hopkins Marine Station (Pacific Grove) in 1931–1933 (Sagarin et al. 1999). Similarly, *T. squamigerus* was absent from surveys conducted at 10 sites in northern Monterey Bay during 1971–1973 (Sagarin et al. 1999, Zabin et al. 2013), and Ricketts and Calvin (1968) reported this species as "only occasional in the Monterey Bay region." In contrast, *T. squamigerus* was common at many sites in Monterey Bay by the mid-1990s (Sagarin et al. 1999, Zabin et al. 2013). This species was also found as far north as Half Moon Bay in 1994 (Sagarin et al. 1999). A single specimen of *T. squamigerus* was found in Bodega Marine Reserve by UC Santa Cruz researchers in 2001, and ES and JLS recorded six individuals at this site during 2006–2013. On 24 and 25

February 2017, ES and JLS recorded four *T. squamigerus* at Salt Point State Park (Fig. S64), a new northern range record for this species.

65. *Triopha maculata* (Spotted Triopha Nudibranch). *Triopha maculata* is a primarily southern nudibranch that can be very common in Monterey Bay tidepools (Nybakken 1978). Its northern range limit was reported previously as Bodega Bay (Ferreira 1977), although MacFarland (1966) reported that *T. maculata* ranged as far north as Crescent City, and Jaeckle (1981) reported this species at Patrick's Point State Park. Millen (1983) reported a new northern range record for *T. maculata* at Bamfield, Vancouver Island, British Columbia, Canada, presumably in association with the 1982–1983 El Niño event. During September and October 2015, 19 specimens of *T. maculata* were observed by Jackie Hildering in the shallow subtidal zone at Bear Cove, Port Hardy, Vancouver Island, British Columbia (Fig. S65), a new northern range record for this species (Goddard et al. 2018).

66. *Tursiops truncatus* (Common Bottlenose Dolphin). *Tursiops truncatus* occurs worldwide in tropical, subtropical, and warm-temperate waters (Allen et al. 2011). In the northeast Pacific, a relatively small population of *T. truncatus* (~450 dolphins) inhabits coastal waters, whereas a distinct, larger population of ~3,500 occurs offshore (Halpin et al. 2018). The coastal population of *T. truncatus* was historically known primarily from south of Point Conception (Allen et al. 2011). However, some *T. truncatus* extended their coastal range north to Monterey Bay during the 1982–1983 El Niño event (Wells et al. 1990). Some *T. truncatus* remained in central California and subsequently moved north to San Francisco Bay, where they have been regularly observed and monitored since 2010 (Bill Keener, Golden Gate Cetacean Research, personal communication to JLS). One of the first sightings of coastal *T. truncatus* north of San Francisco was off Doran Beach in July 2012 (Darris Nelson, personal communication to JLS). Since September 2014, coastal observations of *T. truncatus* north of San Francisco have become more common. From 2015–2017, *T. truncatus* were reported at sites between Dillon Beach and the Russian River (Fig. S66) on a frequent basis (>25 times; JLS and ES, personal observations; and reports to JLS). During this period, the farthest north coastal observation of *T. truncatus* was from Little River, CA, in April 2016 (Bill Keener, personal communication to JLS), a new northern range record for the coastal ecotype. The offshore population had been observed previously as far north as 41°N. However, in July 2017, *T. truncatus* was observed 180 km off the coast of British Columbia, Canada (Halpin et al. 2018). This is the first confirmed record of *T. truncatus* in Canadian waters and represents a new geographic range record for this species in the northeast Pacific (Halpin et al. 2018). However, because our study is focused on the distribution of coastal biota, we focus here on the range extension of the coastal ecotype of *T. truncatus* (Table 1).

67. *Velella velella* (By-the-wind Sailor). *Velella velella* is a pelagic, colonial hydrozoan that occurs globally in warm to warm-temperate waters (Wrobel and Mills 1998). Although small numbers are washed ashore on beaches in northern California during most years, mass strandings on northern beaches are often associated with strong El Niño events. For example, during the major 1925–1926 El Niño, *V. velella* were abundant as far north as British Columbia,

Canada (Hubbs 1948). Similarly, during the strong 1957–1958 El Niño, an increased abundance of *V. velella* was noted from San Francisco to Alaska (Radovich 1961). Widespread sightings of *V. velella* also occurred in British Columbia during the 1982–1983 El Niño (Pearcy and Schoener 1987). Hubbs (1948) hypothesized that mass strandings of *V. velella* during El Niño events likely arise from onshore transport of animals from warmer, offshore waters. During 2014 and 2015, we observed mass strandings of *V. velella* on Salmon Creek Beach on multiple dates (Fig. S67; JLS and ES, personal observations). *Velella velella* were also observed during this period in large numbers along much of the U.S. Pacific coast as far north as Sitka, Alaska (e.g., iNaturalist 5102488, 9196515).

B. ACKNOWLEDGEMENTS:

For their contributions to our investigations of recent and historical observations of coastal biota, we are especially grateful to James T. Carlton, Christina Piotrowski, Molly Engelbrecht, Jackie Hildering, Mike Kelly, and Steve Morey.

We thank the following museums, databases, curators, and staff scientists for access to their collections, records, and data:

- California Academy of Sciences, Invertebrate Zoology and Geology, San Francisco, CA (Christina Piotrowski, Elizabeth Kools, Johanna Loacker)
- Smithsonian Museum of Natural History, Washington, D.C. (Christopher Meyer, Martha Nizinski)
- Natural History Museum of Los Angeles County Museum, Los Angeles, CA (Lindsey Groves, Rick Feeney)
- Royal British Columbia Museum, Victoria, British Columbia, Canada (Hugh McIntosh, Heidi Gartner, Gavin Hanke, Henry Choong)
- Academy of Natural Sciences of Drexel University, Philadelphia, PA (Paul Callomon, Ellen Wildner)
- Scripps Institution of Oceanography Benthic Invertebrate Collection, La Jolla, CA (Charlotte Seid)
- Oregon State University Ichthyology Collection (Brian Sidlauskas)
- Burke Museum of Natural History and Culture, Seattle, WA (Melissa Frey)
- University of Alaska Museum of the North, Fairbanks, AK (Andrés Lopez)
- iNaturalist (www.inaturalist.org), and its many contributors
- Global Biodiversity Information Facility (GBIF); www.gbif.org

• Point Blue Conservation Science, ACCESS cruises (Jaime Jahncke)

• National Oceanic and Atmospheric Administration (NOAA), National Marine Fisheries Service, Southwest Fisheries Science Center (Keith Sakuma)

For sharing their observations, photographs, specimens, and insights, we thank: Robin Agarwal, Hollis Bewley, Dave and Diane Bilderback, Paul Bourdeau, Caren Braby, Josh Chow, Todd Cliff, Peter G. Connors, Marco Corrales, Colby Davidson, Catherine V. Davis, Jared Davis, Connor Dibble, Brad Elvert, Phil Fenberg, Jennifer Fisher, Doug Forsell, David Fox, Torre Flagor, Liz Francis, Scott Gabara, Rani Gaddam, Maya George, Kaylee Griffith, Ted Grosholz, Grace Ha, Steven Haddock, Roger Harshaw, Jan Hodder, Steve N.G. Howell, June Jacobson, Norma Jellison, Greg Jensen, Kathy Johnson, Rebecca F. Johnson, Bill Keener, Peter Konstantinidis, Armand Kuris, Chris Kwan, Andy Lamb, Philip Lambert, Russ Lewis, Dave Lohse, Gary R. McDonald, Natalie McNear, Baldo Marinovic, Doug Mason, Peter Mieras, Kathy Ann Miller, Stephanie Miller, Claudia Mills, William A. Newman, Kelly Norris, Tenaya Norris, Phil Nosler, Brent and Edwards Parsons, John and Vicki Pearse, Christopher Pirrone, Bob Pitman, Maxine (Hao Hao) Pontius, Charlie Plybon, Philip Pugh, Lewis Reed, Emily Rivest, Les Rohssler, Jerry and Lynn Rudy, Jason Sadowski, Rafe Sagarin, Linda Schroeder, Roger Seapy, Debi Shearwater, Alex Spooner, Brian Starzomski, David Stein, Jonathon Stillman, Seth Strumwasser, Kelly Sutherland, Chad Tamis, The Marine Mammal Center, Abigail Thompson, Nancy Treneman, Sarit Truskey, Robert Van Syoc, Carol Vines, Carl and Cameron Vogler, Phil Warren, Kerstin Wasson, James Watanabe, Matt Whalen, Alan Wight, Marjorie Wonham, Alison Young.

C. <u>REFERENCES CITED</u>:

Abbott, I. A. & Hollenberg, G. J. (1976) *Marine Algae of California*. (Stanford University Press, Stanford, CA).

Ainley, D. G. (1976) The occurrence of seabirds in the coastal region of California. *Western Birds* **7**, 33–68.

Ainley, D. G. (1990) Seasonal and annual patterns in the marine environment near the Farallones [Ainley, D. G. & Boekelheide, R. J. (eds.)] *Seabirds of the Farallon Islands: Ecology, Dynamics, and Structure of an Upwelling-System Community.* Pp. 23–50 (Stanford University Press , Stanford, CA).

Ainley, D. G. (2008) Black Storm-Petrel (*Oceanodroma melania*) [Shuford, W. D. & Gardali, T. (eds.)] *California Bird Species of Special Concern: A Ranked Assessment of Species, Subspecies, and Distinct Populations of Birds of Immediate Conservation Concern in California*, *Studies of Western Birds 1.* Pp. 125–129 (Western Field Ornithologists and California Department of Fish and Wildlife).

Ainley, D. G., Veit, R. L., Allen, S. G., Spear, L. B. & Pyle, P. (1995) Variations in marine bird communities of the California current, 1986–1994. *Calif. Coop. Ocean. Fish. Invest. Rep.* **36,** 72–77.

Allen, S. G., Mortenson, J. & Webb, S. (2011) *Field Guide to Marine Mammals of the Pacific Coast*. (University of California Press).

Alvariño, A. (1967) Bathymetric distribution of chaetognatha, siphonophorae, medusae, and ctenophorae off San Diego, California. *Pacific Science* **21,** 474–485.

Alvariño, A. (1971) Siphonophores of the Pacific, with a review of the world distribution. *University of California Technical Series* **16,** 1–432.

Alvariño, A. (1980) The relation between the distribution of zooplankton predators and anchovy larvae. *Calif. Coop. Ocean Fish. Invest. Rep.* **21,** 150–160.

Aurioles-Gamboa, D. (2015) *Arctocephalus townsendi*. *The IUCN Red List of Threatened Species 2015*: e.T2061A45224420. http://dx.doi.org/10.2305/IUCN.UK.2015-2.RLTS.T2061A45224420.en

Banner, A. H. & McKernan, D. L. (1943) A record of *Emerita analoga* from the Washington coast. *Science* **97,** 119.

Barlow, J. (2016) Cetacean abundance in the California Current estimated from ship-based line-transect surveys in 1991-2014. Administrative Report LJ-16-01. (NOAA Southwest Fisheries Science Center).

Bé, A. W. H. & Gilmer, R. W. (1977) A zoogeographic and taxonomic review of euthecosomatous Pteropoda. [Ramsey, A. T. S. (ed.)]. *Oceanic Micropaleontology, Vol. 1.* Pp. 733–808 (Academic Press).

Beeman, R. D. & Williams, G. C. (1980) Opisthobranchia and Pulmonata. [Morris, R. H., Abbott, D. P., & Haderlie, E. C. (eds.)]. *Intertidal Invertebrates of California.* Pp. 308–354 (Stanford University Press).

Bergen, M. (1996) Class Holothuroidea. [Blake, J. A., Scott, P. H., & Lissner, A. (eds.)]. *Taxonomic Atlas of the Benthic Fauna of the Santa Maria Basin and the Western Santa Barbara Channel Vol. 14, Miscellaneous Taxa.* Pp. 195–250 (Santa Barbara Museum of Natural History).

Bernard, F. R. (1970) A distributional checklist of the marine molluscs of British Columbia: based on faunistic surveys since 1950. *Syesis* **3,** 75–94.

Berner, L. (1957) *Studies on the Thaliacea of the temperate northeast Pacific Ocean.* PhD thesis, University of California, Los Angeles.

Berry, S. S. (1958) Double-trouble in violet snails. *American Malacological Union Annual Report for 1957.* **Bulletin 24,** 27 (abstract).

Bertsch, H., Gosliner, T., Wharton, R. & Williams, G. (1972) Natural history and occurrence of opisthobranch gastropods from the open coast of San Mateo Co., California. *Veliger* **14,** 302–314.

Beu, A. G. (2017) Evolution of *Janthina* and *Recluzia* (Mollusca: Gastropoda: Epitoniidae). *Records of the Australian Museum* **69,** 119–222.

Blackburn, M. (1979) Thaliacea of the California Current Region: Relations to temperature, chlorophyll, currents, and upwelling. *Calif. Coop. Ocean Fish. Invest. Rep.* **XX,** 184–214.

Boyd, C. M. (1967) The benthic and pelagic habitats of the red crab, *Pleuroncodes planipes*. *Pacific Science* **21,** 394–403.

Brodeur, R. D. *et al.* (2006) Anomalous pelagic nekton abundance, distribution, and apparent recruitment in the northern California Current in 2004 and 2005. *Geophys. Res. Lett.* 33, L22S08; doi:10.1029/2006GL026614

Brodeur, R. *et al.* (2018) An unusual gelatinous plankton event in the NE Pacific: The Great Pyrosome Bloom of 2017. *PICES Press* **26,** 22–28.

Burch, J. Q. (1945) Family Janthinidae. *Minutes of the Conchological Club of Southern California* **63,** 3–5.

California Rare Birds Committee. (2007) *Rare Birds of California* (Western Field Ornithologists).

Carlton, J. T., Newman, W. A. & Pitombo, F. B. (2011) Barnacle invasions: Introduced, cryptogenic, and range expanding cirripedia of North and South America. [Galil, B. S., Clark, P. F., and Carlton, J. T. (eds.)]. *In the Wrong Place – Alien Marine Crustaceans: Distribution, Biology and Impacts*. Invading Nature-Springer Series in Invasion Ecology Vol. 6.) Pp. 159–213 (Springer).

Carpizo-Ituarte, E. D. J. & Rivera, P. R. (2017) Biodiversidad de macroinvertebrados bénticos de la región marina Tijuana-Ensenada Baja California, México. Version 1.3 (Comisión nacional para el conocimiento y uso de la biodiversidad).

Carretta, J. V. *et al.* (2017) Guadalupe Fur Seal (*Arctocephalus townsendi*), in *U.S. Pacific marine mammal stock assessments: 2016,* NOAA-TM-NMFS-SWFSC-577 (U.S. Department of Commerce).

Cavole, L. M. *et al.* (2016) Biological impacts of the 2013-2015 warm-water anomaly in the Northeast Pacific. *Oceanography* **29,** 273–285.

Chace, E.P. (1948) *Minutes of the Conchological Club of Southern California* **84,** 2.

Charlesworth, C. (2016) The Regional Reports: British Columbia. *North American Birds* **69,** 141–142.

Charlesworth, C. (2017) The Regional Reports: British Columbia. *North American Birds* **70,** 104–108.

Cheng, L. & Lewin, R. A. (1976) Goose barnacles (Cirripedia-Thoracica) on flotsam beached at La Jolla, California. *Fish. Bull.* **74,** 212–217.

Chesser, R. T. *et al.* (2012) Fifty-third Supplement to the American Ornithologists' Union Checklist of North American Birds. *Auk* **129,** 573–588.

Connolly, S. R. & Roughgarden, J. (1998) A range extension for the volcano barnacle, *Tetraclita rubescens*. *Calif. Fish Game* **84,** 182–183.

Cornwall, I. E. (1955) *Canadian Pacific Fauna. 10. Arthropoda, 10e: Cirripedia.* (Fisheries Research Board Ottawa).

Costello, D. P. (1938) Notes on the breeding habits of the nudibranchs of Monterey Bay and vicinity. *J. Morphol.* **63,** 319–343.

Davis, J. N., Pandolfino, E., Rottenborn, S. C. & Rogers, M. M. (2016) The Regional Reports: Northern California. *North American Birds* **69,** 148–152.

Davis, J. N., Pandolfino, E., Rottenborn, S. C. & Rogers, M. M. (2017) The Regional Reports: Northern California. *North American Birds* **70,** 112–117.

Dawson, M. N., Grosberg, R. K., Stuart, Y. E. & Sanford, E. (2010) Population genetic analysis of a recent range expansion: mechanisms regulating the poleward range limit in the volcano barnacle *Tetraclita rubescens*. *Mol. Ecol.* **19,** 1585–1605.

Duffy, J. M. (1973) *Status of the Spiny Lobster.* Marine Resources Technical Report No. 10. (California Department of Fish and Game).

eBird. (2017) eBird Basic Dataset. Version: EBD_relNov-2017. (Cornell Lab of Ornithology, Ithaca, New York).

Eckert, G. L. (2007) Spatial patchiness in the sea cucumber *Pachythyone rubra* in the California Channel Islands. *J. Exp. Mar. Biol. Ecol.* **348,** 121–132.

Eschmeyer, W. N. & Herald, E. S. (1983) *A Field Guide to Pacific Coast Fishes of North America from the Gulf of Alaska to Baja California*. (Houghton Mifflin).

Fenberg, P. B. & Rivadeneira, M. M. (2011) Range limits and geographic patterns of abundance of the rocky intertidal owl limpet, *Lottia gigantea*. *J. Biogeogr.* **38,** 2286–2298.

Ferreira, A .J. (1977) A review of the genus *Triopha* (Mollusca: Nudibranchia). *Veliger* **19,** 387–402.

Flagor, T. E. & Bourdeau, P. E. (2018) First record of the predatory snail *Acanthinucella spirata* (Blainville, 1832) north of its known range. *Marine Biodiversity Records* **11,** 19; doi:10.1186/s41200-018-0156-z

Ford, J. K. B. (2005) First records of Long-beaked Common Dolphins, *Delphinus capensis*, in Canadian waters. *Can. Field-Nat.* **119,** 110–113.

Francis, L. (1979) Contrast between solitary and clonal lifestyles in the sea anemone *Anthopleura elegantissima*. *Am. Zool.* **19,** 669–681.

García-Reyes, M. & Sydeman, W. J. (2017) California Multivariate Ocean Climate Indicator (MOCI) and marine ecosystem dynamics. *Ecol. Indic.* **72,** 521–529.

Gasca, R., Hoover, R. & Haddock, S. H. D. (2015) New symbiotic associations of hyperiid amphipods (Peracarida) with gelatinous zooplankton in deep waters off California. *J. Mar. Biol. Assoc. U.K.* **95,** 503–511.

Glynn, P. W. (1961) The first recorded mass stranding of pelagic red crabs, *Pleuroncodes planipes*, at Monterey Bay, California, since 1859, with notes on their biology. *Calif. Fish Game* **47,** 97–101.

Goddard, J. H. R. (1984) The opisthobranchs of Cape Arago, Oregon, with notes on their biology and a summary of benthic opisthobranchs known from Oregon. *Veliger* **27,** 143–163.

Goddard, J. H. R. (1997) Range extensions of eight northeastern Pacific nudibranchs. *Opisthobranch Newsletter* **23,** 13.

Goddard, J. H. R. (2004) Developmental mode in benthic opisthobranch molluscs from the northeast Pacific Ocean: feeding in a sea of plenty. *Canadian Journal of Zoology* **82,** 1954–1968.

Goddard, J. H. R., Gosliner, T. M. & Pearse, J. S. (2011) Impacts associated with the recent range shift of the aeolid nudibranch *Phidiana hiltoni* (Mollusca, Opisthobranchia) in California. *Mar. Biol.* **158,** 1095–1109.

Goddard, J. H. R. *et al.* (2016) Nudibranch range shifts associated with the 2014 warm anomaly in the northeast Pacific. *Bulletin of the Southern California Academy of Sciences* **115,** 15–40.

Goddard, J. H. R. *et al.* (2018) Heterobranch sea slug range shifts in the northeast Pacific Ocean associated with the 2015–16 El Niño. *Proceedings of the California Academy of Sciences Series 4* **65,** 107–131.

Gosliner, T. M. & Williams, G. C. (1970) The opisthobranch mollusks of Marin County, California. *Veliger* **13,** 175–180.

Gould, P., Ostrom, P. & Walker, W. (2000) Foods, trophic relationships, and migration of Sooty and Short-tailed Shearwaters associated with squid and large-mesh driftnet fisheries in the North Pacific Ocean. *Waterbirds* **23,** 165–186.

Haddock, S. H. D. & Case, J. F. (1999) Bioluminescence spectra of shallow and deep-sea gelatinous zooplankton: ctenophores, medusae and siphonophores. *Mar. Biol.* **133,** 571–582.

Hadfield, M. G. (1989) Latitudinal effects on juvenile size and fecundity in *Petaloconchus* (Gastropoda). *Bull. Mar. Sci.* **45,** 369–376.

Hadfield, M. G. & Switzer-Dunlap, M. (1984) Opisthobranchs. [Tompa, A. S., Verdonk, N. H. & Van Den Biggelaar, J. A. M. (eds.)]. *The Mollusca. Vol. 7. Reproduction.* Pp. 209–350 (Academic Press).

Halpin, L. R., Towers, J. R. & Ford, J. K. B. (2018) First record of common bottlenose dolphin (*Tursiops truncatus*) in Canadian Pacific waters. *Marine Biodiversity Records* **11,** 3; doi:10.1186/s41200-018-0138-1

Harbison, G. R., Madin, L. P. & Swanberg, N. R. (1978) On the natural history and distribution of oceanic ctenophores. *Deep-Sea Research* **25,** 233–256.

Hellberg, M. E., Balch, D. P. & Roy, K. (2001) Climate-driven range expansion and morphological evolution in a marine gastropod. *Science* **292,** 1707–1710.

Hobday, A. J. *et al*. (2016) A hierarchical approach to defining marine heatwaves. *Prog. Oceanogr.* **141,** 227–238.

Hodge, R. P. & Wing, B. L. (2000) Occurrences of marine turtles in Alaska Waters: 1960–1998. *Herpetological Review* **31,** 148–151.

Hoover, C., Lindsay, T., Goddard, J. H. R. & Valdes, A. (2015) Seeing double: pseudocryptic diversity in the *Doriopsilla albopunctata–Doriopsilla gemela* species complex of the northeastern Pacific. *Zool. Scr.* **44,** 612–631.

Howell, S. N. G. (2012) *Petrels, Albatrosses, and Storm-Petrels of North America: A Photographic Guide.* (Princeton University Press).

Hubbard, L. T. & Pearcy, W. G. (1971) Geographic distribution and relative abundance of Salpidae off the Oregon coast. *Journal of the Fisheries Research Board of Canada* **28,** 1831–1836.

Hubbs, C. L. (1916) Notes on the marine fishes of California. *University of California Publications in Zoology* **16,** 153–169.

Hubbs, C. L. (1948) Changes in the fish fauna of western North America correlated with changes in temperature. *Journal of Marine Research* **7,** 459–482.

Hyrenbach, K. D. & Veit, R. R. (2003) Ocean warming and seabird communities of the southern California Current System (1987-98): Response at multiple temporal scales. *Deep-Sea Res. Part II-Top. Stud. Oceanogr.* **50,** 2537–2565.

Irons, D., Waggoner, B. & Merrill, R. (2016) The Regional Reports: Oregon and Washington. *North American Birds* **69,** 143–148.

Jaeckle, W. B. (1981) Range extensions of several opisthobranchs from Humboldt County, California. *Opisthobranch Newsletter* **13,** 23–24.

Jaeckle, W. B. (1983) Additions to the opisthobranch mollusk fauna of Marin County, California, with notes on the biology of certain species. *Veliger* **26,** 93–95.

Jaeckle, W. B. (1984) The opisthobranch mollusks of Humboldt County, California. *Veliger* **26,** 207–213.

Jensen, G. C. (1995) *Pacific Coast Crabs and Shrimps*. (Sea Challengers).

Jensen, G. C. (2014) *Crabs and Shrimps of the Pacific Coast: A Guide to Shallow-water Decapods from Southeastern Alaska to the Mexican Border*. (MolaMarine).

Johnson, L. (2016) “Endangered green sea turtle with hypothermia rescued from B.C. beach.” https://www.cbc.ca/news/canada/british-columbia/endangered-sea-turtle-pacific-rim-national-park-1.3419061

Jones, C. (2014) “Fishermen find rare green sea turtle outside Golden Gate.” https://blog.sfgate.com/stew/2014/09/12/fishermen-find-rare-green-sea-turtle-outside-golden-gate/

Kammerichs-Berke, D. (2018) Northernmost record of the Wedge-rumped Storm-Petrel (*Oceanodroma tethys*). *Western Birds* **49,** 74–76.

Keitt, B. S., Tershy, B. R. & Croll, D. W. (2000) Black-vented Shearwater *(Puffinus opisthomelas)* in *The Birds of North America* Vol. 521.

Kirkpatrick, P. A. & Pugh, P. R. (1984) *Siphonophores and Velellids, Synopses of the British Fauna (new series)*. (Linnean Society).

Knudsen, J. W. (1963) Notes on the barnacle *Lepas fascicularis* found attached to the jellyfish *Velella*. *Bulletin of the Southern California Academy of Sciences* **62,** 130–131.

Kozloff, E. N. & Price, L. H. (1987) *Marine Invertebrates of the Pacific Northwest*. (University of Washington Press).

Lamb, A. & Hanby, B. (2005) *Marine Life of the Pacific Northwest*. (Harbour Publishing).

Lambert, P. (2007) Holothuroidea. [Carlton, J. T. (ed.)]. *The Light and Smith Manual: Intertidal Invertebrates from Central California to Oregon. Fourth Edition*. Pp. 941–948 (University of California Press).

Lambert, P. & Austin, W. C. (2007) *Brittle Stars, Sea Urchins and Feather Stars of British Columbia, Southeast Alaska and Puget Sound.* (Royal British Columbia Museum).

Lance, J. R. (1966) New distributions of some northeastern Pacific Opisthobranchiata (Mollusca: Gastropoda) with descriptions of two new species. *Veliger* **9,** 69–81.

Lavaniegos, B. E. & Ohman, M. D. (2003) Long-term changes in pelagic tunicates of the California Current. *Deep-Sea Res. Part II-Top. Stud. Oceanogr.* **50,** 2473–2498.

Lea, R. N. & Rosenblatt, R. H. (2000) Observations on fishes associated with the 1997–98 El Niño off California. *Calif. Coop. Ocean. Fish. Invest. Rep.* **41,** 117–129.

Licandro, P., Ibanez, F. & Etienne, M. (2006) Long-term fluctuations (1974–1999) of the salps *Thalia democratica* and *Salpa fusiformis* in the northwestern Mediterranean Sea: Relationships with hydroclimatic variability. *Limnol. Oceanogr.* **51,** 1832–1848.

Love, M. S., Mecklenburg, C. W., Mecklenburg, T. A. & Thorsteinson, L. K. (2005) Resource inventory of marine and estuarine fishes of the West Coast and Alaska: A checklist of North Pacific and Arctic Ocean species from Baja California to the Alaska-Yukon Border. OCS Study MMS 2005-030 and USGS/NBII 2005-001. (U.S. Department of the Interior, U.S. Geological Survey, Biological Resources Division, Seattle, Washington).

Lowe, C. & Sternfeld, M. (2007) Draft 2007 Alaska Marine Mammal Stranding Summary. (National Marine Fisheries Service, Alaska Region, Protected Resources). https://www.fisheries.noaa.gov/webdam/download/69425130

MacFarland, F. M. (1966) Studies of opisthobranchiate mollusks of the Pacific Coast of North America. *California Academy of Science Memoirs* **6,** 1–546.

Mapstone, G. M. (2009) *Siphonophora (Cnidaria: Hydrozoa) of Canadian Pacific Waters*. (NRC Research Press).

Marcus, E. R. (1964) A new species of *Polycera* (Nudibranchia) from California. *Nautilus* **77,** 128–131.

Marinovic, B. B., Croll, D. A., Gong, N., Benson, S. R. & Chavez, F. P. (2002) Effects of the 1997–1999 El Niño and La Niña events on zooplankton abundance and euphausiid community composition within the Monterey Bay coastal upwelling system. *Prog. Oceanogr.* **54,** 265–277.

McAlice, B. J. (1986) Occurrence of *Thetys vagina* Tilesius (Tunicata, Thaliacea) on the coast of Maine. *Bull. Mar. Sci.* **39,** 717–718.

McAlpine, D. F., Orchard, S. A., Sendall, K. A. & Palm, R. (2004) Status of marine turtles in British Columbia waters: A reassessment. *Can. Field-Nat.* **118,** 72–76.

McClatchie, S. *et al.* (2016) State of the California Current 2015–16: Comparisons with the 1997–98 El Niño. *Calif. Coop. Ocean. Fish. Invest. Rep.* **57,** 5–61.

McFadden, C. S., Grosberg, R. K., Cameron, B. B., Karlton, D. P. & Secord, D. (1997) Genetic relationships within and between clonal and solitary forms of the sea anemone *Anthopleura elegantissima* revisited: Evidence for the existence of two species. *Mar. Biol.* **128,** 127–139.

McGowan, J. A. (1967) *Distributional atlas of pelagic molluscs in the California Current region*. (CalCOFI Atlas No. 6).

McLean, J. H. (1966) *West American prosobranch Gastropoda: Superfamilies Patellacea, Pleurotomariacea and Fissurellacea.* PhD thesis, Stanford University.

Mecklenburg, C. W., Mecklenburg, T. A. & Thorsteinson, L. K. (2002) *Fishes of Alaska*. (American Fisheries Society).

Merlo, E. M. *et al*. (2018) Range extension for the region of sympatry between the nudibranchs *Hermissenda opalescens* and *Hermissenda crassicornis* in the Northeastern Pacific. FACETS **3,** 764–776.

Merrill, R. J. & Hobson, E. S. (1970) Field observations of *Dendraster excentricus*, a sand dollar of western North America. *Am. Midl. Nat.* **83,** 595–624.

Millen, S. V. (1983) Range extensions of opisthobranchs in the northeastern Pacific. *Veliger* **25,** 383–386.

Mills, C. E. & Larson, R. J. (2007) Scyphozoa: Scyphomedusae, Stauromedusae, and Cubomedusae. [Carlton, J. T. (ed.)]. *The Light and Smith Manual: Intertidal Invertebrates from Central California to Oregon. Fourth Edition*. Pp. 168–173 (University of California Press).

Mooi, R. (1997) Sand dollars of the genus *Dendraster* (Echinoidea : Clypeasteroida): Phylogenetic systematics, heterochrony, and distribution of extant species. *Bull. Mar. Sci.* **61,** 343–375.

Morandini, A. C. & Marques, A. C. (2010) Revision of the genus *Chrysaora* Peron & Lesueur, 1810 (Cnidaria: Scyphozoa). *Zootaxa*, 1–97.

Morris, R. H., Abbott, D. P. & Haderlie, E. C. (1980) *Intertidal Invertebrates of California*. (Stanford University Press).

Newman, W. A. & Abbott, D. P. (1980) Cirripedia. [Morris, R. H., Abbott, D. P. & Haderlie, E. C. (eds.)]. *Intertidal Invertebrates of California.* Pp. 504–535 (Stanford University Press).

Newman, W. A. & McConnaughey, R. R. (1987) A tropical eastern Pacific barnacle, *Megabalanus coccopoma* (Darwin), in southern California, following El Niño 1982–83. *Pacific Science* **41,** 31–36.

NMFS and USFWS. (1998a) *Recovery Plan for U.S. Pacific Populations of the Green Turtle (Chelonia mydas)*. (National Marine Fisheries Service).

NMFS and USFWS. (1998b) *Recovery Plan for U.S. Pacific Populations of the Olive Ridley Turtle (Lepidochelys olivacea)*. (National Marine Fisheries Service).

Nybakken, J. (1978) Abundance, diversity and temporal variability in a California intertidal nudibranch assemblage. *Mar. Biol.* **45,** 129–146.

Oregon Bird Records Committee. (2018) The Records of the Oregon Bird Records Committee. http://www.orbirds.org/recordsmay2018.pdf

Pandolfino, E., Rottenborn, S. C., Rogers, M. M. & Davis, J. N. (2016) The Regional Reports: Northern California. *North American Birds* **69,** 288–292.

Pattie, B. H. & Baker, C. S. (1969) Extensions of the known northern range limits of Ocean Whitefish, *Caulolatilus princeps*, and California Halibut, *Paralichthys californicus*. *Journal of the Fisheries Research Board of Canada* **26,** 1371–1372.

Pearcy, W., Fisher, J., Brodeur, R. & Johnson, S. (1985) Effects of the 1983 El Niño on coastal nekton off Oregon and Washington. [Wooster, W. S. & Fluharty, D. L. (eds.)]. *El Niño North: Niño effects in the Subarctic Pacific Ocean.* Pp. 188–204 (Washington Sea Grant Program, Seattle, WA).

Pearcy, W. G. & Schoener, A. (1987) Changes in the marine biota coincident with the 1982–1983 El Niño in the northeastern subarctic Pacific Ocean. *Journal of Geophysical Research-Oceans* **92,** 14417–14428.

Pearse, V. & Francis, L. (2000) *Anthopleura sola*, a new species, solitary sibling species to the aggregating sea anemone, *A. elegantissima* (Cnidaria: Anthozoa: Actiniaria: Actiniidae). *Proc. Biol. Soc. Wash.* **113,** 596–608.

Pickens, F. A. T., Ortega, J. D., Le, A. P. & Konstantinidis, P. (2018) First records of the Pacific Snake Eel, *Ophichthus triserialis*, in Oregon waters. *Northwestern Naturalist* **99,** 239–241.

Preovolos, C. (2015) "Endangered sea turtle spotted in San Joaquin River." https://www.sfgate.com/news/article/Rare-sea-turtle-spotted-in-California-6640769.php

Pynn, L. (2015) "New species of dolphin to B.C. waters shows up alive for the first time." *Vancouver Sun* (1 December 2015).

Quirollo, L. F. & Dinnel, P. A. (1975) Latitudinal range extensions for yellow and spotted snake eels (genus *Ophichthus*). *Calif. Fish Game* **61,** 156–157.

Radovich, J. (1961) Relationships of some marine organisms of the northeast Pacific to water temperatures particularly during 1957 through 1959. *Fish Bull. Sacramento* **No. 112,** 1–62.

Ricketts, E. F. & Calvin, J. (1968) *Between Pacific Tides*. *Fourth edition*. [Hedgpeth, J. W. (ed.)] (Stanford University Press).

Ristau, D. A., Tarp, C. & Hand, C. (1978) A Survey of the Open Coast at Bodega Marine Life Refuge (Area of Special Biological Significance).

Robinson, J. G. (1965) New northern record for ocean whitefish. *Res Briefs Fish Comm Oregon* **11,** 52.

Rogers, M. M., Davis, J. N., Pandolfino, E. & Rottenborn, S. C. (2015) The Regional Reports: Northern California. *North American Birds* **68,** 549–552.

Rottenborn, S. C., Rogers, M. M., Davis, J. N. & Pandolfino, E. (2016) The Regional Reports: Northern California. *North American Birds* **69,** 482–489.

Rutherford, J. C. (1975) Class Holothuroidea. [Smith, R. I. & Carlton, J. T. (eds.)]. *Light's Manual: Intertidal Invertebrates of the Central California Coast. Third edition*. Pp. 634–637 (University of California Press).

Sadowski, J. S. *et al*. (2018) Temperature-induced range expansion of a subtropical crab along the California coast. *Marine Ecology* **39,** e12528; doi:10.1111/maec.12528.

Sagarin, R. D., Barry, J. P., Gilman, S. E. & Baxter, C. H. (1999) Climate-related change in an intertidal community over short and long time scales. *Ecol. Monogr.* **69,** 465–490.

Sakuma, K. M. *et al.* (2016) Anomalous epipelagic micronekton assemblage patterns in the neritic waters of the California Current in spring 2015 during a period of extreme ocean conditions. *Calif. Coop. Ocean. Fish. Invest. Rep.* **57,** 163–183.

Savilov, A. L. (1969) Pleuston of the Pacific Ocean. [Zenkevich, L. A. (ed.)]. *The Pacific Ocean. Biology of the Pacific Ocean. Vol. 7, Part 2.* Pp. 264–353 (Moscow Publishing House "Nauka").

Searcy, A. J., Daniels, B. E., Feenstra, J. S., Tietz, J. R. & Benson, T. A. (2018) The 41st Annual Report of the California Bird Records Committee: 2015 Records. *Western Birds* **49,** 24–46.

Serfling, S. A. & Ford, R. F. (1975) Ecological studies of the puerulus larval stage of the Californian spiny lobster *Panulirus interruptus*. *Fish. Bull.* **73,** 360–377.

Shiomi, K. & Ogi, H. (1992) Feeding ecology and body size dependence on diet of the Sooty Shearwater (*Puffinus griseus*) in the North Pacific. *Proc. NIPR Symp. Polar Biol.* **5,** 105–113.

Sims, D. W., Queiroz, N., Doyle, T. K., Houghton, J. D. R. & Hays, G. C. (2009) Satellite tracking of the World's largest bony fish, the ocean sunfish (*Mola mola* L.) in the North East Atlantic. *J. Exp. Mar. Biol. Ecol.* **370,** 127–133.

Sorte, C. J., Peterson, W. T., Morgan, C. A. & Emmett, R. L. (2001) Larval dynamics of the sand crab, *Emerita analoga*, off the central Oregon coast during a strong El Niño period. *J. Plankton Res.* **23,** 939–944.

Soule, D. F., Soule, J. D. & Chaney, H. W. (2005) The Bryozoa. [Blake, J. A., Chaney, W. Scott, P. H. & Lissner, A. L. (eds.)]. *Taxonomic Atlas of the Santa Maria Basin and Western Santa Barbara Channel* Vol. 13. Pp. 1–344 (Santa Barbara Museum of Natural History).

Soule, D. F., Soule, J. D., Morris, P. A. & Chaney, H. W. (2007) Bryozoa. [Carlton, J. T. (ed.)]. *The Light and Smith Manual: Intertidal Invertebrates from Central California to Oregon. Fourth edition*. Pp. 866–904 (University of California Press).

Spear, L. B. & Ainley, D. G. (2007) Storm-petrels of the eastern Pacific Ocean: Species assembly and diversity along marine habitat gradients. *Ornithological Monographs* **62,** 1–77.

Standing, J. D., Browning, B. & Speth, J. W. (1975) The Natural Resources of Bodega Harbor. (California Department of Fish and Game).

Stearns, R. E. C. (1867) List of shells collected at Bodega Bay, California, June, 1867. *Proceedings of the California Academy of Sciences Series 4* **3,** 382–383.

Sutherland, K. R., Sorensen, H. L., Blondheim, O. N., Brodeur, R. D. & Galloway, A. W. W. (2018) Range expansion of tropical pyrosomes in the northeast Pacific Ocean. *Ecology* **99,** 2397–2399.

Swezey, D. S. *et al.* (2017) Interactive effects of temperature, food and skeletal mineralogy mediate biological responses to ocean acidification in a widely distributed bryozoan. *Proc. R. Soc. B-Biol. Sci.* **284,** 20162349; doi:10.1098/rspb.2016.2349.

Tesch, J. J. (1946) The thecosomatous pteropods. I. The Atlantic. *Dana Report No. 28*, 1–82.

Tesch, J. J. (1948) The thecosomatous pteropods. II. The Indo-Pacific. *Dana Report No. 30*, 1–44.

Tietz, J. & McCaskie, G. (2018) Update to Rare Birds of California: 1 January 2004–20 May 2018. https://californiabirds.org/cbrc_book/update.pdf

Tilbrook, K. J., Hayward, P. J. & Gordon, D. P. (2001) Cheilostomatous Bryozoa from Vanuatu. *Zool. J. Linn. Soc.* **131,** 35–109.

Tobish, T. (2016) The Regional Reports: Alaska. *North American Birds* **69,** 138–141.

Tobish, T. (2017) The Regional Reports: Alaska. *North American Birds* **70,** 99–103.

Valdes, M. (2016) "Recovering sea turtles flown from Seattle to San Diego." *San Diego Union-Tribune* (21 April 2016).

Waggoner, B. & Merrill, R. J. (2017) The Regional Reports: Oregon and Washington. *North American Birds* **70,** 108–111.

Washington Birds Committee. (2017) Summary of records accepted by the Washington Birds Records Committee. http://wos.org/documents/WBRC/WBRC Accepted Oct 2017.pdf

Wasson, K., Lyon, B. E. & Knope, M. (2002) Hair-trigger autotomy in porcelain crabs is a highly effective escape strategy. *Behav. Ecol.* **13,** 481–486.

Weisbord, N. E. (1979) Lepadomorph and verrucomorph barnacles (Cirripedia) of Florida and adjacent waters, with an addendum of the Rhizocephala. *Bulletin of American Paleontology* **76,** 1–156.

Wells, B. K. *et al.* (2013) State of the California Current 2012–13: No such thing as an "average" year. *Calif. Coop. Ocean. Fish. Invest. Rep.* **54,** 37–71.

Wells, B. K. *et al*. (2017) State of the California Current 2016–17: Still anything but “normal” in the north. *Calif. Coop. Ocean. Fish. Invest. Rep.* **58,** 1–55.

Wells, R. S. *et al.* (1990) Northward extension of the range of Bottlenose Dolphins along the California Coast. [Leatherwood, S. & Reeves, R. R. (eds.)]. *The Bottlenose Dolphin.* Pp. 421–431 (Academic Press).

Wonham, M. J. & Hart, M. W. (2018) El Niño range extensions of Pacific Sand Crab (*Emerita analoga*) in the Northeastern Pacific. *Northwest Sci.* **92,** 53–60.

Wootten, D. (1949) *The development of Thyonepsolus nutriens Clark.* PhD thesis, Stanford University.

Wrobel, D. and Mills, C. (1998) *Pacific Coast Pelagic Invertebrates: A Guide to the Common Gelatinous Animals*. (Monterey, California, Sea Challengers and the Monterey Bay Aquarium).

Zabin, C. J. *et al.* (2013) A comparison of intertidal species richness and composition between Central California and Oahu, Hawaii. *Mar. Ecol.-Evol. Persp.* **34,** 131–156.

Zullo, V. A. (1968) Extension of range for *Balanus tintinnabulum californicus* Pilsbry, 1916 (Cirripedia, Thoracica). *Occasional Papers of the California Academy of Sciences* **70,** 1–3.

D. SUPPLEMENTARY TABLES

Table S1. Names and geographic coordinates of all study sites and collection locations referred to in the text and Supplementary Information. CA = California; OR = Oregon; WA = Washington; AK = Alaska; BC = British Columbia, Canada. Listed alphabetically by site name.

Site Name	Latitude	Longitude
Abalone Beach, CA	41.1225 N	-124.158889 W
Abbotts Lagoon, CA	38.125845 N	-122.956785 W
Agate Beach, OR	44.65821 N	-124.06085 W
Amlia Island, AK	52.083360 N	-173.556035 W
Año Nuevo Cove, CA	37.11285 N	122.329080 W
Arcadia Beach, OR	45.847350 N	-123.961220 W
Bahia Ballenas, Baja California	26.7083 N	-113.571 W
Bamfield, BC	48.833435 N	-125.142430 W
Bandon, OR	43.091380 N	-124.434137 W
Biorka Island, AK	56.853250 N	-135.538570 W
Bodega Bay, CA	38.305175 N	-123.053030 W
Bodega Canyon	38.206877 N	-123.593028 W
Bodega Harbor, CA	38.322295 N	-123.049540 W
Bodega Line Station 4	38.194175 N	-123.400000 W
Bodega Marine Laboratory oceanographic buoy	38.31180556 N	-123.083028 W
Bodega Marine Reserve, CA	38.318740 N	-123.074110 W
Boiler Bay, OR	44.831380 N	-124.060930 W
Bolinas Lagoon, CA	37.921145 N	-122.680105 W
Brookings, OR	42.052830 N	-124.284350 W
Bruhel Point, CA	39.602060 N	-123.788920 W
Burnt Hill, OR	42.22796 N	-124.38774 W
Calvert Island, BC	51.558070 N	-128.037400 W
Cape Arago, OR	43.304765 N	-124.400805 W
Cape Flattery, WA	48.38265 N	-124.714540 W
Cape Meares, OR	45.485810 N	-123.974710 W
Cape Mendocino, CA	40.341370 N	-124.362620 W
Cape Sebastian, OR	42.334250 N	-124.421895 W
Channel Islands, CA	33.469065 N	-119.418375 W
Charleston Boat Basin, OR	43.3453 N	-124.3220 W
Chup Point, BC	48.9562 N	-125.8937 W
Coleman Beach, CA	38.362950 N	-123.070679 W
Coos Bay, OR	43.356955 N	-124.327915 W
Coos Bay entrance, OR	43.35048 N	-124.329216 W
Copalis Beach, WA	47.112185 N	-124.179605 W
Cordell Bank	38.033060 N	-123.444035 W

Site Name	Latitude	Longitude
Cordova, AK	60.542195 N	-145.752105 W
Crescent City, CA	41.756115 N	-124.202985 W
Crissy Field, CA	37.805580 N	-122.465220 W
Curry County, OR	42.544050 N	-124.267610 W
Devil's Gate, CA	40.396 N	-124.378 W
Dillon Beach, CA	38.254200 N	-122.969385 W
Doran Beach, CA	38.312590 N	-123.033475 W
Drakes Beach, CA	38.027485 N	-122.961645 W
Drakes Estero, CA	38.054200 N	-122.940765 W
Duxbury Reef, CA	37.901315 N	-122.720366 W
Elkhorn Slough, CA	36.824265 N	-121.740505 W
Estero de San Antonio, CA	36.270215 N	-122.979905 W
Ensenada, Baja California	31.867175 N	-116.596840 W
Farallon Islands, CA	37.698795 N	-123.003250 W
Fort Bragg, CA	39.4513 N	-123.8139 W
Fort Funston, CA	37.719780 N	-122.502880 W
Fort Ross Reef, CA	38.502030 N	-123.230115 W
Fort Ross State Park, CA	38.512700 N	-123.247720 W
Golden Gate, CA	37.819325 N	-122.478585 W
Gregory Point, OR	43.3400 N	-124.3749 W
Grays Canyon, WA	46.9167 N	-125.183 W
Guadalupe Island, Baja California	29.057150 N	-118.282230 W
Haines, AK	59.236135 N	-135.446325 W
Half Moon Bay, CA	37.464020 N	-122.428660 W
Haven's Neck, CA	38.809030 N	-123.601150 W
Horseshoe Cove (Bodega Marine Reserve), CA	38.316795 N	-123.069410 W
Humboldt Bay, CA	40.760990 N	-124.234300 W
Humbug State Park, OR	42.683253 N	-124.449214 W
Isla Todos Santos, Baja California	31.811590 N	-116.805050 W
Ketchikan, AK	55.342390 N	-131.645890 W
Klamath River, CA	41.542840 N	-124.079830 W
Kodiak Island, AK	57.498820 N	-153.478575 W
Kyuquot, BC	50.030775 N	-127.378720 W
La Jolla, CA	32.859260 N	-117.257165 W
Leadbetter Point, WA	46.630833 N	-124.0716 W
Lighthouse Beach, OR	43.340167 N	-124.364028 W
Lincoln City, OR	44.958945 N	-124.017350 W
Little River, CA	39.271975 N	-123.790050 W
Los Angeles, CA	34.051260 N	-118.246560 W
Lost Creek State Park, OR	44.545490 N	-124.074015 W

Site Name	Latitude	Longitude
Macaulay Waste Water Treatment Plant, BC	48.4035 N	-123.407516 W
MacKerricher State Park, CA	39.536724 N	-123.772568 W
Mad River Beach, CA	40.916195 N	-124.140865 W
Manchester Beach, CA	38.958 N	-123.727 W
Mendocino, CA	39.307795 N	-123.799480 W
Miwok Beach, CA	38.361470 N	-123.069915 W
Monas Island, BC	49.187245 N	-125.884340 W
Monterey Bay, CA	36.80405 N	-121.945835 W
Moresby Island, BC	48.701135 N	-123.302475 W
Morro Bay, CA	35.375820 N	-120.875625 W
Moss Beach, CA	37.524040 N	-122.518115 W
Neah Bay, WA	48.368465 N	-124.624260 W
Neskowin, OR	45.106835 N	-123.985110 W
Nestucca River, OR	45.156530 N	-123.972930 W
Netarts Bay, OR	45.429470 N	-123.947760 W
Newport, OR	44.638855 N	-124.063355 W
Ocean Beach, CA	37.759800 N	-122.510845 W
Oyster Point Marina, CA	37.662410 N	-122.374810 W
Pacific Grove, CA	36.619150 N	-121.941730 W
Patrick's Point State Park, CA	41.131970 N	-124.164030 W
Pigeon Point, CA	37.181380 N	-122.394180 W
Pill Point, BC	48.966325 N	-125.083175 W
Pillar Point Harbor, CA	37.501618 N	-122.483152 W
Pinnacle Gulch, CA	38.305185 N	-123.077205 W
Point Cabrillo, CA	39.348890 N	-123.827135 W
Point Conception, CA	34.448789 N	-120.471570 W
Point Delgada, CA	40.021645 N	-124.069105 W
Point Reyes, CA	38.018465 N	-122.991345 W
Point St. George, CA	41.770605 N	-124.253885 W
Point Sur, CA	36.306730 N	-121.902140 W
Port Hardy, BC	50.743919 N	-127.496977 W
Princess Royal Island, BC	52.682022 N	-128.952633 W
Rainy Bay, BC	48.968870 N	-125.022978 W
Rancho Palos Verdes, CA	33.741045 N	-118.386750 W
Redding Rock, CA	41.383333 N	-124.35 W
Russian River mouth, CA	38.451420 N	-123.131300 W
Salisbury Sound, AK	57.355556 N	-135.733333 W
Salmon Creek Beach, CA	38.324860 N	-123.072650 W
Salt Point State Park, CA	38.565245 N	-123.333850 W
San Benito Islands, Baja California	28.304705 N	-115.588350 W

Site Name	Latitude	Longitude
San Diego, CA	32.304705 N	-117.164600 W
San Francisco Bay, CA	37.843280 N	-122.396560 W
San Joaquin River, CA	37.785485 N	-121.306450 W
San Miguel Island, CA	34.038960 N	-120.372595 W
Santa Barbara, CA	34.411760 N	-119.689195 W
Saunders Reef, CA	38.866667 N	-123.655820 W
Seal Rock, OR	44.497115 N	-124.083225 W
Sea Ranch, CA	38.731111 N	-123.49 W
Seaside, OR	45.993880 N	-123.922860 W
Shell Beach, CA	38.417430 N	-123.107280 W
Shelter Cove, CA	40.030590 N	-124.073105 W
Sitka, AK	57.053345 N	-135.330385 W
Southeast Farallon Island, CA	37.698795 N	-123.003250 W
South Jetty (Bodega Harbor), CA	38.304505 N	-123.054190 W
South Spit, CA	40.741270 N	-124.243780 W
Tillamook Bay, OR	45.568400 N	-123.956410 W
Tofino, BC	49.153100 N	-125.906425 W
Tomales Bay, CA	38.213000 N	-122.955465 W
Tomales Point, CA	38.240895 N	-122.994750 W
Trinidad, CA	41.056495 N	-124.142825 W
Van Damme State Park, CA	39.281600 N	-123.803455 W
Vancouver Island, BC	49.656695 N	-125.428330 W
Westport, WA	46.888349 N	-124.129810 W
Whiskey Creek, OR	42.2227 N	-124.3830 W
Wickaninnish Beach, BC	49.034605 N	-123.034605 W
Wilson Creek, CA	41.601090 N	-124.106430 W
Yaquina Bay, OR	44.616460 N	-124.019600 W
Zayas Island, BC	54.602025 N	-131.076990 W

Table S2. Summary statistics for all marine heatwave events identified from an analysis of sea surface temperature (SST) data at **buoy 46013 (Bodega Bay)** during the period 2014–2016. Heatwaves were characterized following Hobday et al. (2016) as any event that lasted ≥5 days with SST warmer than the 90th percentile based on the 30-year climatology (1981–2011). Rows colored in red indicate events that rank among the 10 most intense events since 1981 (based on cumulative intensity, °C days). The most intense event occurred during August 2014–February 2015 lasting 199 days. The last two rows (in gold) indicate the 2nd and 3rd most intense events since 1981, for reference. See Hobday et al. (2016) for additional methods.

**Maximum intensity (°C)**	**Average intensity (°C)**	**Cumulative intensity (°C days)**	**Duration (days)**	**Start date**	**End date**
4.2	3.3	53.0	16	16 July 2014	31 July 2014
4.4	2.6	518.3	199	12 August 2014	26 February 2015
5.2	3.8	45.6	12	3 July 2015	14 July 2015
3.6	2.8	22.5	8	18 July 2015	25 July 2015
4.3	3.2	28.9	9	31 July 2015	8 August 2015
3.7	3.0	29.9	10	18 August 2015	27 August 2015
3.6	3.0	84.4	28	6 October 2015	2 November 2015
2.1	1.8	14.5	8	6 December 2015	13 December 2015
1.4	1.2	16.8	14	25 December 2015	7 January 2016
1.5	1.3	23.8	18	12 January 2016	29 January 2016
1.9	1.8	9.0	5	1 March 2016	5 March 2016
2.1	1.8	14.5	8	17 March 2016	24 March 2016
2.8	2.4	16.9	7	12 April 2016	23 April 2016
2.8	2.2	53.5	24	4 November 2016	27 November 2016
3.4	2.4	281.5	118	27 October 1997	21 February 1998
3.8	2.4	242.0	102	9 February 1992	20 May 1992

Table S3. Summary statistics for all marine heatwave events identified from an analysis of sea surface temperature (SST) data at **buoy 46014 (Point Arena)** during the period 2014–2016. Heatwaves were characterized following Hobday et al. (2016) as any event that lasted ≥5 days with SST warmer than the 90th percentile based on the 30-year climatology (1981–2011). Rows colored in red indicate events that rank among the 10 most intense events since 1981 (based on cumulative intensity, °C days). The last row (in gold) indicates the most intense event since 1981, for reference. The 2nd most intense event occurred during October–December 2014 lasting 74 days. See Hobday et al. (2016) for additional methods.

**Maximum intensity (°C)**	**Average intensity (°C)**	**Cumulative intensity (°C days)**	**Duration (days)**	**Start date**	**End date**
3.2	2.7	16.2	6	30 April 2014	5 May 2014
3.5	3.0	17.8	6	26 August 2014	31 August 2014
3.8	3.1	61.9	20	11 September 2014	30 September 2014
3.9	3.1	231.8	74	17 October 2014	29 December 2014
2.1	1.6	26.3	16	18 January 2015	2 February 2015
1.8	1.6	19.5	12	6 February 2015	17 February 2015
2.2	1.8	14.6	8	21 March 2015	28 March 2015
3.8	2.7	43.7	16	28 June 2015	13 July 2015
4.8	2.9	55.7	19	29 July 2015	16 August 2015
3.8	2.7	70.8	26	8 October 2015	2 November 2015
1.4	1.3	25.6	20	11 January 2016	30 January 2016
2.5	1.9	92.3	49	7 February 2016	26 March 2016
2.6	2.3	16.0	7	17 April 2016	23 April 2016
2.3	2.2	10.8	5	7 November 2016	11 November 2016
3.4	2.5	283.5	114	3 November 1997	24 February 1998

Table S4. Recorded egg-laying seasons in California and southern Oregon of heterobranch sea slugs found north of their usual ranges during the 2014–2016 marine heatwaves in the northeast Pacific Ocean (Goddard et al. 2016, 2018). We compiled dates of observation for species whose egg-laying has been recorded by multiple sources including MacFarland (1966), Costello (1938), Goddard (1984), Goddard (unpublished data, 1975–2018), and images on iNaturalist (www.inaturalist.org). We used observations from Rancho Palos Verdes, California (near the southern edge of the Oregonian Biogeographic Province) to Cape Arago, Oregon. Observations were lumped by season for each species, with winter defined as starting January 1. Most species of heterobranchs in the region hatch as planktotrophic veliger larvae from their egg masses after an embryonic period of 1–2 weeks, and then have obligatory planktonic periods of at least 1 month (Hadfield and Switzer-Dunlap 1984, Goddard 2004).

	**Season**			
**Species**	**Winter**	**Spring**	**Summer**	**Fall**
Acanthodoris rhodoceras		x	x	x
Anteaeolidiella oliviae		x	x	x
Aplysia californica		x	x	x
Berthella strongi	x	x		x
Crimora coneja		x	x	x
Dirona picta		x	x	x
Doriopsilla albopunctata		x	x	x
Doriopsilla fulva	x		x	x
Flabellinopsis iodinea	x	x	x	x
Hancockia californica		x	x	x
Janolus barbarensis			x	x
Navanax inermis	x	x	x	x
Okenia angelensis			x	
Okenia rosacea	x	x	x	x
Phidiana hiltoni	x	x	x	x
Polycera atra	x	x	x	x
Polycera hedgpethi		x	x	
Triopha maculata		x	x	x

E. SUPPLEMENTARY FIGURES:

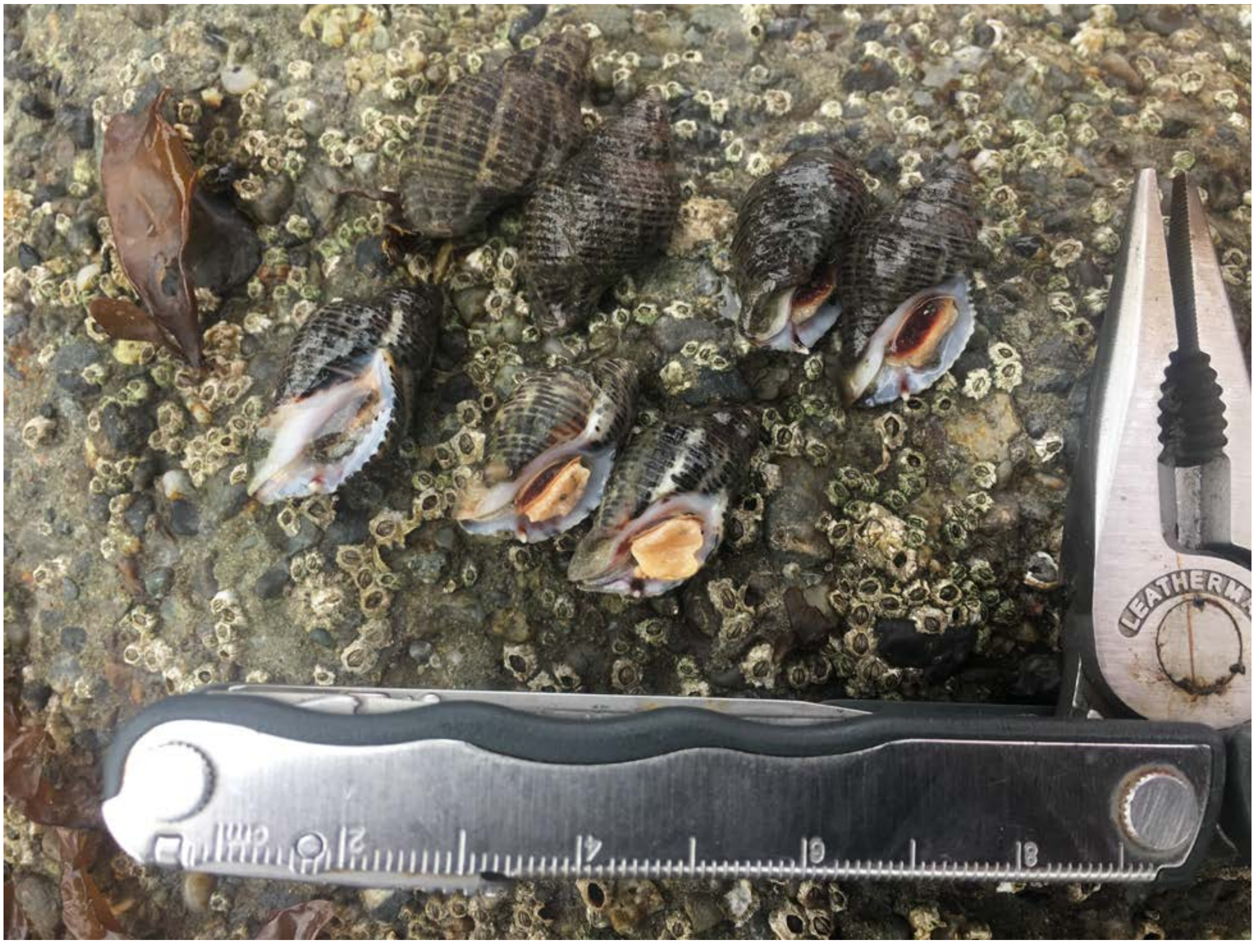

Figure S1. ***Acanthinucella spirata*** **(Angular Unicorn Snail).** Photographed on 10 June 2017 at Cape Mendocino, CA. Photo credit: Torre Flagor.

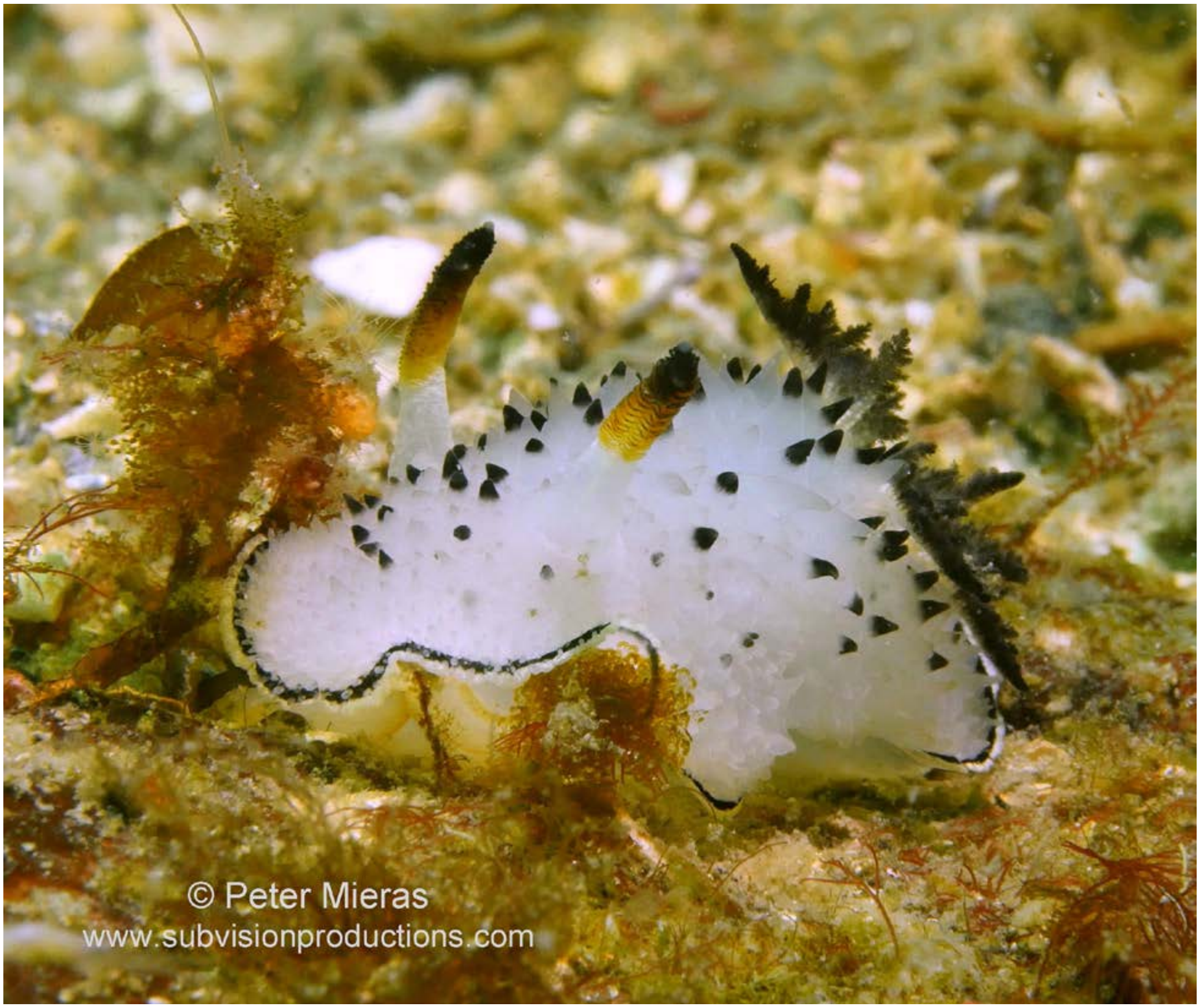

Figure S2. ***Acanthodoris rhodoceras*** **(Black-tipped Spiny Dorid Nudibranch).** Photographed on 21 May 2018 at Chup Point in Barkley Sound, British Columbia, Canada. Photo credit: © Peter Mieras/www.subvisionproductions.com

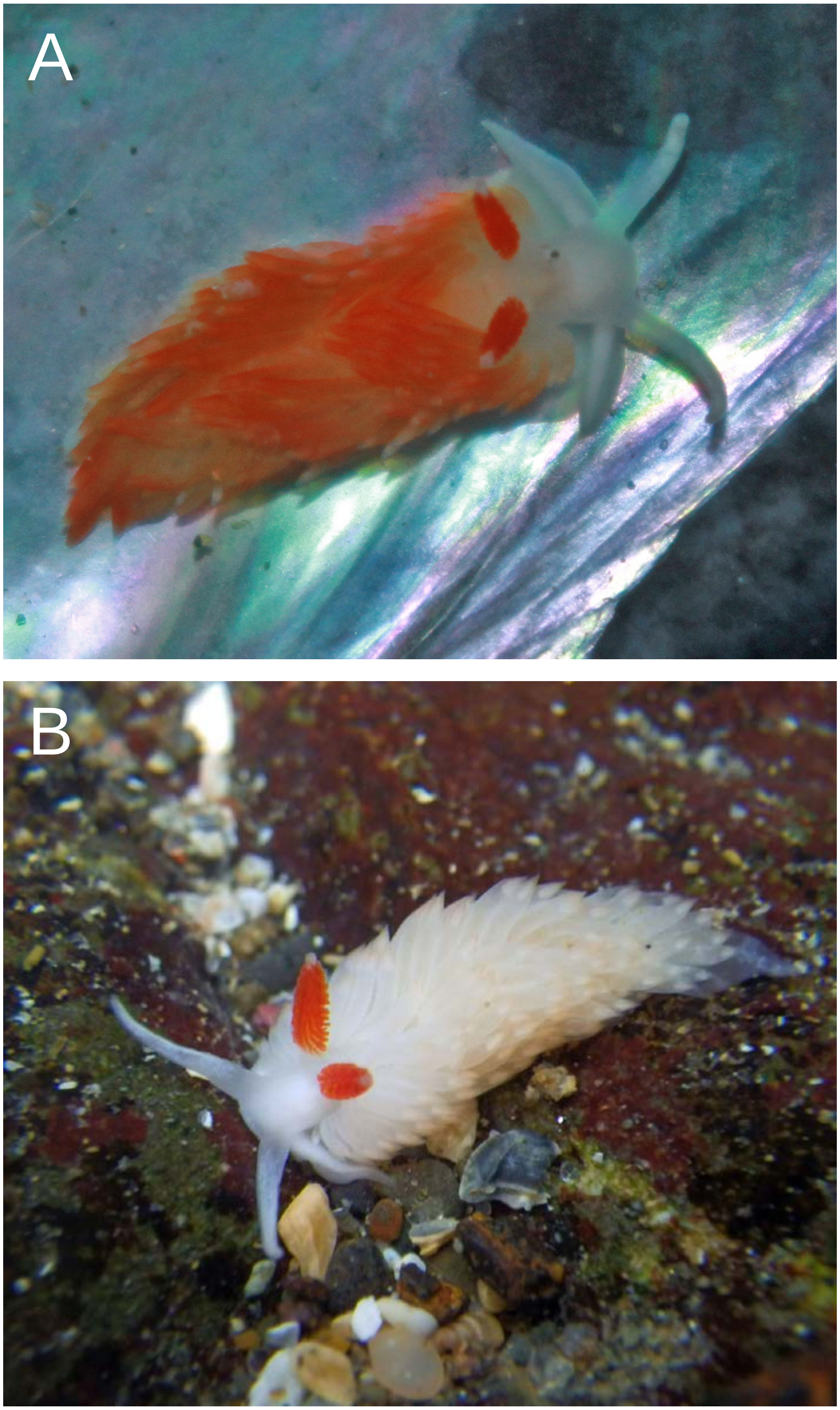

Figure S3. ***Anteaeolidiella oliviae*** **(Olive's Nudibranch).** **(A)** Photographed on 25 November 2015 at Pinnacle Gulch, CA. Photo credit: Jacqueline Sones. **(B)** Photographed on 16 February 2015 at Fort Bragg, CA. See Goddard et al. (2018). Photo credit: Douglas E. Mason.

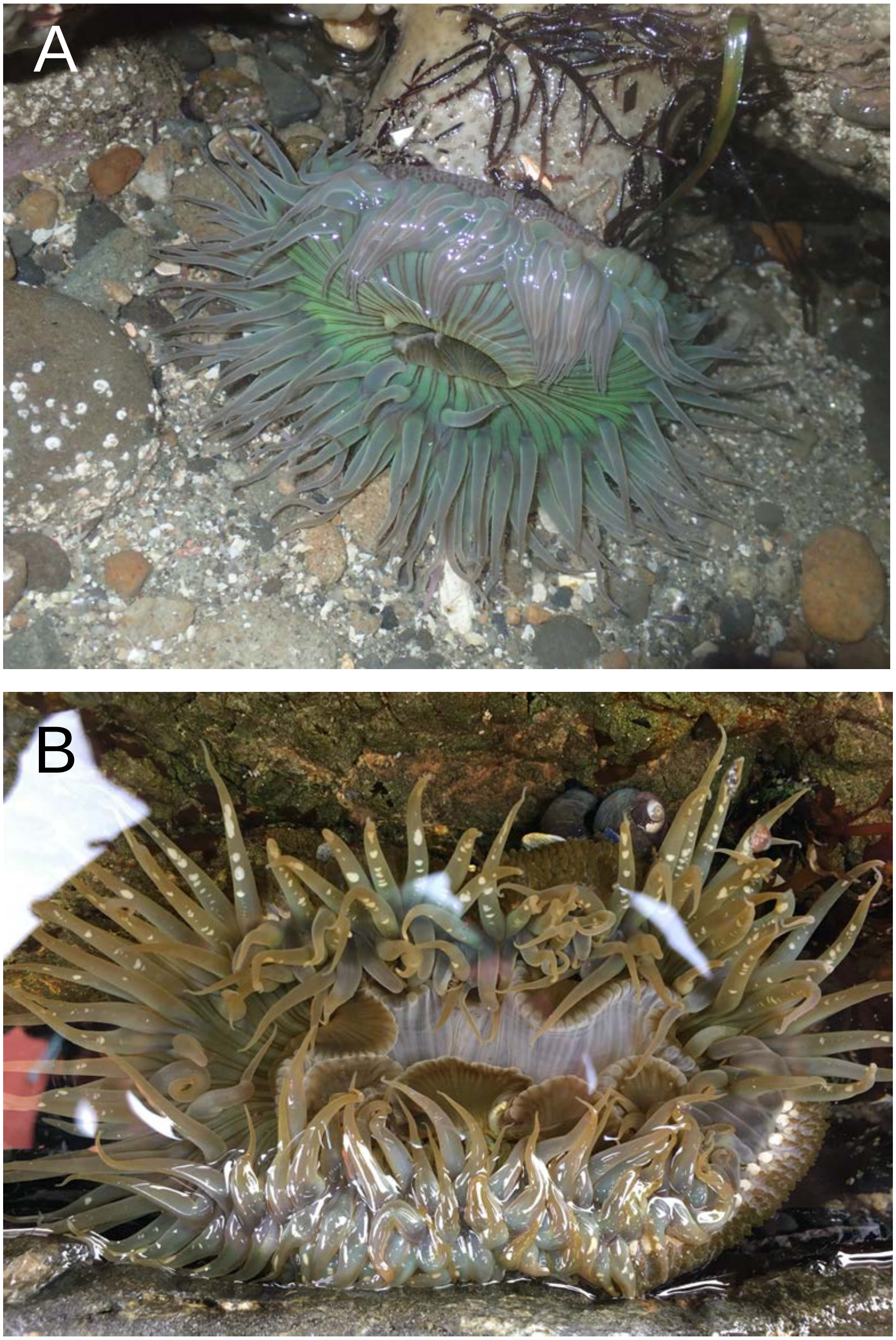

Figure S4. ***Anthopleura sola*** **(Sunburst Sea Anemone). (A)** Photographed on 2 November 2017 at Fort Ross State Park, CA. Photo credit: Jacqueline Sones. **(B)** Photographed on 27 July 2018 at Bruhel Point (Kibesillah Hill), CA. Photo credit: Seth Strumwasser.

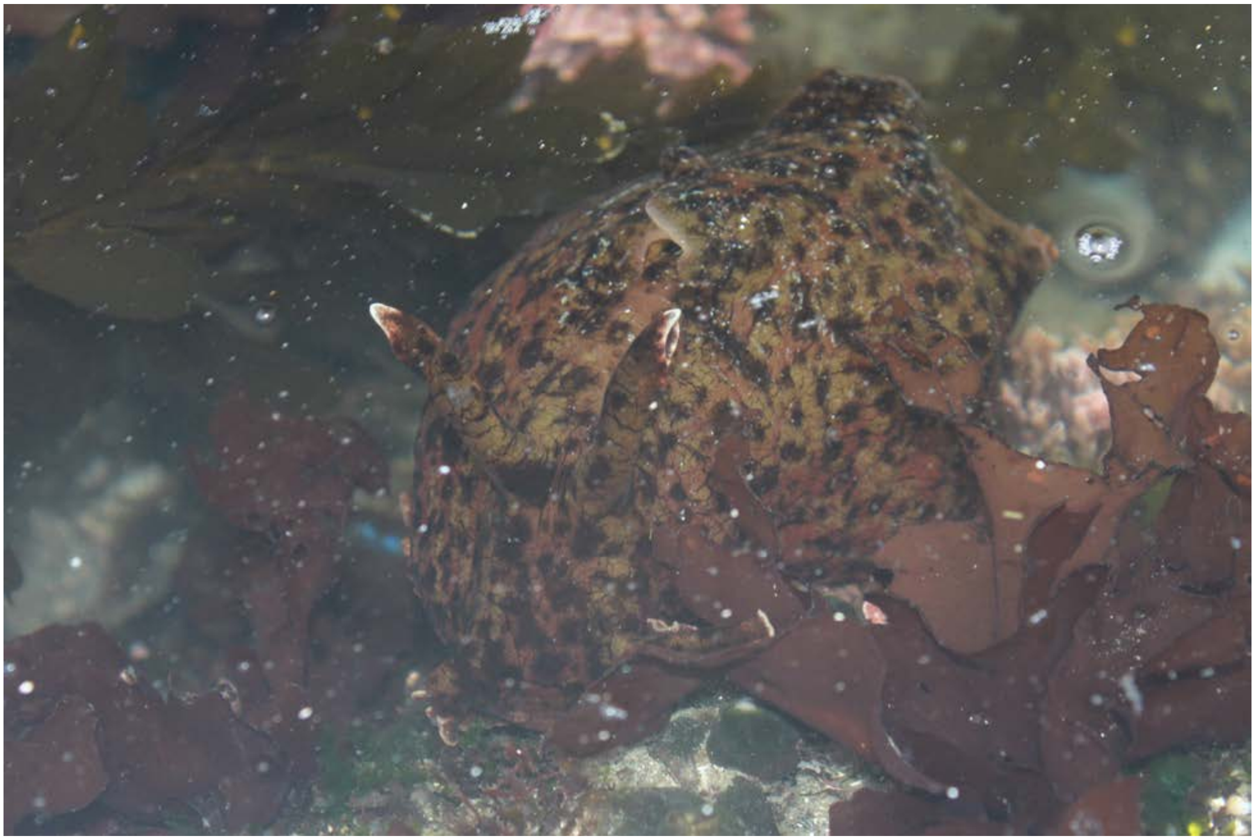

Figure S5. *Aplysia californica* (California Sea Hare). Photographed on 23 May 2015 at Miwok Beach, CA. Photo credit: Jacqueline Sones.

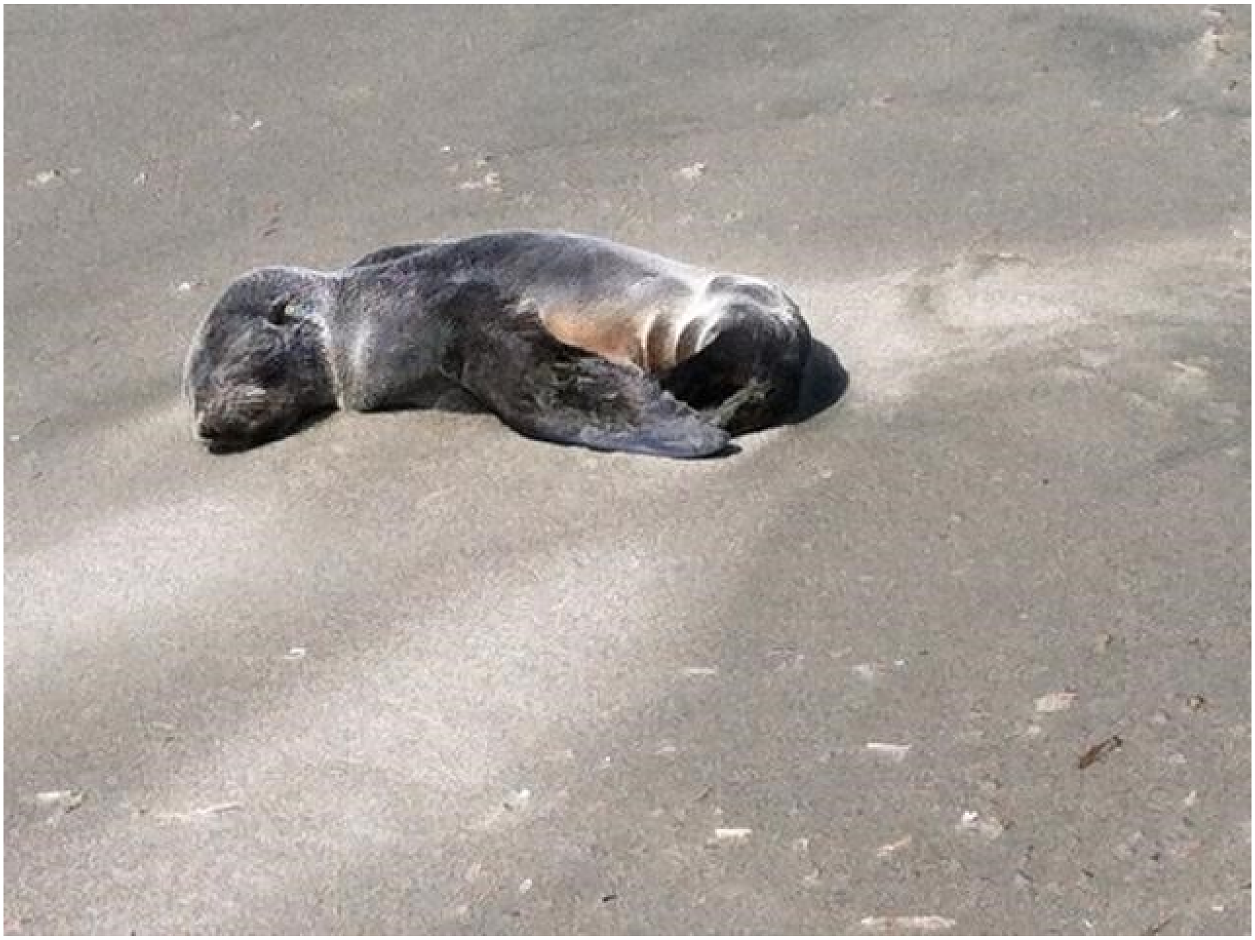

Figure S6. *Arctocephalus townsendi* (Guadalupe Fur Seal). Guadalupe Fur Seal pup photographed on 1 April 2015 at Doran Beach, CA. Photo credit: Phil Warren © The Marine Mammal Center / NOAA permit #932-1905-01.

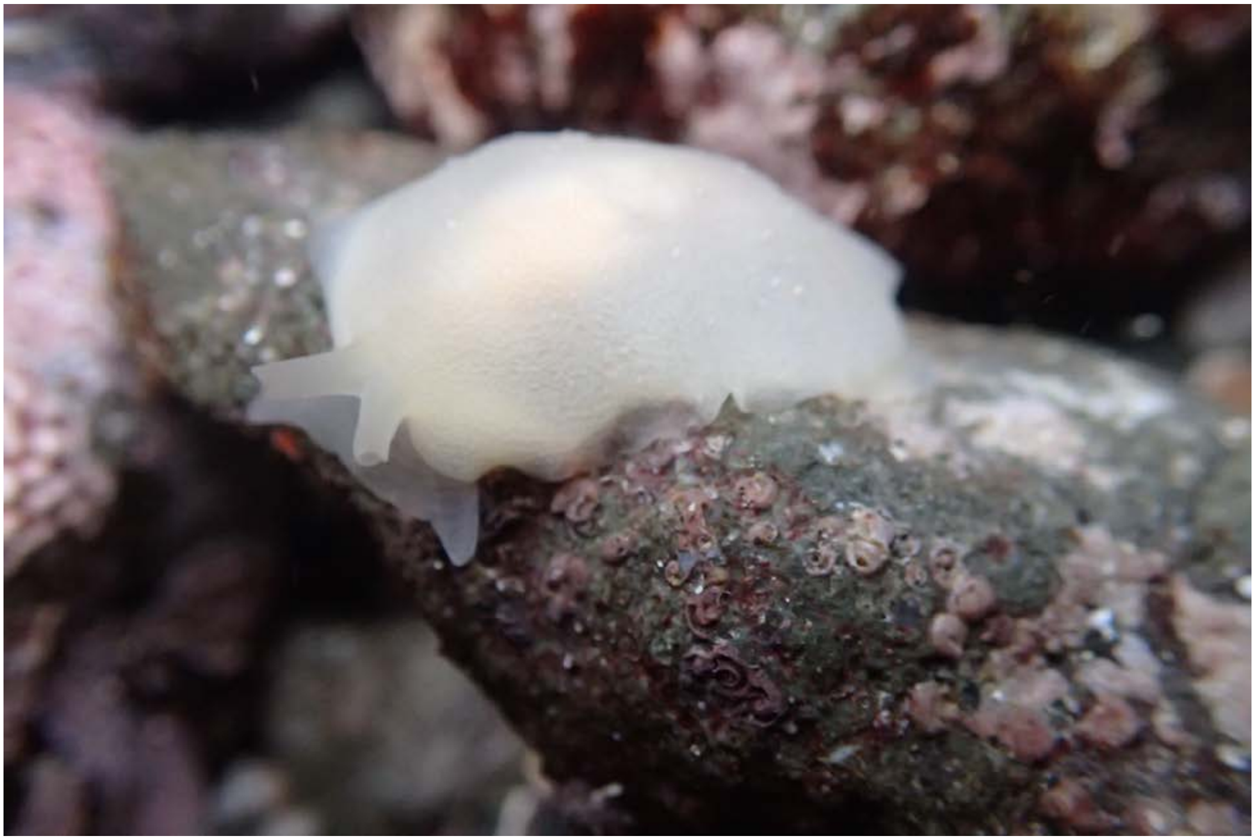

Figure S7. *Berthella strongi* (Strong's Sidegill). Photographed on 4 July 2016 at MacKerricher State Park, CA. Photo credit: Christopher Pirrone.

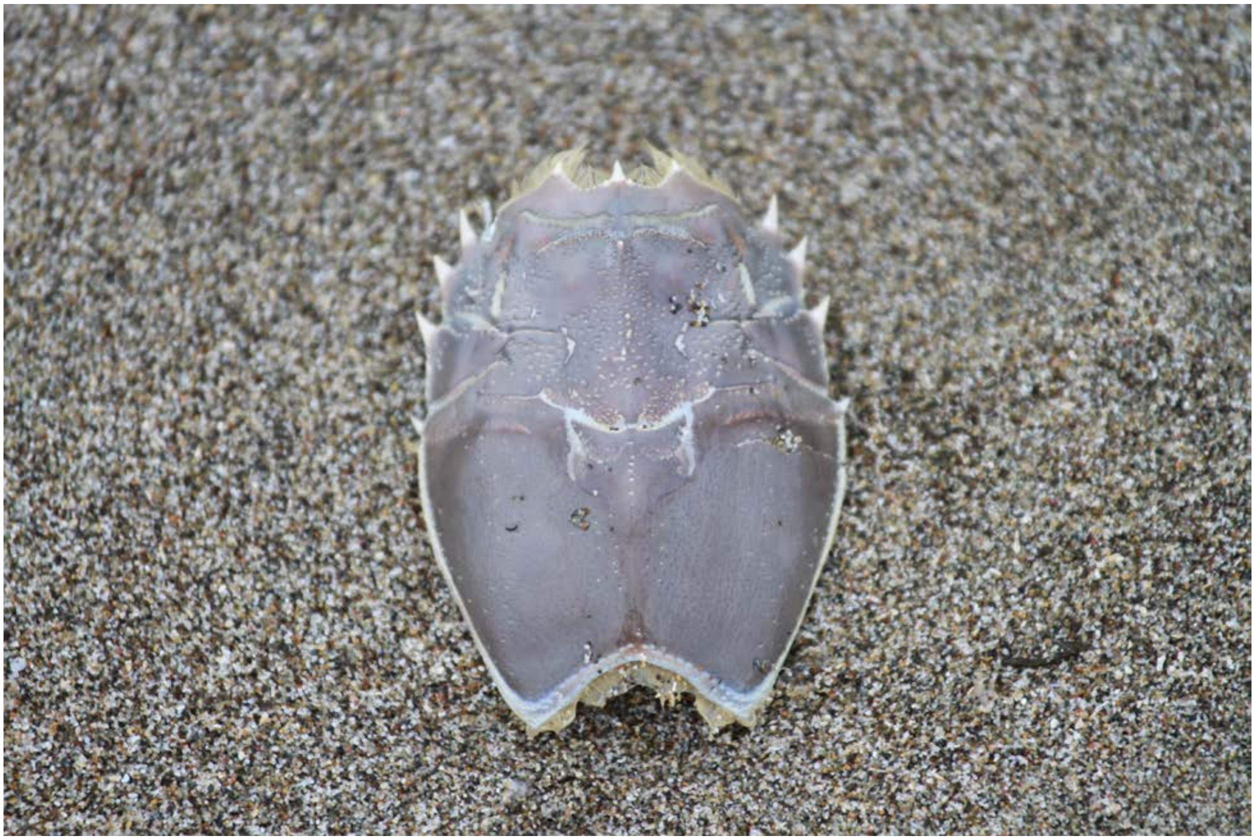

Figure S8. *Blepharipoda occidentalis* (Spiny Mole Crab). Molt photographed on 27 June 2015 at Salmon Creek Beach, CA. Photo credit: Jacqueline Sones.

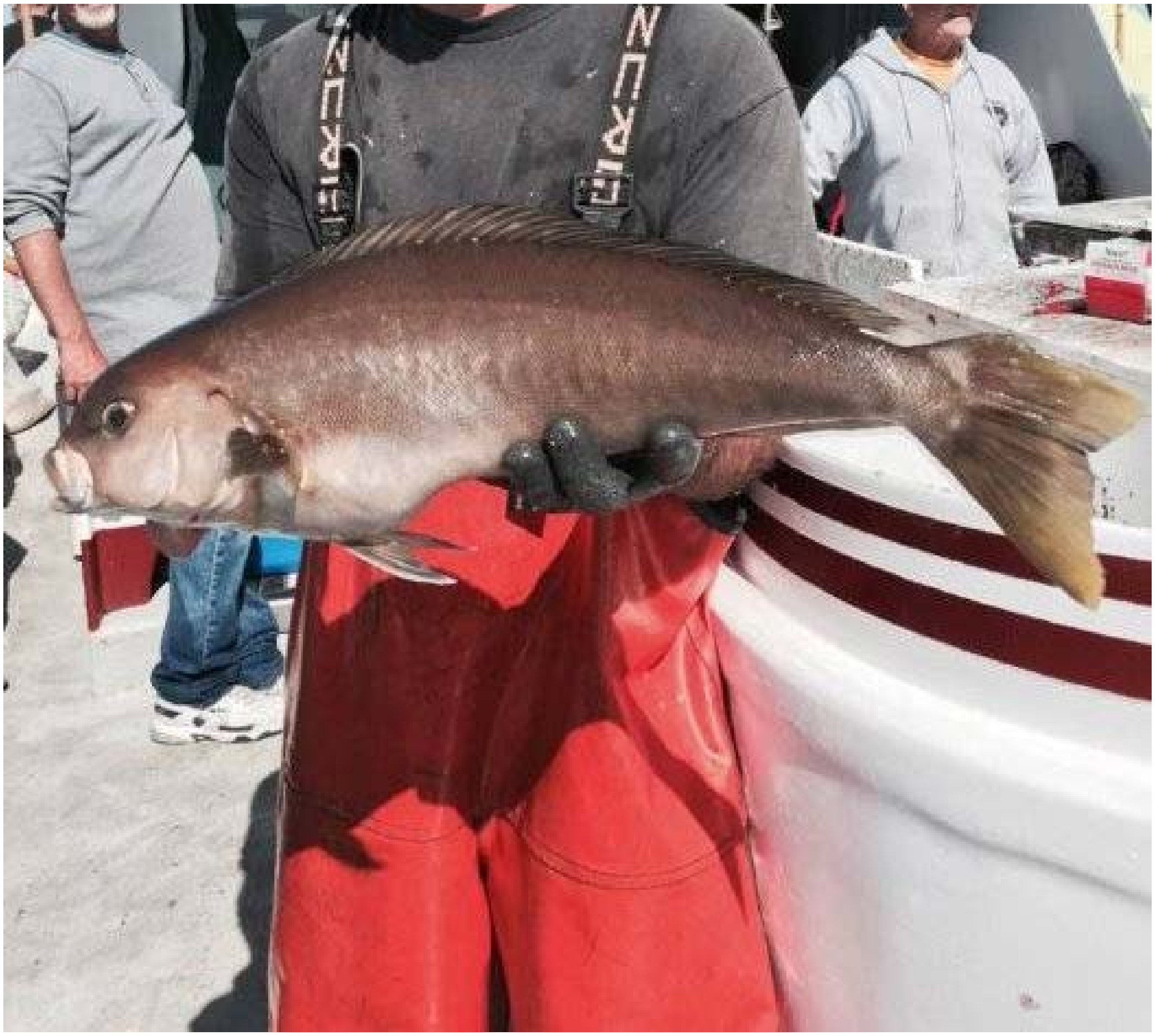

Figure S9. *Caulolatilus princeps* (Ocean Whitefish). Caught off the Farallon Islands, CA, on 10 July 2015. Photo credit: James Smith/ *California Dawn*.

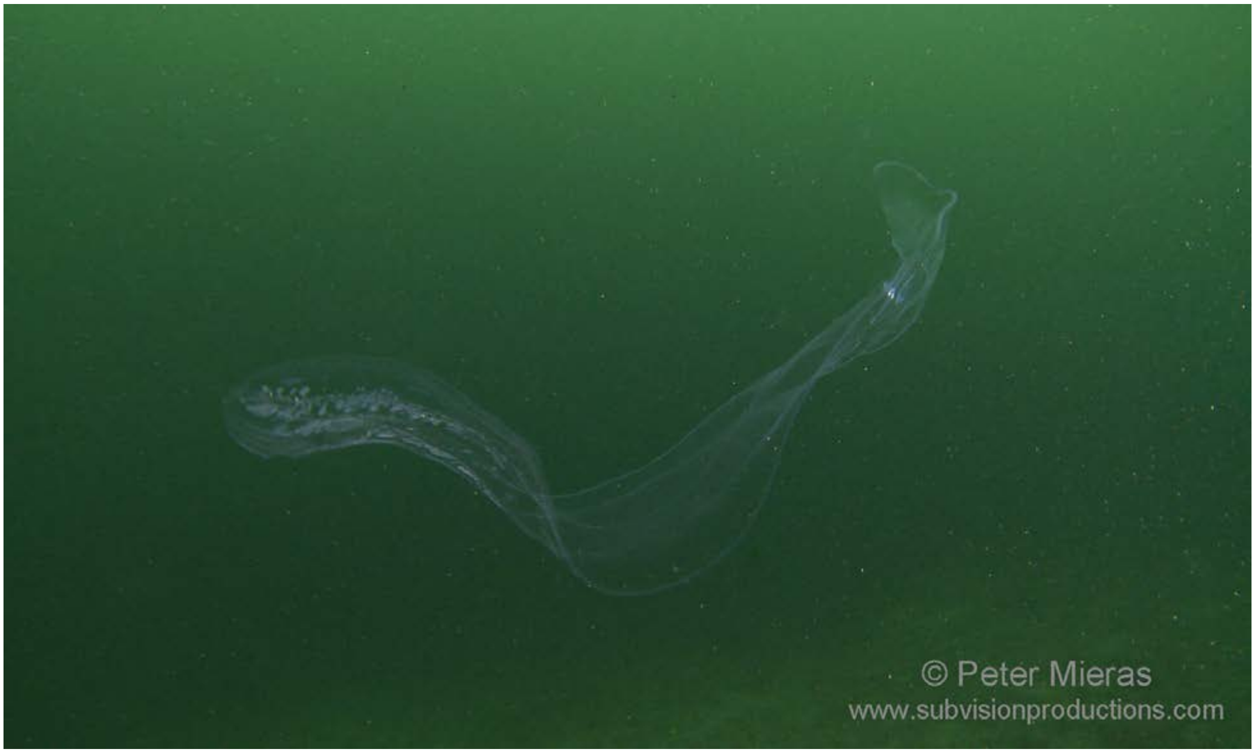

Figure S10. ***Cestum veneris*** **(Venus' Girdle Ctenophore).** Photographed on 1 April 2014 in Rainy Bay, Barkley Sound, British Columbia, Canada. Photo credit: © Peter Mieras/www.subvisionproductions.com

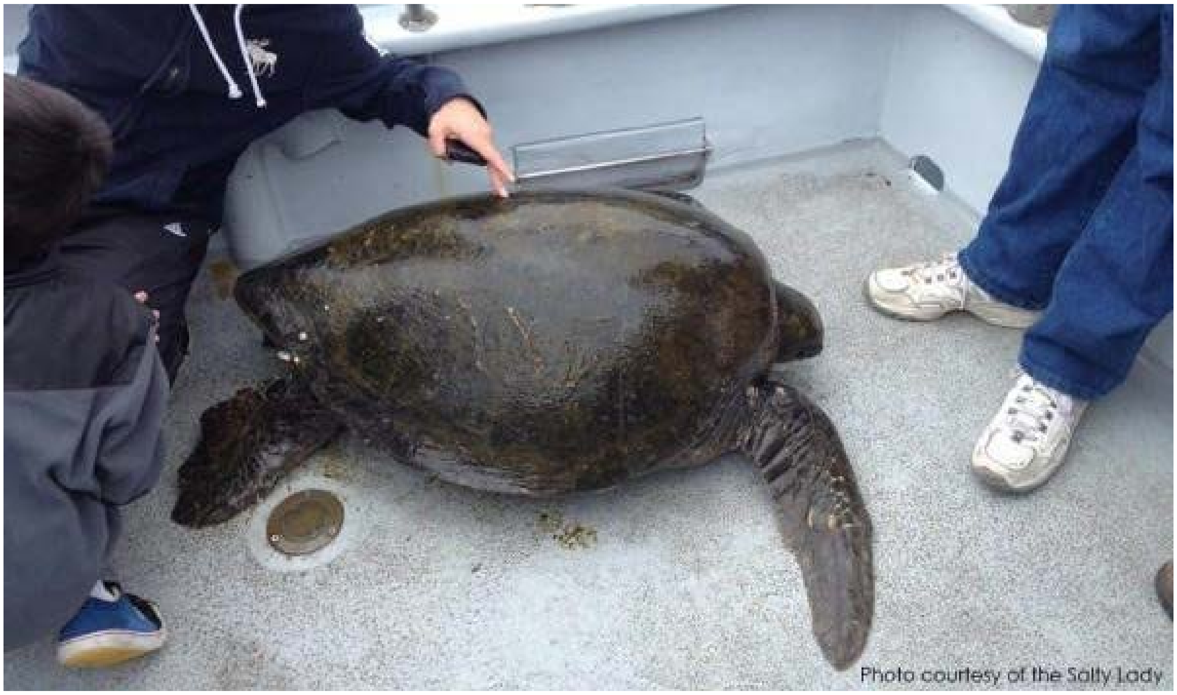

Figure S11. *Chelonia mydas* (Green Sea Turtle). Photographed on 6 September 2014 outside the Golden Gate, CA. Note that the turtle was briefly brought onboard to disentangle the animal from fishing lines and then was promptly released unharmed. Photo credit: Jared Davis/*The Salty Lady*.

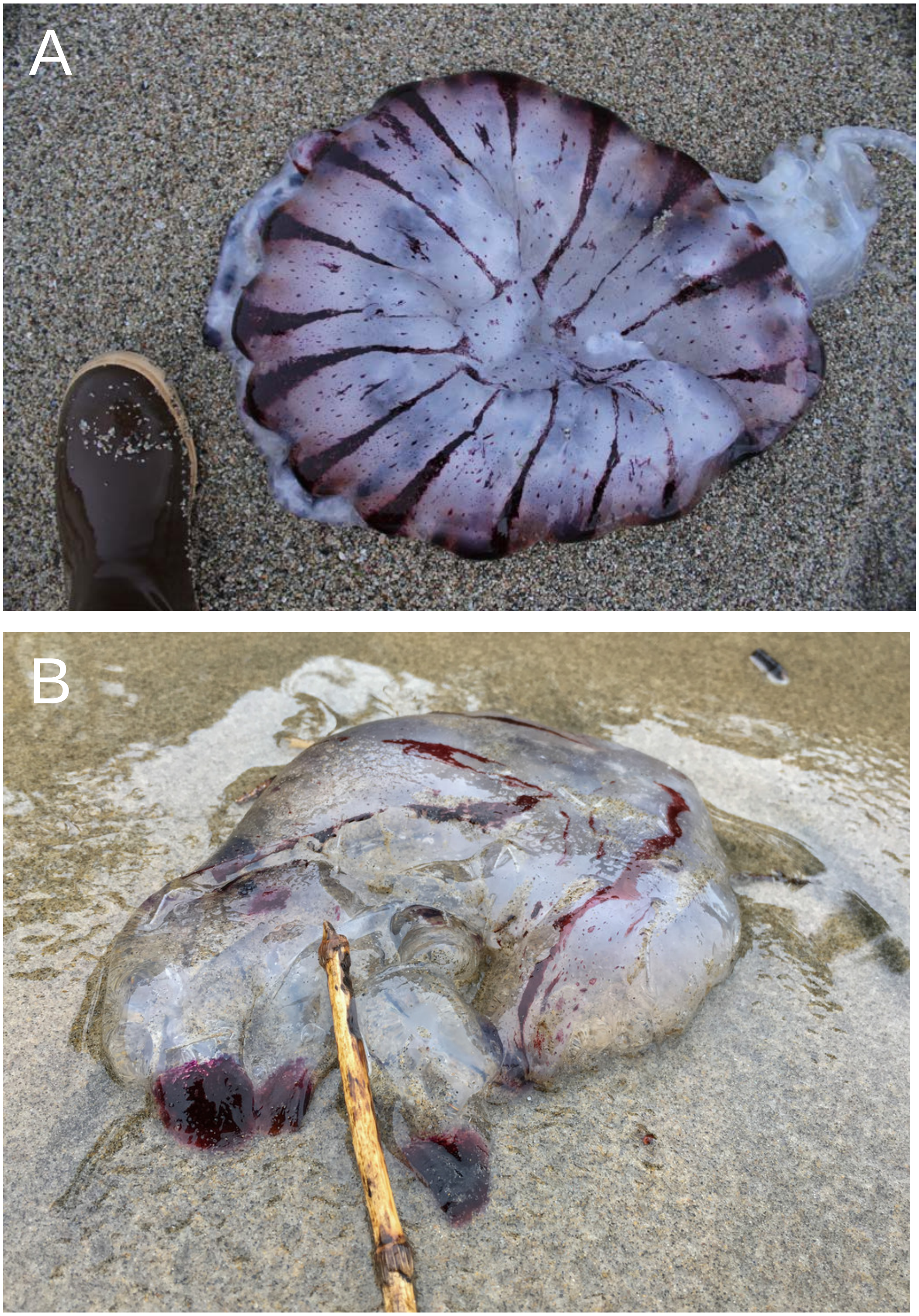

Figure S12. ***Chrysaora colorata*** **(Purple-striped Jellyfish).** **(A)** Photographed on 27 July 2014 at Horseshoe Cove, Bodega Marine Reserve, CA. Photo credit: Jacqueline Sones. **(B)** Photographed on 20 February 2017 at Arcadia Beach, Oregon. Photo credit: Steve Morey.

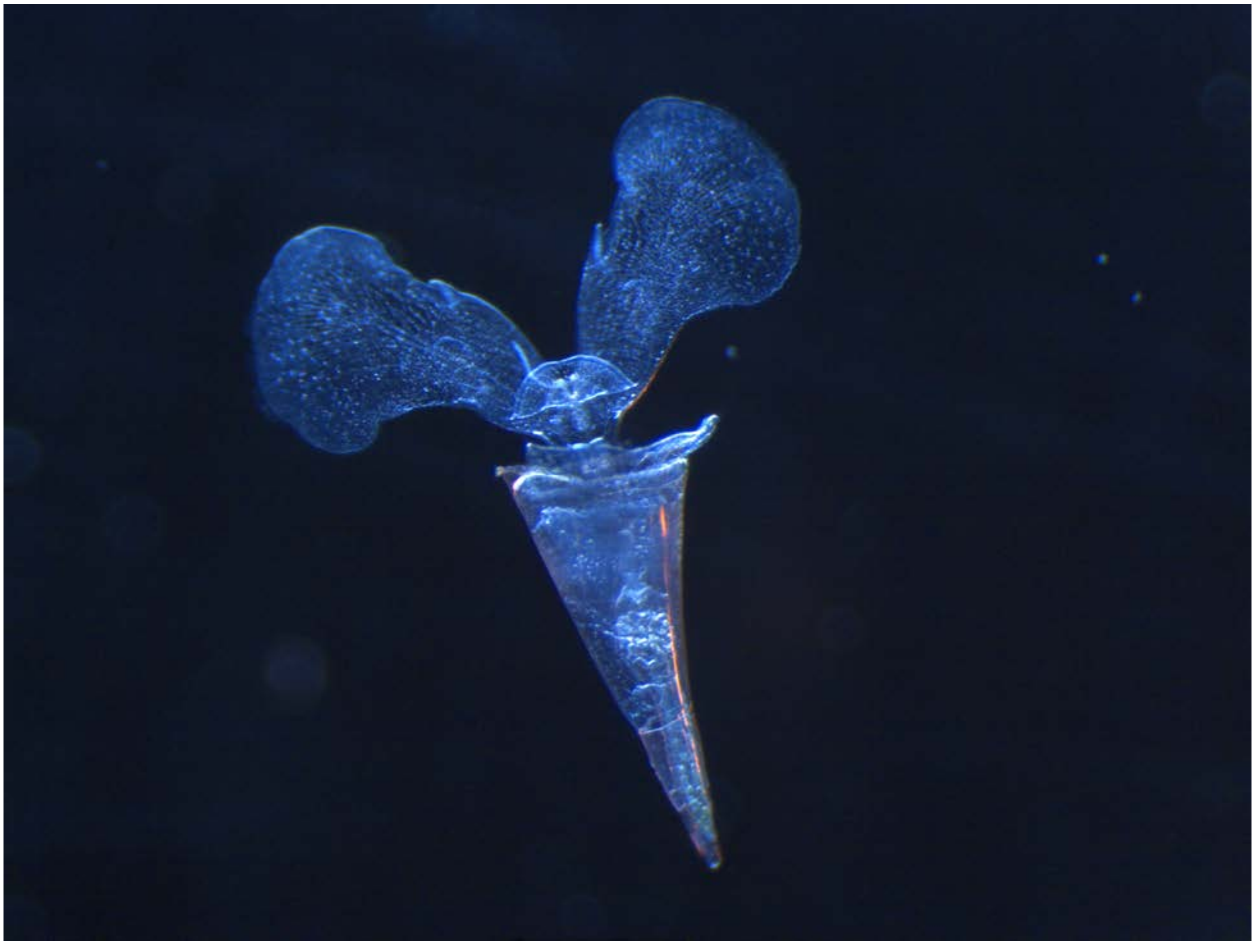

Figure S13. ***Creseis virgula*** **(Curved Needle Pteropod).** Collected on 4 November 2014 by C.V. Davis and E.B. Rivest in a plankton tow at Bodega Line Station 4, located ~32 km offshore from Bodega Marine Laboratory. Photo credit: Jacqueline Sones.

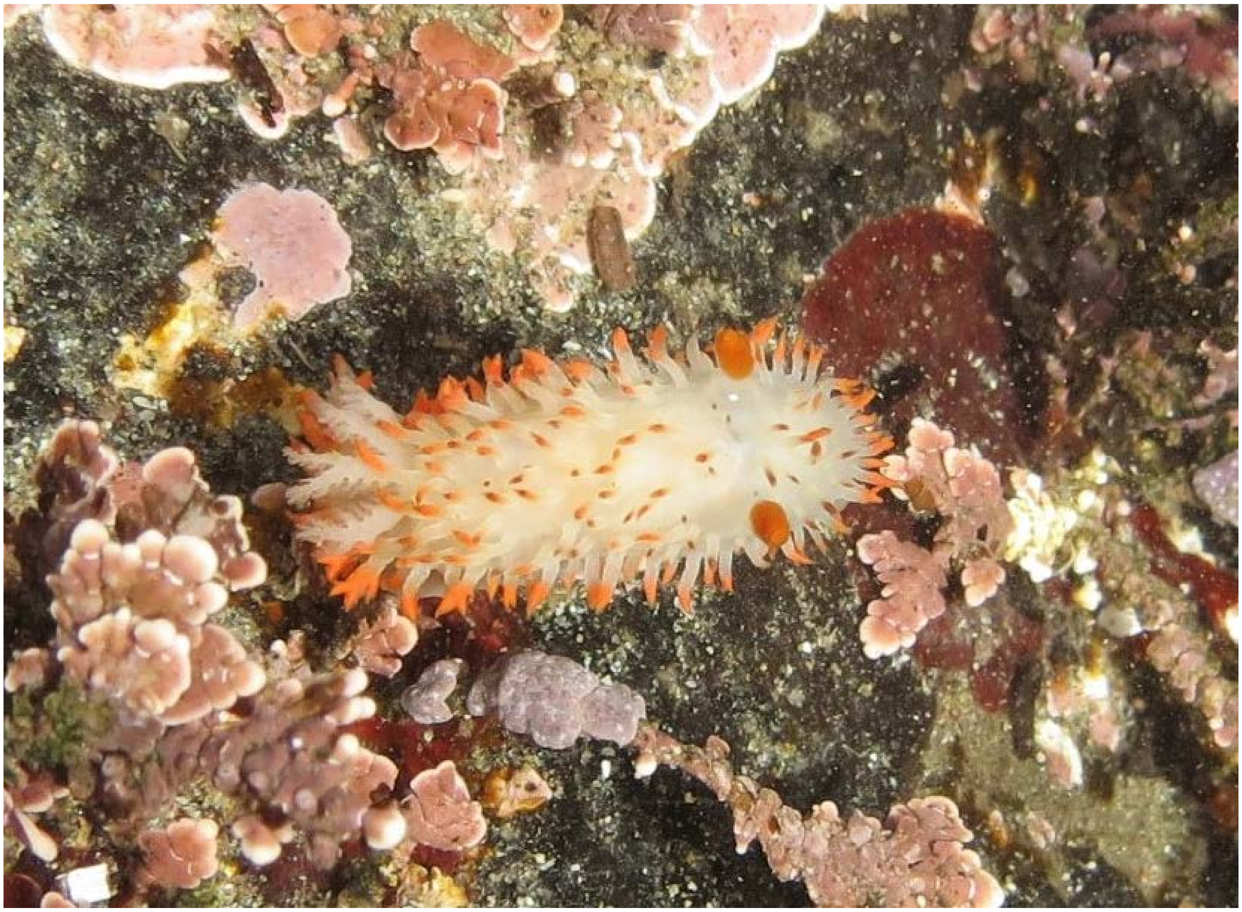

Figure S14. ***Crimora coneja*** **(Rabbit Dorid Nudibranch).** Photographed on 29 April 2017 at Boiler Bay, Oregon. See Goddard et al. 2018. Photo credit: Phil Nosler.

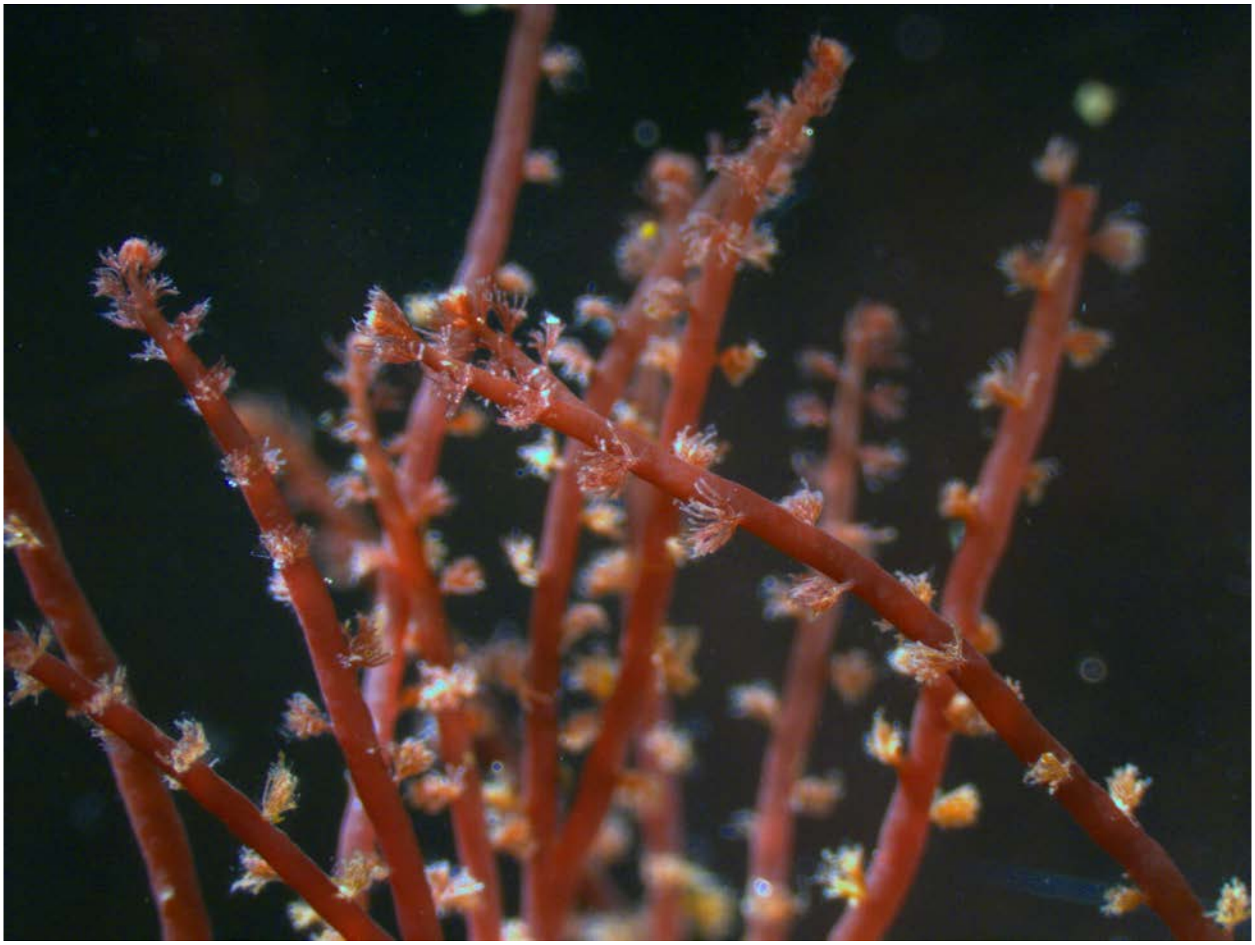

Figure S15. *Dasya binghamiae* (Red Alga). Collected on 18 June 2015 by Peter G. Connors from the South Jetty at the Bodega Harbor entrance, CA. Specimen deposited at University Herbarium, UC Berkeley, UC2056114. Photo credit: Jacqueline Sones.

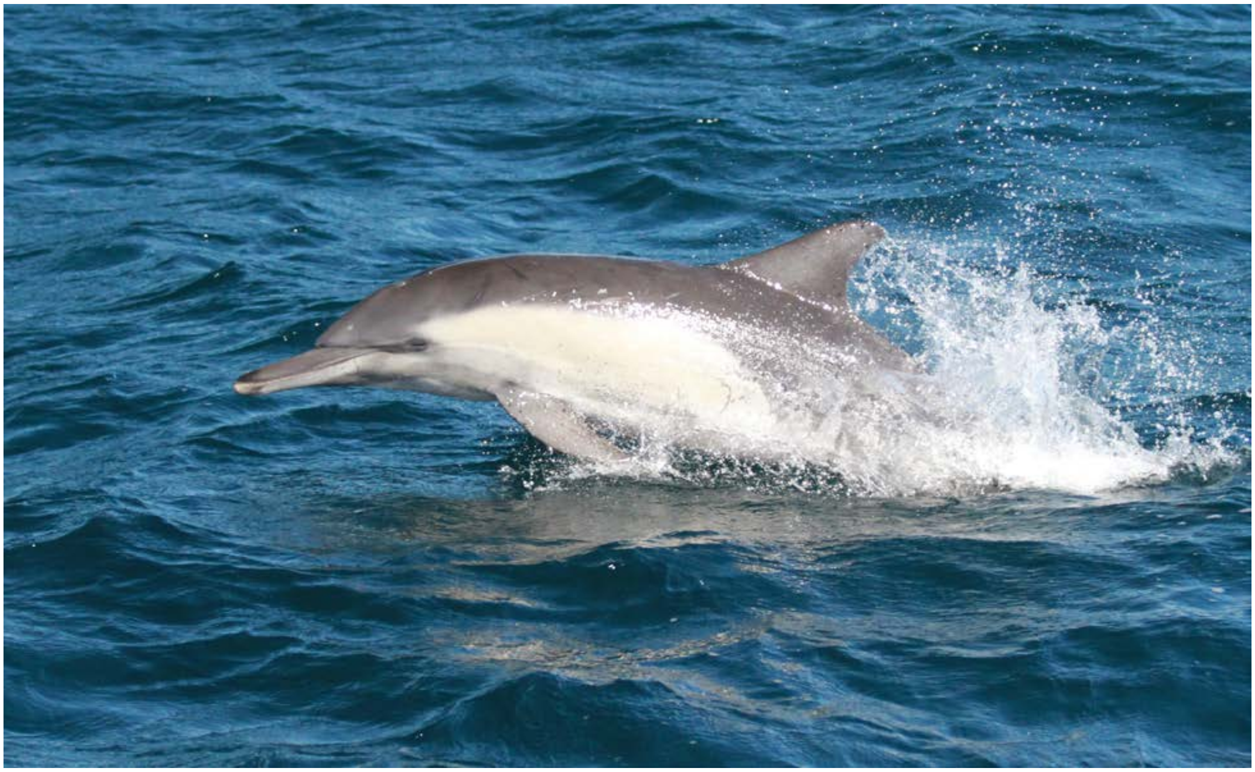

Figure S16. *Delphinus capensis* (Long-beaked Common Dolphin). Photographed on 18 September 2015 near Cordell Bank. Photo credit: Jacqueline Sones.

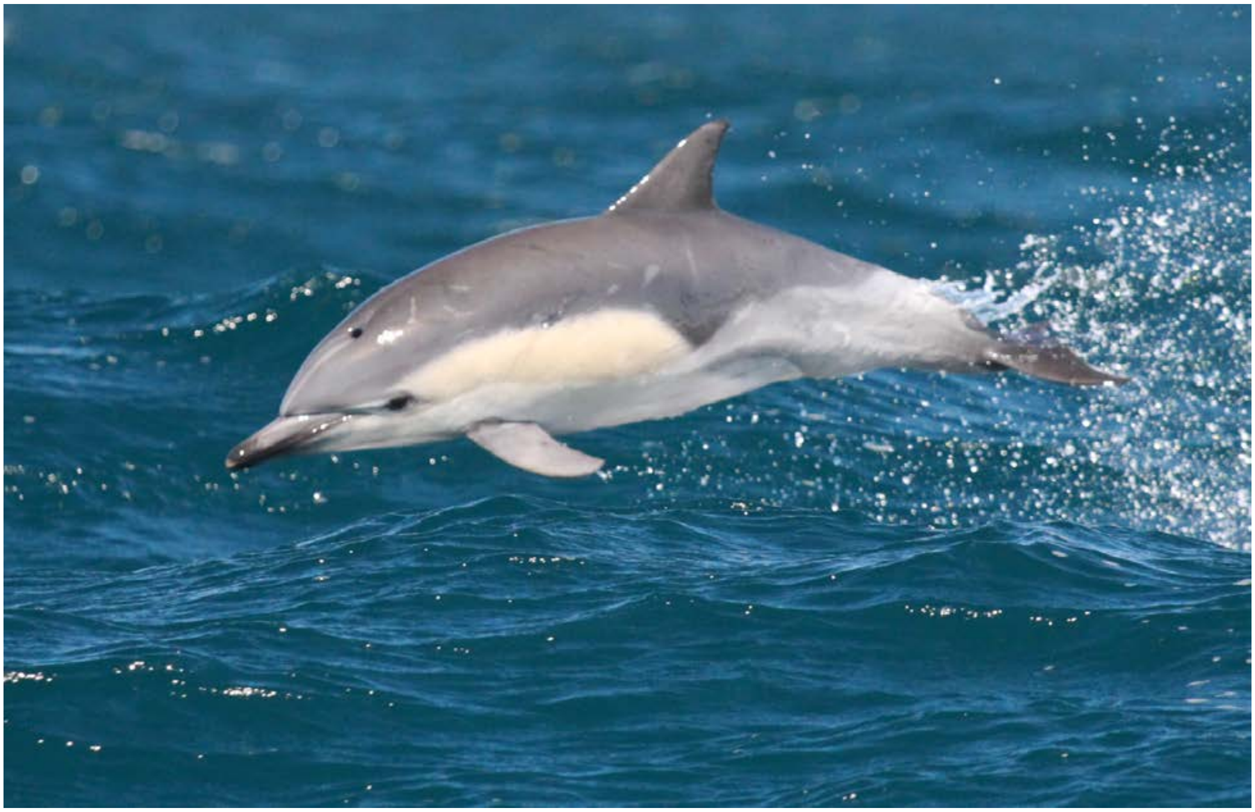

Figure S17. *Delphinus delphis* (Short-beaked Common Dolphin). Photographed on 18 September 2015 at Cordell Bank. Photo credit: Jacqueline Sones.

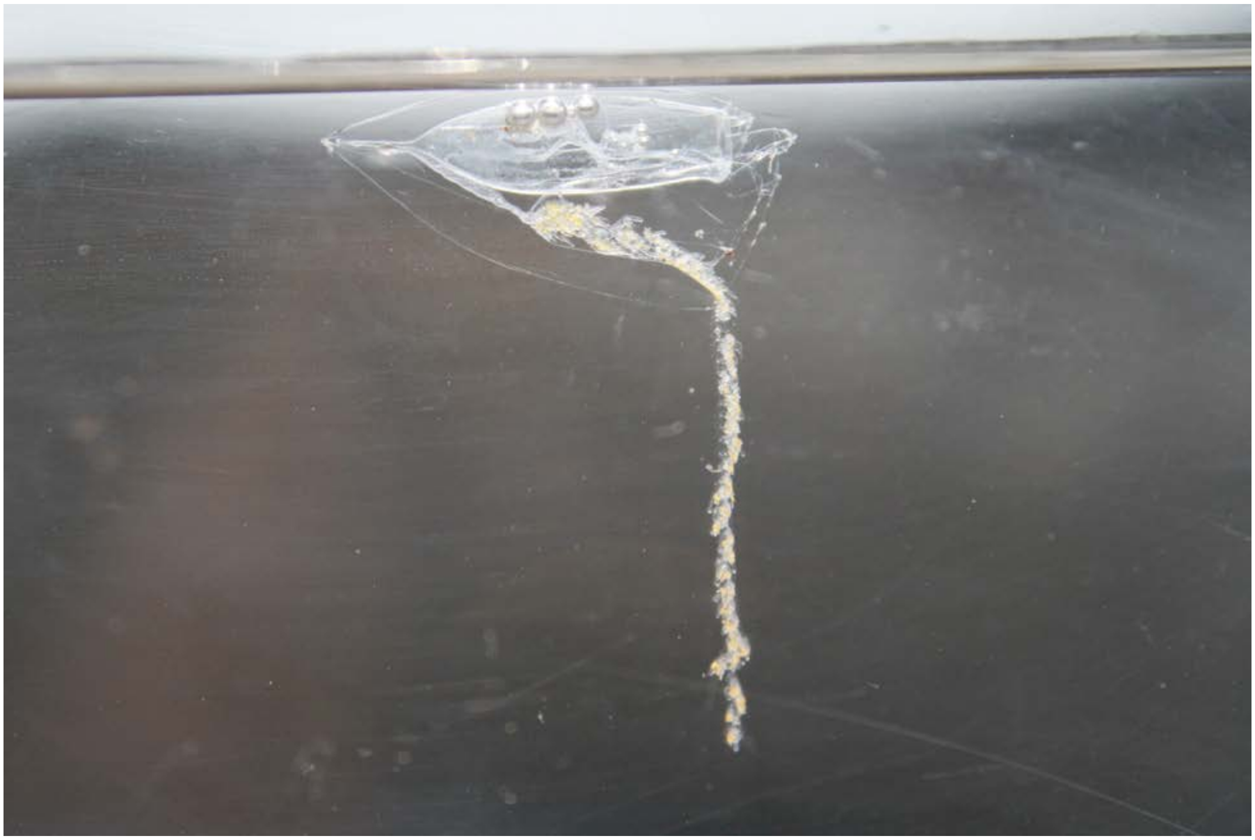

Figure S18. ***Diphyes dispar*** **(Siphonophore).** Collected on 14 September 2014 from Salmon Creek Beach, CA. Photo credit: Jacqueline Sones.

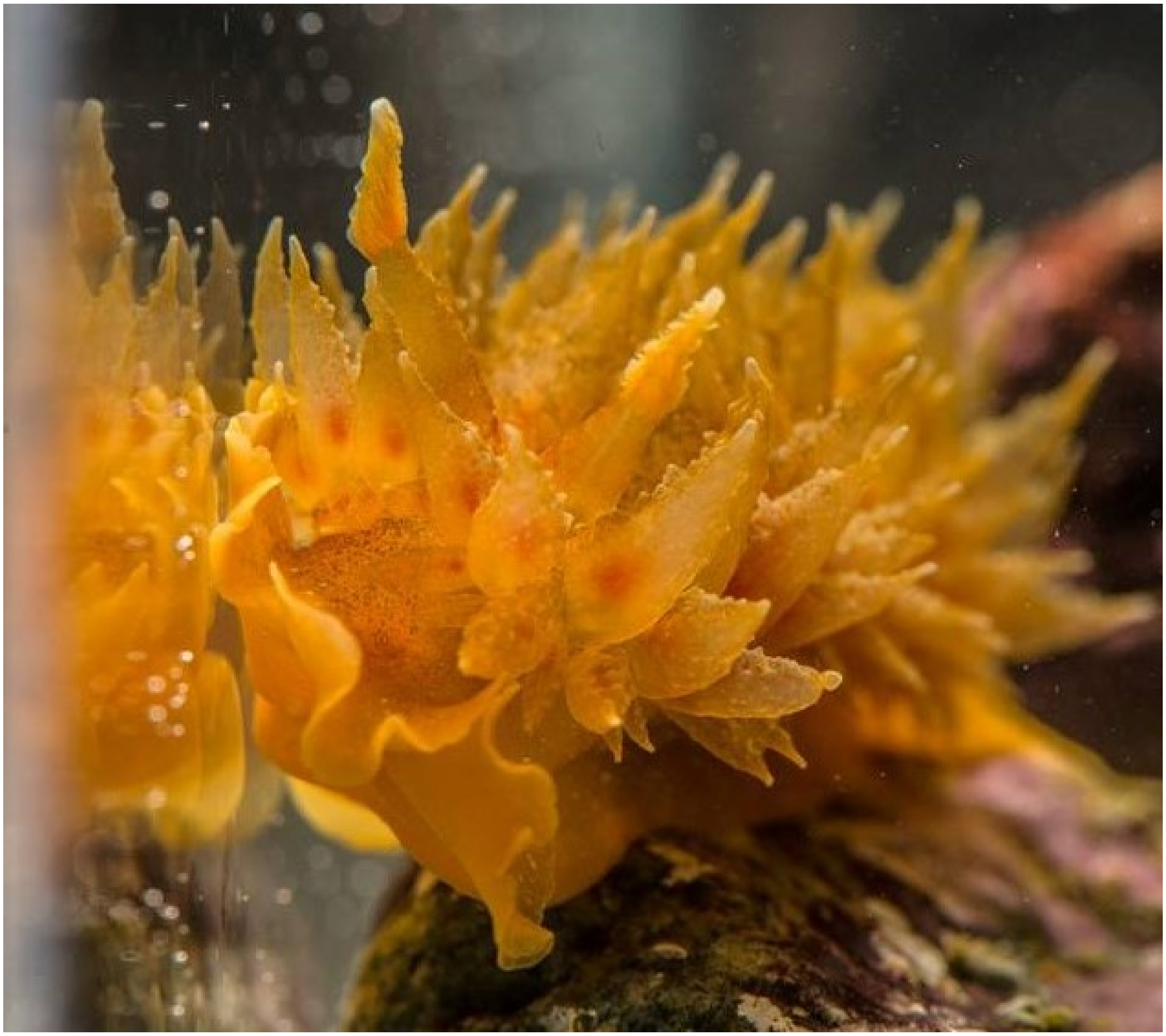

Figure S19. ***Dirona picta*** **(Colorful Dirona Nudibranch).** Photographed on 6 July 2016 in Barkley Sound, Vancouver Island, British Columbia, Canada. Photo credit: Chad Tamis.

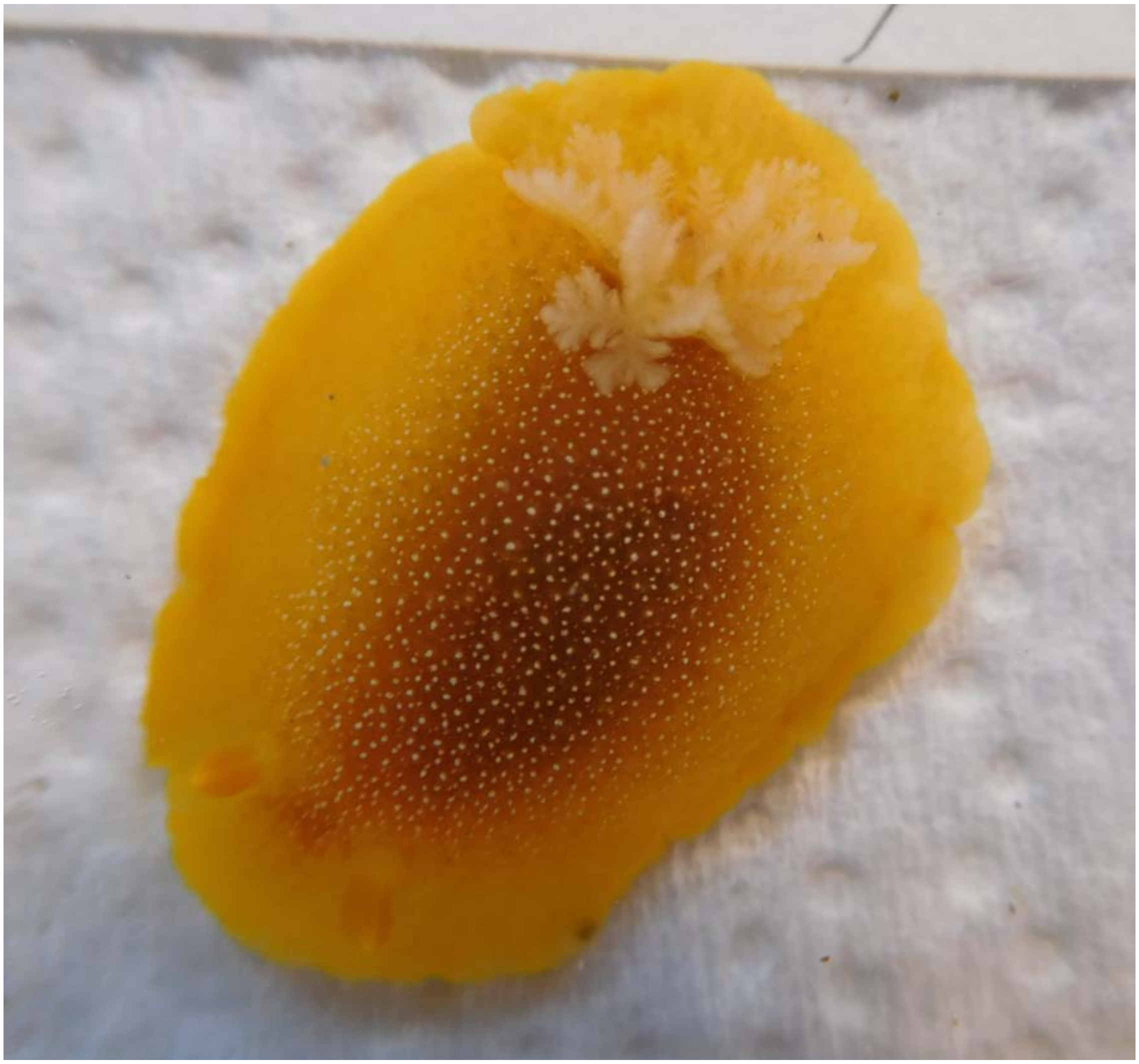

Figure S20. *Doriopsilla albopunctata* (White-spotted Sea Goddess Nudibranch). Photographed on 29 May 2017 at Whiskey Creek, Oregon. See Goddard et al. 2018. Photo credit: Nancy Treneman.

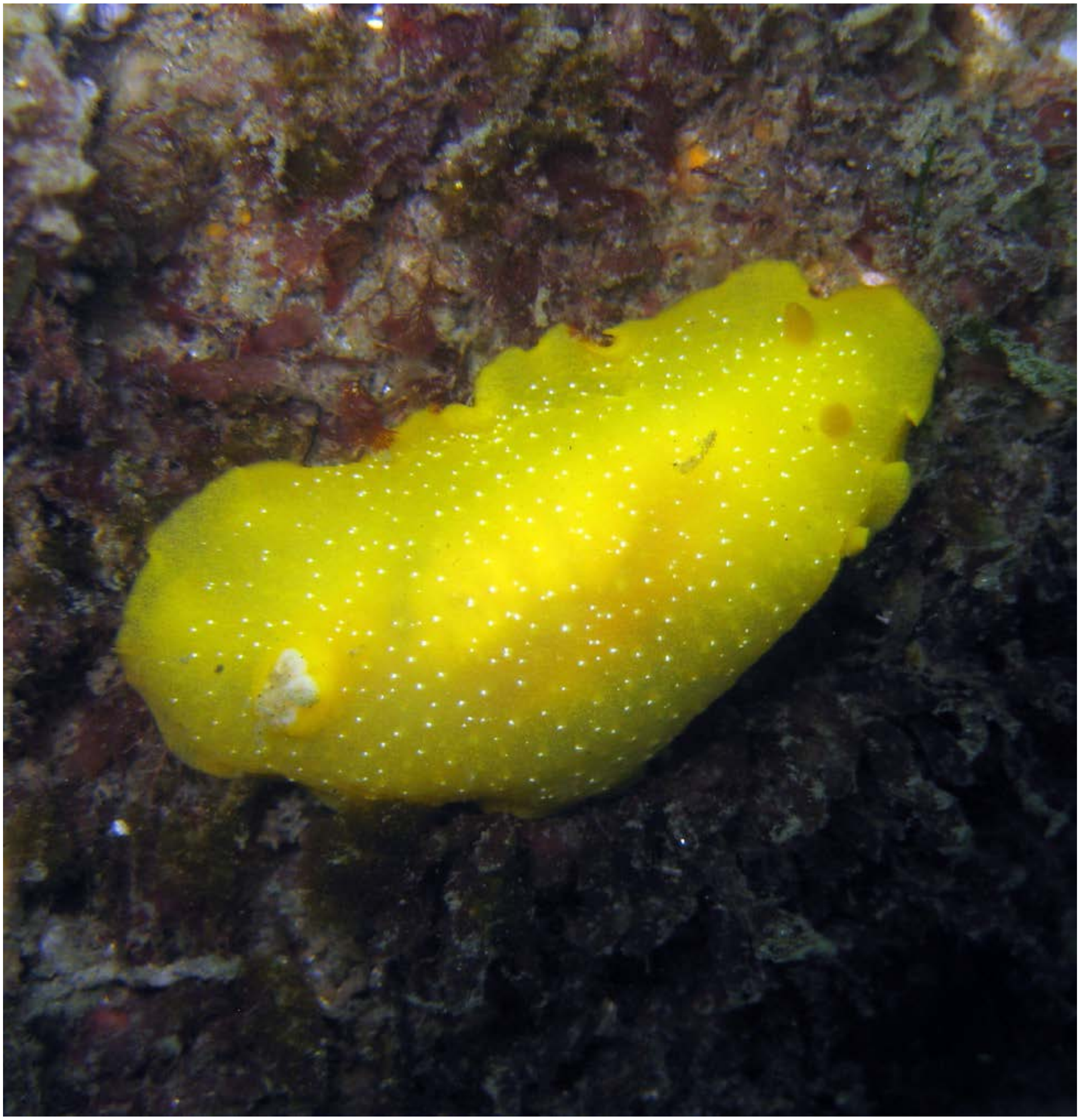

Figure S21. ***Doriopsilla fulva*** **(White-spotted Dorid Nudibranch).** Photographed on 16 July 2016 at Netarts Bay, Oregon. See Goddard et al. 2018. Photo credit: Todd Cliff.

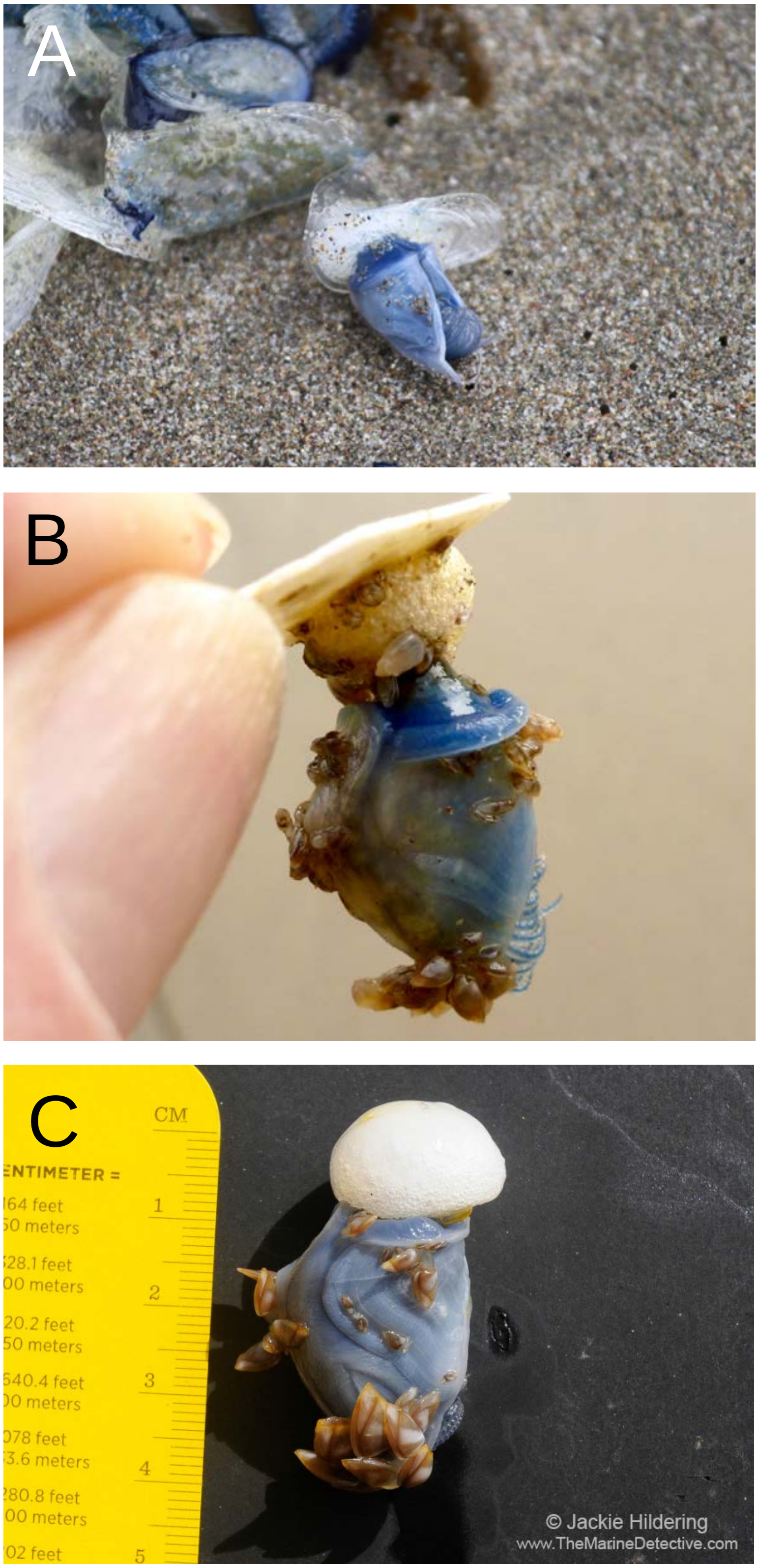

Figure S22. ***Dosima fascicularis*** **(Blue Buoy Barnacle).** **(A)** Photographed on 28 May 2015 on Salmon Creek Beach, CA. Photo credit: Jacqueline Sones. **(B)** Photographed on 26 March 2016 on Arcadia Beach, Oregon. Photo credit: Steve Morey. **(C)** Collected on 11 July 2014 from about 65 km offshore from the southern end of Moresby Island, British Columbia, Canada. Photo credit: © Jackie Hildering.

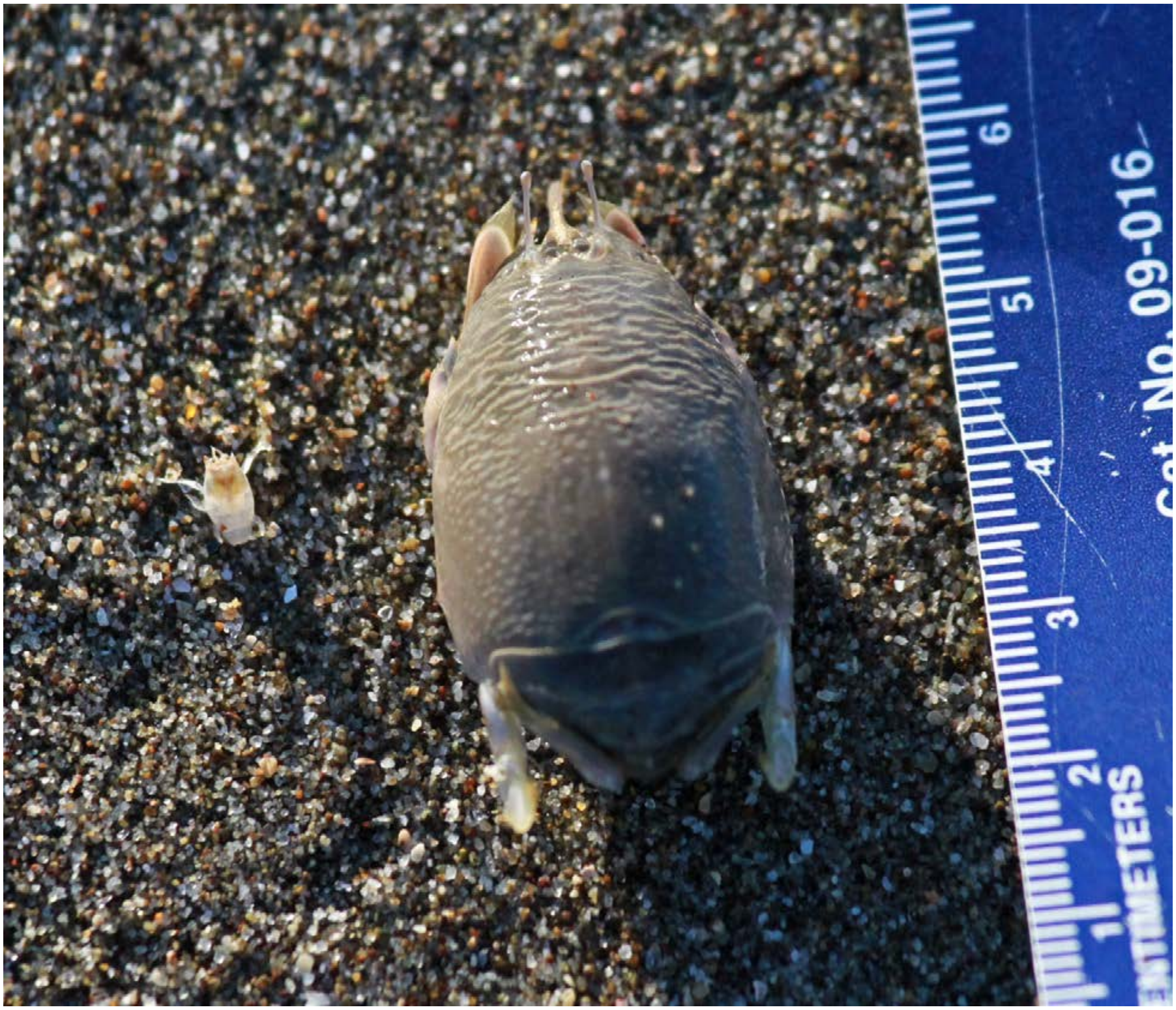

Figure S23. ***Emerita analoga* (Mole Crab).** Juvenile (left) and adult (right) mole crabs photographed on 1 March 2015 at Salmon Creek Beach, CA. Photo credit: Jacqueline Sones.

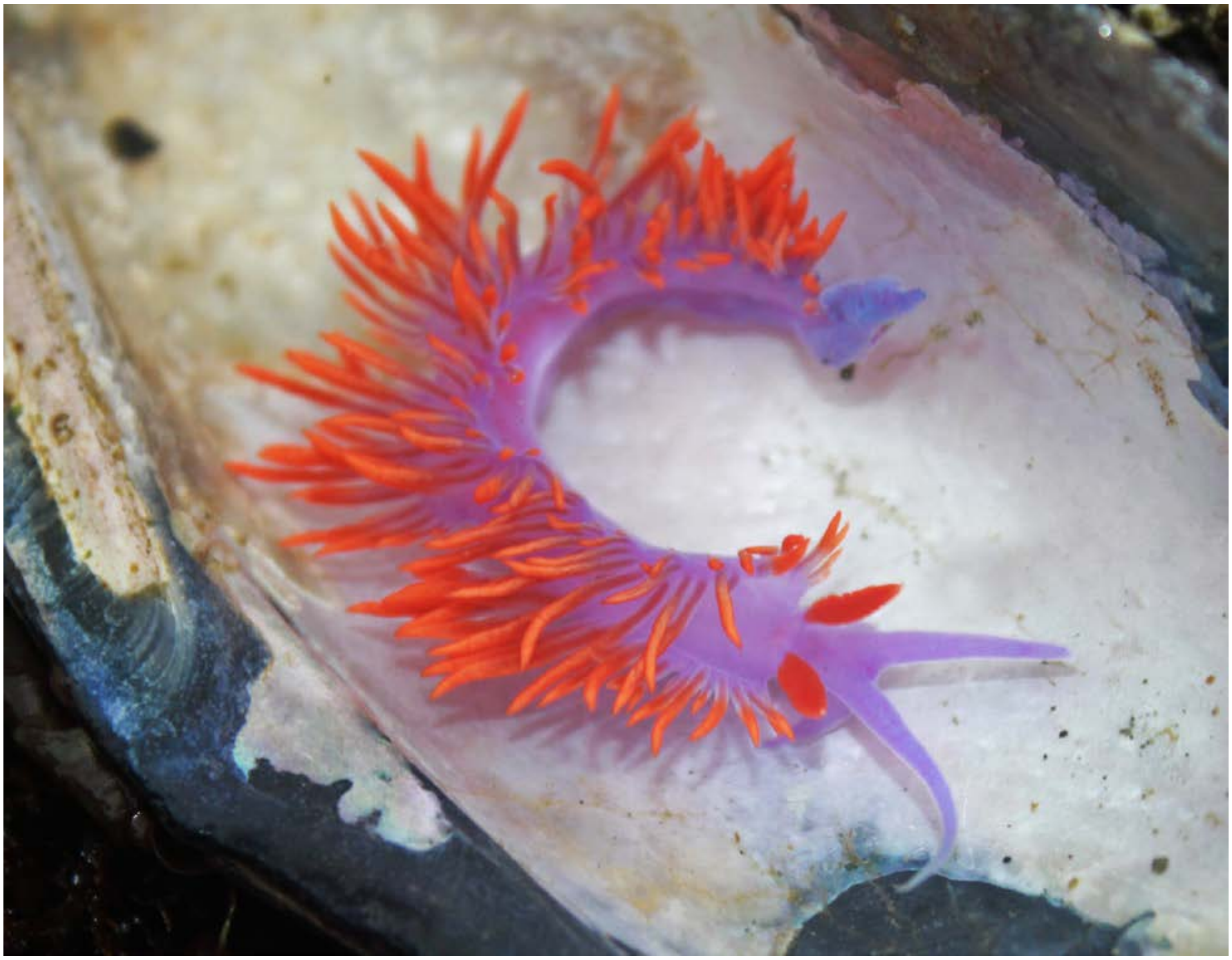

Figure S24. *Flabellinopsis iodinea* (Spanish Shawl Nudibranch). Photographed on 18 May 2015 at Pinnacle Gulch, CA. Photo credit: Jacqueline Sones.

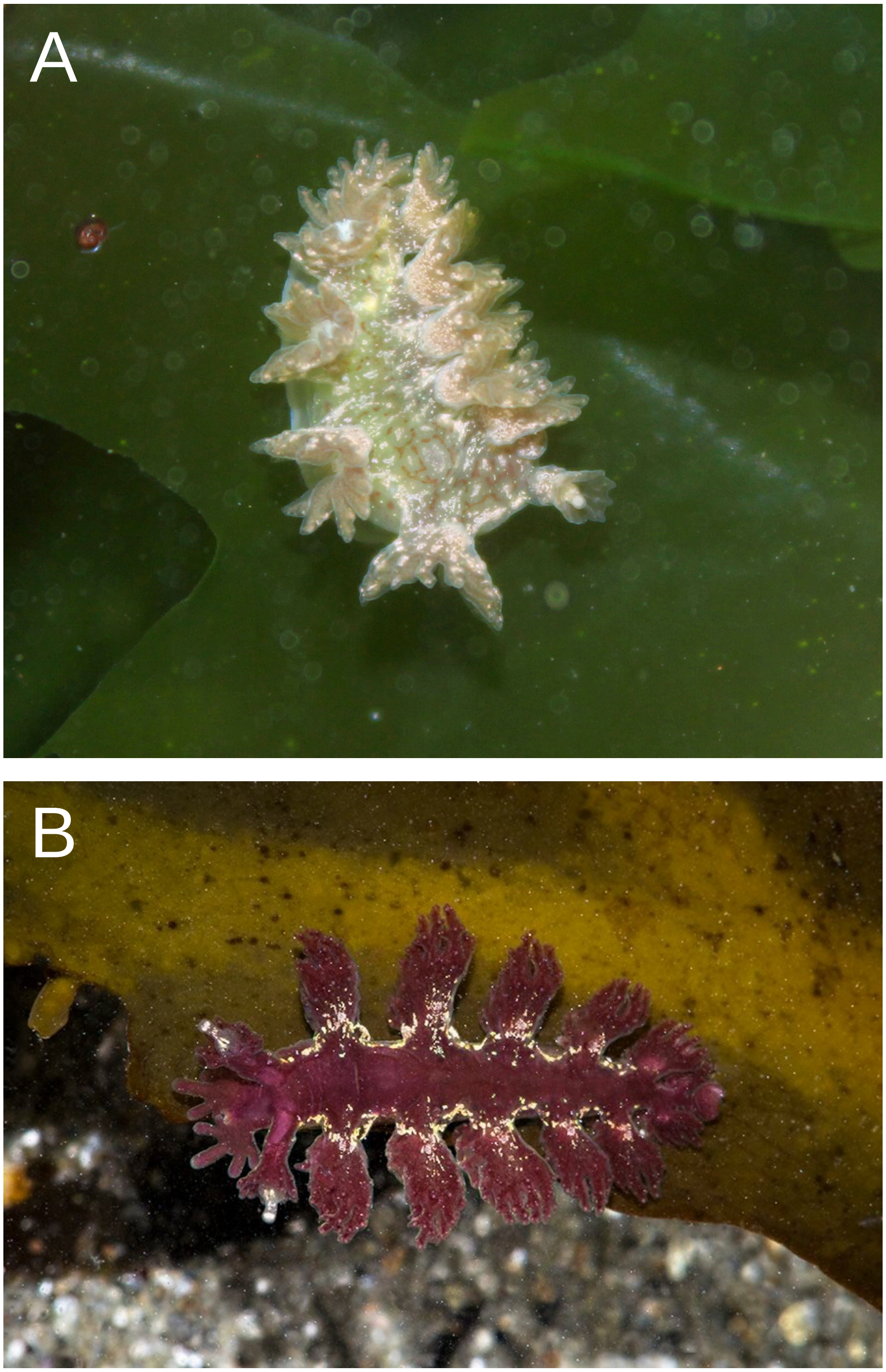

Figure S25. ***Hancockia californica*** **(Hancock's Nudibranch). (A)** Photographed on 23 May 2015 at Coleman Beach, CA. Photo credit: Jacqueline Sones. **(B)** Photographed on 29 August 2015 at Trinidad, CA. Photo credit: Natalie McNear.

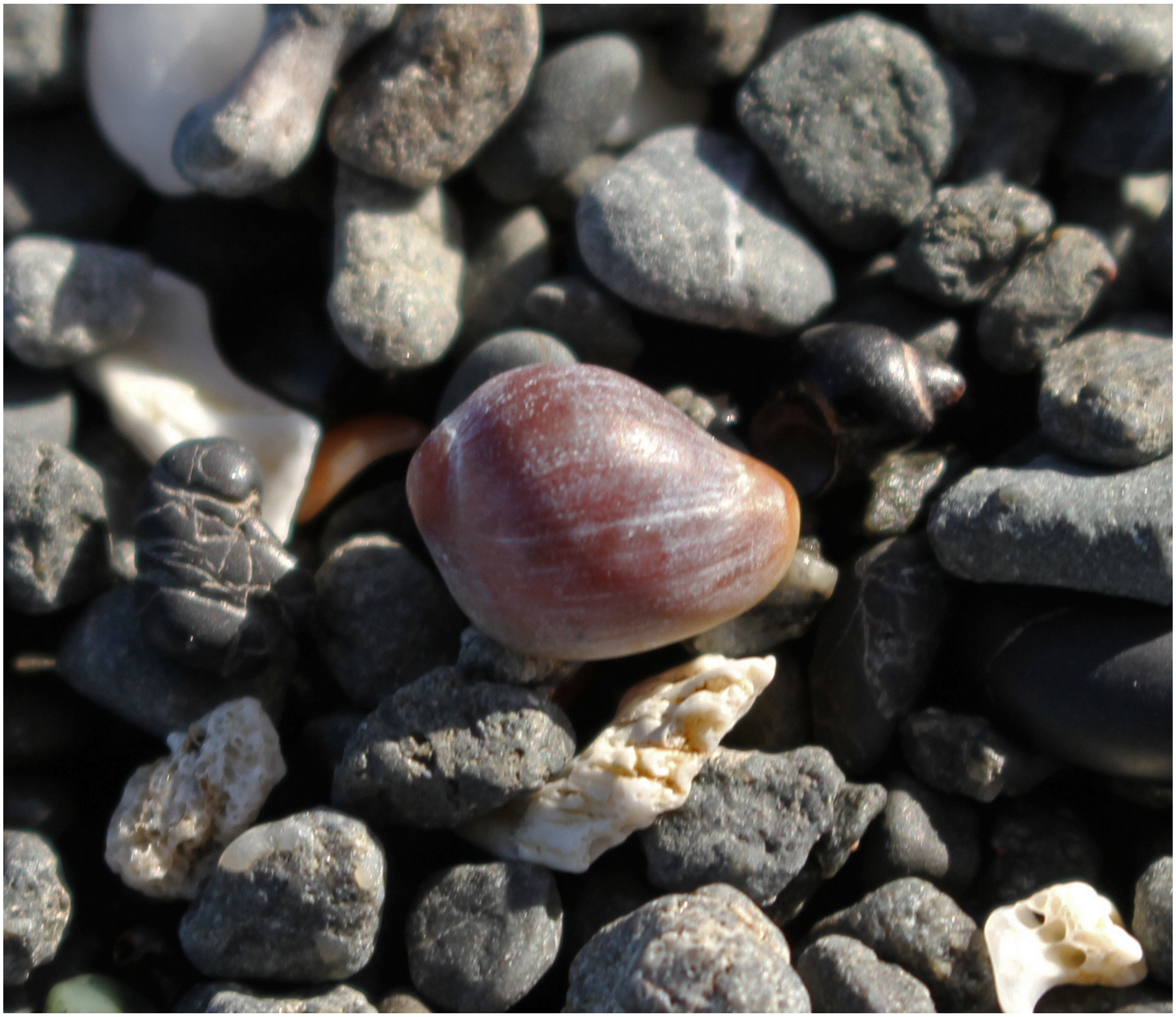

Figure S26. *Hespererato vitellina* (Appleseed Erato Snail). Photographed on 25 May 2017 at Cape Mendocino, CA. Specimen deposited at California Academy of Sciences (CASIZ 224108). Photo credit: Jacqueline Sones.

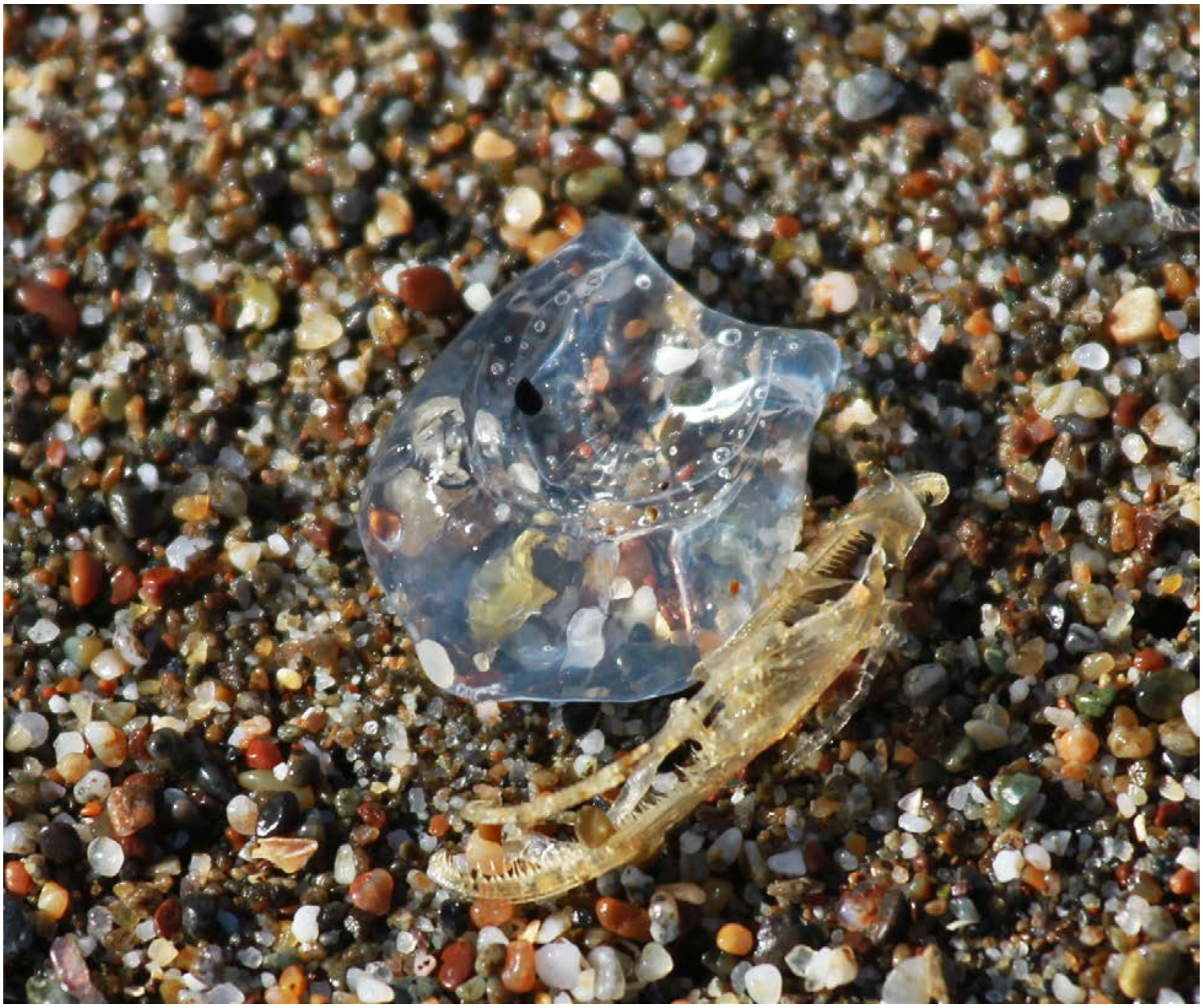

Figure S27. *Hippopodius hippopus* (Siphonophore). Distinctive horseshoe-shaped nectophore of *Hippopodius hippopus*, photographed on 22 March 2016 at Salmon Creek Beach, CA. [Note there is a brown barnacle molt at the lower right of the nectophore.] Photo credit: Jacqueline Sones.

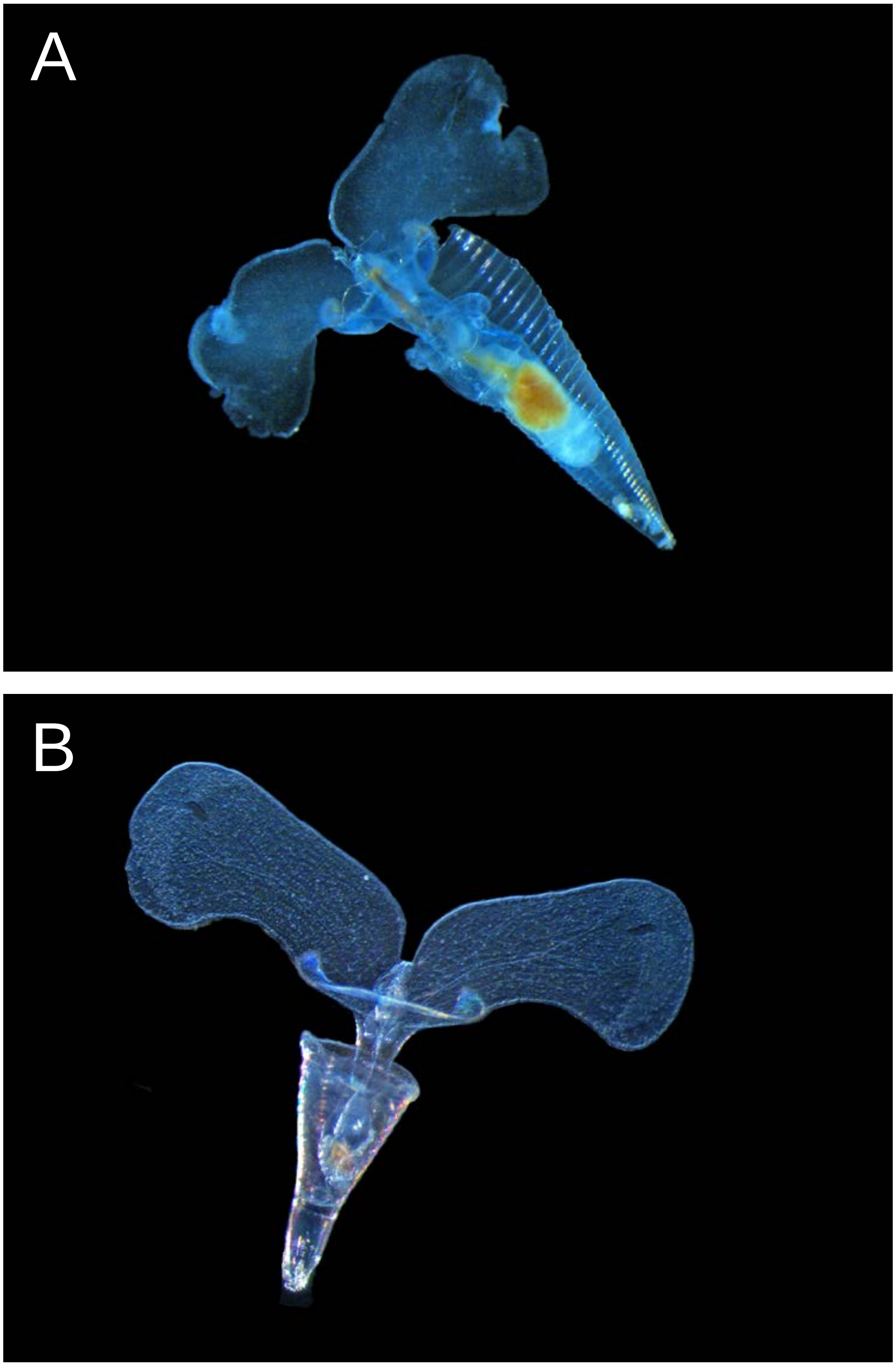

Figure S28. ***Hyalocylis striata*** **(Striated Sea Butterfly). (A)** Collected on 24 September 2014 at Bodega Canyon. Specimen deposited at California Academy of Sciences (CASIZ 202355). Photo credit: Jacqueline Sones. **(B)** Specimen (CASIZ 202356) collected on 21 October 2014 at the Bodega Marine Laboratory oceanographic buoy (located 1.2 km offshore from the laboratory). Photo credit: Eric Sanford.

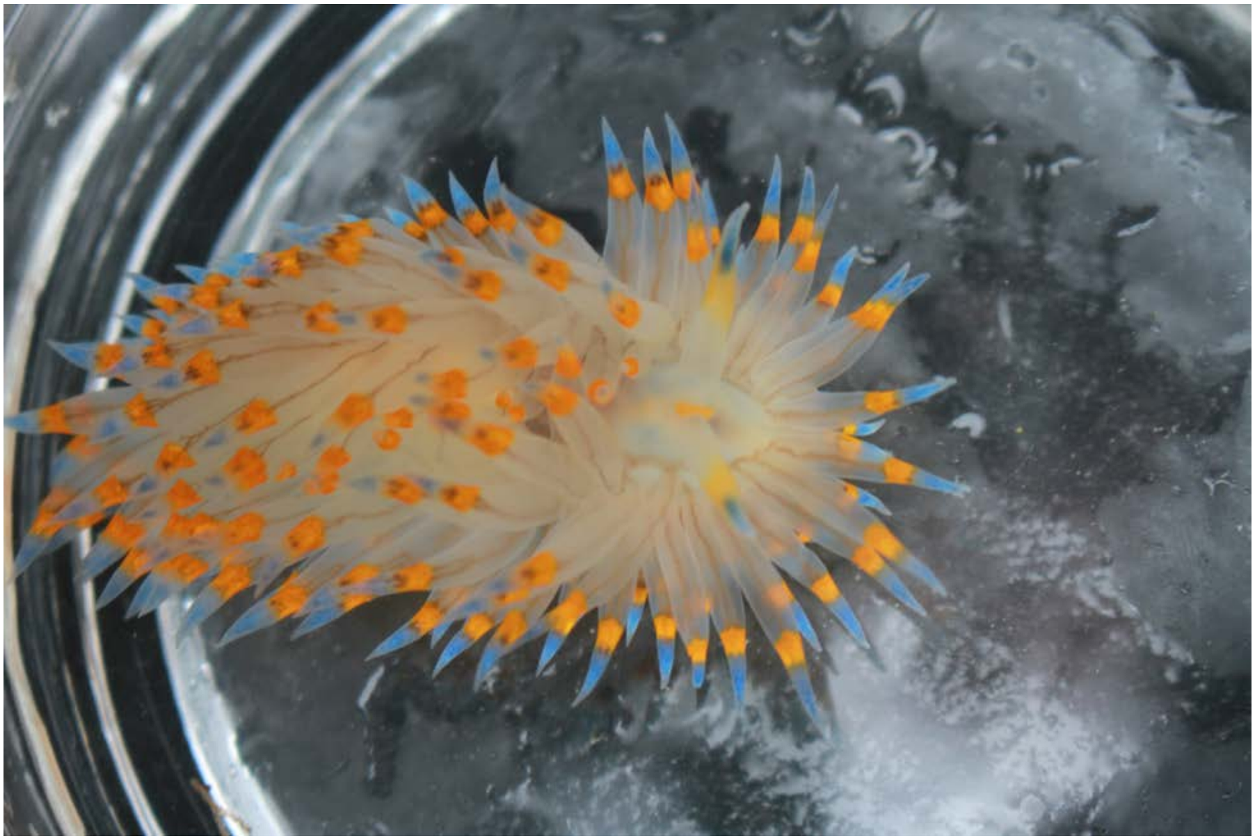

Figure S29. ***Janolus barbarensis*** **(Janolus Nudibanch).** Photographed in Bodega Harbor, CA, on 12 September 2015. Photo credit: Jacqueline Sones.

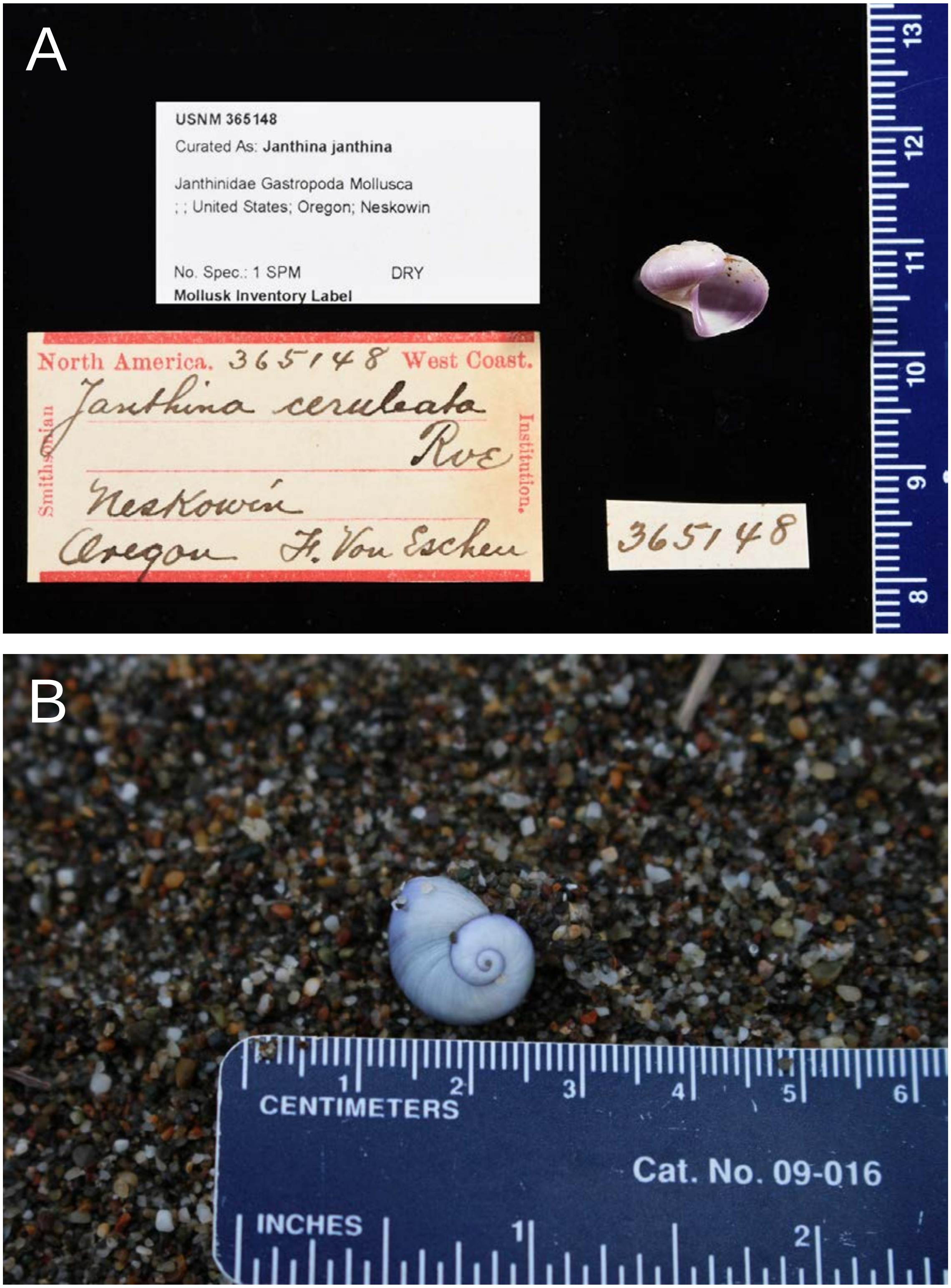

Figure S30. ***Janthina janthina*** **(Violet Sea Snail).** **(A)** Collected at Neskowin, Oregon, by Florian Von Eschen and deposited at the United States National Museum in July 1926 (USNM 365148). Photo credit: Christopher Meyer. **(B)** Photographed on Salmon Creek Beach, CA, on 12 March 2016. Photo credit: Jacqueline Sones.

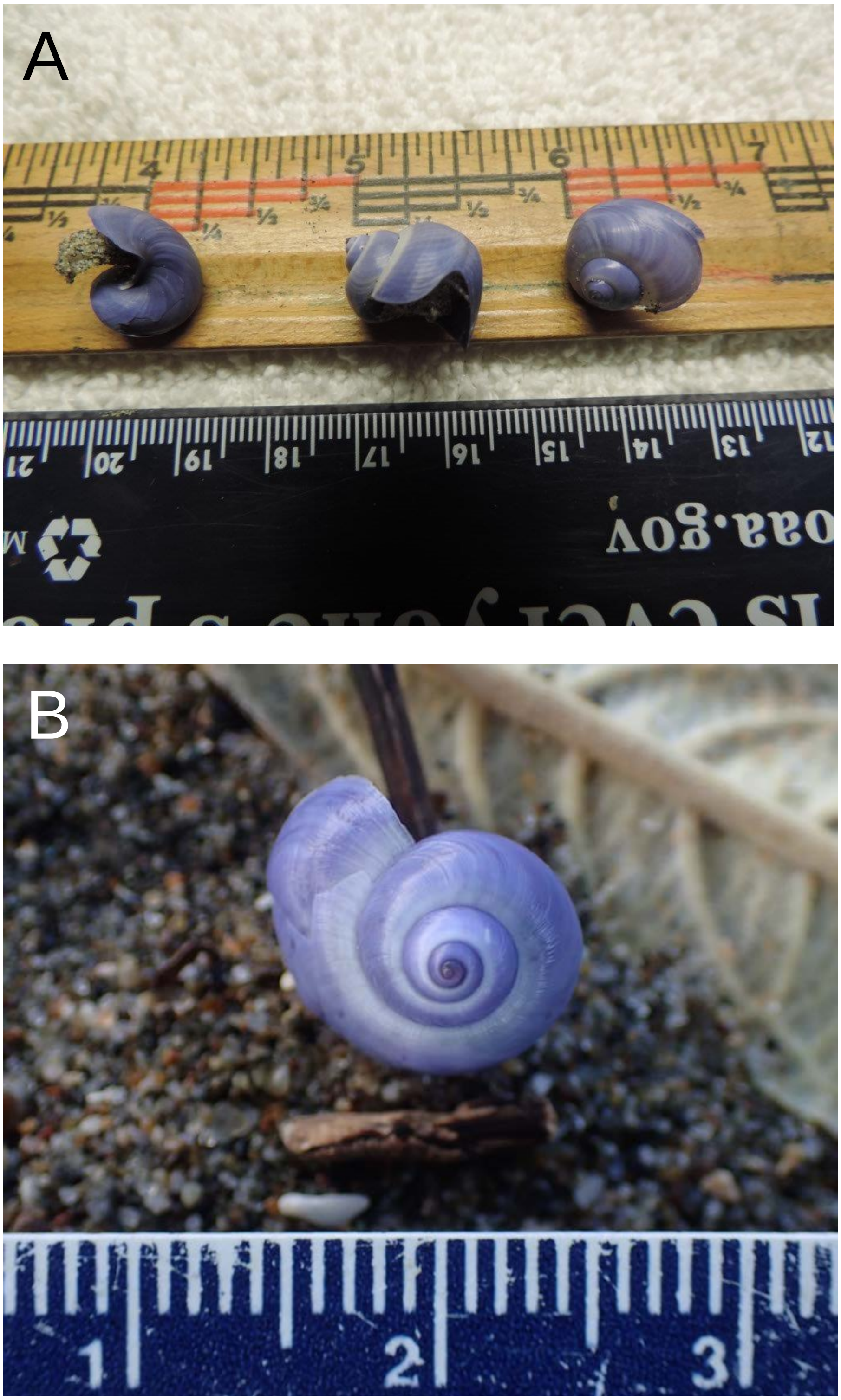

Figure S31. ***Janthina umbilicata*** **(Purple Sea Snail). (A)** Collected at Leadbetter Point, Washington, on 21–22 November 2015. Specimens deposited at California Academy of Sciences (CASIZ 227279). Photo credit: Russ Lewis. **(B)** Photographed on Salmon Creek Beach, CA, on 25 January 2018. Photo credit: Jacqueline Sones.

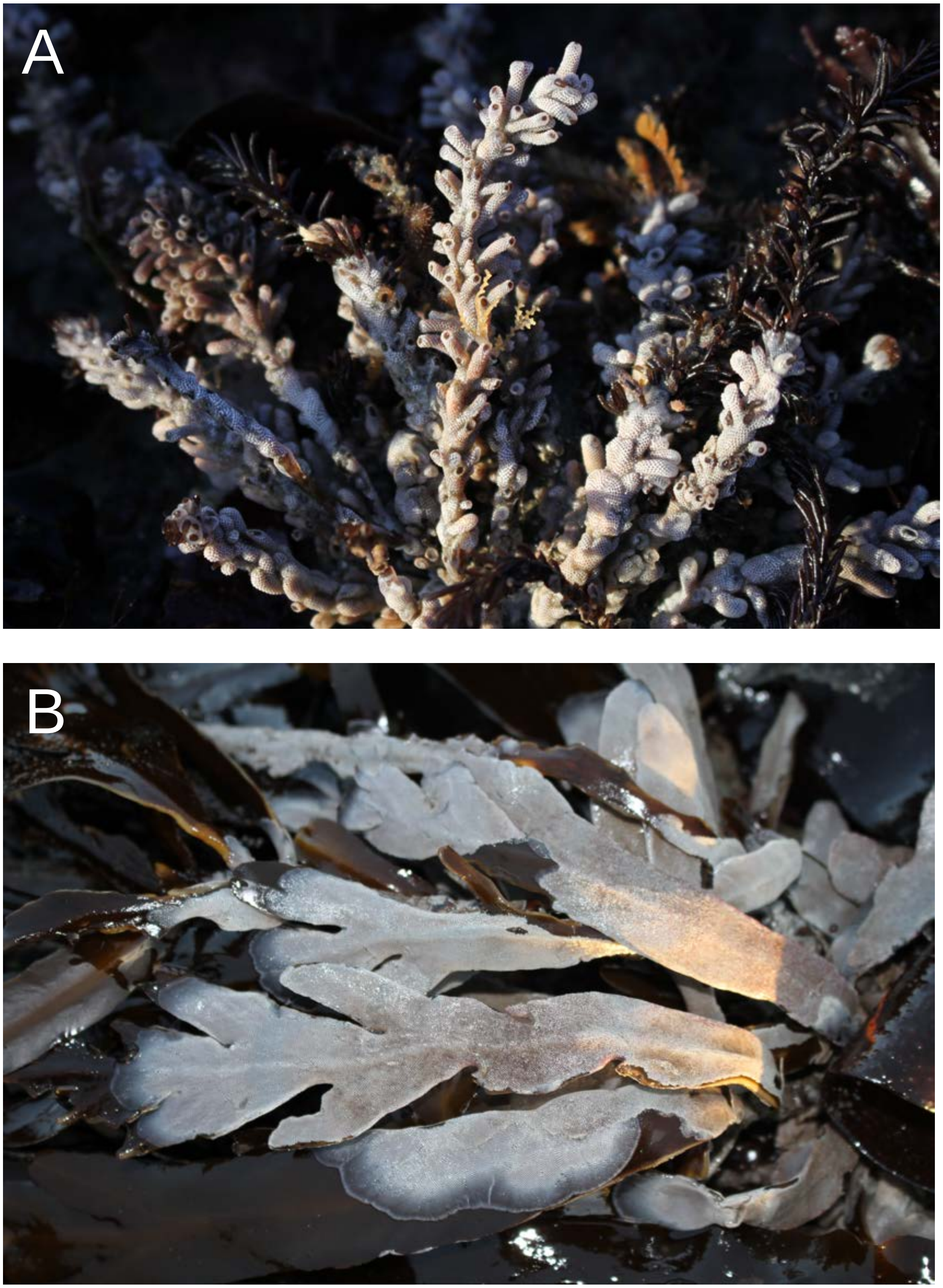

Figure S32. *Jellyella tuberculata* (Bryozoan). Photographed growing on low intertidal algae at Pinnacle Gulch, CA, on 14 November 2016. Photo credit: Jacqueline Sones.

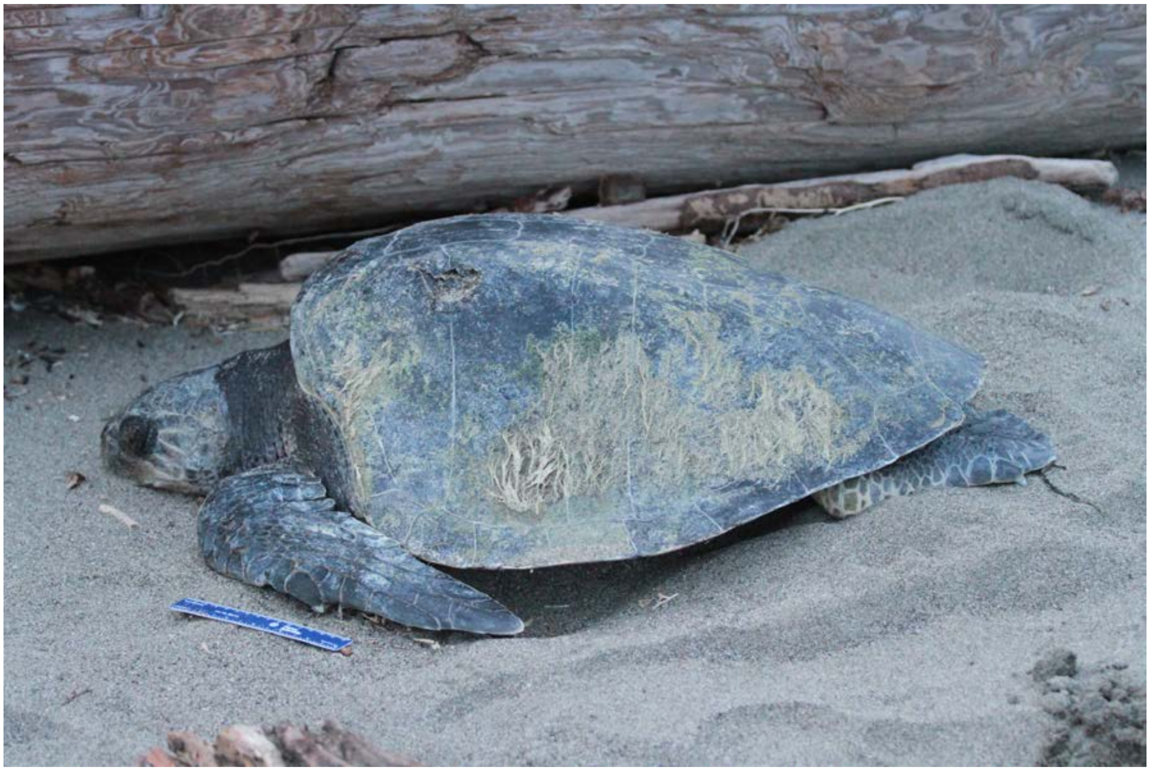

Figure S33. ***Lepidochelys olivacea*** **(Olive Ridley Sea Turtle).** Photographed on Salmon Creek Beach, CA, on 15 January 2016. Photo credit: Jacqueline Sones.

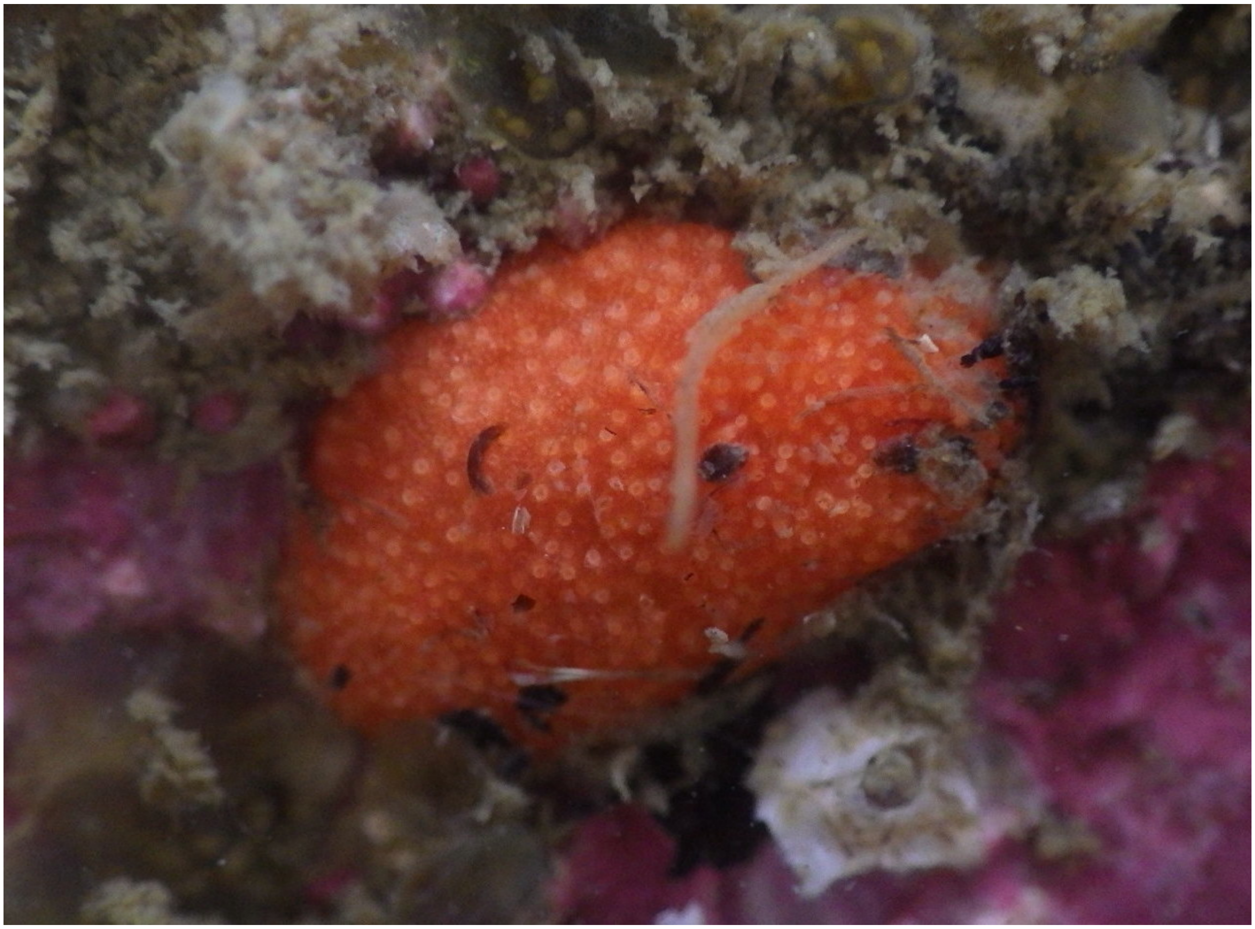

Figure S34. *Lissothuria nutriens* (Scarlet Sea Cucumber). Photographed at Fort Ross Reef, CA, on 25 June 2017. Photo credit: Jeff Goddard.

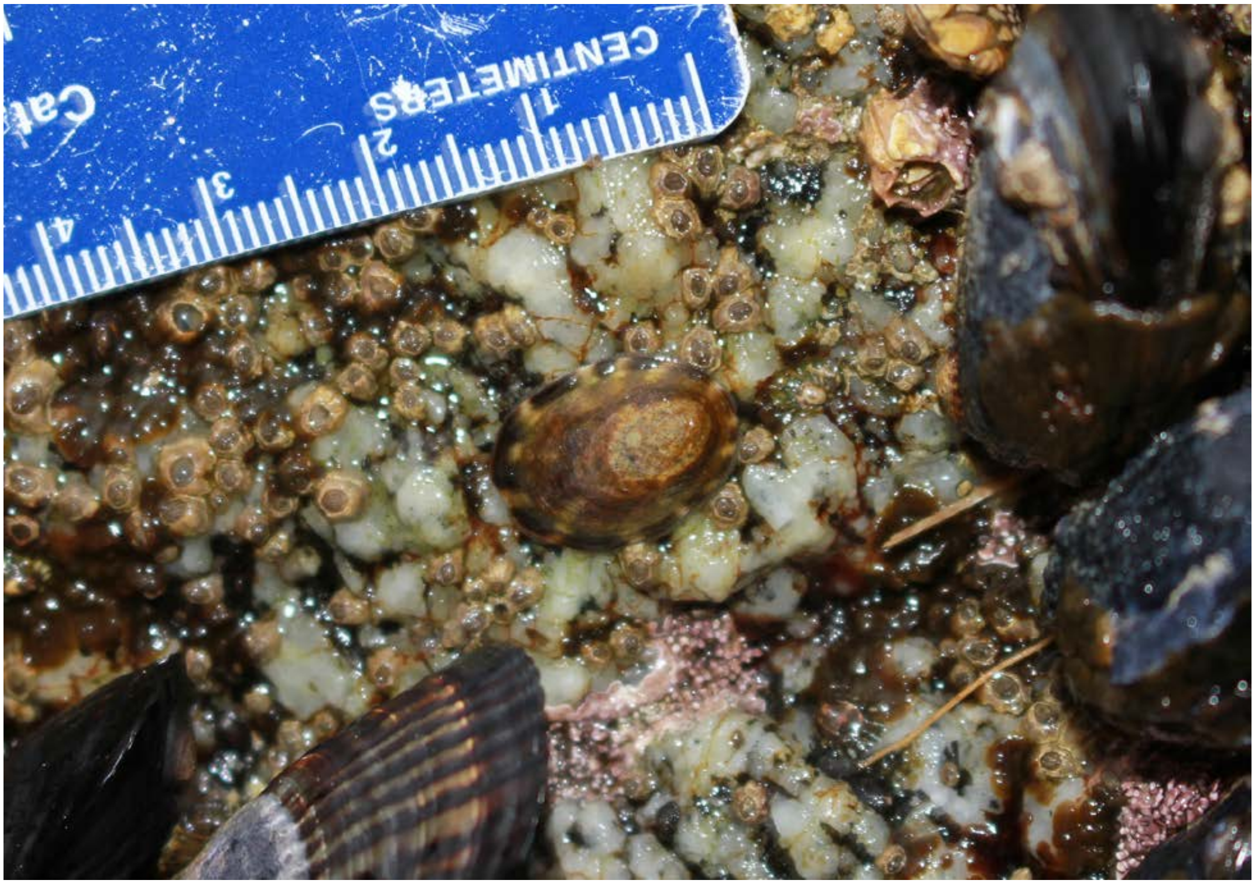

Figure S35. *Lottia gigantea* (Owl Limpet). Juvenile photographed at Bodega Marine Reserve, CA, on 6 June 2016. Photo credit: Jacqueline Sones.

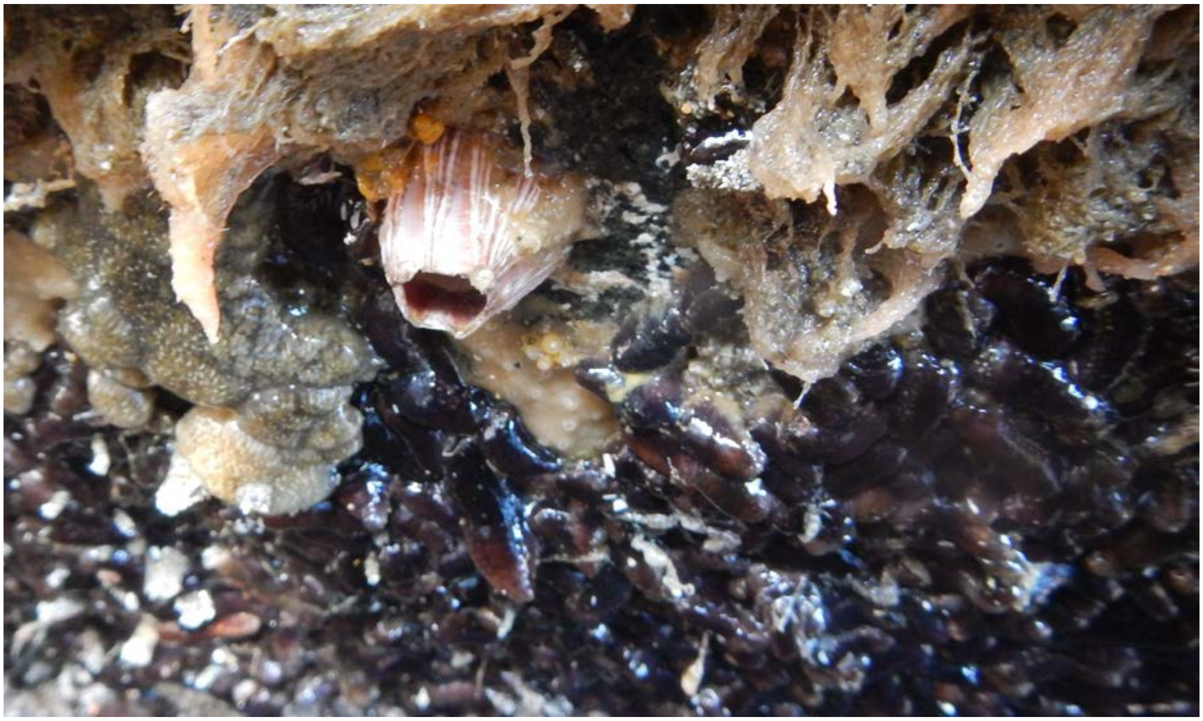

Figure S36. *Megabalanus californicus* (Pink-striped Barnacle). Photographed at Humbug State Park, Oregon, on 5 June 2015. Photo credit: Nancy Treneman.

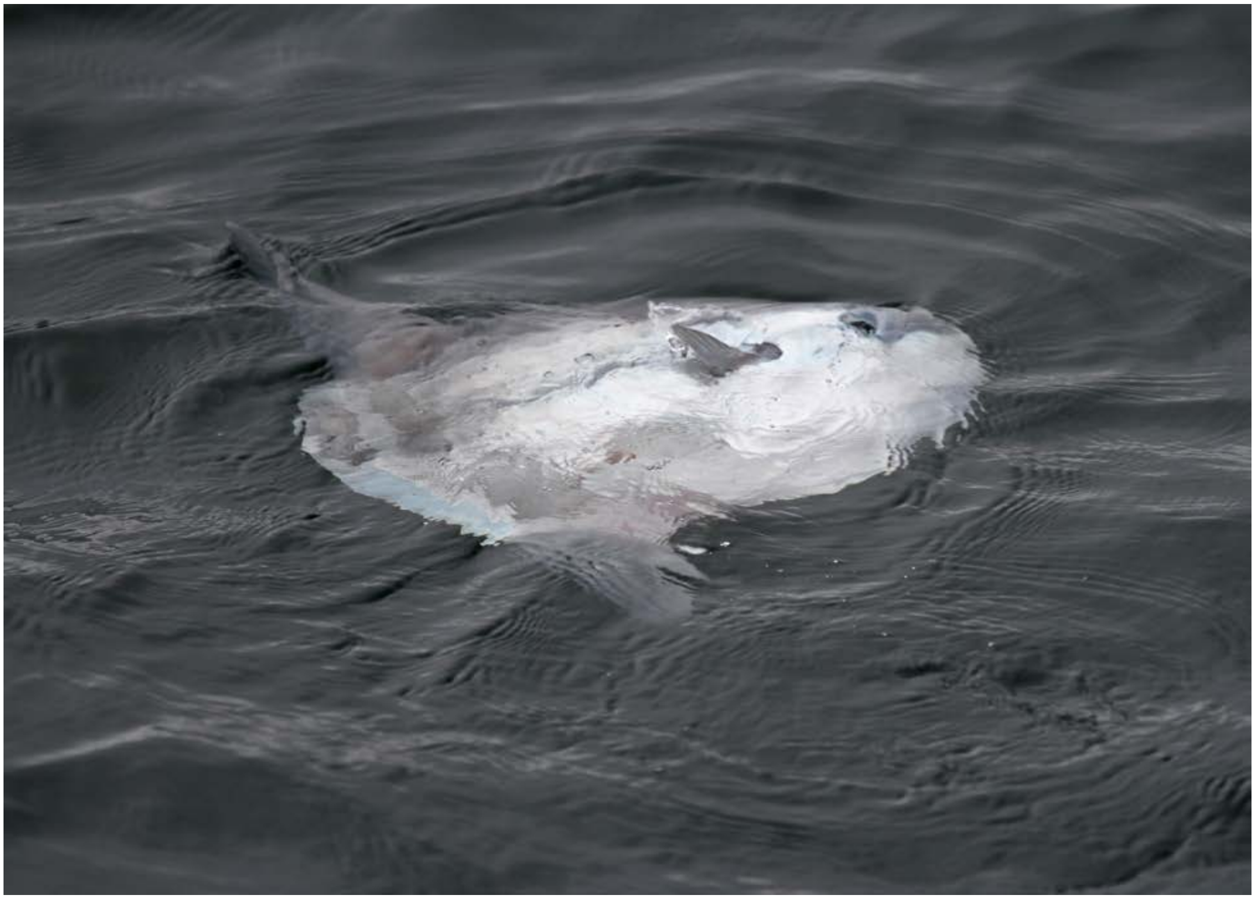

Figure S37. ***Mola mola*** **(Ocean Sunfish).** Photographed near Cordell Bank on 7 September 2014. Photo credit: Jacqueline Sones.

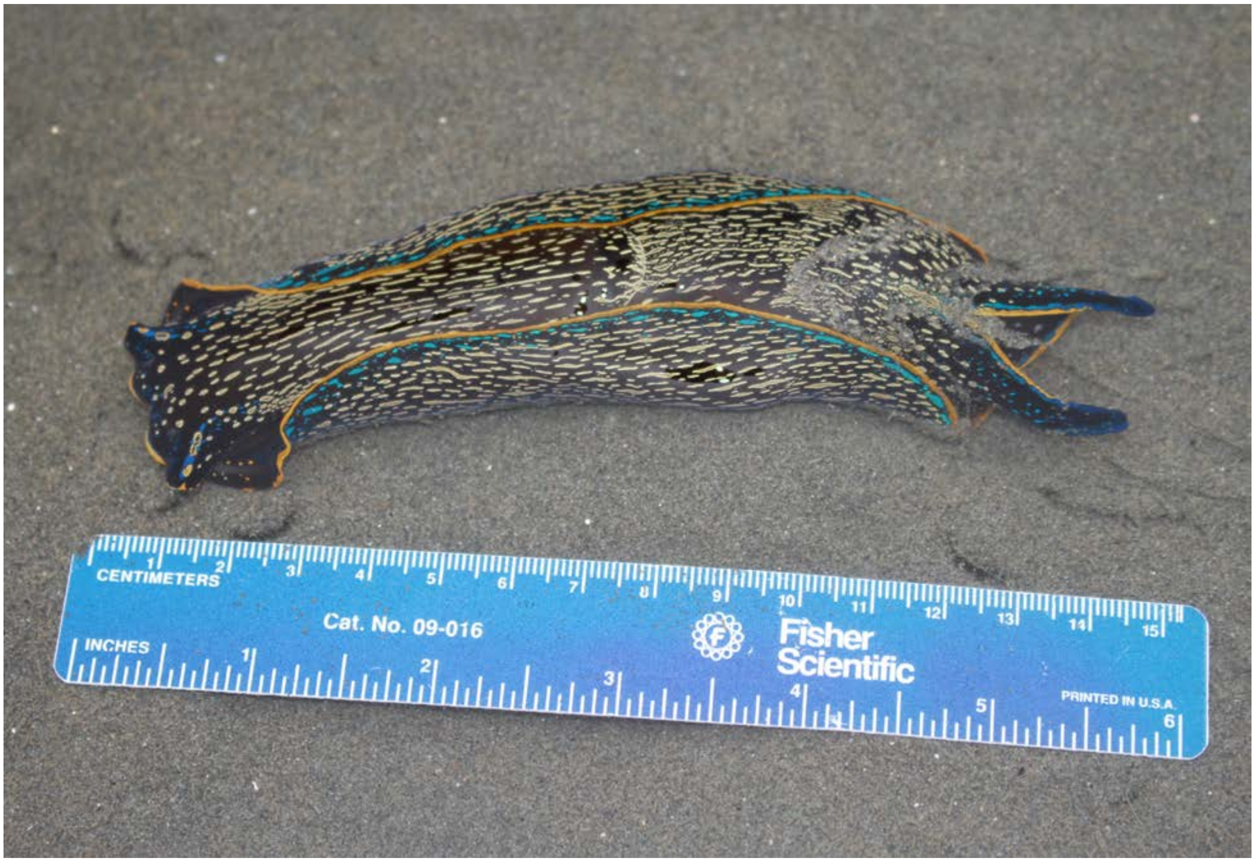

Figure S38. *Navanax inermis* (California Aglaja). Photographed in Bodega Harbor, CA, on 8 June 2016. Ruler = 15 cm. Photo credit: Jacqueline Sones.

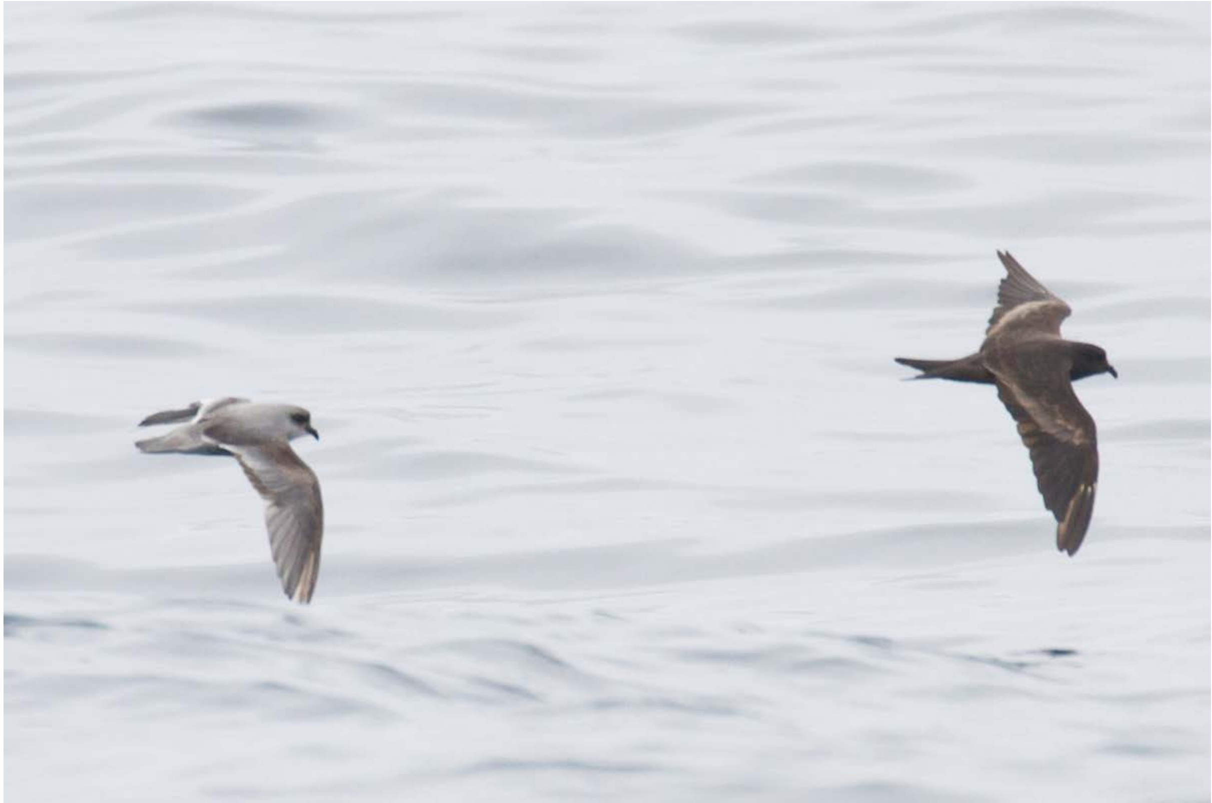

Figure S39. ***Oceanodroma melania*** **(Black Storm-Petrel)**. Black Storm-Petrel (right), photographed near Cordell Bank on 23 August 2015. Photo credit: © Steve N. G. Howell.

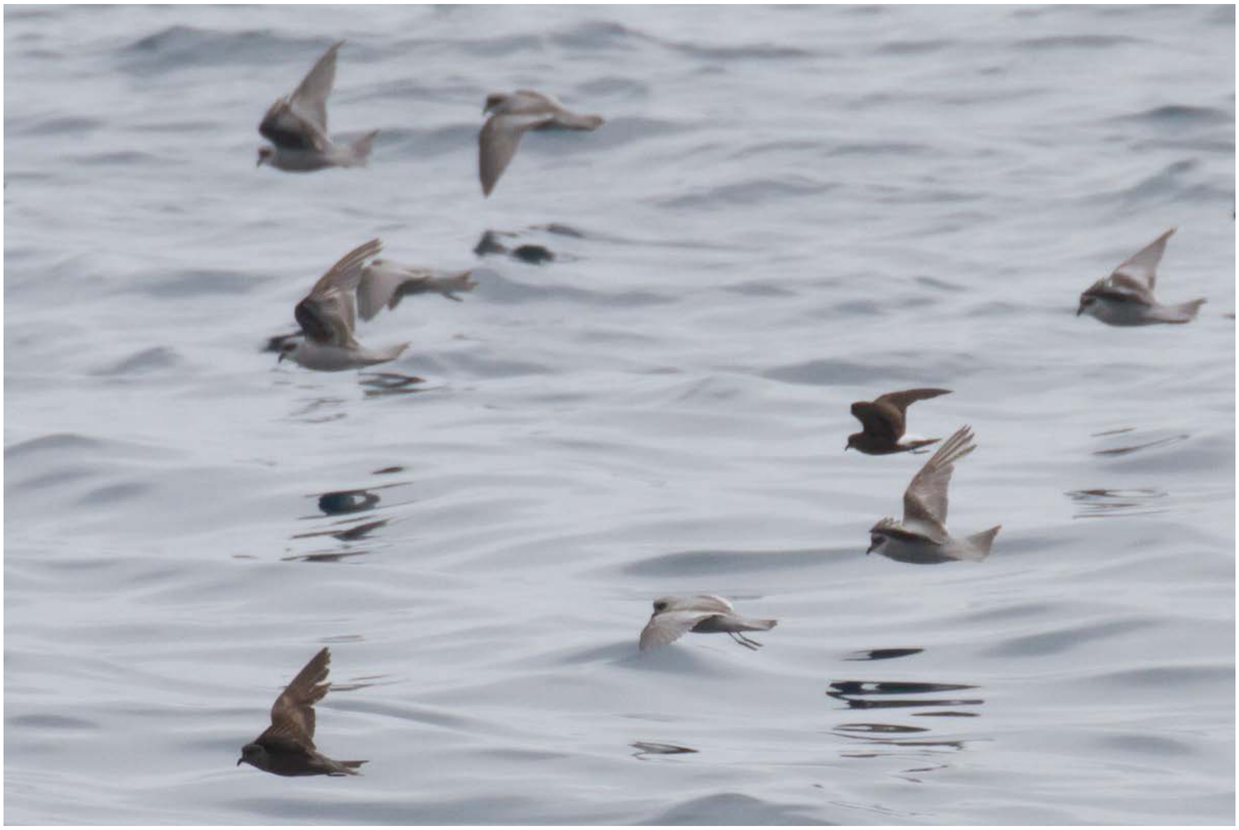

Figure S40. *Oceanodroma tethys* (Wedge-rumped Storm-Petrel). Wedge-rumped Storm-Petrel (smaller bird, center right), photographed near Cordell Bank on 23 August 2015. Photo credit: © Steve N.G. Howell.

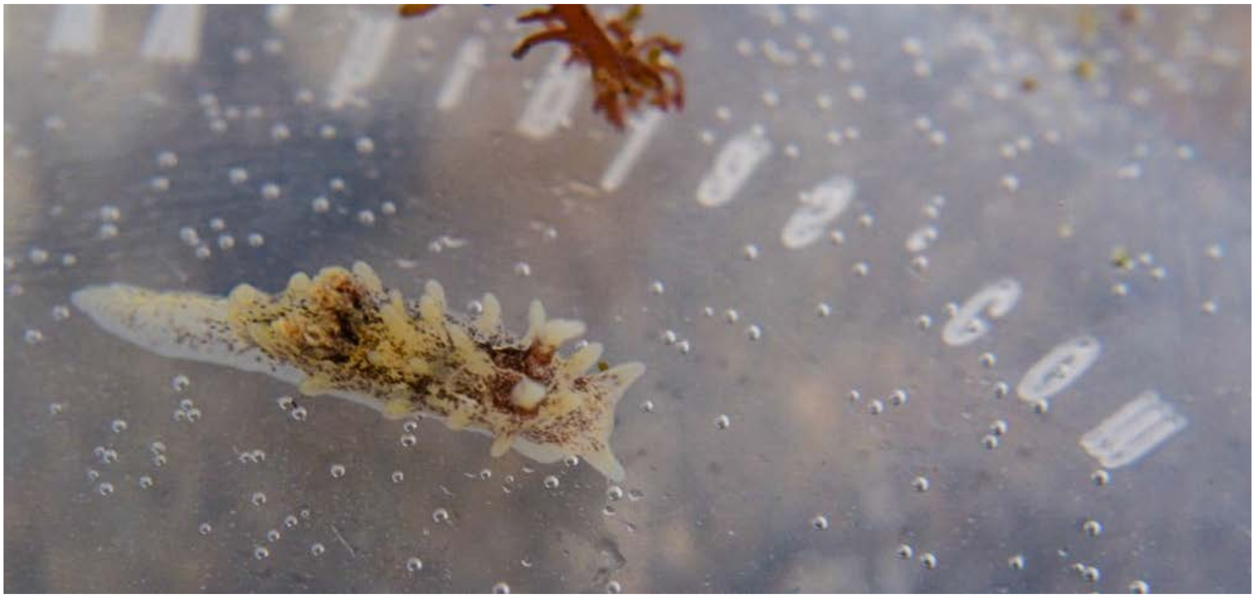

Figure S41. ***Okenia angelensis*** **(Los Angeles Okenia Nudibranch).** Photographed on 4 May 2017 at Miwok Beach, CA. Photo credit: Colby Davidson.

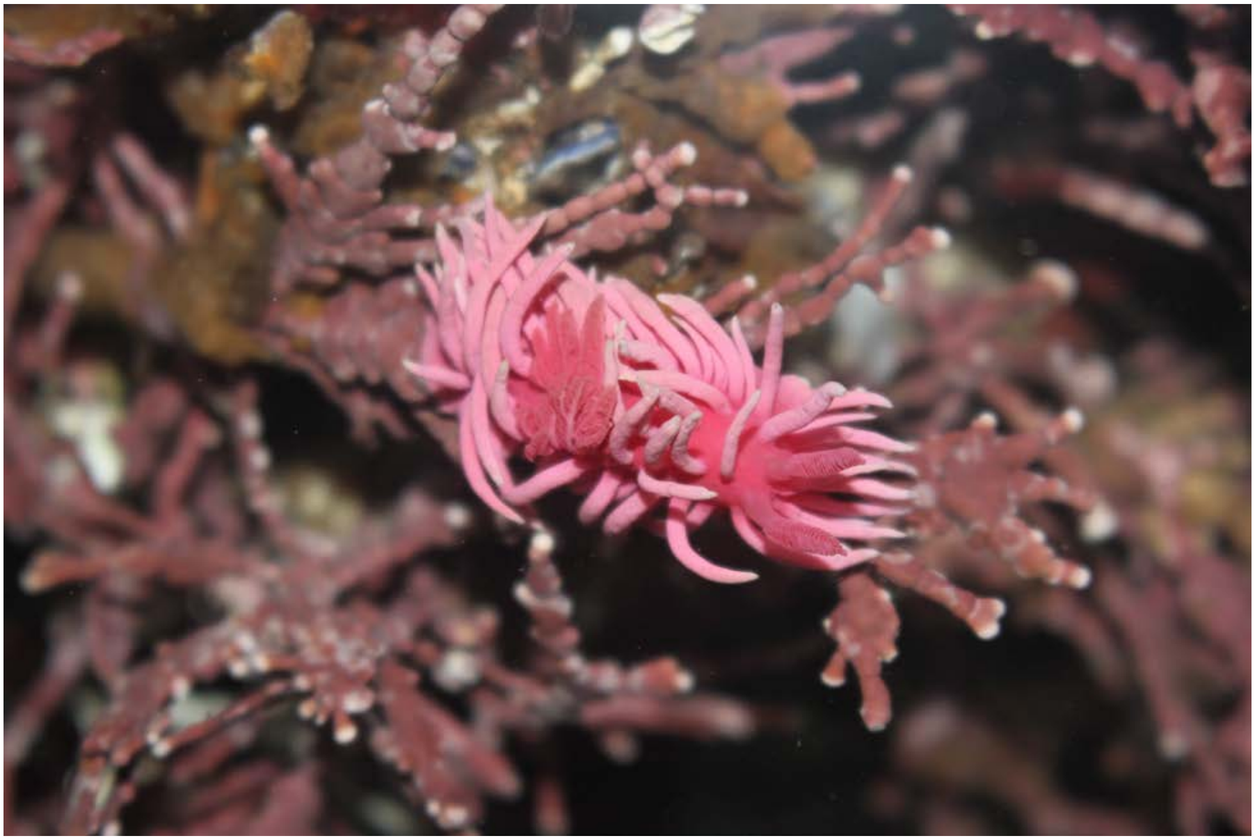

Figure S42. ***Okenia rosacea*** **(Hopkins' Rose Nudibranch).** Photographed on 25 February 2017 at Salt Point State Park, CA. Photo credit: Jacqueline Sones.

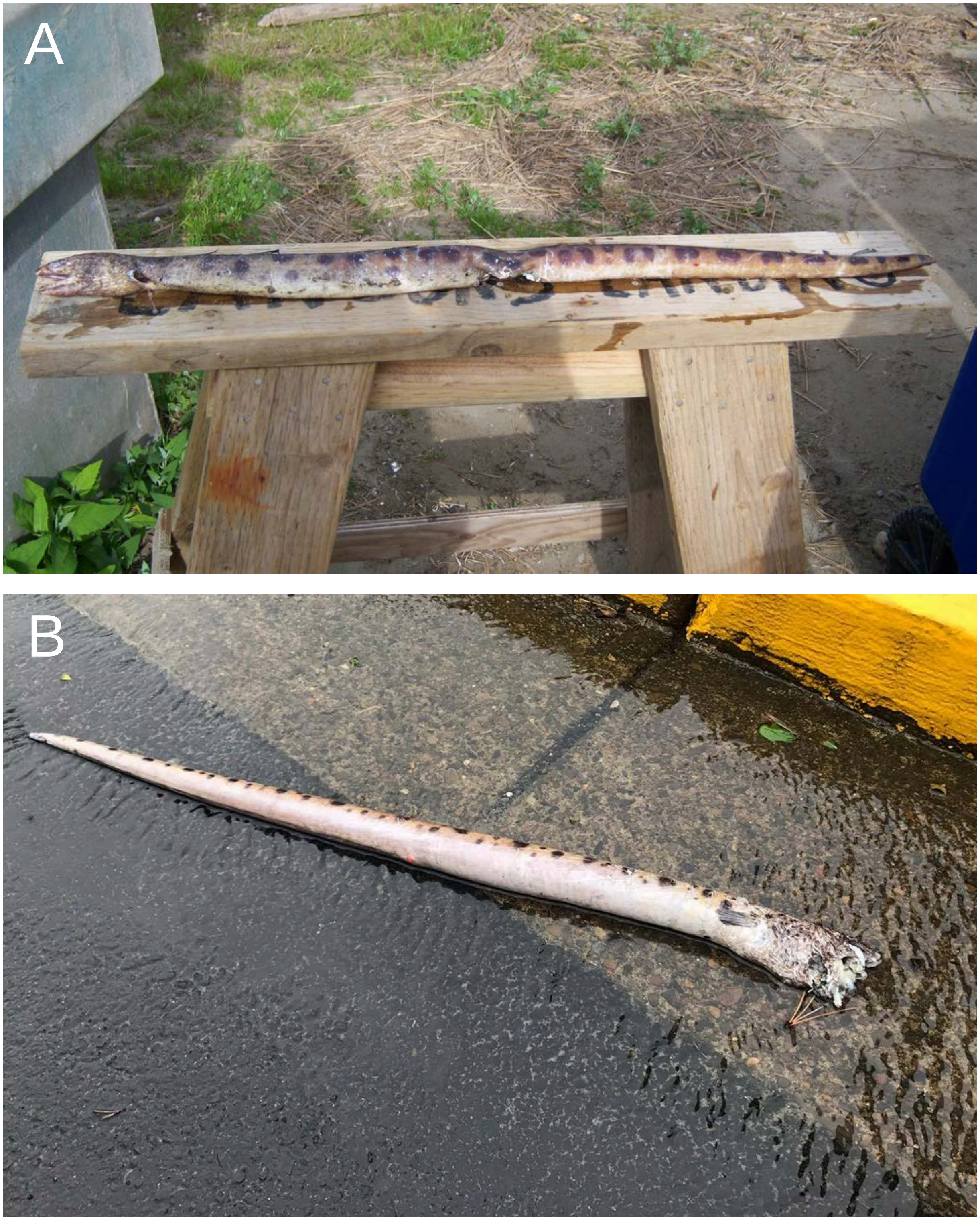

Figure S43. ***Ophichthus triserialis*** **(Pacific Snake Eel).** **(A)** Photographed on 4 May 2014 at Dillon Beach, CA. Photo credit: Cameron Vogler. **(B)** Photographed on 21 January 2017 in Lincoln City, Oregon (Oregon State Ichthyology Collection, OS 20112). Photo credit: Oregon Coast Aquarium.

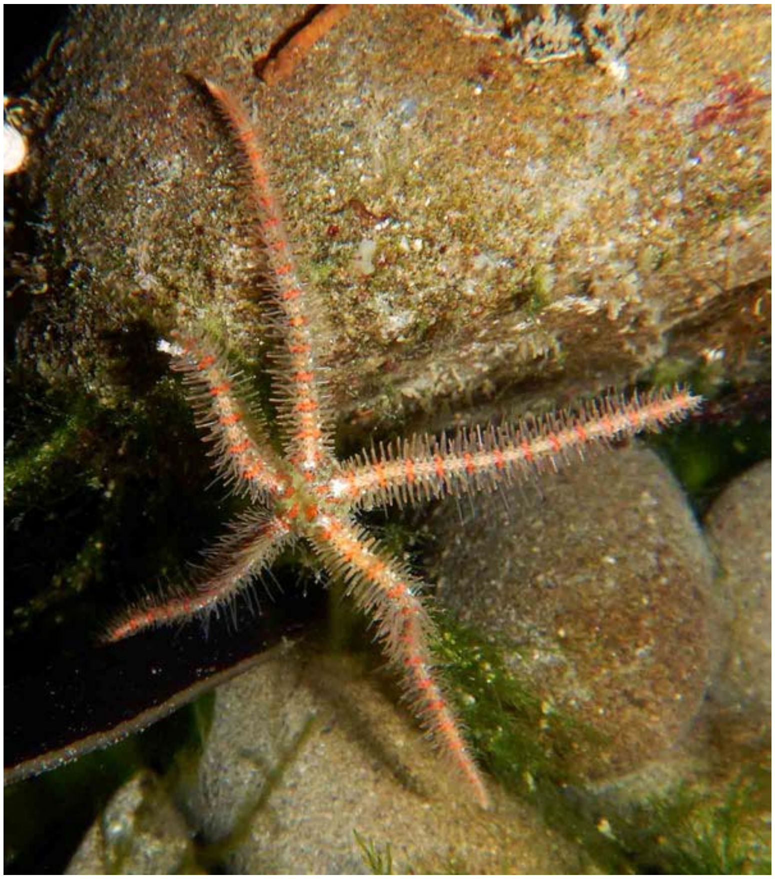

Figure S44. ***Ophiothrix spiculata*** **(Glass-spined Brittle Star).** Photographed on 4 July 2015 at Patrick's Point State Park, CA. Photo credit: Mike Kelly.

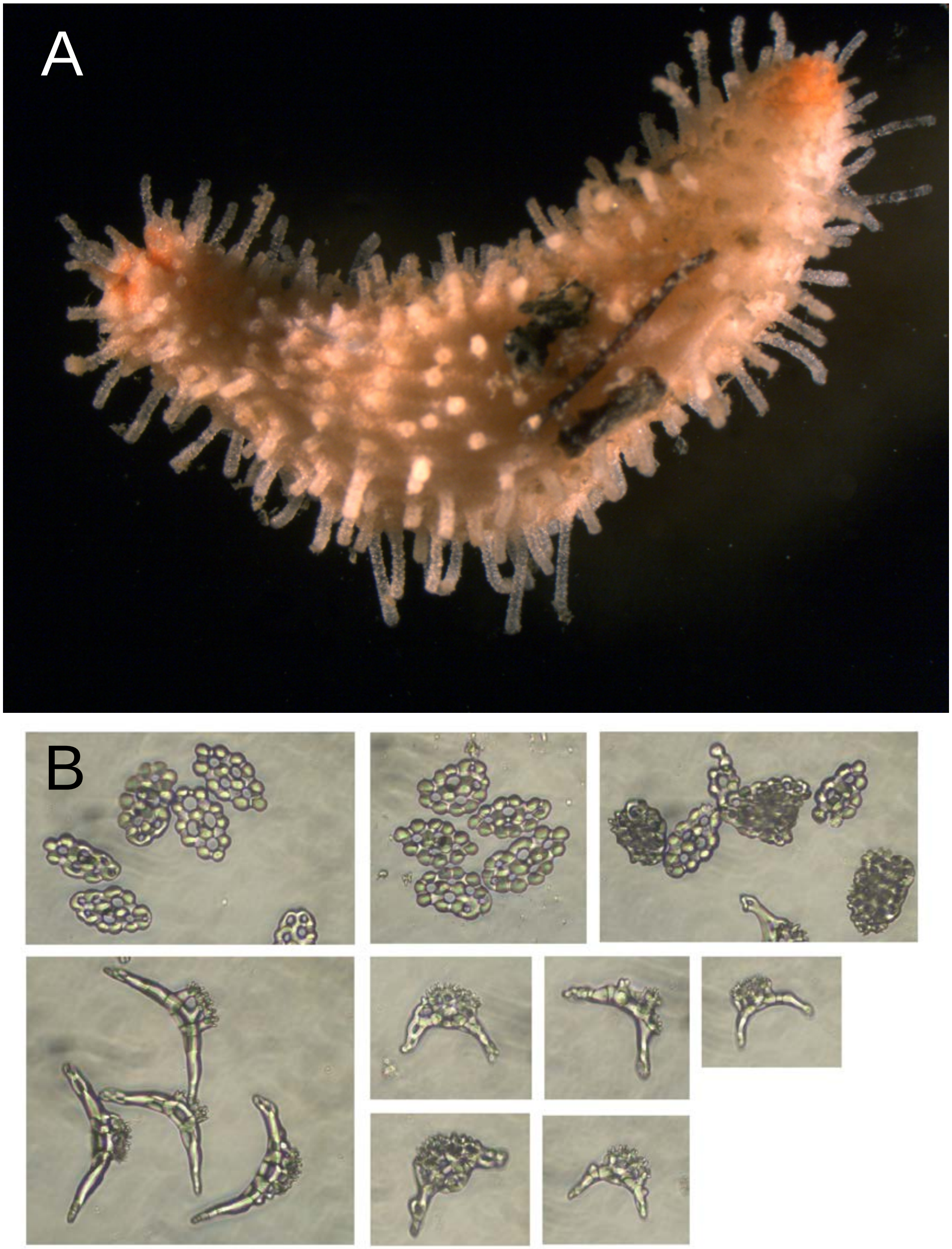

Figure S45. *Pachythyone rubra* (Red Sea Cucumber). (A) Collected on 30 December 2017 from Bodega Marine Reserve, CA. Specimen deposited at California Academy of Sciences (CASIZ 227281). Photo credit: Jacqueline Sones. **(B)** Species identification confirmed by inspection of microscopic skin ossicles (upper row) and tube foot ossicles (lower rows). All photographs taken at 200x magnification. Photo credit: Eric Sanford.

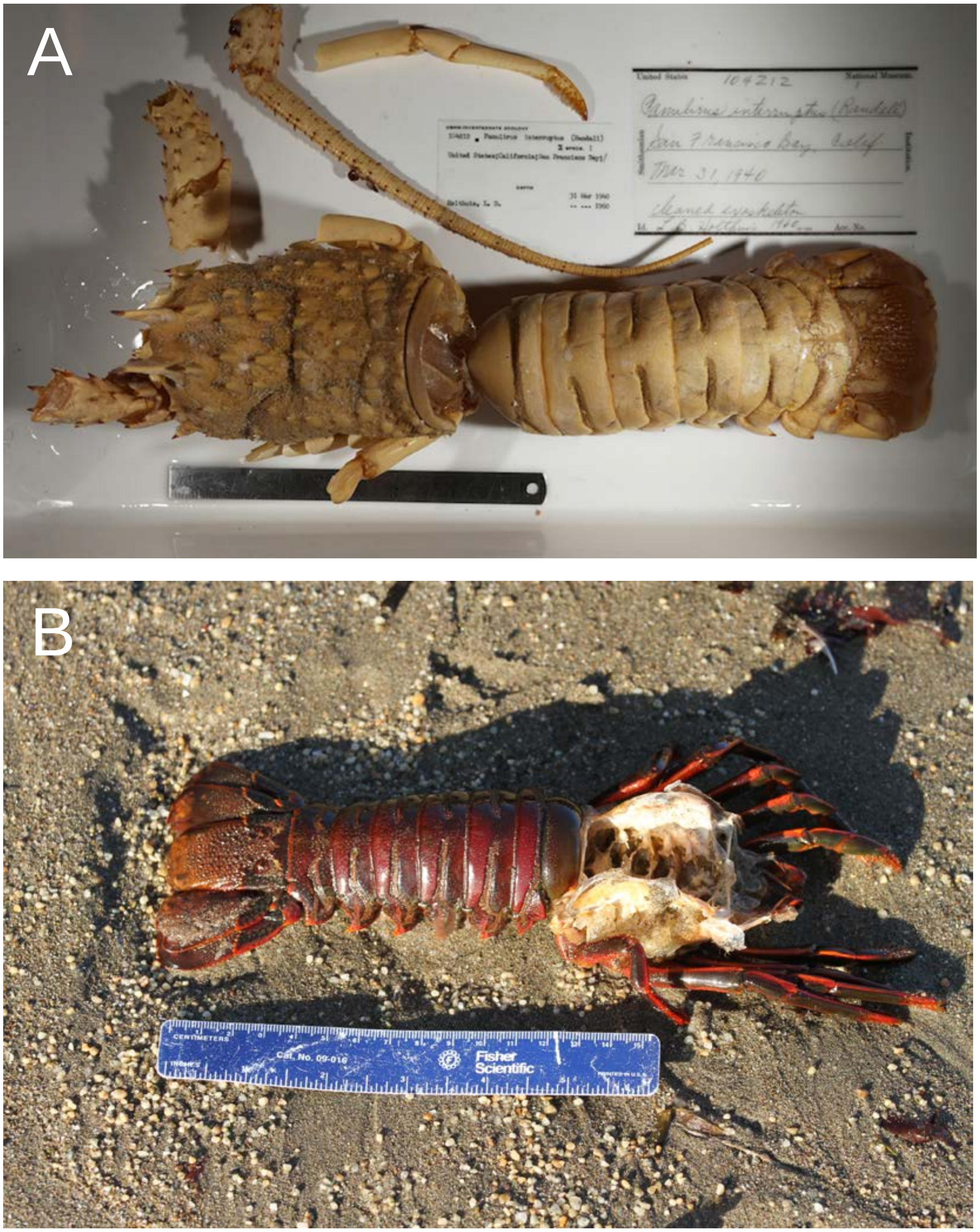

Figure S46. *Panulirus interruptus* (Spiny Lobster). (A) Specimen from the United States National Museum, collected in San Francisco Bay, CA, in March 1940 (USNM 104212). Ruler = 15 cm. Photo credit: Sarit Truskey. **(B)** Molt collected on 19 September 2017 in Horseshoe Cove (Bodega Marine Reserve), CA. Specimen deposited at California Academy of Sciences (CASIZ 224109). Photo credit: Jacqueline Sones.

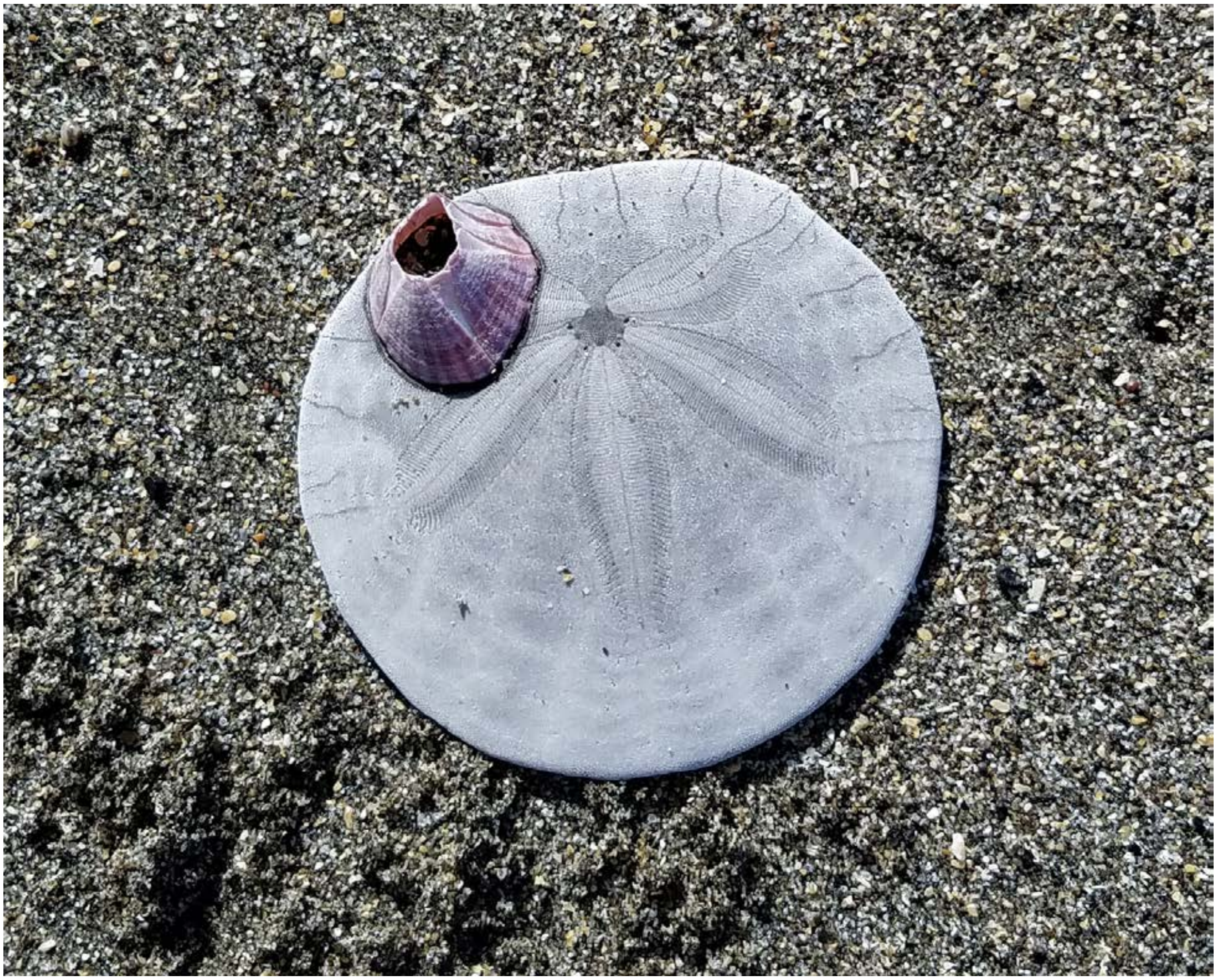

Figure S47. *Paraconcavus pacificus* (Red-striped Barnacle). Photographed at MacKerricher State Park, CA, on 12 November 2017. Specimen deposited at California Academy of Sciences (CASIZ 224114). Photo credit: Les Rohssler.

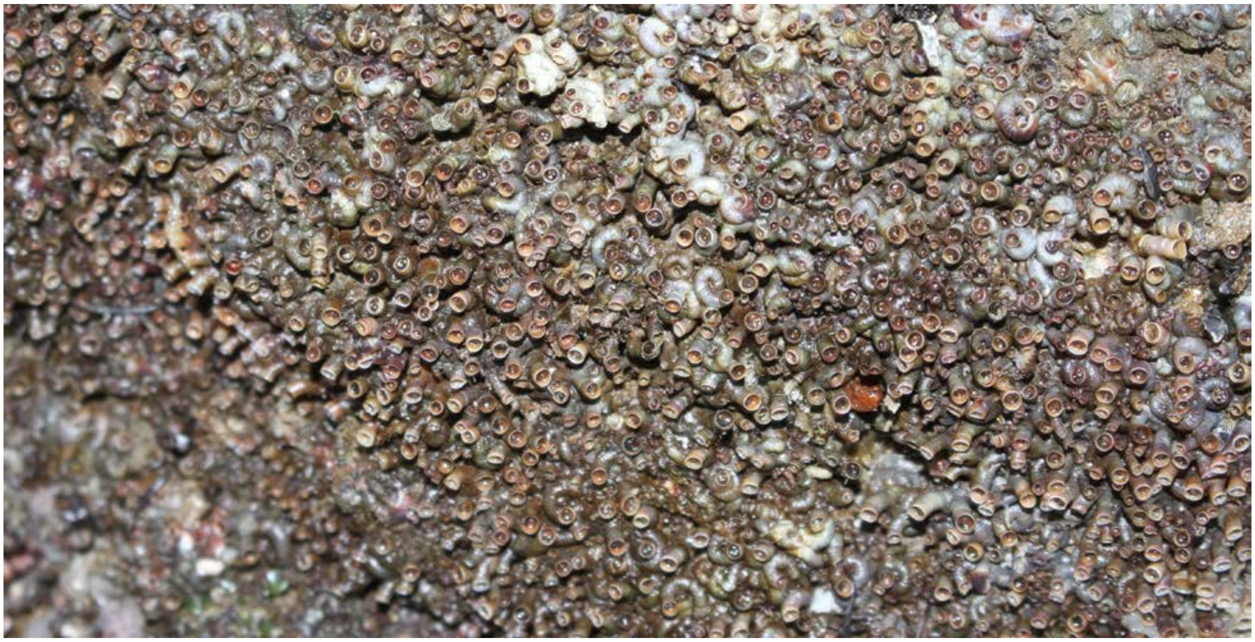

Figure S48. *Petaloconchus montereyensis* (Monterey Tube Snail). High density of *Petaloconchus montereyensis* in the low intertidal zone. Photographed on 24 February 2017 at Salt Point State Park, CA. Photo credit: Jacqueline Sones.

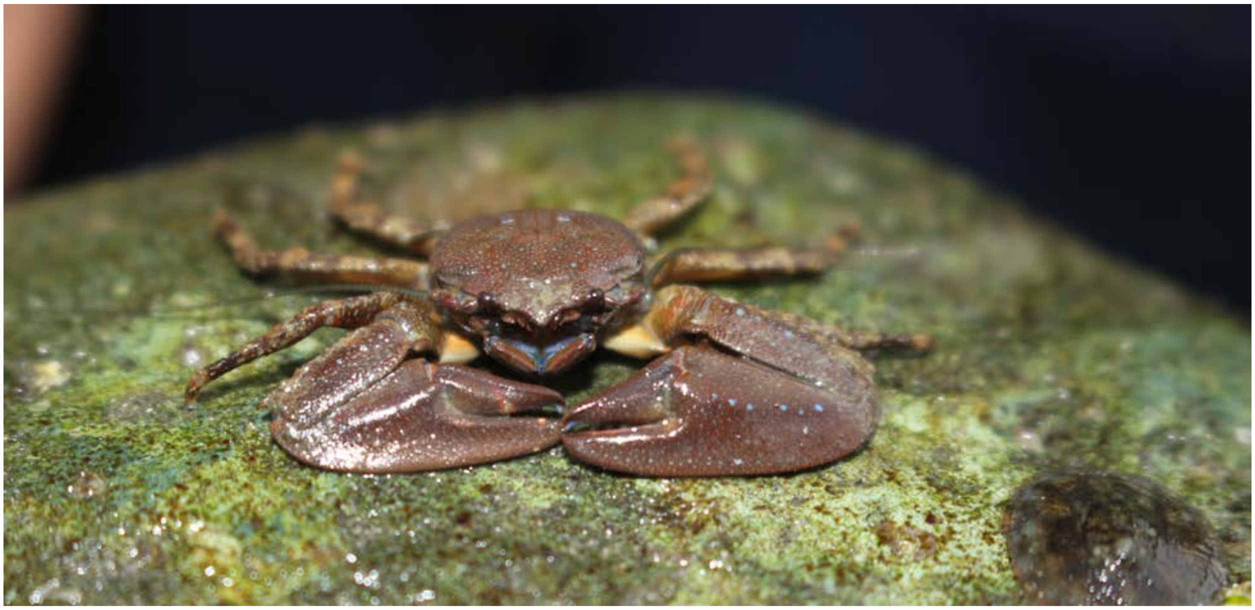

Figure S49. ***Petrolisthes manimaculis*** **(Chocolate Porcelain Crab).** Photographed on 27 May 2017 at Point St. George, CA. Specimen deposited at California Academy of Sciences (CASIZ 224112). Photo credit: Jacqueline Sones.

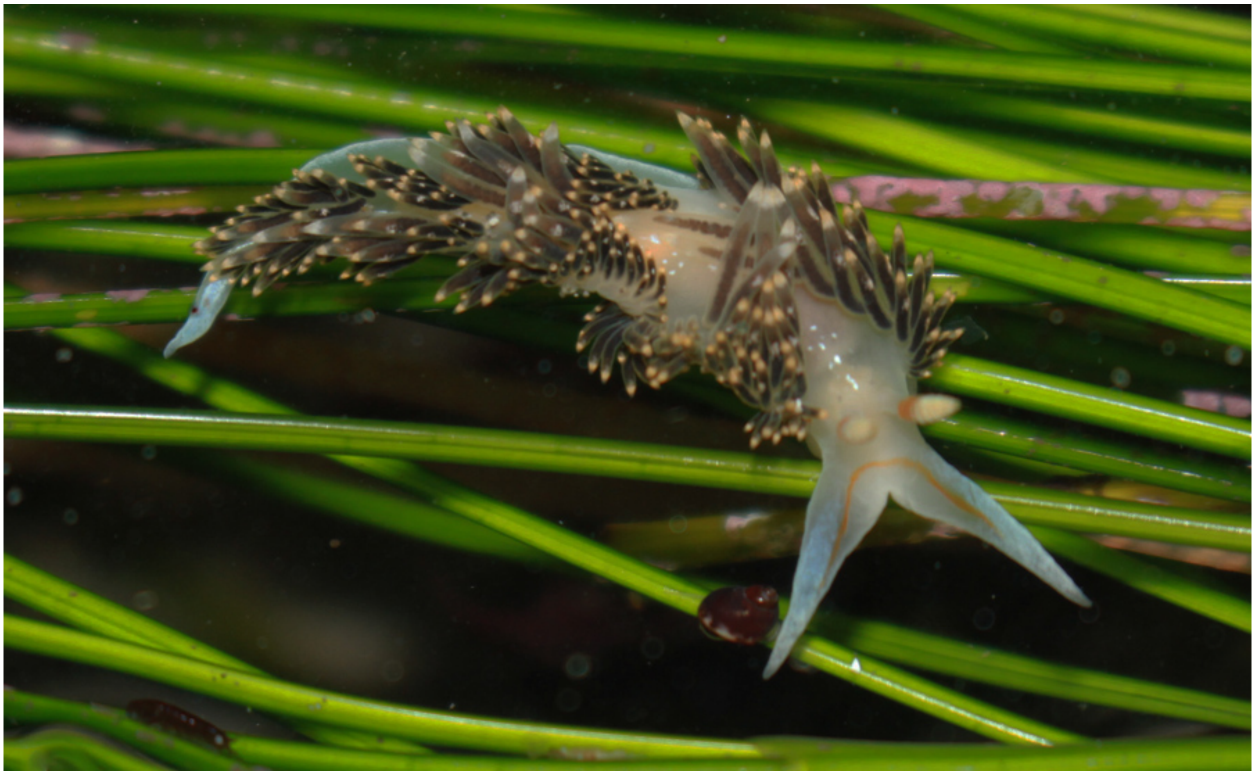

Figure S50. ***Phidiana hiltoni*** **(Hilton's Nudibranch).** Photographed on 25 November 2015 at Pinnacle Gulch, CA. Photo credit: Jacqueline Sones.

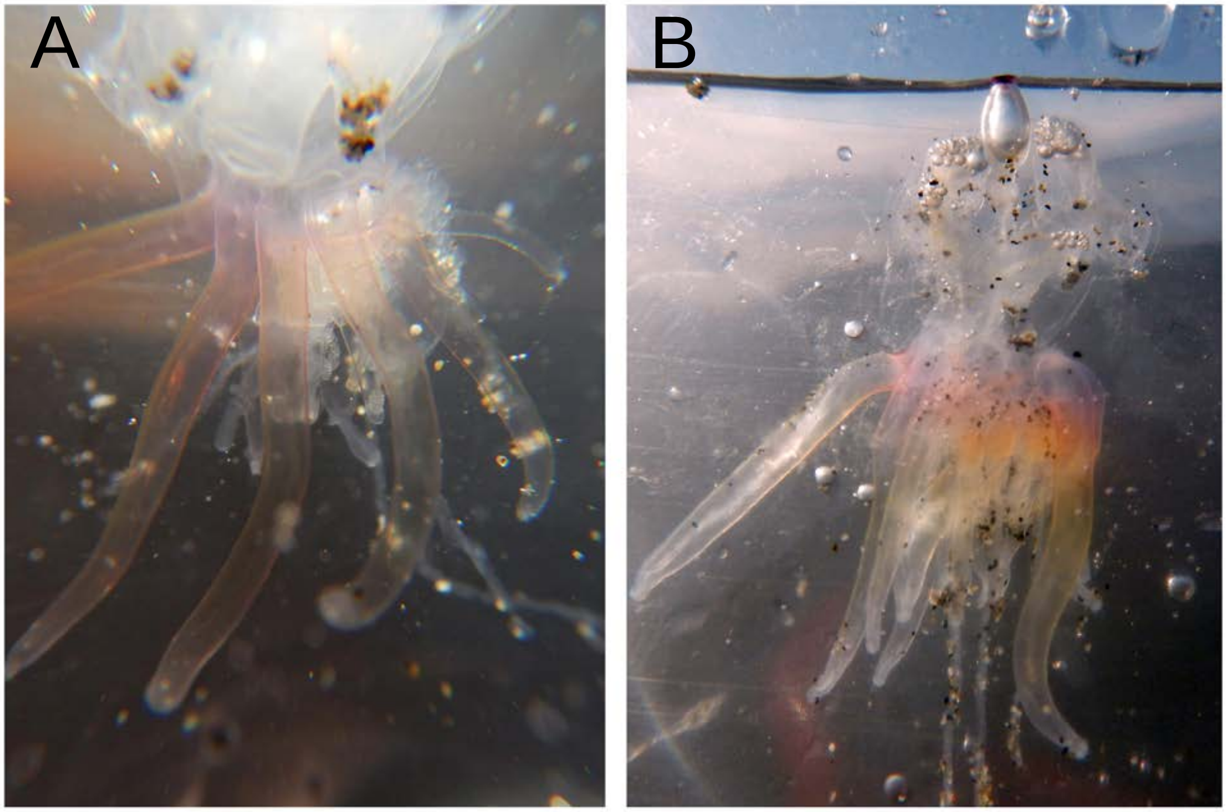

Figure S51. *Physophora hydrostatica* (Hula Skirt Siphonophore). Photographed at Mad River Beach, CA, on **(A)** 13 December 2017 and **(B)** 5 March 2018. Photo credits: Mike Kelly.

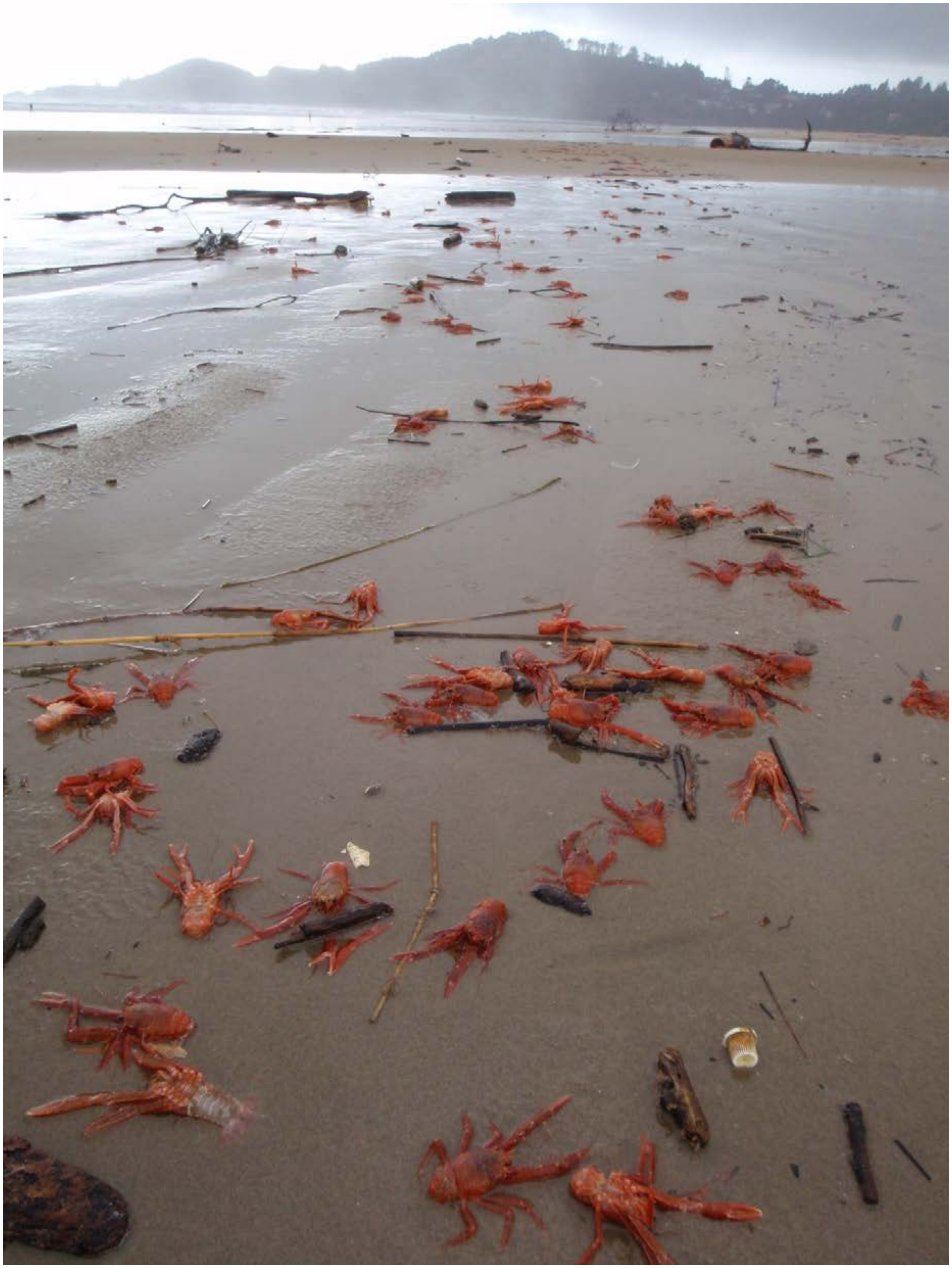

Figure S52. ***Pleuroncodes planipes*** **(Pelagic Red Crab).** Photographed on 1 March 2017 at Agate Beach, Oregon. Photo credit: David Fox, Oregon Department of Fish and Wildlife Marine Resources Program.

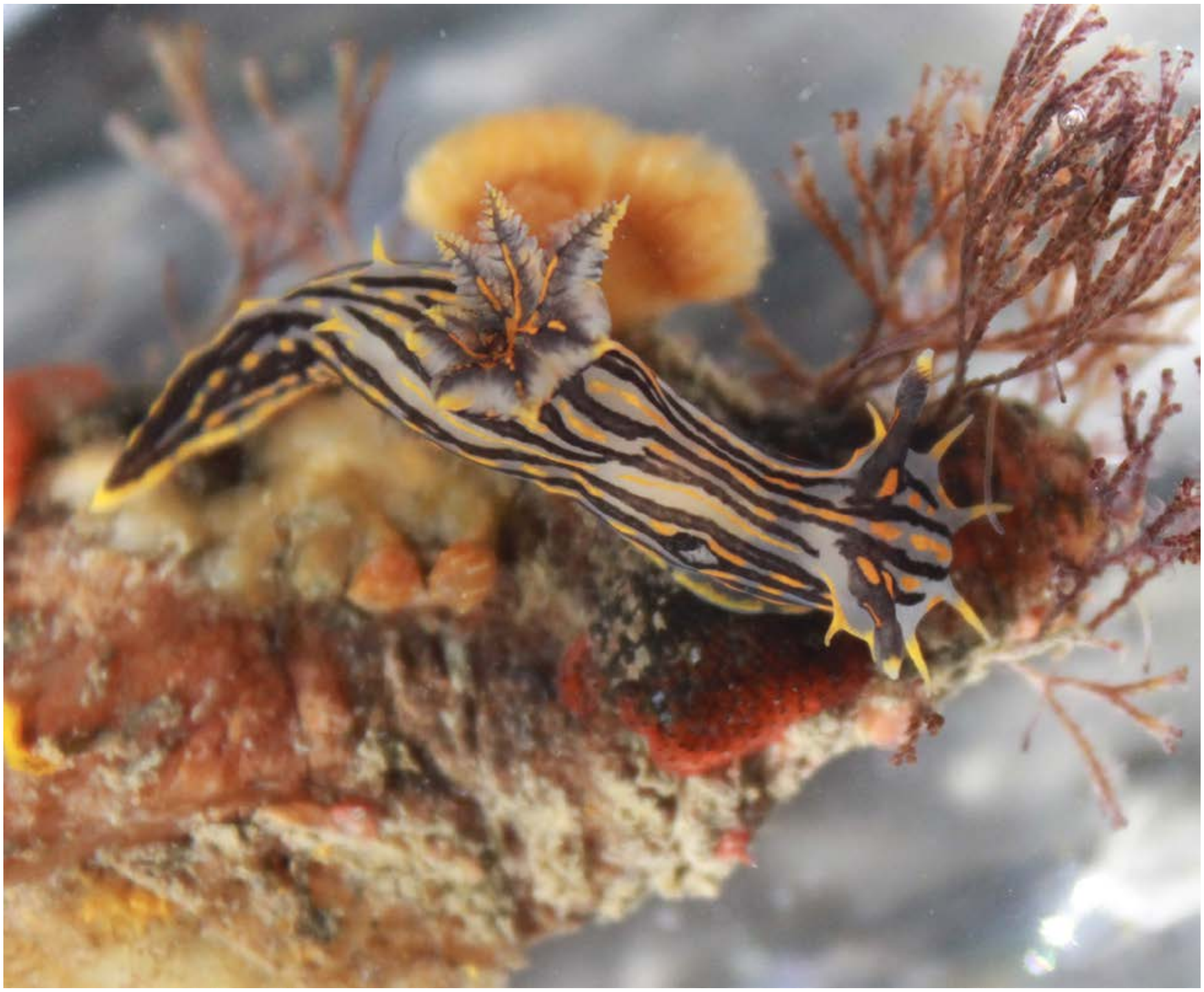

Figure S53. ***Polycera atra*** **(Orange-spike Polycera Nudibranch).** Photographed on 19 October 2014 in Bodega Harbor, CA. Photo credit: Jacqueline Sones.

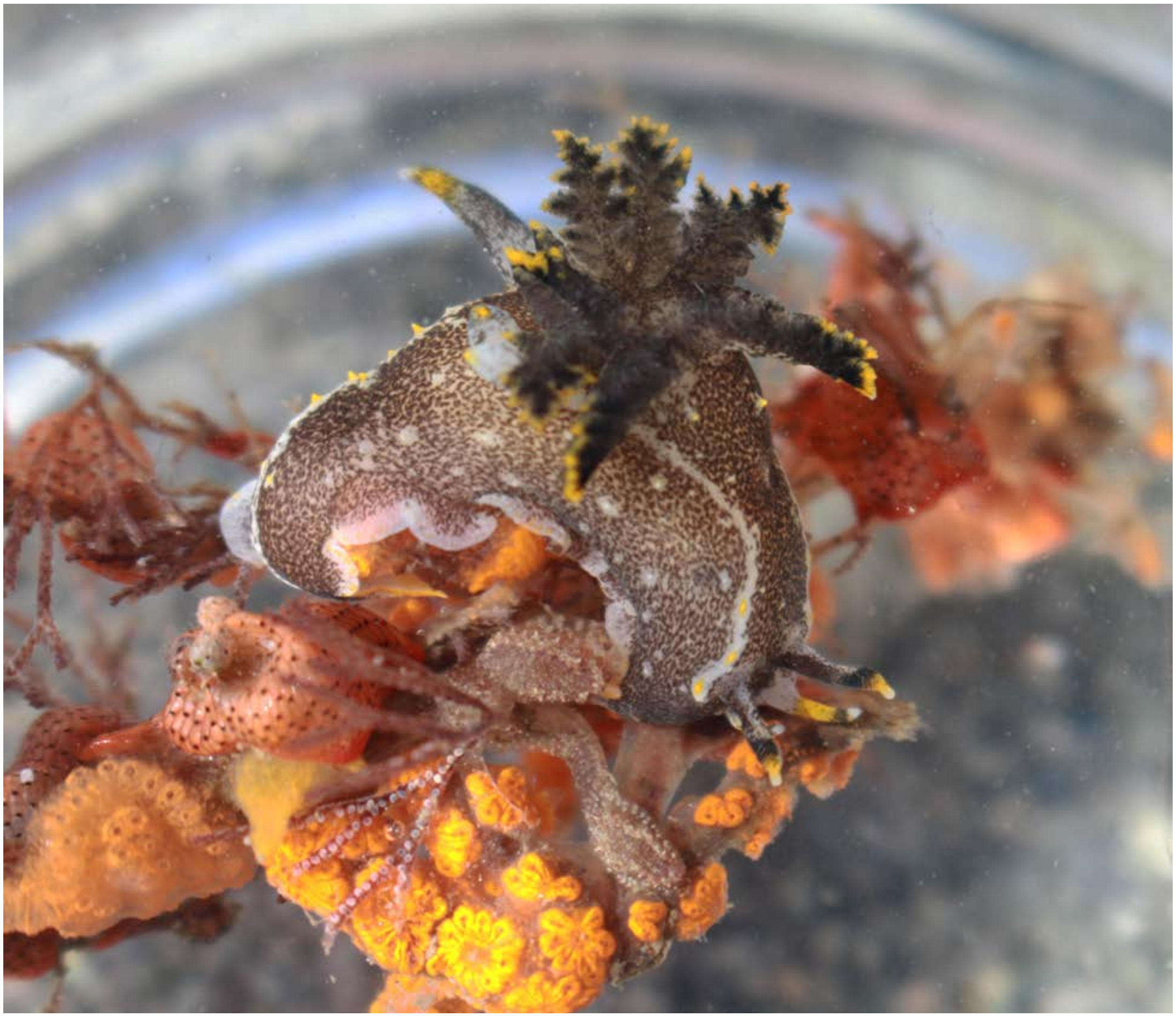

Figure S54. *Polycera hedgpethi* (Hedgpeth's Nudibranch). Photographed on 16 November 2015 in Bodega Harbor, CA. Photo credit: Jacqueline Sones.

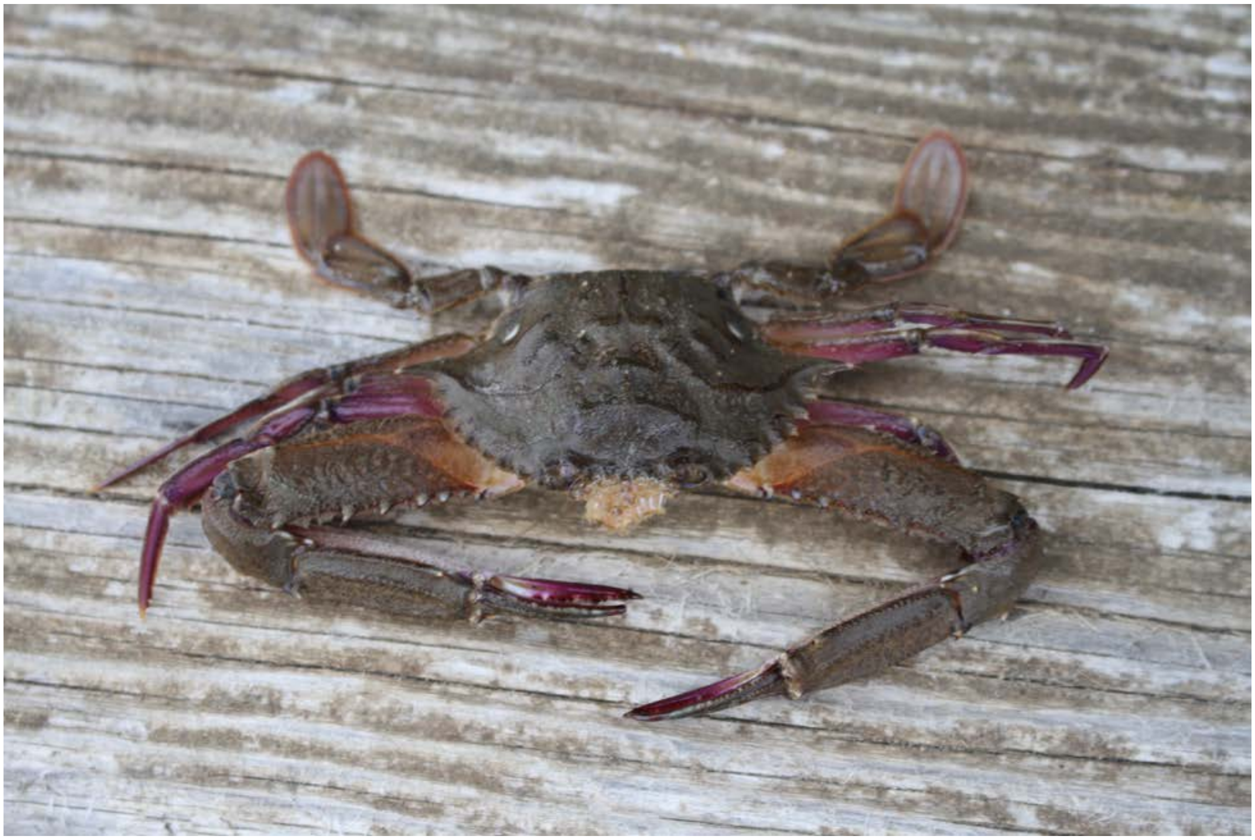

Figure S55. ***Portunus xantusii*** **(Xantus' Swimming Crab).** Collected on 21 August 2016 in Tomales Bay, CA, by Jason Sadowski and Ted Grosholz. Photo credit: Jacqueline Sones.

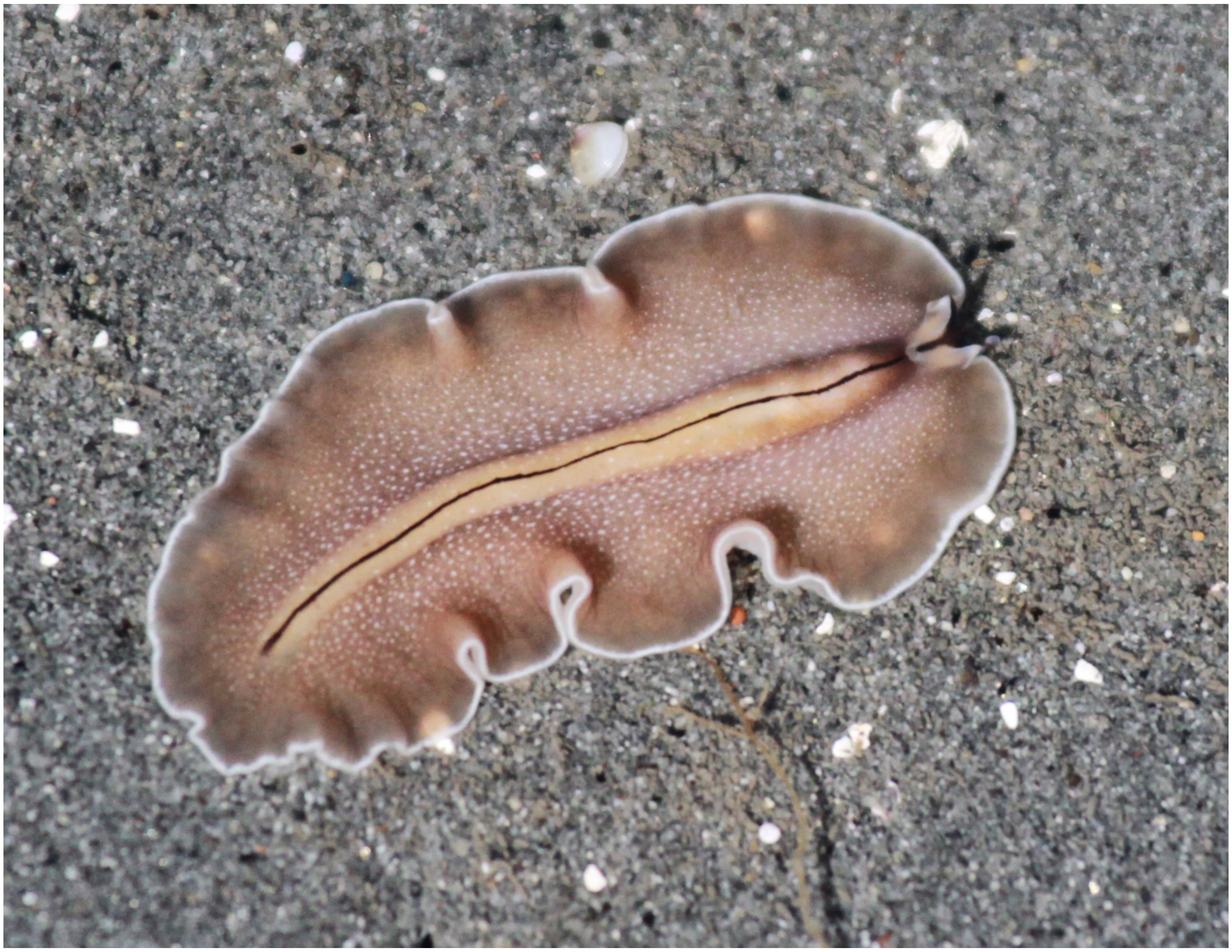

Figure S56. ***Pseudoceros luteus*** **(White Flatworm).** Photographed on 24 September 2015 in Bodega Harbor, CA. Photo credit: Jacqueline Sones.

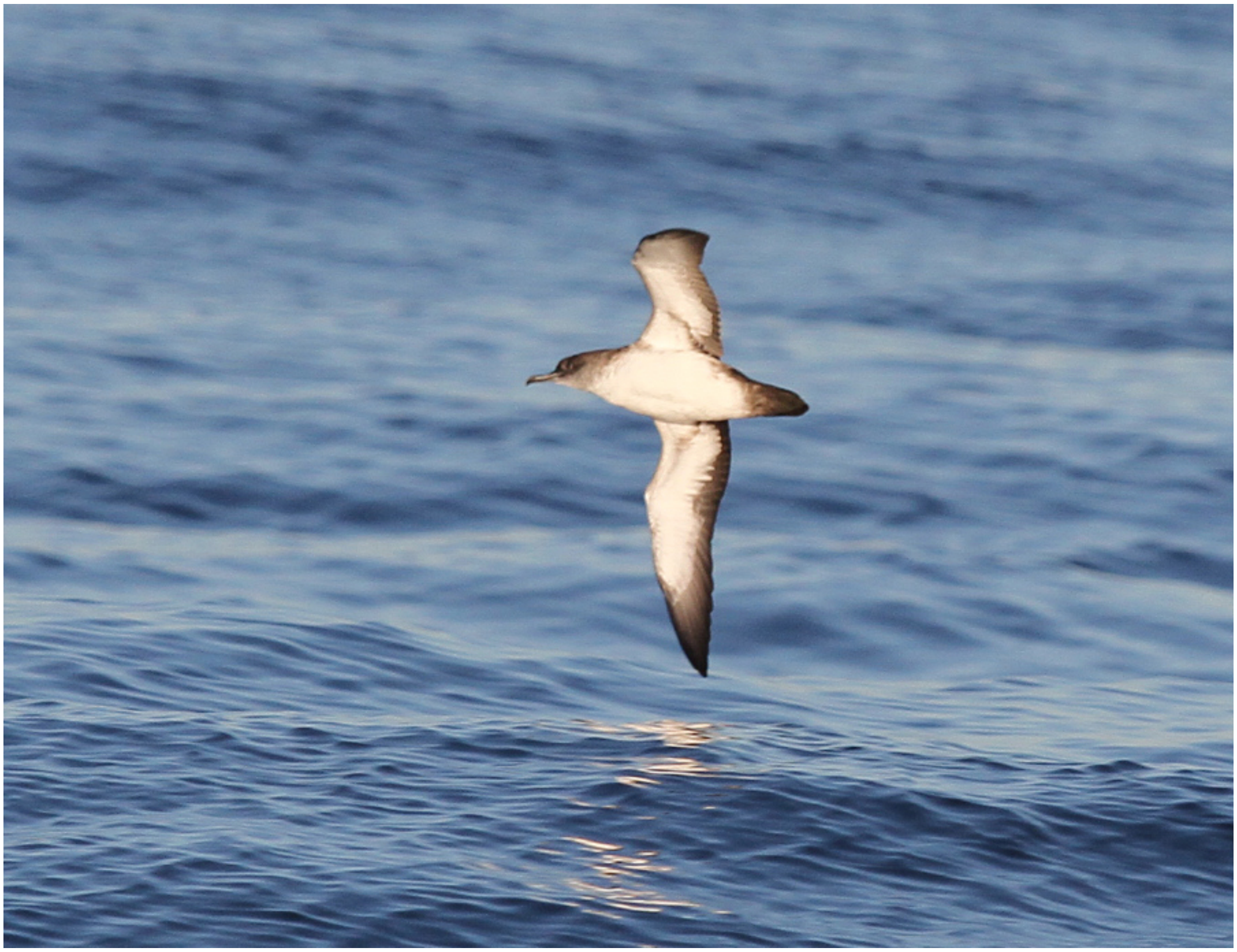

Figure S57. ***Puffinus opisthomelas*** **(Black-vented Shearwater).** Photographed on 9 October 2016 off Bodega Head, CA. Photo credit: Jacqueline Sones.

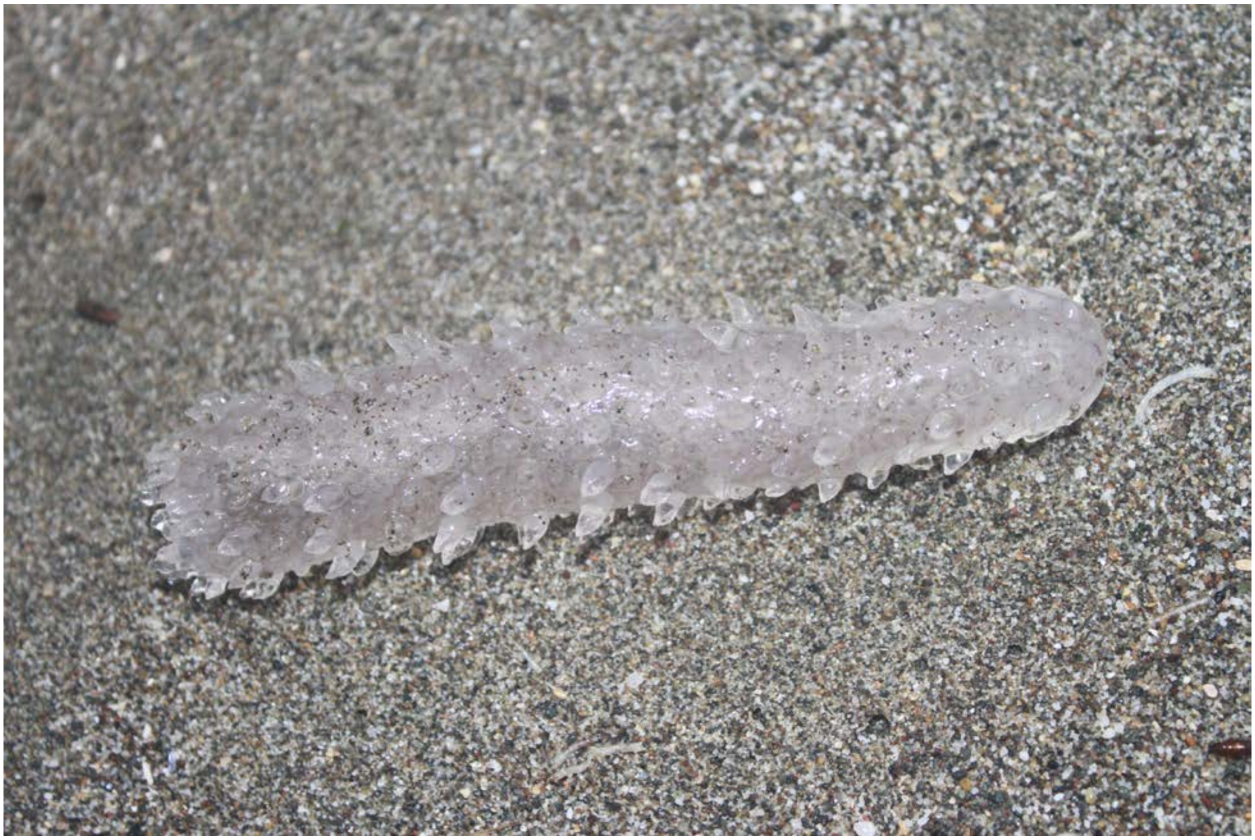

Figure S58. *Pyrosoma atlanticum* (Pyrosome). Photographed on 8 December 2014 on Salmon Creek Beach, CA. Photo credit: Jacqueline Sones.

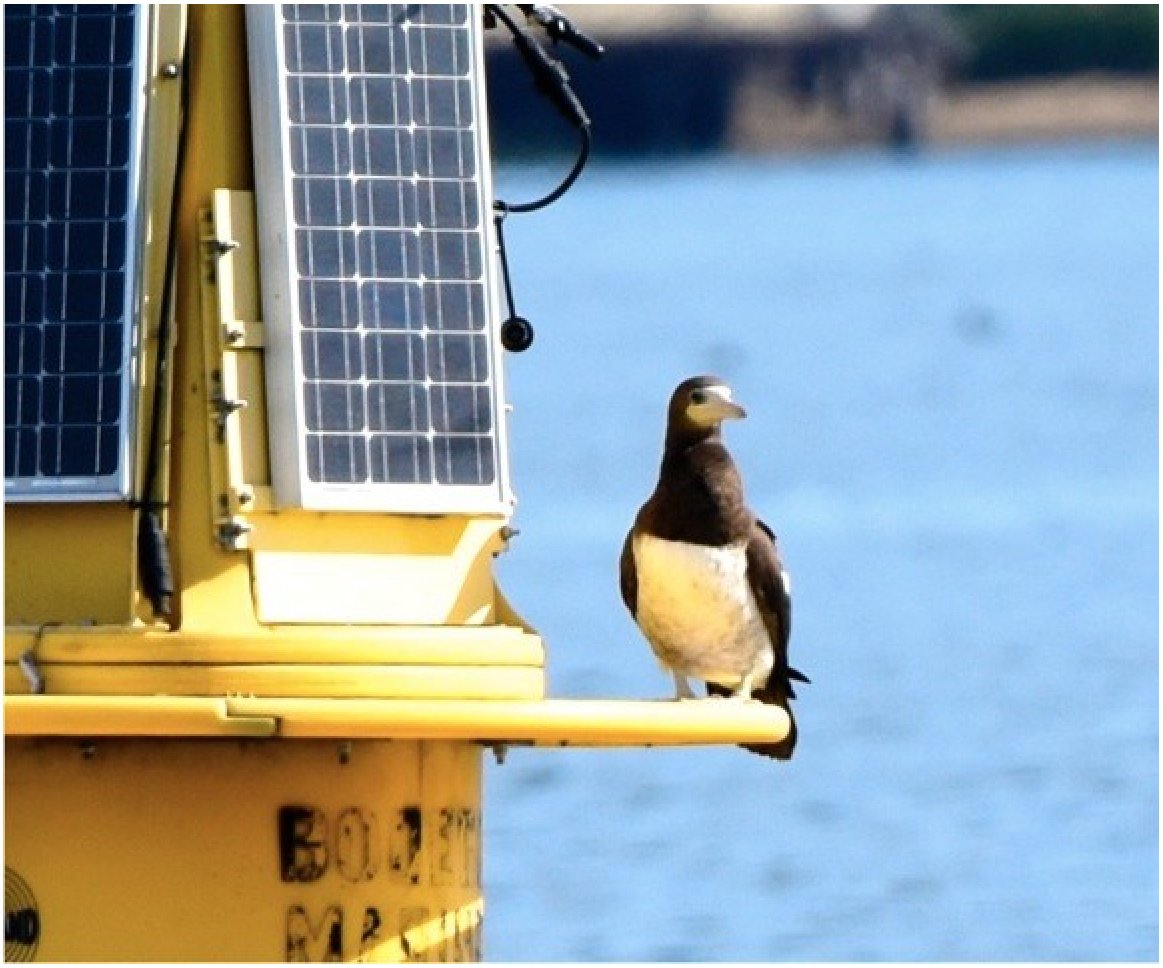

Figure S59. ***Sula leucogaster*** **(Brown Booby).** Photographed on 6 February 2016 in Tomales Bay, CA. Photo credit: Roger Harshaw.

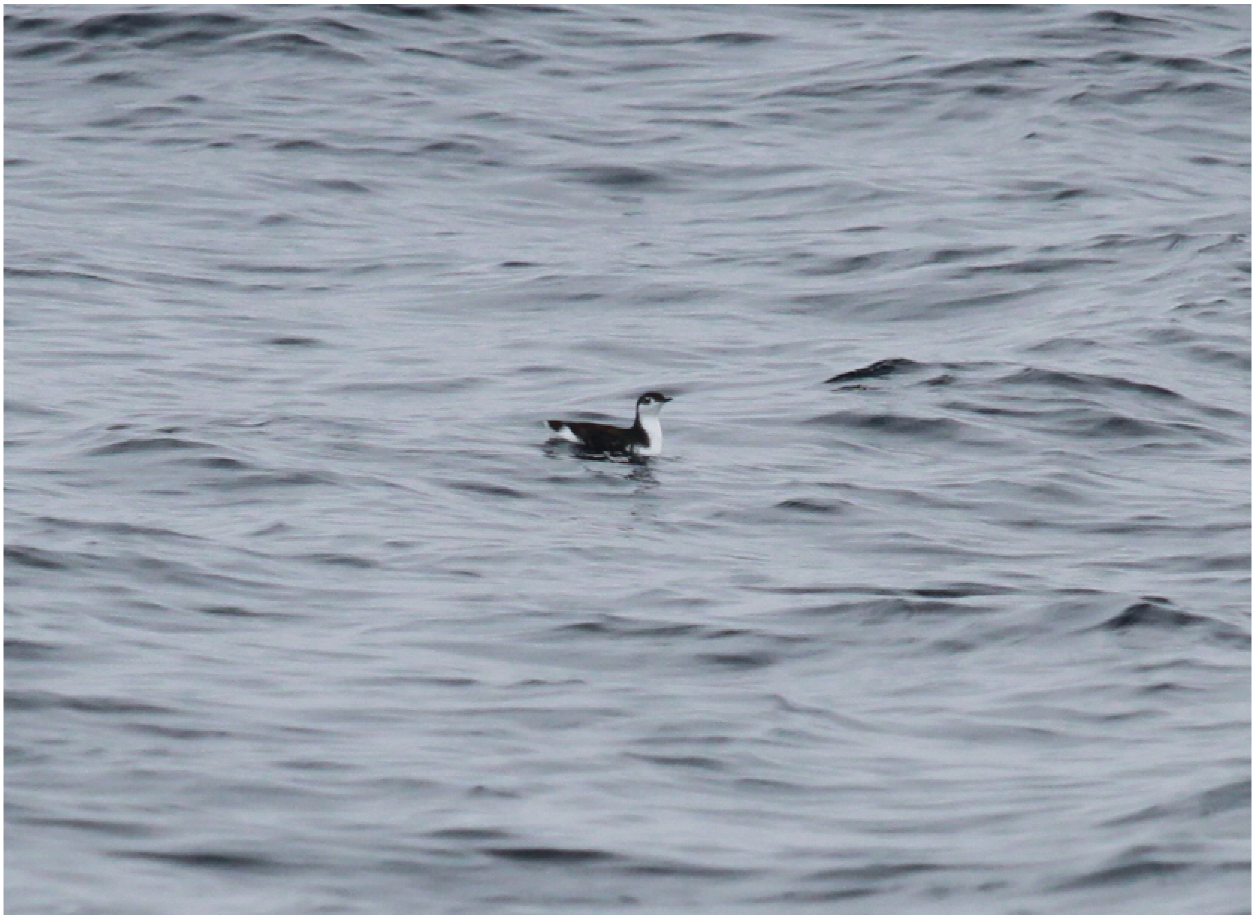

Figure S60. *Synthliboramphus hypoleucus* (Guadalupe Murrelet). Photographed on 19 September 2014 near Cordell Bank. Photo credit: Jacqueline Sones.

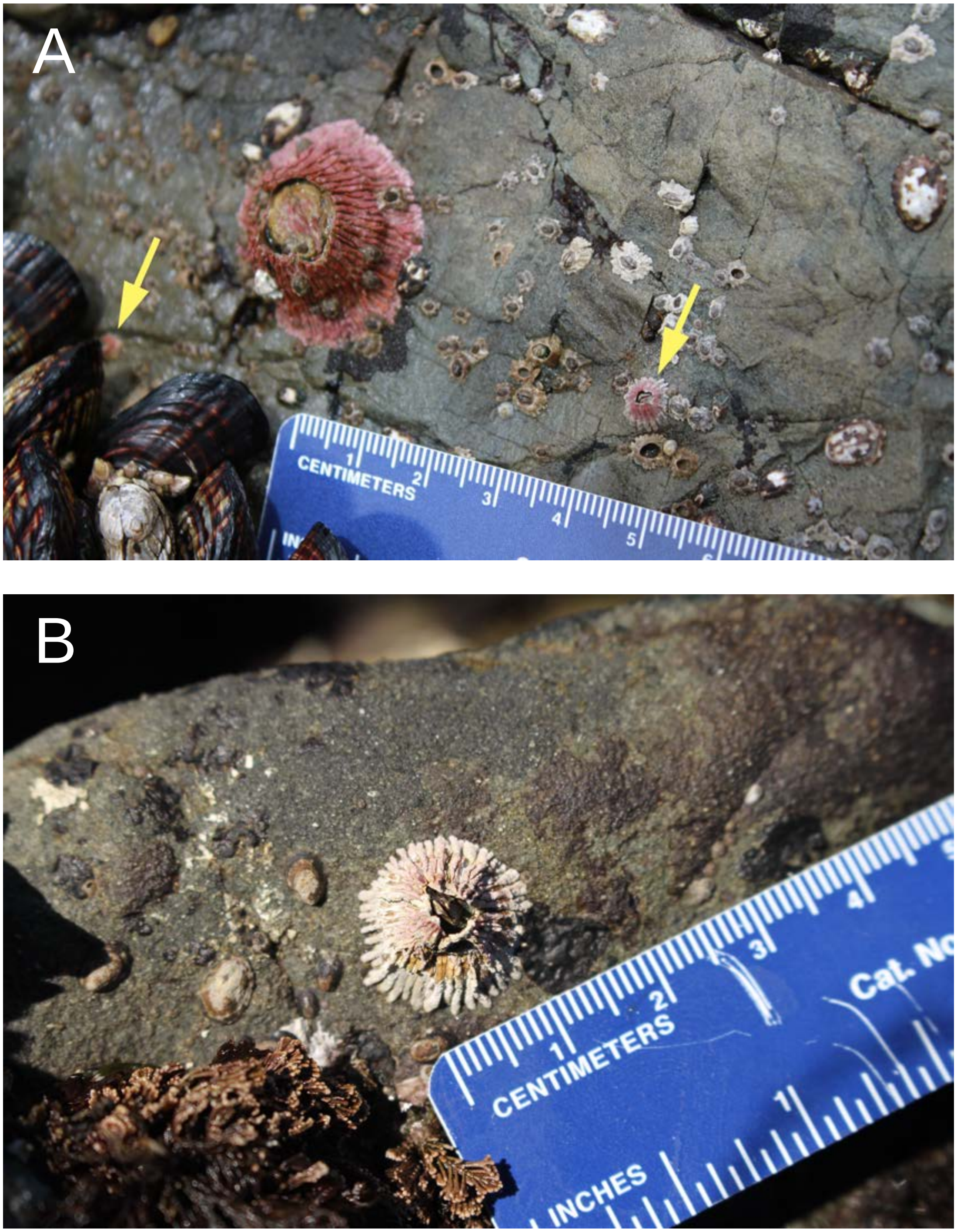

Figure S61. *Tetraclita rubescens* (Pink Volcano Barnacle). (A) Juvenile *Tetraclita* (yellow arrows) photographed on 7 June 2015 at Van Damme State Park, CA. Photo credit: Jacqueline Sones. **(B)** One of five individuals observed on 31 May 2017 at Patrick’s Point State Park, CA. Photo credit: Jacqueline Sones.

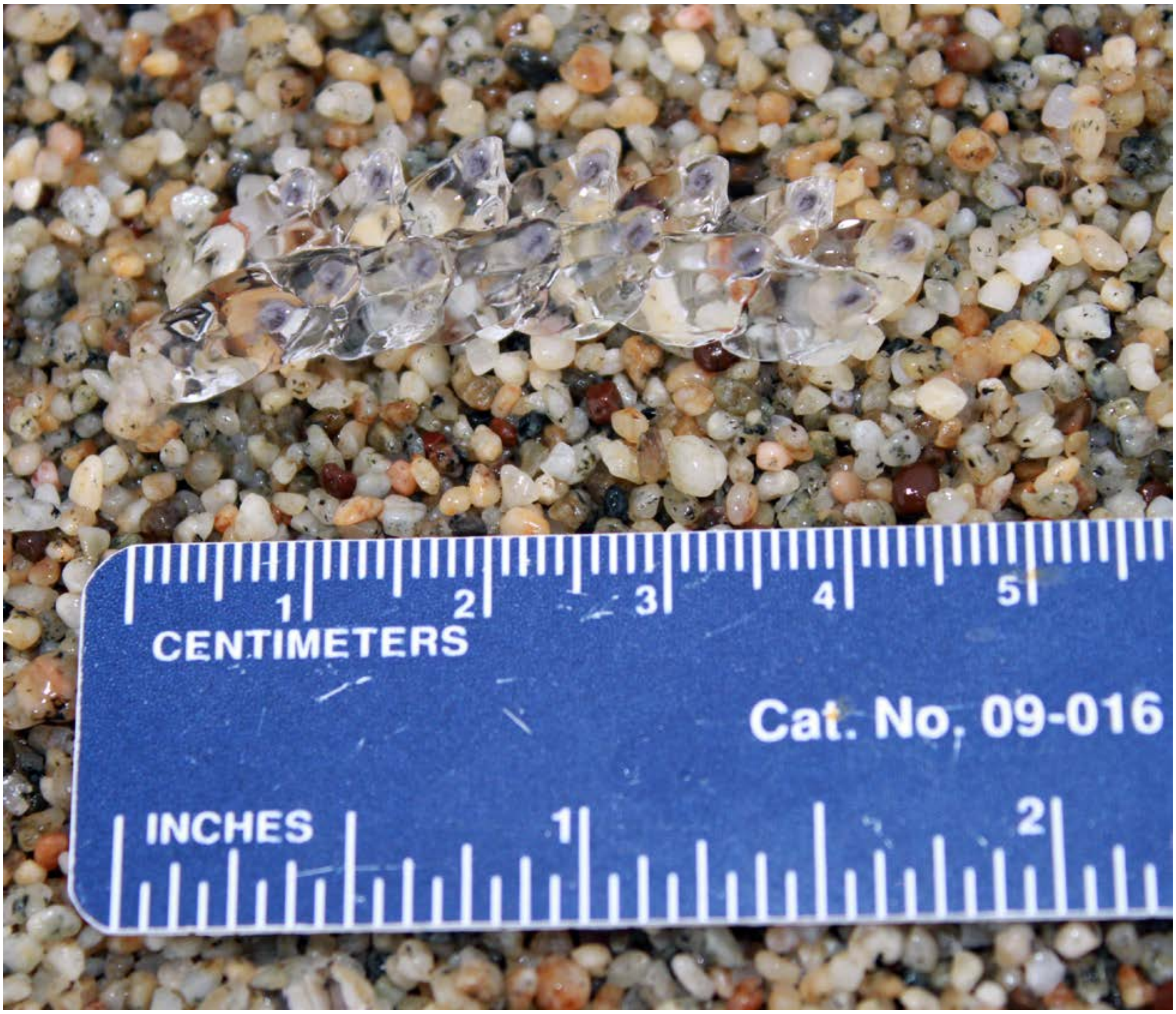

Figure S62. ***Thalia democratica*** **(Salp).** Photographed on 14 September 2014 at Horseshoe Cove (Bodega Marine Reserve), CA. Photo credit: Jacqueline Sones.

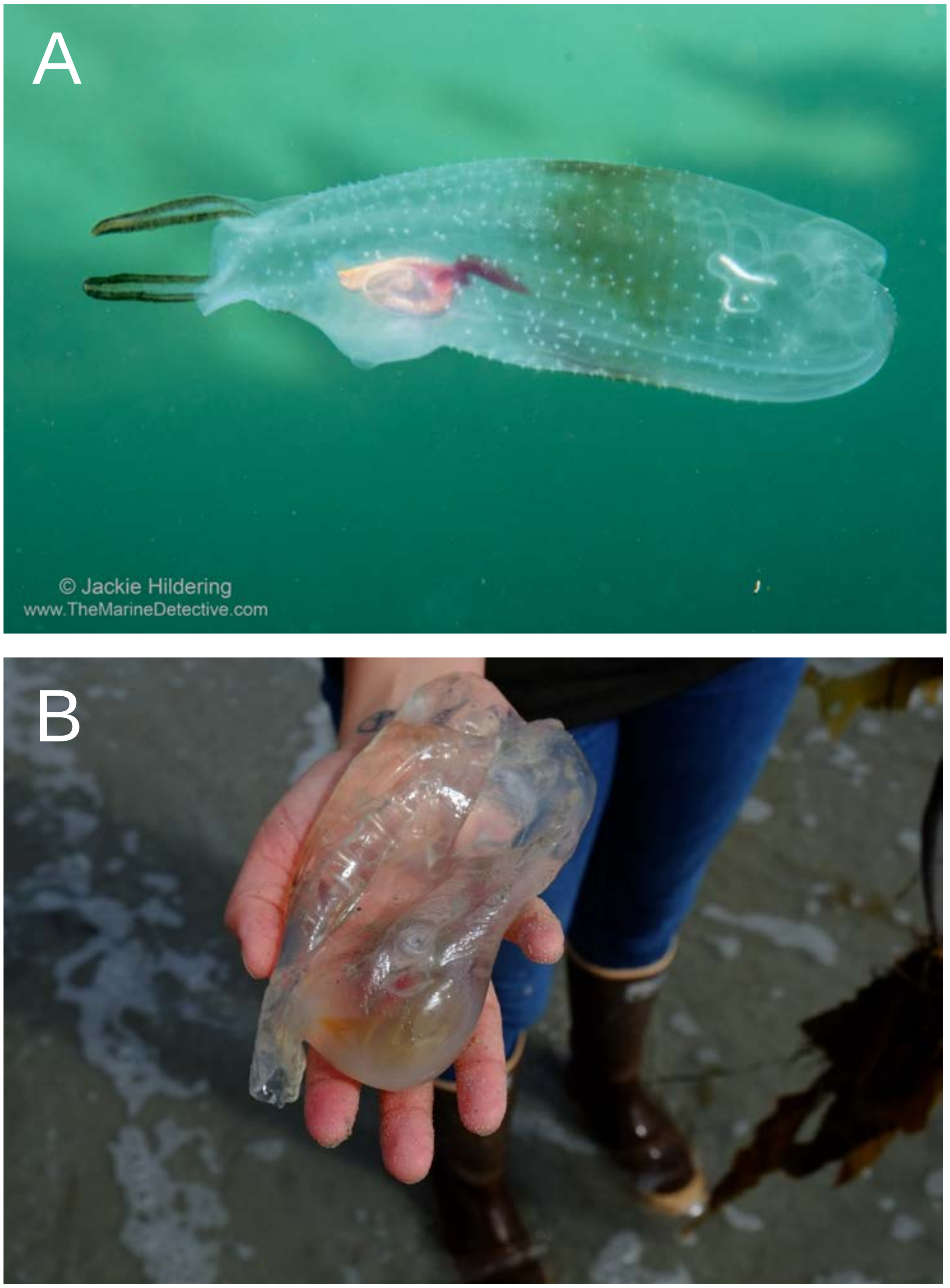

Figure S63. ***Thetys vagina*** **(Salp).** **(A)** Photographed on 15 February 2015 in the shallow subtidal zone near Port Hardy, British Columbia. Photo credit: © Jackie Hildering. **(B)** Photographed on 13 June 2017 at Calvert Island, British Columbia. Photo credit: Brian Starzomski.

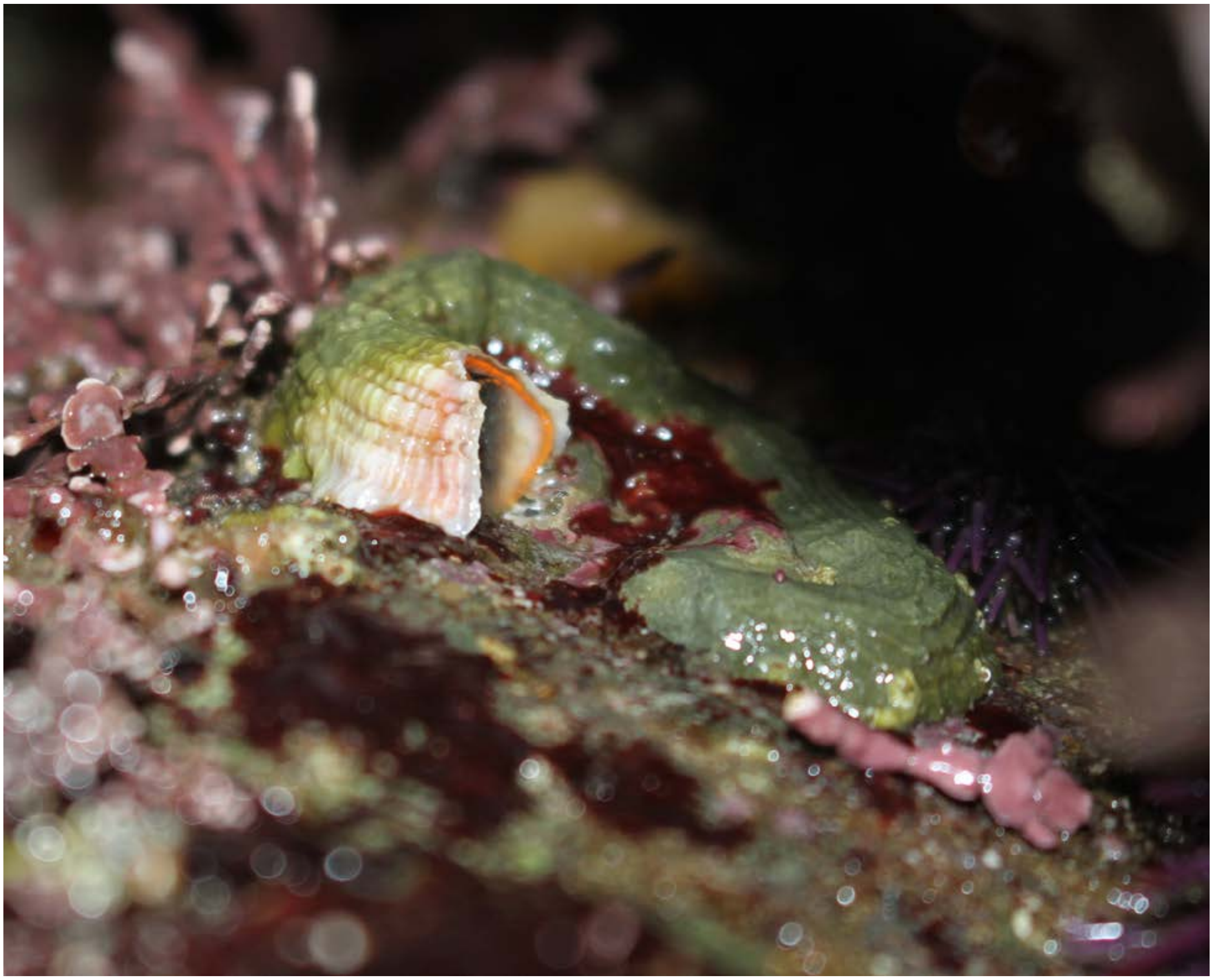

Figure S64. *Thylacodes squamigerus* (Scaled Tube Snail). Photographed on 25 February 2017 at Salt Point State Park, CA. Photo credit: Jacqueline Sones.

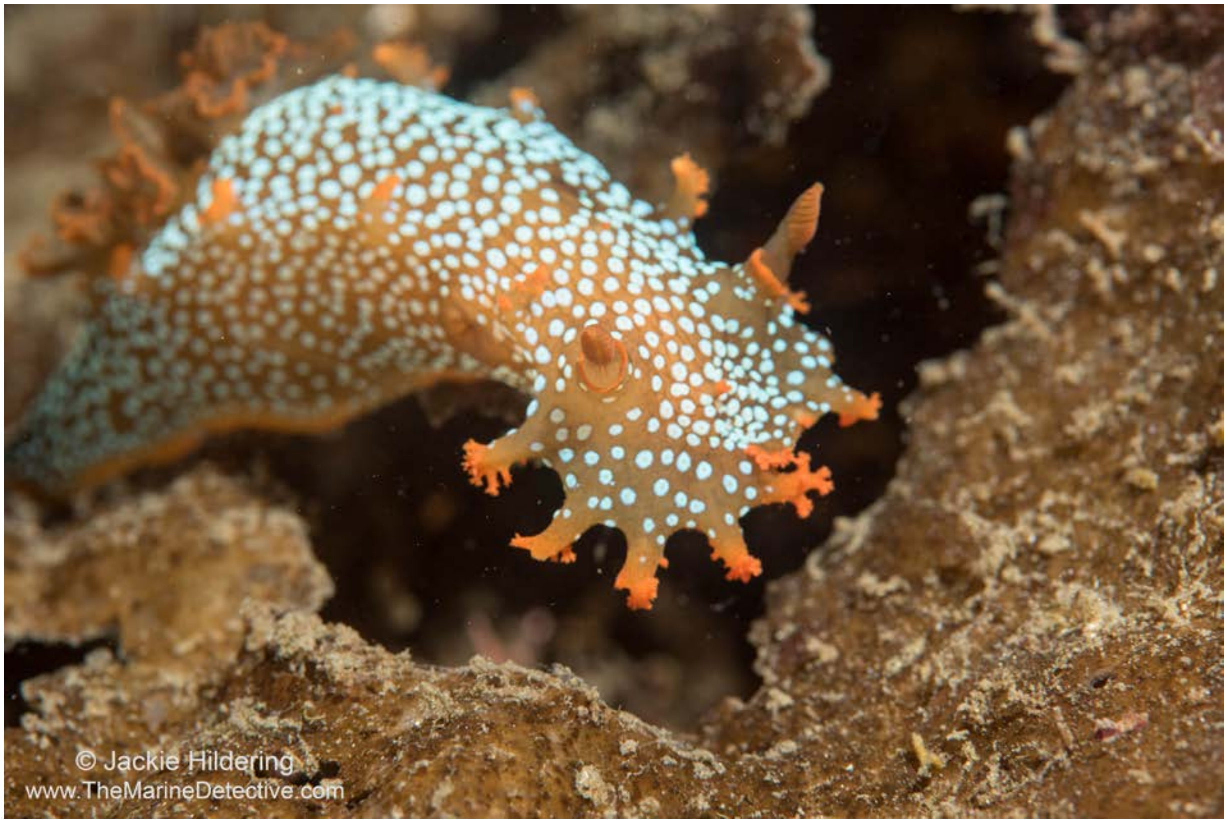

Figure S65. ***Triopha maculata*** **(Spotted Triopha Nudibranch).** Photographed on 18 September 2015 at Port Hardy, British Columbia. Photo credit: © Jackie Hildering.

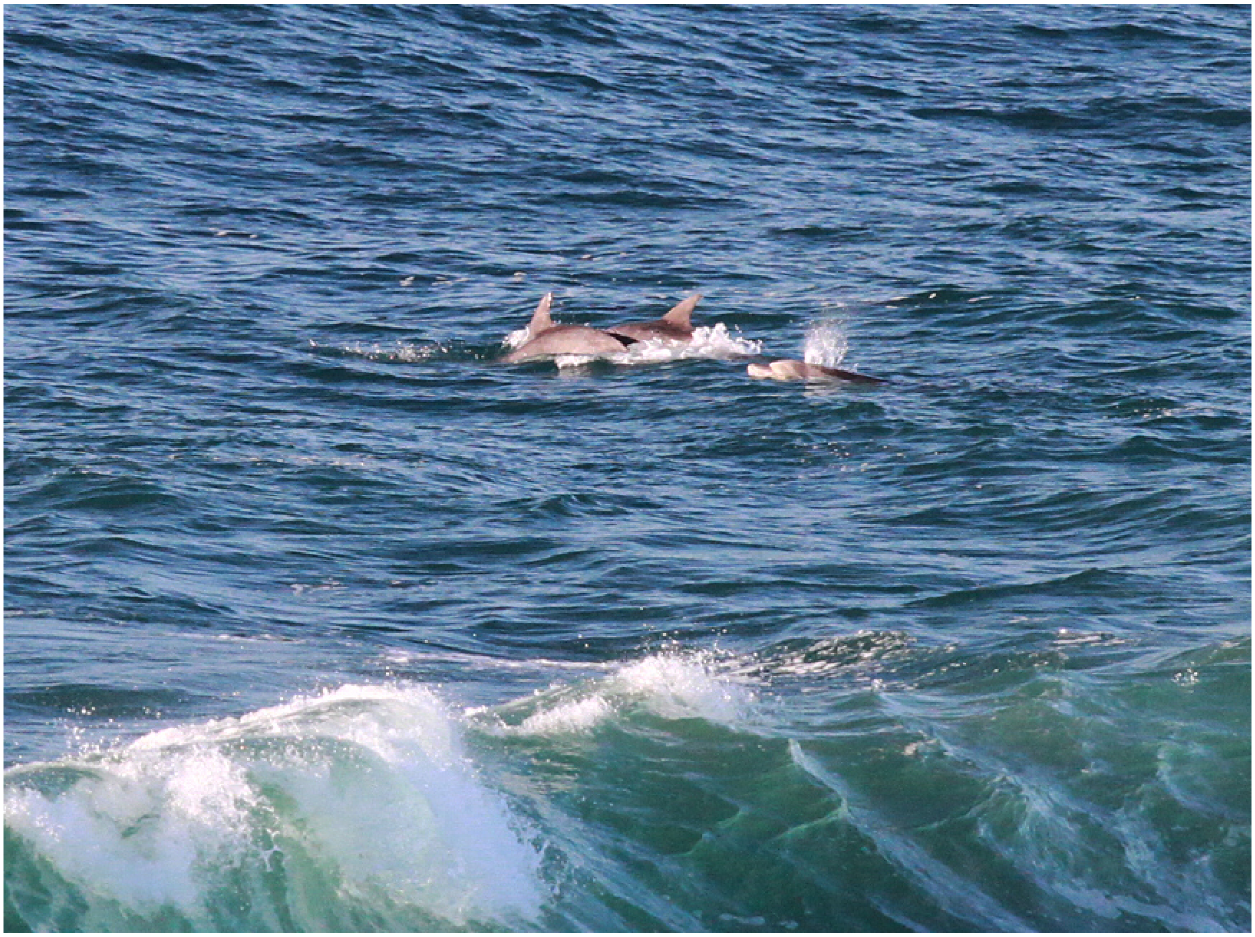

Figure S66. ***Tursiops truncatus*** **(Common Bottlenose Dolphin).** Photographed on 1 March 2015 off Salmon Creek Beach, CA. Photo credit: Jacqueline Sones.

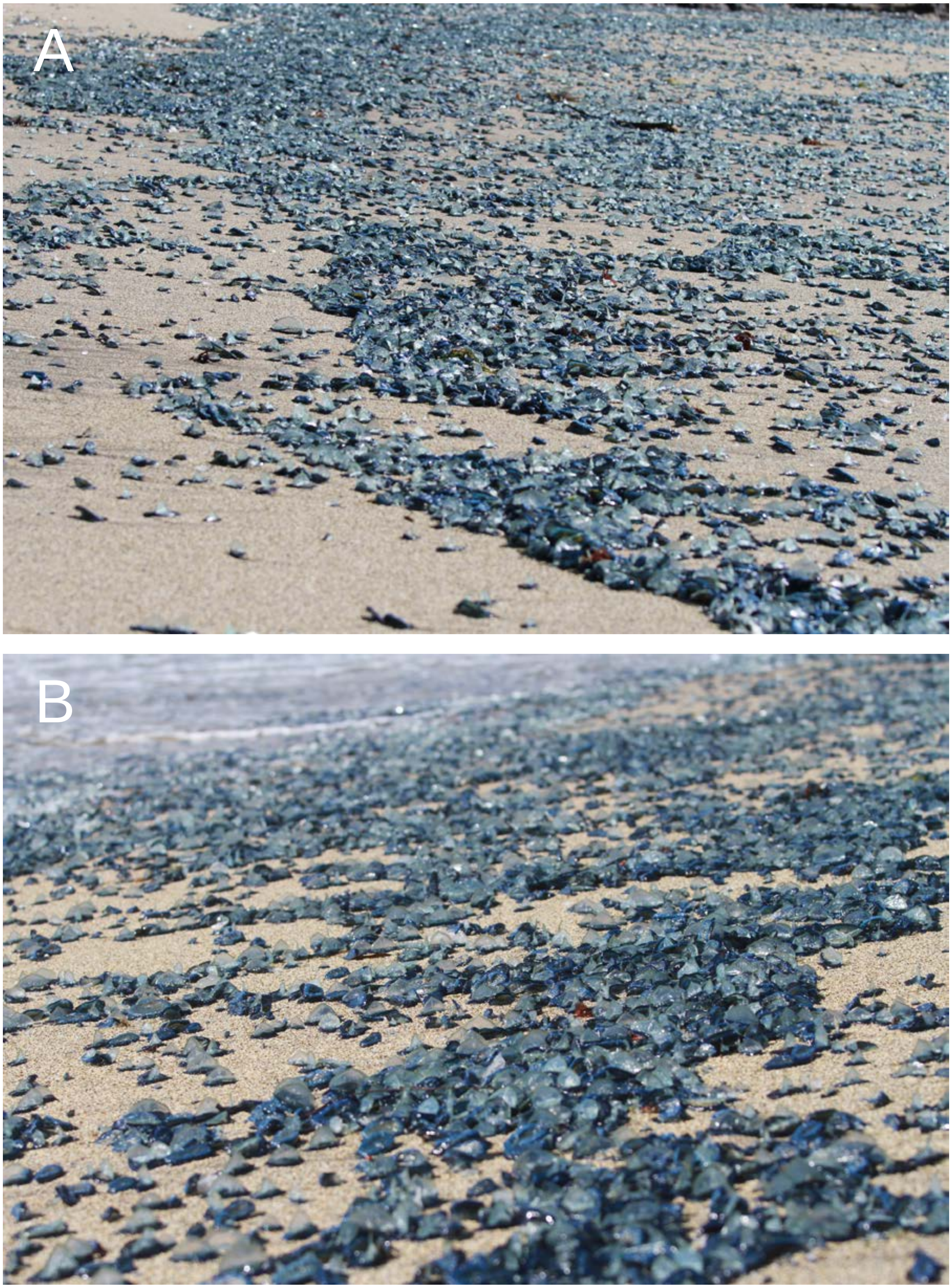

Figure S67. *Velella velella* (By-the-wind Sailor). Mass stranding of *Velella velella* photographed on 31 March 2015 at Horseshoe Cove (Bodega Marine Reserve), CA. Photo credit: Jacqueline Sones.

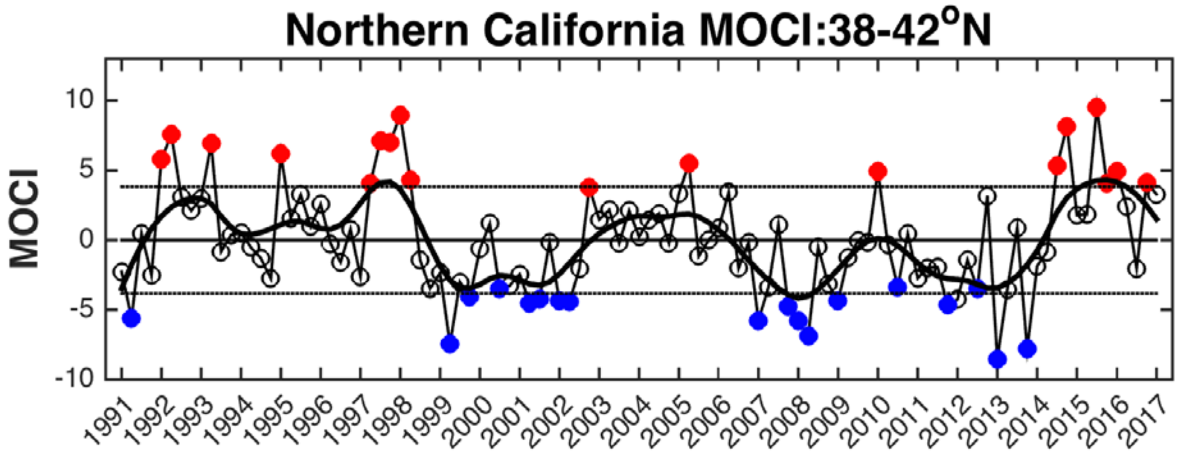

Figure S68. Multivariate Ocean Climate Indicator (MOCI) for northern California, from winter 1991 to winter 2017. MOCI synthesizes coastal ocean and atmospheric conditions as well as regional climate indices like ENSO and PDO (García-Reyes and Sydeman 2017). Positive (red) values indicate seasons that are warm and with weak upwelling, while negative (blue) values are cold conditions with strong upwelling. The 2014–2016 period shows many (non-continuous) seasons with warm conditions, only comparable to ENSO years in magnitude, although more persistent than ENSO. Warm conditions were broken by quasi-normal upwelling season conditions in northern California, but were more persistent in central and southern California.
